# A ghost moth olfactory prototype of the lepidopteran sex communication

**DOI:** 10.1093/gigascience/giae044

**Published:** 2024-07-19

**Authors:** Rui Tang, Cong Huang, Jun Yang, Zhong-Chen Rao, Li Cao, Peng-Hua Bai, Xin-Cheng Zhao, Jun-Feng Dong, Xi-Zhong Yan, Fang-Hao Wan, Nan-Ji Jiang, Ri-Chou Han

**Affiliations:** Guangdong Key Laboratory of Animal Conservation and Resource Utilization, Guangdong Public Laboratory of Wild Animal Conservation and Utilization, Institute of Zoology, Guangdong Academy of Sciences, Guangzhou, 510260, China; State Key Laboratory for Biology of Plant Diseases and Insect Pests, Institute of Plant Protection, Chinese Academy of Agricultural Sciences, Beijing, 100193, China; Shenzhen Branch, Guangdong Laboratory for Lingnan Modern Agriculture, Genome Analysis Laboratory of the Ministry of Agriculture, Agricultural Genomics Institute at Shenzhen, Chinese Academy of Agricultural Sciences, Shenzhen, 518120, China; College of Plant Protection, Shanxi Agricultural University, Shanxi, 030801, China; Guangdong Key Laboratory of Animal Conservation and Resource Utilization, Guangdong Public Laboratory of Wild Animal Conservation and Utilization, Institute of Zoology, Guangdong Academy of Sciences, Guangzhou, 510260, China; Guangdong Key Laboratory of Animal Conservation and Resource Utilization, Guangdong Public Laboratory of Wild Animal Conservation and Utilization, Institute of Zoology, Guangdong Academy of Sciences, Guangzhou, 510260, China; Institute of Plant Protection, Tianjin Academy of Agricultural Sciences, Tianjin, 300384, China; Henan International Laboratory for Green Pest Control, College of Plant Protection, Henan Agricultural University, Zhengzhou, 450046, China; Forestry College, Henan University of Science and Technology, Luoyang, 471000, China; College of Plant Protection, Shanxi Agricultural University, Shanxi, 030801, China; State Key Laboratory for Biology of Plant Diseases and Insect Pests, Institute of Plant Protection, Chinese Academy of Agricultural Sciences, Beijing, 100193, China; Shenzhen Branch, Guangdong Laboratory for Lingnan Modern Agriculture, Genome Analysis Laboratory of the Ministry of Agriculture, Agricultural Genomics Institute at Shenzhen, Chinese Academy of Agricultural Sciences, Shenzhen, 518120, China; Department of Evolutionary Neuroethology, Max Planck Institute for Chemical Ecology, Jena, D-07745, Germany; Guangdong Key Laboratory of Animal Conservation and Resource Utilization, Guangdong Public Laboratory of Wild Animal Conservation and Utilization, Institute of Zoology, Guangdong Academy of Sciences, Guangzhou, 510260, China

**Keywords:** genome, olfactory evolution, neuroecology, Lepidoptera, ghost moth, sex role

## Abstract

Sex role differentiation is a widespread phenomenon. Sex pheromones are often associated with sex roles and convey sex-specific information. In Lepidoptera, females release sex pheromones to attract males, which evolve sophisticated olfactory structures to relay pheromone signals. However, in some primitive moths, sex role differentiation becomes diverged. Here, we introduce the chromosome-level genome assembly from ancestral Himalaya ghost moths, revealing a unique olfactory evolution pattern and sex role parity among Lepidoptera. These olfactory structures of the ghost moths are characterized by a dense population of trichoid sensilla, both larger male and female antennal entry parts of brains, compared to the evolutionary later Lepidoptera. Furthermore, a unique tandem of 34 odorant receptor 19 homologs in *Thitarodes xiaojinensis* (*TxiaOr19*) has been identified, which presents overlapped motifs with pheromone receptors (PRs). Interestingly, the expanded *TxiaOr19* was predicted to have unconventional tuning patterns compared to canonical PRs, with nonsexual dimorphic olfactory neuropils discovered, which contributes to the observed equal sex roles in *Thitarodes* adults. Additionally, transposable element activity bursts have provided traceable loci landscapes where parallel diversifications occurred between *TxiaOr19* and PRs, indicating that the *Or19* homolog expansions were diversified to PRs during evolution and thus established the classic sex roles in higher moths. This study elucidates an olfactory prototype of intermediate sex communication from Himalaya ghost moths.

## Graphical Abstract

**Graphical Abstract:**
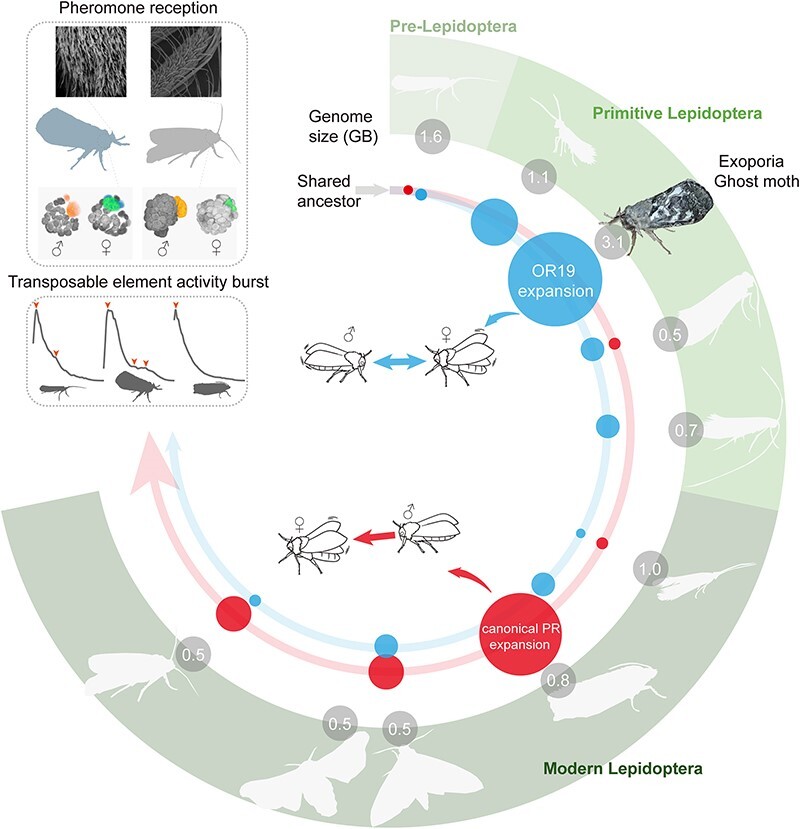

## Introduction

Sexual dimorphism is ubiquitous across the animal kingdom [1]. For most animals, mating by partner allocation is an indispensable process to ensure population continuity [2]. Sex roles often form under the pressure of sexual selection [3]. In general, the female exhibiting greater parental investment becomes a limiting resource for the less caring male so that the latter competes for accessing to the former [4]. In insects, sex pheromone becomes an effective investment for the male to gain opportunities to mate with the female successfully. One well-studied example is that, in fruit fly *Drosophila melanogaster*, males typically release a specific pheromone called *cis*-11-vaccenyl acetate (cVA) to gain an advantage in mating [5]. However, sex roles appear to be reversed in moths [6]. Female moths invest in synthesizing and releasing sex pheromones to attract male moths, and males have evolved distinct structures for sensing pheromones [7, 8]. Therefore, the study of pheromone and pheromone perception can expand our understanding of the evolution of sexual roles in animals.

One well-known animal lineage that relies on pheromone communication is Lepidoptera, comprising nearly 160,000 extant species and forming a key branch of insects [9]. Lepidoptera pheromones were well studied in the past decade, and most can be classified into type 0, I, II, and III, according to their hydrocarbon chains, double-bond allocations, and terminal functional groups [10]. Among them, type I pheromones, consisting of straight-chain acetates, alcohols, or aldehydes with 10 to 18 carbon atoms, make up 75% of all known sex pheromones and are employed by most moth families [11]. Pheromones are detected by pheromone receptors (PRs)/odorant receptor coreceptors (ORco) on the dendrites of olfactory sensory neurons. Based on the pheromone types, the corresponding PR family can be classified into type 0, I, and II clades [12]. However, the recent discovery of *Lampronia capitella* odorant receptor (OR) 6/ORco and *Spodoptera littoralis* OR5/ORco has revealed a novel “PR clade” that is distant from the type I PR clade [13, 14]. This implies that the mechanisms underlying the evolutionary process of ORs for detecting pheromones in Lepidoptera need to be explored.

The neural architectures of pheromone perception appear to be conserved in moths [15]. A typical perception of type I pheromone is achieved through a label-lined olfactory coding pattern in higher moths, such as Noctuidae. Pheromones are tuned by olfactory sensory neurons housed in sensilla trichoidae on the antennae. After the PR/ORco complex has been activated by the corresponding pheromone, the potential signals are projected to the primary olfactory center, the antennal lobe [16]. In Lepidoptera, the antennal lobe shows obvious sexual dimorphism. The male-specific macroglomerular complex (MGC) locates at the entry of the antenna and mainly processes pheromone signals [17]; in addition, in some species (e.g., *Cydia pomonella* and *Bombyx mori*), it also responds to plant volatiles [18, 19]. The counterparts of the MGC in females are usually called the large female glomeruli (LFG) that process oviposition and host-choosing signals, but LFG glomeruli are not generally enlarged in size as the MGC [20, 21].

The ghost moths (Hepialoidae: Hepialidae) from Exoporia are primitive Lepidoptera species and form an especially interesting lineage for studying the evolution of sex roles and pheromone communication [22]. Hepialids represent an early branch from the line leading to the heteroneuran Ditrysia, and the latter includes almost all the lepidopteran species that use typical PR-based olfaction for pheromones. Notably, the sex roles of Hepialidae species show diversity; for example, *Hepialus hecta* and *Hepialus humuli* exhibit courtship behavior that is very different from the usual moth pattern, as males hover in groups to attract females [22]. Moreover, ghost moths have undergone asymmetrical divergence of a duplicated gene (e.g., *zen* gene) to deliver functional alterations in subsequent species, providing insights into the evolutionary process of Lepidoptera [23]. *Thitarodes, Ahamus*, and *Hepialus* ghost moths, as the hosts of *Ophiocordyceps sinensis* medicinal fungus, are endemic to the Qinghai–Tibet Plateau [24]. The isolated ecological habitat and prolonged life cycle of these so-called Himalaya ghost moths provide ideal opportunities for the retention of pheromone receptive characteristics from shared ancestors of Lepidoptera [25–27]. While most previous research has focused on mating behaviors and pheromone identifications in hepialids [22, 28–34], few studies have explored their potential pheromone-sensing neural architecture or annotated the odorant receptor family in Hepialidae species.

In this study, we presented the unique evolutionary position of olfaction in ghost moths, characterized by comparative neurology and phylogenomics. Our results demonstrated that the antennal lobes of both male and female of 3 Himalaya ghost moth species (*Ahamus jianchuanensis, Thitarodes armoricanus, Thitarodes xiaojinensis*) have an enlarged antennal entry part and lack obvious sexual dimorphism, when compared to evolutionary later Lepidopterans. Comparative genomics further revealed that the ghost moth *T. xiaojinensis* expanded a specific *Or19* tandem array instead of the classic type I PR clade. Behavioral tests indicated that the ghost moth *T. xiaojinensis* exhibits similar sex roles between males and females in courtship, possibly due to their pheromonal neural architectures without sexual dimorphism and the specific *Or19* tandem array. In summary, this study uncovers a mechanism for the occurrence of functional ORs such as PRs through asymmetric divergence in Lepidoptera.

## Results

### Chromosome-level genome assembly of Himalaya ghost moth

A *T. xiaojinensis* (NCBI:txid1589740) larva was sequenced using Nanopore long-read technology, resulting in 319.9 Gb of clean reads. The draft genome of 3.1 Gb, comprising 1,645 contigs with a contig N50 of 5.4 Mb, was assembled using NextDenovo, corrected with minimap2 and NextPolish, and refined by removing contaminants. Utilizing Hi-C interaction data, the primary assembly was divided into 31,434 contigs, and 31,391 contigs (99.86% in length) were anchored to 32 chromosomes (Supplementary Fig. S1). BUSCO analysis revealed 91.8% complete genes in the final chromosome-level genome assembly, which was subsequently employed in downstream analysis.

The genome of *T. armoricanus* (NCBI:txid92013) was sequenced on Illumina HiSeq2000, yielding 877.7 Gb clean data for scaffolding. The final assembly presented a total length of 3,168 Mb for the scaffolds, with N50 of 27.8 kb and 176.2 kb for contigs and scaffolds, respectively. BUSCO analysis indicates 90% of single-copy insect orthologs were complete. We also conducted BUSCO analysis toward the transcriptomes of *A. jianchuanensis* (NCBI:txid92022), and a completeness of 96.6% was observed. Genome and transcriptomes were subsequently employed in downstream analysis.

### Evolutionary position of ancient Himalaya ghost moths based on phylogenomics analysis

**Figure 1:**
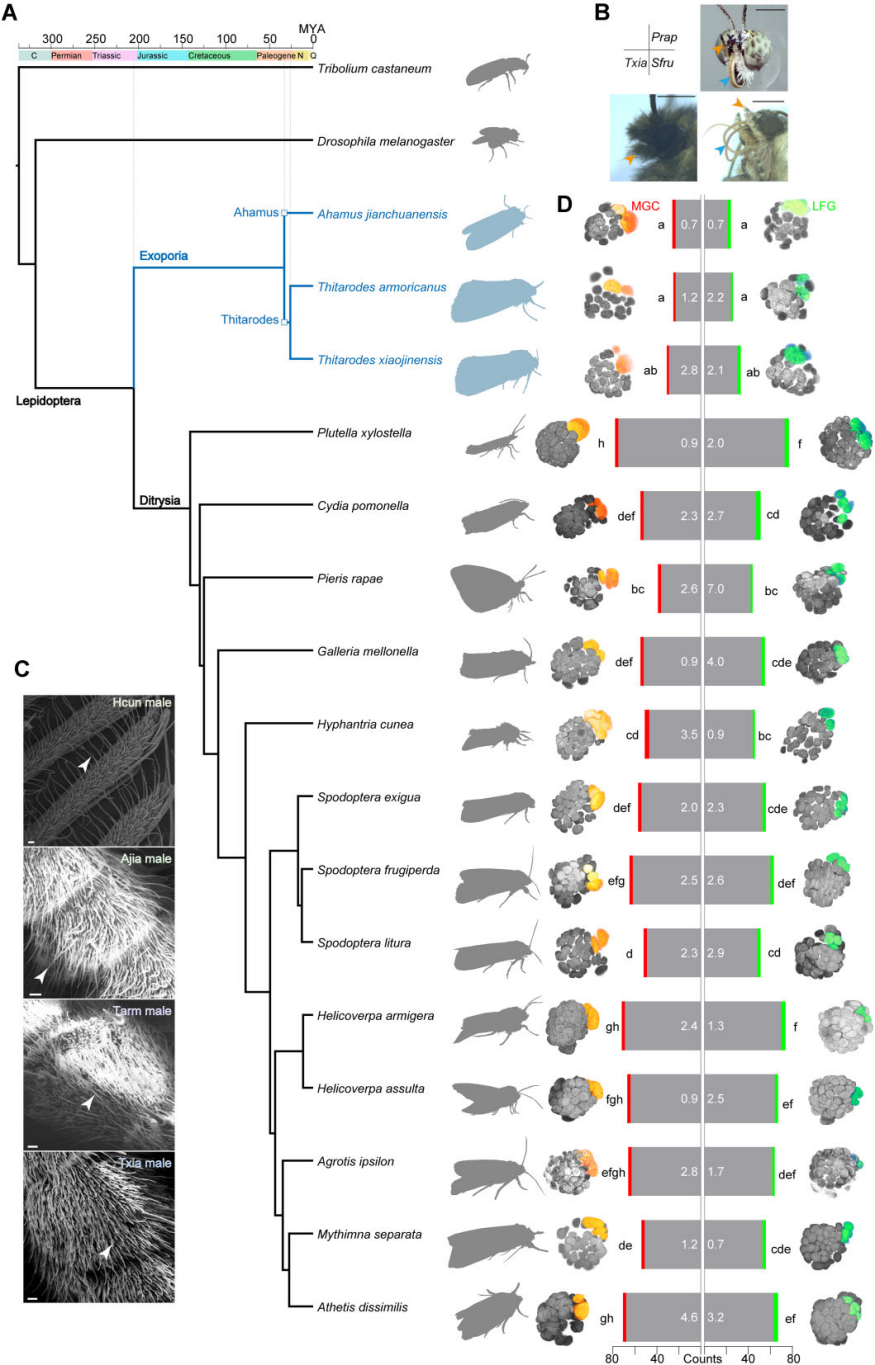
Phylogenomics and olfactory morphology of the ghost moths compared with other species in Lepidoptera. (A) Dated evolutionary tree of Lepidoptera relationships. Two of the nonlepidopteran species were placed on outgroup branches including *D. melanogaster* and *T. castaneum*. The tree was inferred through a maximum likelihood analysis of 634,106 amino acid sites from 1,547 strict single-copy genes employing the VT + F model and 1,000 bootstrap replicates. Branch lengths were optimized and node ages estimated using the penalized likelihood methods with the truncated Newton algorithm in r8s [56]. Scale bar is in millions of years. Data resources are listed in Supplementary Table S1. (B) Adult head development of Hepialidae *T. xiaojinensis* compared to moth *S. frugiperda* and butterfly *P. rapae*. Orange arrow indicates the labial palp. Blue arrow indicates the proboscis, which is lacking in the ghost moth. (C) Antennal sensilla morphology of selected Lepidoptera by scanning electron microscope. (D) Glomerular counts of tested Lepidoptera observed by confocal laser scanning microscopy system. Red bars indicate predicted male MGCs, and green bars indicate female LFGs. Numbers indicate standard errors of means. Lowercase letters indicate significant differences of glomerular counts among species (GLM and Tukey HSD, male: *F*_15, 32_ = 48.2, *P* < 0.0001, female: *F*_15, 32_ = 30.9, *P* < 0.0001).

We carried out a phylogenomics analysis based on genomes and transcriptomes of 3 ghost moth species, together with 13 Lepidoptera and 2 outgroups (data source see Supplementary Table S1). The separation of exoporian and ditrysian Lepidoptera occurred by the end of the Triassic Period, around 205 million years ago, while the speciation of the *Thitarodes* moths soon followed *A. jianchuanensis*, approximately 26 million years ago, by the end of the Paleogene Period. Although *Ahamus* and *Thitarodes* represented an ancient moth lineage, the species within were diverged in parallel with higher moths (Fig. 1A). We next asked what olfactory traits were maintained during the evolution of Himalaya ghost moths.

### Nonsexual dimorphic shortened antennae and enlarged glomeruli of Himalaya ghost moths

The Himalaya ghost moths had no observable proboscis but possessed intact antennae and labial palps (Fig. 1B). Additionally, we found that these moths had the shortest antennae among 32 lepidopteran families [35] (Supplementary Fig. S2), and their antennae were dominated by sensilla trichoidae (Fig. 1C, Supplementary Fig. S3).

The antennal lobe morphological atlas showed that 3 ghost moth species overall presented significantly less glomeruli (23 to 37), compared with other moths (45 to 80; Fig. 1D, Supplementary Data S1). Among 96 tested brains, the ordinary glomeruli arrangements in Himalaya ghost moths were distinguishable from those in the compared species. The families of Hepialidae, Pieridae, and Plutellidae had less intraspecies variations in glomerular arrangements compared to the other 4 higher moth families (Supplementary Fig. S4). The MGC consisted of 2 to 3 glomeruli in Himalaya ghost moths, and identical areas were confirmed in all tested species. Female LFGs was distinguishable in earlier species, including the Himalaya ghost moths and the diamondback moth *Plutella xylostella*, compared with the later species (Fig. 1D).

Volume proportions of the MGC and LFG glomeruli across tested species were compared. It showed that Himalaya ghost moths had both the largest MGCs and LFGs (Supplementary Fig. S5). The cumulus, which represents a major area, occupied 23.8% ± 5.5% of the antennal lobes in *A. jianchuanensis*, 15.9% ± 2.0% in *T. armoricanus*, and 19.5% ± 3.0% in *T. xiaojinensis*, respectively (Supplementary Fig. S5, Supplementary Data S1). The other species had relatively smaller MGC glomeruli in volumes (e.g., on average, 9.8% + 0.7% of the cumulus occupation for Noctuidae). As for females, LFG1 in 3 ghost moth species were significantly larger than those within higher moths (Supplementary Fig. S5, Supplementary Data S1). We checked the MGCs of 16 species in terms of volumes and shapes by utilizing a principal component analysis test, covering 73.8% for explained variances. Twelve of 16 lepidopteran species had a similar trend in MGC organizations, but the Himalaya ghost moths and *H. cunea* exhibited separated patterns, and especially *T. xiaojinensis* was totally isolated from higher Lepidoptera (Supplementary Fig. S6). We wonder if these structural specificities may reflect the genomic backgrounds and receptor repertoires in the Himalaya ghost moths.

### A unique large OR array on the ghost moth *T. xiaojinensis* chromosome

**Figure 2:**
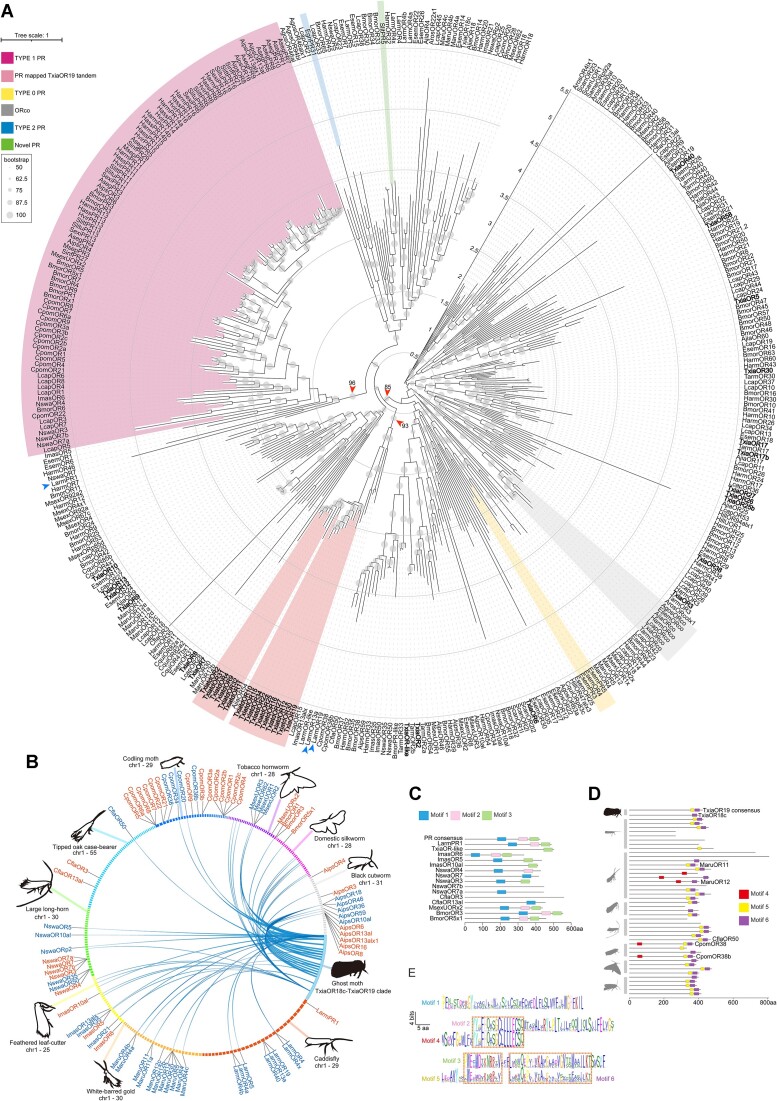
Evolution of sex pheromone–related odorant receptors among *T. xiaojinensis*, caddisfly, and other Lepidoptera. (A) Rooted maximum likelihood (ML) tree of 387 selected lepidopteran ORs, which included reported ORs of moths, mapped ORs in the linearization analysis in (B) by TxiaOR19 tandem, and ORs obtained from prelepidopteran species by blasting with TxiaOR19 tandem against the nr database (Supplementary Data S4). The evolutionary distances were computed using the “Auto” option in IQ-TREE [68] with ultrafast [69] 1,000 bootstraps and the Shimodaira–Hasegawa–like approximate likelihood ratio test [70]. Tested species included *A. jianchuanensis* (Ajia), *T. armoricanus* (Tarm), *T. xiaojinensis* (Txia), *P. xylostella* (Pxyl), *C. pomonella* (Cpom), *B. mori* (Bmor), *S. exigua* (Sexi), *S. litura* (Slitu), *S. littoralis* (Slit), *Heliothis virescens* (Hvir), *H. armigera* (Harm), *H. assulta* (Hass), *Ectropis grisescens* (Egri), *Operophtera brumata* (Obru), *Agrotis segetum* (Aseg), *Eriocrania semipurpurella* (Esem), *Lampronia capitella* (Lcap), *Limnephilus marmoratus* (Larm), *Micropterix aruncella* (Maru), *Incurvaria masculella* (Imas), *Nematopogon swammerdamellus* (Nswa), *Coleophora flavipennella* (Cfla), *Manduca sexta* (Msex), *A. ipsilon* (Aips), *Athalia rosae* (Aros), *Hhermetia illucens* (Hill), *Culex quinquefasciatus* (Cqui), *Aphis gossypii* (Agos), *Schistocerca americana* (Same), and *S. cancellata* (Scan). Tree topology was cross-checked with neighbor-joining and Bayes approaches (Supplementary Fig. S9). Red arrows indicate key bootstrap values related to type I PRs and TxiaOR19 tandem. Blue arrows indicate ancestral ORs from caddisflies related to type I PRs and TxiaOR19 tandem. (B) Linearization of TxiaOR18c–TxiaOR19 tandem proteins with chromosomes from selected species showed by circos plot. Reported chromosome assemblies (chr) from *L. marmoratus, M. aruncella, I. masculella, N. swammerdamellus, C. flavipennella, C. pomonella, M. sexta, B. mori*, and *A. ipsilon* were used (Supplementary Table S1). ORs mapped by TxiaOR18c or TxiaOR19 tandem were colored in blue, and those PR mapped were colored in red. (C) Motif identification toward PR mapped ORs from caddisflies and moths. (D) Motif identification toward TxiaOR18c/19 tandem mapped ORs from caddisflies and moths. (E) Distribution of motifs identified in (C) and (D), showing overlaps from PR and TxiaOR18c/19 clades.

A total of 23 *TxiaOrs* were confirmed to be expressed in the antennae of *T. xiaojinensis* via reverse transcription PCR verification, out of annotations from genome and antennal transcriptome assembly (Supplementary Fig. S7, Supplementary Data S2). This number was higher than those found in *A. jianchuanensis* (10) and *T. armoricanus* (16) (Supplementary Table S2). Notably, a large array comprising 34 tandem duplications was mapped by higher moth PRs on chr14 of the chromosome-level assembly of *T. xiaojinensis*. This array, homologous to *TxiaOr1*9, contained 16 homologs (*TxiatdOrs*) and 18 pseudogenes (*TxiatdpOrs*), which maintained the largest tandem duplications reported in lepidopterans (Supplementary Fig. S8, Supplementary Data S2). The *TxiaOr19* array was located on the same chromosome with an upstream *TxiaOr18c*, which mapped to Noctuidae homologs [36]. Maximum likelihood phylogeny analysis using 387 ORs showed that the *TxiaOr19* array joined an earlier group in which canonical type I PRs arose (Fig. 2A, Supplementary Data S3). The earlier separation of canonical PRs and TxiaOR19 tandem was also observed when cross-checked with the neighbor-joining and Bayes method (Supplementary Fig. S9). A female-biased expression pattern was observed in *TxiatdOr15* and *TxiatdOr25* of the 16 *TxiaOr19* homologs (Supplementary Fig. S10). Differing from ORs found in evolutionarily later moths, the TxiaOR19 array could blast to ORs in locusts, aphids, soldier flies, mosquitoes, and fleas, with homologs predicted by CLAN [37] (Fig. 2A and Supplementary Figs. S9, S11). However, the male-biased TxiaOR7 failed to blast to any ORs from those earlier species (Supplementary Figs. S11, S12).

We investigated the evolution of the *TxiaOr19* array by mapping it to chromosomes of caddisflies, as well as primitive and higher moths, and cross-checking with canonical PR mapped regions (Fig. 2B). The results indicated that tandem ORs were identified in linearized regions of almost all species, except for *C. flavipennella* and *B. mori*. Some of these linearized regions did not contain ORs that could be annotated using FGENESH [38]. Mapped ORs from linearization analysis and nr blast ORs by TxiaOR19 tandem were used in a Bayesian phylogenic analysis, which showed that canonical PRs and a single LarmPR1 in the caddisfly formed a clade in the phylogeny (Fig. 2A, Supplementary Fig. S13), and expansions of PR tandems were consistently observed within species after the Himalaya ghost moths, except for *C. flavipennella* (Fig. 2B, Supplementary Fig. S13). Notably, regions containing *TxiaOr19* and PR had mixed tandem patterns in the early moths succeeding *T. xiaojinensis* but tended to be separated in later species (Fig. 2B). Bayesian phylogenetic analysis showed that the homologs of *TxiaOr19* underwent significant diversification from their ancestors (Supplementary Fig. S13). Specifically, *TxiaOr19* array formed the same clade with *LarmOr19-Or13a* tandem (Fig. 2A, Supplementary Fig. S13). The majority of PR-mapped ORs formed a single cluster that possibly diverged from LarmPR1, with a few remaining in the TxiaOR19 mapped phyla (Fig. 2A, Supplementary Fig. S13).

Using MEME suite, we found that LarmPR1 exhibited all 3 signature motifs of PR consensus regions as reported before [12], indicating the potential emergence of canonical PRs prior to the evolution of lepidopteran insects (Fig. 2C). However, most PR-mapped ORs from non-Ditrysia primitive moths did not meet the requirements of canonical PR motifs (Fig. 2C). On the other hand, the majority of TxiaOR19-mapped ORs had 2 motifs, with some having 3 motifs (named motifs 4–6) upon checking with the same approach (Fig. 2D). Notably, motifs 4–6 from TxiaOR19 homologs showed overlaps with motifs 2–3 from PR homologs, suggesting the existence of a common ancestor for TxiaOR19 and canonical PRs at an earlier evolutionary stage (Fig. 2E).

### Transposable elements involved in the evolution of ORs

**Figure 3:**
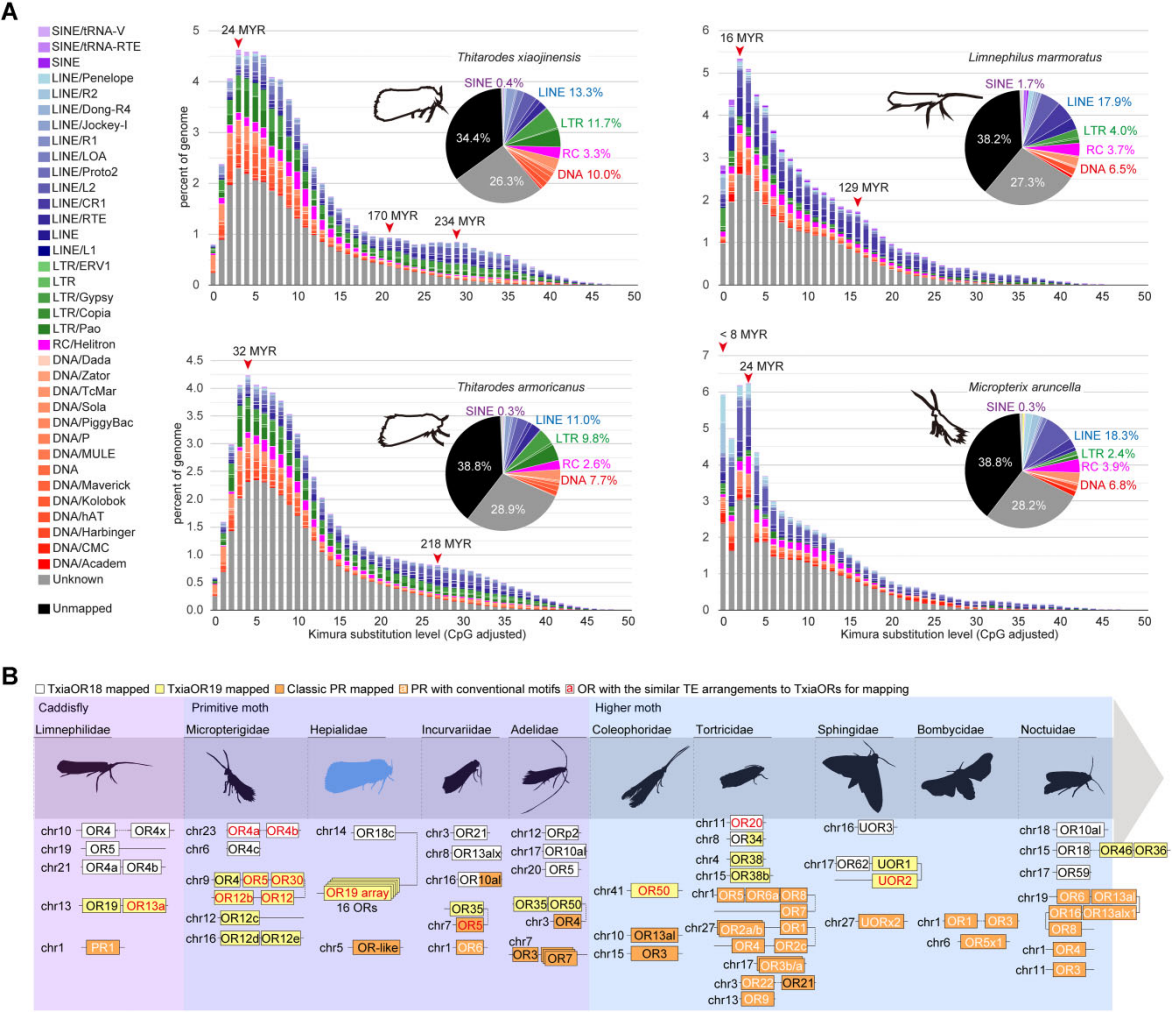
Genome and OR evolution reflected by landscapes of transposable elements (TEs). (A) Detailed TE landscapes of the ghost moths, caddisflies, and white-barred gold. Times of TE burst events were estimated according to CpG-adjusted Kimura substitution levels and a reported arthropod substitution rate of 6.19 × 10^−10^ per site per generation [76]. Red arrowheads indicate TE burst events. (B) Overview of asymmetric divergence of duplicated pheromone-related ORs from caddisfly to higher Lepidoptera. The TxiaOR18c/19 duplications predominate in caddisflies and primitive moths, but they were replaced by functional PR duplications in higher moths during evolution.

Tandem gene duplications and chromosome linearization patterns have been reported to be associated with transposable element (TE) activities [39]. To explore how a large and specific OR array was formed in the ghost moth *T. xiaojinensis*, we characterized the landscape of TEs in the genomes of above species. It showed that 2–3 TE burst events occurred in the Himalaya ghost moths. These bursts likely took place around the same time as the successive divergences of Exoporia and *Hepialus* (Figs. 1A and 3A). Caddisflies and primitive and modern Lepidoptera experienced more recent bursts of TE activity compared to Himalaya ghost moths (Fig. 3A, Supplementary Fig. S14). Specifically, various TE arrangements were observed in previously identified OR loci. The TE arrangements of the *TxiaOr19* tandem were correlated with that of the *LarmOr13a* (Supplementary Fig. S15). Furthermore, the TE landscapes in the *TxiaOr18/19* homologs were found to be similar mostly with *Ors* from non-Ditrysia species and later diversified in higher moths (Supplementary Fig. S15).

In conclusion, our findings suggest that both the TxiaOR19 tandem and PR clusters had already emerged in caddisflies. The OR19 lineage underwent expansion within the ancestral moth lineage, leading to the formation of a large duplicated tandem in *T. xiaojinensis*. Furthermore, linkages between OR19 and PRs were observed in species predating Ditrysia. In later higher moths, PRs became predominant, while the OR19 cluster contracted through asymmetric diversifications of their homologs, as supported by the similar TE arrangements (Fig. 3B).

### Equal sex roles of ghost moth *T. xiaojinensis* adults

The replacement of the TxiaOR19 duplications with canonical PRs suggests possible different tuning characteristics of TxiaOR19 from a PR that narrowly tunes to sex pheromone components. To confirm this assumption, we first analyzed the emissions of adult *T. xiaojinensis* using both solvent extraction and solid-phase microextraction (SPME) methods. We found that male and female adults were not distinguishable by tracing the volatile blends in abdomen tip extractions. However, SPME samples collected within the first 24 hours after female emergence exhibited a significant peak corresponding to oleamide (Supplementary Fig. S16A). We performed successive docking simulations using the TxiaOR19 array and 4 sex-biased TxiaORs against the identified major components. The results showed that the TxiaOR19 array had less binding affinity toward the panel of ghost moth emissions, and the responding spectrum was relatively broad for all 16 tandem ORs (Supplementary Fig. S16B).

**Figure 4:**
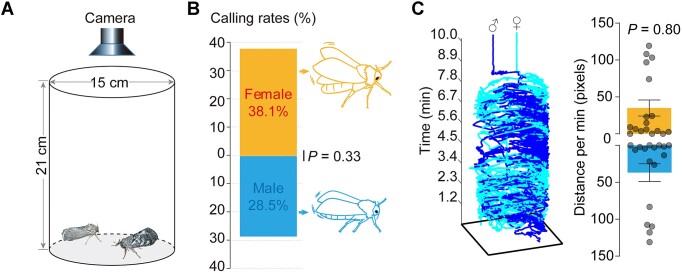
Resulting dual attraction in sex communications of the ghost moth adults. (A) Schematic shows setup of the courtship arena of *T. xiaojinensis* adults. (B) Comparison of calling rates, which were reflected by fluttering behaviors in both sexes (binary test against even distribution, *P* = 0.33). (C) Left shows representative behavioral traces of male and female adults tracked by idTracker [81]. Right shows comparison of distance per minute between male and female *T. xiaojinensis* adults (Mann–Whitney test, *U* = 137, *P* = 0.8055).

Considering ghost moths, including *T. xiaojinensis*, lack sexual dimorphism in their antennal lobe (Fig. 1D), we hypothesized that *T. xiaojinensis* may also exhibit unconventional calling and mating behaviors compared to higher moths. We then tested adult pairs of *T. xiaojinensis* in a courtship arena (Fig. 4A). Unlike the female calling behaviors of higher moths with wing beats and extruded pheromone gland, calling behavior of this ghost moth involved hovering with wing beats. Females fluttered with substantial wing beats, while males fluttered by small-range vibrating-like wing beats (Fig. 4B). Interestingly, male and female adults exhibited similar amounts of calling behaviors and tracing velocities (Fig. 4B, C). Our results indicated that the sex roles of *T. xiaojinensis* adults differed from those of higher lepidopterans during mating allocation, which may be attributed to the predicted unconventional TxiaOR19 tandem, noncanonical female emission, and olfactory architectural observations.

## Discussion

Himalaya ghost moths offer a basal model for the study of olfaction evolution in Lepidoptera due to their unique olfactory system, limited distribution, and possession of the largest genomes in Lepidoptera. Our study has successfully generated the first chromosome-level assembly for this lineage, providing valuable insights into the genetic characteristics of these ghost moths. We have discovered that the OR evolutionary pathway in ghost moth *T. xiaojinensis* parallels with that of modern moths and identified molecular traces that reveal the origins of the modern pheromone sensory system. Interestingly, the presence of unique olfactory structures, including enlarged glomeruli observed in both sexes and dominated long trichoid sensilla, mostly nonbiased expressions of *TxiaOr19* homologs, and their nonfeeding life traits in adulthood, suggests that these ghost moths may employ a primitive pheromone sensing system to locate potential partners.

Why the lineage of Himalaya ghost moths keeps primitive may be due to their isolated habitats, uneven long life cycle for larvae (3–6 years in nature), and a brief adult stage (several days) [26, 27], which greatly reduces the evolution speed of this lineage. They have developed a unique evolutionary strategy of focusing solely on reproduction in adulthood while forgiving foraging [40]. The redundancy of their giant genomes is an example of the basal genomic features possessed by the ghost moths [41]. Asymmetrically diverged duplications and frequent TE activity bursts have played a critical role in both PR formation and the emergence of other functional genes in Lepidoptera [23]. As a result, the expanded OR19 homologs in primitive species were later diversified into canonical PRs, establishing the advanced sex pheromone–based communication system. One interesting result in our molecular docking predictions is that the male-biased OR7 shows a higher affinity to oleamide, suggesting that TxiaOR7 potentially serves as an ancestral PR of the novel PR clade in lepidopteran species. However, more experimental functional evidence needs to be provided for both TxiaOR19 array and TxiaOR7 to support their evolutionary roles in lepidopteran species.

Canonical PRs of ancient moth species were broadly characterized [13, 43]. In this study, we showed that the TxiaOR19 array and LarmPR1 share motifs. These motifs were likely to be already separated before Lepidoptera and Trichoptera diverged from each other. Considering that motif shifts accompany similar transposable element arrangements within the tested OR loci, it is likely that the first PR emerged from exonization, driven by TE activities. This effect has been commonly observed in other organisms [44]. On the other hand, PRs shared motif regions with TxiaOR19 and the later could be blasted to ORs in dipteran species, where large OR duplications existed, such as in *Bactrocera dorsalis* [45]. The disappearance of large tandem arrays in later lepidopterans suggests the separation of ancestral duplicated ORs. This can be supported by scattered chromosome linearization and increased DNA transposons during TE activity bursts. The duplicated ORs themselves could reflect rapid olfactory evolution for species adaptation [46]. Although TEs may not be determining factors for the functional emergence of ORs, as shown in the clonal raider ant *Ooceraea biroi* [47], we cannot exclude possible TE involvement in PR emergence due to the horizontally diverged TE landscapes among lepidopteran ORs.

Evolutionary later moths show larger interspecies variations and increased numbers of glomeruli in antennal lobes, suggesting potential positive selection in olfactory systems, along with their distribution in different ecological niches [48]. The enlarged MGCs in the butterflies, such as *Pieris rapae* and *Godyris zavaleta* [49], suggest that the sexual dimorphic brain organizations are widely presented by Lepidoptera, but the link between morphology and pheromone recognition in butterflies requires further examination. On the contrary, the sexual dimorphic olfactory neuropils are not suitable for Himalaya ghost moths, as females have also retained the enlarged LFGs. We argue that female LFGs are more likely involved in mating allocation rather than egg-laying orientation since these ghost moths spray eggs in nature, unlike the majority of modern moth egg-laying behavior in the selected locations [40, 50]. Therefore, they may not require a sophisticated olfactory system for precise assessment of corresponding sites. This is also supported by our behavioral assays in the *T. xia* that both male and female were moving to find partners. Given their ecological traits, highly redundant genome, and a smaller number of ORs, we believe that these Himalayan ghost moths may retain pheromone-sensing abilities and exhibit primitive sex roles within Lepidoptera. Further investigation into the function of ORs remains crucial for refining this perspective.

Himalaya ghost moths retain ancestral traits of olfactory system for equal sex roles, which may be attributed to the mechanisms of asymmetric divergence and redundant genome formation. The lack of sexual dimorphism in the antennal lobes and expansion of TxiaOR19 array other than canonical PRs also contribute to the nonbiased sex role differentiation within this primitive lineage. Overall, these findings highlight the unique evolutionary features of Himalaya ghost moths and shed light on the mechanisms shaping olfactory systems in insects.

## Materials and Methods

### Insects

Newly emerged lepidopteran species from lab colonies were sexed, and 3- to 5-day-old adults were used in all tests. *A. jianchuanensis, T. armoricanus, T. xiaojinensis, Agrotis ipsilon, S. frugiperda, Galleria mellonella*, and *P. rapae* were obtained from Institute of Zoology, Guangdong Academy of Sciences. *H. cunea* was obtained from Chinese Academy of Forestry. *Athetis dissimilis* was obtained from Henan University of Science and Technology. *Helicoverpa armigera* and *Helicoverpa assulta* were obtained from Henan Agricultural University. *Mythimna separata, C. pomonella*, and *S. litura* were obtained from the Institute of Plant Protection, Chinese Academy of Agricultural Sciences. *S. exigua* was obtained from Qingdao Agricultural University. *P. xylostella* was obtained from Shanxi Agricultural University. All lab colonies were regularly rejuvenated with natural populations.

### Morphometric measurement

A total of 7 strains of Himalaya ghost moths were measured for the lengths of antennae and forewings. Three *T. xiaojinensis* strains were from the lab colony mentioned above, as well as Xiaojin (N30.99, E102.27), Hongkou (N31.16, E103.84) field populations. The other 4 strains consisted of 2 *A. jianchuanensis* populations collected from Jiulong (N28.99, E101.51), Gongga (N29.56, E101.98) and 2 *T. armoricanus* populations from Yala (N30.11, E102.25), Kangding (N30.08, E101.97), respectively. Intact appendages were removed and embedded on glass slides before being processed under an AXIO Imager microscope (Zeiss) equipped with an Axiocam 512 camera (Zeiss). A ZEN 2.3 software (Zeiss) was used to acquire scale bar labeled photographs of antennae and wings. Lengths of interest were manually assigned to the scale bar using ImageJ 1.53f51 (National Institutes of Health) and then recorded. A total 5 to 21 replicates were carried out for each strain, and means were used to develop olfactory indexes using the formula [antenna/wing]. Data from other species were referred to the previous publication [35].

### Scanning electron microscopy

The antennae of 1- to 3-day adults were cut from the base and fixed in 0.25% glutaraldehyde at 4°C overnight. After 3 washes at room temperature with 0.1 M phosphate-buffered saline (PBS, pH 7.4) for 15 minutes each, the antennae were dehydrated through a laddered ethanol series (30%, 50%, 70%, 80%, 90%, and 100%) for 15 minutes each and dried for 15 minutes in a critical point drier (Bal-Tel CPD 030) before being mounted on aluminum stubs. The mounted antennae were coated with gold spray (Bal-Tel SCD 005) and observed with an SEM instrument (FEI Quanta 200).

### Antennal lobe atlas

Lepidopteran brains were labeled according to previous work [21]. Newly dissected intact brains were successively processed with 4% paraformaldehyde in 0.1 M PBS for fixation (24 h), preincubating with 5% normal goat serum in 0.1 M PBS containing 0.5% Triton X-100 (NGS-PBST) (0.5 hours), incubating with 1% SYNORF1 (Developmental Studies Hybridoma Bank, University of Iowa) in 5% NGS-PBST (72 hours), and incubating with Alexa Fluor 488 goat anti-mouse (Invitrogen) at 1:500 with 1% NGS-PBST (48 hours). After being rinsed 6 times in PBS and dehydrated with a laddered ethanol series, brain samples were mounted with antifade mounting medium (Beyotime) in perforated aluminum slides that was sandwiched by 2 glass coverslips. Three brains of each sex from each species were prepared for imaging.

All image stacks were acquired with a confocal laser scanning microscopy system with a 10–20× objective. Data for *A. jianchuanensis, T. armoricanus, T. xiaojinensis, G. mellonella*, and *A. ipsilon* were collected with FV3000 (Olympus). Data for *H. cunea, A. dissimilis, H. armigera, H. assulta, M. separata, S. litura*, and *P. xylostella* were collected with LSM 780 (Zeiss). Data for *S. frugiperda, P. rapae, C. pomonella*, and *S. exigua* were collected with A1 HD25 (Nikon). An argon laser at 488 nm was used to excite the Alexa Fluor. The resolution of the x-axis was set to 500–2,048 voxels and the section interval was set to 3 or 5 μm. Amira software (AMIRA 5.3; Visage Imaging) was used as previously described to conduct segmentation, tissue statistics, and 3-dimensional reconstructions of the antennal lobes [21].

### Genome and transcriptome sequencing

Genomic DNA of *T. xiaojinensis* larva was extracted for library establishment and then sequenced with Nanopore PromethION platform (Oxford Nanopore Technology). After quality control, a total 319.9 Gb clean data were assembled by using NextDenovo (RRID:SCR_025033). Meanwhile, a short-reads library was constructed by Illumina platform with the same batch of *T. xiaojinensis* DNA, and 165 Gb raw data were generated. After filtering, the remaining clean reads with Q > 20 were used for minimap2 mapping onto the genome assembly, which was later polished by NextPolish (RRID:SCR_025232). To remove the DNA contamination from the other organisms, the polished genome was aligned against the NCBI nucleotide (NT) database, and the contigs that were aligned to the sequences from fungi, plants, or virus were removed.

To obtain a chromosome-level assembly, Hi-C scaffolding was further carried out with the same larval sample following reported protocols [51–53]. Specifically, samples were fixed using 2% formaldehyde to establish cross-links, followed by cell lysis and sample quality assessment through extraction. Chromatin digestion was carried out using a restriction endonuclease, with enzyme cleavage efficacy evaluated through sampling. Subsequent steps included biotin-14-dCTP (Invitrogen) labeling, blunt-end ligation, DNA purification, and Hi-C sample preparation. After passing quality control, Hi-C fragments underwent end-biotin removal, sonication, end repair, A-tailing, and adapter ligation to form ligated products. Subsequent PCR steps were amplified to generate library-enriched products. Library amplification products were sampled for Hi-C fragment junction quality control, and the entire library preparation was sequenced using Illumina HiSeq with a PE150 sequencing strategy (NextOmics Biotech). The fastp v.0.12.6 (RRID:SCR_016962) with default parameters was used to filter the raw sequences, resulting in high-quality clean reads. The sequenced Reads1 and Reads2 were separately aligned to the assembled genome sequence using bowtie2 v.2.3.2 (end-to-end alignment mode, parameters: –very-sensitive -L 30) (RRID:SCR_016368) to obtain the alignment information. For the unmapped reads after alignment, we searched for reads containing ligation junction sites, trimmed them, and performed alignment again. Finally, the alignment results were combined, and the proportion of unique mapped paired-end reads was calculated. The LACHESIS software (RRID:SCR_017644) was used to cluster the contig sequences of the draft assembly into chromosome groups using agglomerative hierarchical clustering. The final genome was further assessed with BUSCO [54] for completeness.

Genome of *T. armoricanus* was obtained from the DNA of a fourth instar larva without gut. A total of 23 different insert size libraries were constructed and 67 lanes were sequenced on an Illumina HiSeq2000 platform (RRID:SCR_020130), resulting in 1,344.5 Gb raw data and 877.7 Gb filtered data. The genome was assembled using SOAPdenovo (RRID:SCR_010752) (v2.04) [55] and SSPACE (RRID:SCR_005056) (v2.0) [56] software. We used all 549.3 Gb (180.4×) clean data of short insert size libraries to construct contigs and all 877.7 Gb (266.4×) clean data to construct scaffolds. In total, 283.4 Gb (86.0×) data of large insert size libraries were used again to construct scaffolds by using SSPACE. Then, all clean data of short insert size libraries were used to fill the gaps. TrimDup3 (Rabbit2.6) [57] was used to remove the large redundant sequences. RNA sequencing data from 14 different developmental stages of *T. armoricanus* were assembled by Trinity (RRID:SCR_013048) v2.4.0 [58] and mapped to the assembled genome sequence using BLAT (RRID:SCR_011919) (v. 34) [59], to check the coverage rate. The results showed that 96.8% of the sequences could be mapped to the assembly.

Respective antennae, heads, and labial palps from *A. jianchuanensis* and *T. xiaojinensis* were collected in liquid nitrogen and sequenced with Illumina according to the manufacturer’s instructions. The transcriptomes were assembled by Trinity v2.4.0 [58] with default parameters.

### Phylogenetic analysis and estimation of divergence time

To reconstruct the phylogenetic tree of 16 lepidopteran insect species with 2 outgroups of *Tribolium castaneum* and *Drosophila melanogaster*, we first downloaded the genome annotations or raw data of transcriptomes for other 15 species from NCBI (Supplementary Table S1). The transcripts were assembled by Trinity v2.4.0 [58] with default parameters. Subsequently, the orthologs of these 18 insect species were inferred from their genomic or transcriptomic protein annotations by using OrthoFinder (RRID:SCR_017118) [60] with the default parameters. Single-copy orthologs from each species were selected for phylogenetic reconstruction. The protein sequences of each ortholog were independently aligned with MAFFT (RRID:SCR_011811) v7.407 [61], and the aligned results were trimmed by trimAl (RRID:SCR_017334) [62] to remove low-quality regions with the parameter “-automated1,” with the trimmed sequences concatenated into a single super sequence. RAxML (RRID:SCR_006086) [63] was then used with the VT + F model, which is inferred by ProtTest (RRID:SCR_014628) v3.4.2 [64], to estimate a maximum likelihood tree starting with 1,000 bootstraps followed by likelihood optimization.

We used r8s (RRID:SCR_021161) (V1.7.1) [65] to estimate the divergence time. The phylogenetic tree constructed by RAxML [63] was used as an input tree. A smoothing parameter of 3 was selected, which was estimated by the cross-validation approach (with parameters “cvstart=0, cvinc=1, cvnum=18”). The calibration points were (i) the most recent common ancestor of the clade, including *T. castaneum* and *P. xylostella*, constrained to be 337 million years ago (Mya); (ii) the most recent common ancestor of the clade, including *D. melanogaster* and *C. pomonella*, constrained to be 318 Mya; and (iii) the most recent common ancestor of the clade, including *P. rapae* and *S. litura*, constrained to be 125 Mya [42].

### Annotation of the *Or* gene family

The protein sequences of lepidopteran insect ORs were collected from NCBI. These protein sequences were then used as queries in iterative TBLASTN searches with the parameter “-evalue 1e-5” against the assembly of the 3 ghost moth species to find candidate *Or* genes. A local command line HMMER (RRID:SCR_005305) (version 3.1b2) [66] search was conducted for these candidate ORs against the Pfam-A database (RRID:SCR_004726) to find the 7tm_6 (PF02949) or 7tm_4 (PF13853) HMM profiles for ORs. FGENESH 2.6 [38] prediction of potential genes was performed for contigs of interests. Data from other species were collected according to the reported works (Supplementary Table S1).

### Characterizations of *Ors*

CDS cloning verifications were carried out targeting on annotated *TxiaOrs* using adult antennal cDNA. Gene-specific primers were designed (Supplementary Table S3) and PCRs were done on a Veriti 96-well thermal cycler (Applied Biosystems) using High Fidelity (HiFi) PCR SuperMix (Trans). Products were processed with 1% agarose (BBI) on a PowerPac electrophoresis system (Bio-Rad) and visualized with a GelDoc-It TS3315 imaging system (UVP). Multiple bands such as for *TxiaOr18* were separately collected and purified with a gel extraction kit (GenStar) before Sanger sequencing (Sangon Biotech). Later analysis was based on the longest sequenced *TxiaOrs* for each locus. *Or* expressions were shown as autoscaled heatmaps indicating the FPKMs (fragments per kilobase of transcript per million mapped reads), which were calculated by RSEM [67] from head, antenna, and labial palp transcriptomes of adult ghost moths.

Phylogenetic analysis of 387 ORs was carried out with the abovementioned protocol using MAFFT [61], trimAl [62], and IQ-TREE (RRID:SCR_017254) [68] using the “Auto” option for model, with 1,000 ultrafast [69] bootstraps, as well as the Shimodaira–Hasegawa–like approximate likelihood ratio test [70]. Verifications were done to the tree topology with MEGA X [71] and MrBayes (RRID:SCR_012067) 3.2.6 [72] to establish the NJ tree based on the Dayhoff model and BY tree based on the Blosum62 model, respectively. Homologs of the TxiaOR19 array were predicted using CLANS [37] using the blastx results against the NCBI nr database. For chromosome linearization tests, local tblastn was applied to map the selected ORs toward chromosomes of each species (Supplementary Table S1), and results were visualized as circos plots by using TBtools (RRID:SCR_023018) v1.113 [73]. Evolution of mapped ORs was inferred using MrBayes 3.2.6 [72] under the JTT+F+G4 model (2 parallel runs, 200,000 generations), in which the initial 25% of sampled data were discarded as burn-in. The final average standard deviation of split frequencies was 0.069772. Protein motifs were predicted using MEME Suite (RRID:SCR_001783) v5.5.2 [74].

### Annotation of repeats and transposable element families

For transposable element analysis, we first performed the *de novo* predictions for each species by RepeatModeler (RRID:SCR_015027) version open-1.0.11 to generate a specific library. Then we annotated the genome assembly by RepeatMasker (RRID:SCR_012954) version open-4.0.7 with the “ncbi” search algorithm. Annotated transposable element sequences were manually verified and classified with Dfam (RRID:SCR_021168) [75]. The calcDivergenceFromAlign.pl and createRepeatLandscape.pl scripts in the RepeatMasker package were used to calculate the Kimura divergence values and plot the repeat landscape, respectively. Estimations for transposable element burst times were based on the recently reported substitution rate of 6.19 × 10^−10^ per site per generation in arthropods [76].

### Chemical analysis

Hexane extraction method was adopted from our previous works on moth pheromone identifications [77]. Abdomen tips of calling adult *T. xiaojinensis* were cut with dissection scissors and immediately put in 20 μL hexane (HPLC purity; Kermel Chemical Reagent Co.), which was kept at 4°C for 1 day prior to the test. Head space SPME method was adopted from our previous works on body surface volatile emissions of insects [78]. Newly emerged male or female adults were kept in a mesh cage in separated rearing chambers for sampling. A 50/30-μm DVB/CAR/PDMS stableflex fiber (Supelco) was penetrated into the cage for sampling at 10°C for 24 hours. The volatile blends sampled were either injected for 1 μL or subjected to an Agilent 7890B GC–5977 MSD coupled system equipped with an HP-5MS column (0.25 μm × 30 m × 0.250 mm) (Agilent). A 60-minute oven temperature program was used as follows: 40°C for 2 minutes, 40°C to 150°C at 5°C/min, 150°C for 2 minutes, 150°C to 200°C at 10°C/min, 200 °C for 5 minutes, 200°C to 230°C at 5°C/min, and 230°C for 18 minutes. Raw data were analyzed with MSD ChemStation (G1701FA F. 01. 03. 2357) by searching against an NIST 17 MS library (Agilent). A total of 40 individuals were tested for SPME from 2 stratified groups. Each hexane extraction sample included 20 individuals and at least 3 replicates were done toward each sex.

### Docking simulation

TxiaOR19 and the other 4 sex-biased OR sequences of *T. xiaojinensis* were predicted by AlphaFold2 [79] for their tertiary structures. The 3D structures of 18 ligands were downloaded from PubChem [80]. The Molecular Operating Environment software (MOE; Chemical Computing Group ULC) was used to dock the ligands with ORs. Briefly, ORs were prepared using MOE QuickPrep and ligands were energy minimized with the MOE Energy Minimize prior to the simulation. Triangle Matcher algorithm was selected for placement and 30 top-scoring placement poses were selected by the London dG empirical scoring function, while the rigid receptor was selected for refinement and top-scoring poses were selected by the GBVI/WSA dG empirical scoring function. The binding free energy of respective OR–ligand was estimated by using S Score function and later used for establishment of a color-coded map.

### Courtship arena

The assays were carried out using 1-day emerged naive moths at peak mating hours 6 to 8 p.m. during sunset. One randomly chosen pair of *T. xiaojinensis* adults was placed in a paper funnel and recorded for 1 hour. A total of 20 pairs were tested and recorded for calling and tracing behaviors. Recorded footages were processed through the idTracker [81] pipeline to obtain the velocities of moths shown as per pixel distances per minute. Fluttering behaviors were observed by manually checking each video file.

### Statistics and data processing

Comparison of means was done by using either unpaired *t*-test or the general linear model (GLM) followed by multiple comparisons according to treatment sizes (SPSS 22.0.0.0; IBM Corp.). Simple linear regression and data plotting were done using Prism 5.01 (GraphPad Software). Multivariate tests were carried out with MetaboAnalyst (RRID:SCR_015539) 5.0 [82] server, which integrates R statistics (RRID:SCR_001905). All error bars indicate standard errors of the means unless otherwise indicated in the figure legends.

## Supplementary Material

giae044_GIGA-D-23-00252_Original_Submission

giae044_GIGA-D-23-00252_Revision_1

giae044_GIGA-D-23-00252_Revision_2

giae044_Response_to_Reviewer_Comments_Original_Submission

giae044_Response_to_Reviewer_Comments_Revision_1

giae044_Reviewer_1_Report_Original_Submission William B. Walker, Ph.D. -- 10/6/2023 Reviewed

giae044_Reviewer_1_Report_Revision_1 William B. Walker, Ph.D. -- 5/2/2024 Reviewed

giae044_Reviewer_2_Report_Original_Submission Yi-Ming Weng, Ph.D -- 1/24/2024 Reviewed

giae044_Reviewer_2_Report_Revision_1 Yi-Ming Weng, Ph.D -- 5/4/2024 Reviewed

giae044_Supplemental_Files

## Data Availability

The whole-genome sequence data of *T. xiaojinensis* reported in this article have been deposited in NCBI (Bioproject: PRJNA1006505). All additional supporting data are available in the *GigaScience* repository, GigaDB [83].

## Additional Files

**Supplementary Fig. S1**. Strength of chromosomal interactions within intervals. Based on the strength signals of interactions between contig sequences with their final determined order and orientation, we generated a Hi-C interaction heatmap for the chromosomes. The heatmap coordinates represent chromosomes, and the color of each point corresponds to the log value of the interaction strength of the respective genome bin pair, ranging from red to black, indicating increasing interaction strength. Notably, the 32 chromosomal groups are clearly distinguishable. Within each group, interactions along the diagonal are stronger than those off the diagonal, indicating higher interaction strength between adjacent sequences (diagonal positions) in the Hi-C assembled chromosomes, consistent with the principles of Hi-C–assisted genome assembly. There is minimal noise (strong interaction strength) outside the diagonal, affirming the effectiveness of the genome assembly.

**Supplementary Fig. S2**. Antennal and forewing length comparison among Lepidoptera. Where square dots indicate each measurement of individual sample, gray bars indicate the ranges of O.I. from the species. O.I. indicates olfactory index, which equals antenna/forewing. Data of hepialids were collected in the current work (blue), and other families were referred to published works [26].

**Supplementary Fig. S3**. Representative SEM photos of selected moth species. Arrowheads indicate representative sensilla trichoidae morphology in each panel. (A) *A. jianchuanensis* male. (B) *A. jianchuanensis* female. (C) *T. armoricanus* male. (D) *T. armoricanus* female. (E) *T. xiaojinensis* male. (F) *T. xiaojinensis* female. (G) *H. cunea* male. (H) *H. cunea* female. (I) *P. rapae* male showing only distributions of sensilla trichoidae in the “sacculus.”

**Supplementary Fig. S4**. Evolution of ordinary glomerular morphology in Lepidoptera. (A) Schematic shows registration of glomerular organizations among species. Ordinary glomeruli were assigned according to their spatial locations as A: anterior, D: dorsal, V: ventral, C: central, L: lateral, and P: posterior. Arrows indicate numerical order of each spatial cluster. (B) Comparison of annotated glomerular volumes between male and female Lepidoptera. The analysis includes 101 assigned glomeruli. Fold change threshold was 2.0. (C) Debiased sparse partial correlation (DSPC) network concerning volume relationships of selected 43 glomeruli across species. Glomerular volume was assigned as 0 when they were not observed in a species. Square size indicates correlation degree of each glomerulus. Line weight indicates the *P* value between each pair. (D) Correlation map of all glomeruli across tested 18 species. Representative MGC/LFG-free ALs of the species are shown on top, together with family ranges indicated. (E) Self-organizing map (SOM) cluster analysis of tested species according to ordinary glomeruli.

**Supplementary Fig. S5**. Comparison of the cumulus considering volume weights in males (left) and LFG1 in females (right). Lowercase letters indicate significant differences among species (GLM and Duncan multiple comparison, cumulus: *F*_15, 32_ = 6.05, *P* < 0.0001; LFG1: *F*_15, 32_ = 14.91, *P* < 0.0001).

**Supplementary Fig. S6**. PCA plot of MGC measurements with 16 tested Lepidoptera species at 73.8% fraction of explained variance. Representative MGC glomeruli from *A. jianchuanensis, T. armoricanus*, and *T. xiaojinensis* are shown. Cu indicates the cumulus.

**Supplementary Fig. S7**. PCR verifications for annotated *TxiaOrs*. (A) Cloned PCR products of respective *TxiaOrs* from antennal cDNA. M: DNA 2000D ladder. Captions indicate corresponding *TxiaOrs* according to the primers in Supplementary Supplementary Table S2. *TxiaOr18* was not cloned in this test, but we harvested its clones in another run using optimized primers [*TxiaOr18new*]. (B) PCR verifications and alternative splicing of *TxiaOr18*. The longest correspondence TxiaOR18c was used in all successive analysis.

**Supplementary Fig. S8**. Tandem *TxiaOr19* intact *Or* duplications exclude pseudogenes. (**A**) Gene locations of the *TxiaOr19* tandem on chr14 of *T. xiaojinensis*. (**B**) Exon–intron arrangements of the *TxiaOr19* tandem.

**Supplementary Fig. S9**. Verifications of ML tree structures with NJ and BY methods. The same batch of 387 ORs was used to establish the phylogeny as used in Fig. 2A. Red highlights TxiaOR19 array ORs, and blue indicates canonical PRs. (A) NJ tree was established using MEGA X with the Dayhoff matrix-based method. (B) Bayes tree was established by MrBayes using Blosum62 model. A total 200,000 generations were run and a relative burn-in of 25% was used for diagnostics. The final average standard deviation of split frequencies was 0.12.

**Supplementary Fig. S10**. Expression profiles of *TxiaOrs*. Volcano plot (up) shows significantly different expressed *Ors* in *T. xiaojinensis* between male and female adults. Color-coded map (down) showing expression levels of identified *Ors* in *T. xiaojinensis* adult heads, antennae, or labial palps.

**Supplementary Fig. S11**. Distributions of predicted TxiaOR homologs across species by nr blasting.

**Supplementary Fig. S12**. Clustering of blasted ORs by TxiaOR19 lineage. CLANS results indicate clustering of all identified ORs by blasting against nr protein library with TxiaOR19 and reference TxiaOR7, respectively. CLANS was conducted using default parameters.

**Supplementary Fig. S13**. Bayes tree showing evolutionary relationships of ORs mapped from linearization analysis and nr blast with TxiaOR19 tandem. Bayesian inference phylogenies were inferred using MrBayes 3.2.6 under the JTT+F+G4 model (2 parallel runs, 200,000 generations), in which the initial 25% of sampled data were discarded as burn-in. The final average standard deviation of split frequencies was 0.069772. Color indications were used as per Fig. 2B. Uncolored ORs were selected according to blastp results of the TxiaOR19 array against the nr database. Species included *T. xiaojinensis, C. pomonella, L. marmoratus, M. aruncella, I. masculella, N. swammerdamellus, C. flavipennella, M. sexta, B. mori, A. ipsilon, A. rosae, H. illucens, C. quinquefasciatus, A. gossypii, S. americana*, and *S. cancellata*.

**Supplementary Fig. S14**. Comparison of overall TE distributions among caddisfly and moths.

**Supplementary Fig. S15**. Aligned correlation matrix showing arrangement patterns of TEs in TxiaOR18c/19 mapped OR loci. Screened regions for TEs included coding area and respective 5,000 bp per up- and downstream. Ribbons indicate neighboring of ORs on the same chromosome, and directions were from wider to narrower. Blue-colored ORs were from *T. xiaojinensis*, and those in red were ORs mapped simultaneously by TxiaOR18c/19 and classic PRs.

**Supplementary Fig. S16**. Adult emissions and predicted non-PR functioning of TxiaOR19. (A) GC-MS traces of abdomen tip extracts (gray) or head space volatiles (blue) from *T. xiaojinensis* adults (*n* = 20). Blue traces indicate chemicals collected by SPME from the same female after emerging 0–24 hours and 24–48 hours, respectively. (B) Color-coded map indicates results from docking simulations of TxiaOR19 array and the other 4 sex-biased TxiaORs against tested ligands.

**Supplementary Table S1**. Data resources for the phylogenomics and comparative genomic analysis.

**Supplementary Table S2**. Comparison of OR repertoires among the tested Lepidoptera.

**Supplementary Table S3**. List of PCR primers used in the study.

## Abbreviations

BUSCO: Benchmarking Universal Single-Copy Orthologs; LFG: large female glomeruli; MGC: macroglomerular complex; Mya: million years ago; OR: odorant receptor; ORco: odorant receptor coreceptor; PBS: phosphate-buffered saline; PR: pheromone receptor; SPME: solid-phase microextraction; TE: transposable element.
