# Supplementary material for: A ghost moth olfactory prototype of the lepidopteran sex communication
Source: Gigascience. 2024 Jul 19;13:giae044. doi: 10.1093/gigascience/giae044 (PMC11258902; doi:10.1093/gigascience/giae044)

GigaScience

A ghost moth olfactory prototype of the lepidopteran sex communication

--Manuscript Draft--

**Manuscript Number:**	GIGA-D-23-00252
**Full Title:**	A ghost moth olfactory prototype of the lepidopteran sex communication
**Article Type:**	Research
**Funding Information:**	Major Science and Technology Project of Qinghai Province (2021-SF-A4-1) — Prof Ri-Chou Han<br>GDAS Special Project of Science and Technology Development (2022GDASZH-2022010106) — Dr Rui Tang<br>Guangzhou Science and Technology Project (202201010039) — Dr Rui Tang<br>Guangdong Basic and Applied Basic Research Foundation (2020A1515011366) — Prof Ri-Chou Han
**Abstract:**	Sex role differentiation is a widespread phenomenon. Sex pheromones are often associated with sex roles and convey sex-specific information. In Lepidoptera, females release sex pheromones to attract males, which evolve sophisticated olfactory structures to relay pheromone signals. However, in some primitive moths, sex role differentiation becomes diverged. Here, we introduce the inaugural chromosome-level genome assembly from ancestral Himalaya ghost moths, revealing a unique olfactory evolution pattern and gender parity among Lepidoptera. These olfactory structures of the ghost moths are characterized by a denser population of trichoid sensilla, both larger male and female antennal entry parts of brains, compared to modern lepidopterans. Furthermore, a unique tandem of 34 odorant receptor 19 homologs in Thitarodes xiaojinensis (TxiaOr19) has been identified, which presents overlapped motifs with pheromone receptors (PRs). Interestingly, the expanded TxiaOr19 does not function as canonical PRs, with non-sexual dimorphic olfactory neuropils discovered, which contributes to the observed equal sex roles in Thitarodes adults. Additionally, transposable element activity bursts have provided traceable loci landscapes where parallel diversifications occurred between TxiaOr19 and PRs, indicating that the Or19 homolog expansions were diversified to PRs during evolution and thus established the classic sex roles in higher moths. This study elucidates an olfactory prototype of intermediate sex communication from Himalaya ghost moths.
**Corresponding Author:**	Ri-Chou Han, Ph.D.<br>Guangdong Academy of Sciences<br>Guangzhou, Guangdong CHINA
**Corresponding Author Secondary Information:**	
**Corresponding Author's Institution:**	Guangdong Academy of Sciences
**Corresponding Author's Secondary Institution:**	
**First Author:**	Rui Tang, Ph.D.
**First Author Secondary Information:**	
**Order of Authors:**	Rui Tang, Ph.D.
	Cong Huang
	Jun Yang
	Zhong-Chen Rao
	Li Cao
	Peng-Hua Bai
	Xin-Cheng Zhao

	Jun-Feng Dong
	Xi-Zhong Yan
	Fang-Hao Wan
	Nan-Ji Jiang
	Ri-Chou Han
**Order of Authors Secondary Information:**	
**Additional Information:**	
**Question**	**Response**
Are you submitting this manuscript to a special series or article collection?	No
**Experimental design and statistics**<br><br>Full details of the experimental design and statistical methods used should be given in the Methods section, as detailed in our Minimum Standards Reporting Checklist. Information essential to interpreting the data presented should be made available in the figure legends.<br><br>Have you included all the information requested in your manuscript?	Yes
**Resources**<br><br>A description of all resources used, including antibodies, cell lines, animals and software tools, with enough information to allow them to be uniquely identified, should be included in the Methods section. Authors are strongly encouraged to cite Research Resource Identifiers (RRIDs) for antibodies, model organisms and tools, where possible.<br><br>Have you included the information requested as detailed in our Minimum Standards Reporting Checklist?	Yes
**Availability of data and materials**<br><br>All datasets and code on which the conclusions of the paper rely must be	Yes

either included in your submission or deposited in publicly available repositories (where available and ethically appropriate), referencing such data using a unique identifier in the references and in the "Availability of Data and Materials" section of your manuscript.<br><br>Have you have met the above requirement as detailed in our Minimum Standards Reporting Checklist?	

Main text and figure legends Click here to access/download;Manuscript;manuscript.docx

Research Article

A ghost moth olfactory prototype of the lepidopteran sex communication

Rui Tang[1#a], Cong Huang[23#], Jun Yang[4], Zhong-Chen Rao[1], Li Cao[1], Peng-Hua Bai[5], Xin-Cheng Zhao[6], Jun-Feng Dong[7], Xi-Zhong Yan[4], Fang-Hao Wan[23], Nan-Ji Jiang[8]*, Ri-Chou Han[1]*

1 Guangdong Key Laboratory of Animal Conservation and Resource Utilization, Guangdong Public Laboratory of Wild Animal Conservation and Utilization, Institute of Zoology, Guangdong Academy of Sciences, Guangzhou, China 510260

2 State Key Laboratory for Biology of Plant Diseases and Insect Pests, Institute of Plant Protection, Chinese Academy of Agricultural Sciences, Beijing, China 100193

3 Shenzhen Branch, Guangdong Laboratory for Lingnan Modern Agriculture, Genome Analysis Laboratory of the Ministry of Agriculture, Agricultural Genomics Institute at Shenzhen, Chinese Academy of Agricultural Sciences, Shenzhen, China 518120

4 College of Plant Protection, Shanxi Agricultural University, Taigu, Shanxi, China 030801

5 Institute of Plant Protection, Tianjin Academy of Agricultural Sciences, Tianjin, China 300384

6 Henan International Laboratory for Green Pest Control, College of Plant Protection, Henan Agricultural University, Zhengzhou, China 450046

7 Forestry College, Henan University of Science and Technology, Luoyang, China 471000

8 Department of Evolutionary Neuroethology, Max Planck Institute for Chemical Ecology, Hans-Knöll-Straße 8, Jena, Germany D-07745

*Correspondence: Nan-Ji Jiang, njiang@ice.mpg.de Hans-Knöll-Straße 8, Jena, Germany. Tel. +49 (0)3641 57-1456; Ri-Chou Han, hanrc@giz.gd.cn, 105 Xingang West Road, Haizhu District, Guangzhou. Tel. +86 020-84191089

# Equal contribution was claimed.

ORCID.ORG: a 0000-0002-9313-0802

Abstract

Sex role differentiation is a widespread phenomenon. Sex pheromones are often associated with sex roles and convey sex-specific information. In Lepidoptera, females release sex pheromones to attract males, which evolve sophisticated olfactory structures to relay pheromone signals. However, in some primitive moths, sex role differentiation becomes diverged. Here, we introduce the inaugural chromosome-level genome assembly from ancestral Himalaya ghost moths, revealing a unique olfactory evolution pattern and gender parity among Lepidoptera. These olfactory structures of the ghost moths are characterized by a denser population of trichoid sensilla, both larger male and female antennal entry parts of brains, compared to modern lepidopterans. Furthermore, a unique tandem of 34 odorant receptor 19 homologs in *Thitarodes xiaojinensis* (*TxiaOr19*) has been identified, which presents overlapped motifs with pheromone receptors (PRs). Interestingly, the expanded *TxiaOr19* does not function as canonical PRs, with non-sexual dimorphic olfactory neuropils discovered, which contributes to the observed equal sex roles in *Thitarodes* adults. Additionally, transposable element activity bursts have provided traceable loci landscapes where parallel diversifications occurred between *TxiaOr19* and *PRs*, indicating that the *Or19* homolog expansions were diversified to *PRs* during evolution and thus established the classic sex roles in higher moths. This study elucidates an olfactory prototype of intermediate sex communication from Himalaya ghost moths.

Keywords: Genome; Olfactory evolution; Neuroecology; Lepidoptera; Ghost moth; Sex role

Introduction

Sexual dimorphism is ubiquitous across the animal kingdom [1]. For most animals, mating by partner allocation is an indispensable process to ensure population continuity [2]. Sex roles often form under the pressure of sexual selection [3]. In general, the female exhibiting greater parental investment becomes a limiting resource for the less caring male so that the latter competes for accessing to the former [4]. In most insects, such as the vinegar fly *Drosophila melanogaster*, males usually release the specific pheromone cis-vaccenyl acetate to gain an advantage in recruiting females [5]. However, sex roles appear to be reversed in moths [6]. Female moths invest in synthesizing and releasing sex pheromones to attract male moths, and males have evolved distinct structures for sensing pheromones [7,8]. Therefore, the study of pheromone and pheromone perception can expand our understanding of the evolution of sexual roles in animals.

One well-known animal lineage that relies on pheromone communication is Lepidoptera, comprising nearly 160,000 extant species and forming a key branch of insects [9]. Lepidoptera pheromones were well-studied in the last decade, and most can be classified into type 0, I, II, and III, according to their hydrocarbon chains, double-bond allocations, and terminal functional groups [10]. Among them, type I pheromones, consisting of straight-chain acetates, alcohols, or aldehydes with 10 to 18 carbon atoms, make up 75% of all known sex pheromones and are employed by most moth families [11]. Pheromones are detected by pheromone receptors (PRs)/odorant receptor co-receptors (ORco) on the dendrites of olfactory sensory neurons. Based on the pheromone types, the corresponding PR family can be classified into type 0, I, and II clades [12]. However, the recent discovery of *Lampronia capitella* OR6/Orco and *Spodoptera littoralis* OR5/ORco has revealed a novel 'PR clade' that is distant from the type I PR clade [13,14]. This implies that the mechanisms underlying the evolutionary process of ORs for detecting pheromones in Lepidoptera need to be

explored.

The neural architectures of pheromone perception appear to be conserved in moths [15]. A typical perception of type I pheromone is achieved through a label-lined olfactory coding pattern in higher moths, such as Noctuidae. Pheromones are tuned by olfactory sensory neurons housed in sensilla trichoidae on the antennae. After the PR/ORco complex has been activated by the corresponding pheromone, the potential signals are projected to the primary olfactory center, the antennal lobe [16]. In Lepidoptera, the antennal lobe shows obvious sexual dimorphism. The male-specific macroglomerular complex (MGC) is located at the entry of the antenna and exclusively processes pheromone signals [17]. The counterparts of the MGC in females are usually called the large female glomeruli (LFG) that process oviposition and host-choosing signals, but LFG glomeruli are not generally enlarged in size as the MGC [18,19].

The ghost moths (Hepialoidae: Hepialidae) from Exoporia are primitive Lepidoptera species and form an especially interesting lineage for studying the evolution of sex roles and pheromone communication [20]. Hepialids represent an early branch from the line leading to the heteroneuran Ditrysia, and the latter includes almost all the lepidopteran species which use typical PR-based olfaction for pheromones. Notably, the sex roles of Hepialidae species show diversity; for example, *Hepialus hecta* and *H. humuli* exhibit courtship behavior that is very different from the usual moth pattern, as males hover in groups to attract females [20]. Moreover, ghost moths have undergone asymmetrical divergence of duplicated genes to deliver functional alterations in subsequent species, providing insights into the evolutionary process of Lepidoptera [21]. *Thitarodes*, *Ahamus*, and *Hepialus* ghost moths, as the hosts of *Ophiocordyceps sinensis* medicinal fungus, are epidemic only in the Qinghai-Tibet Plateau [22]. The isolated ecological habitat and prolonged life cycle of these so-called Himalaya ghost moths provide ideal opportunities for the retention of pheromone

receptive characteristics from shared ancestors of Lepidoptera [23-25]. These findings make ghost moths promising target insects for studying the evolution of pheromone communication and sex roles in Lepidoptera.

In this study, we presented the unique evolutionary position of olfaction in ghost moths, characterized by comparative neurology and phylogenomics. Our results demonstrated that the antennal lobes of both male and female of three Himalaya ghost moth species (*Ahamus jianchuanensis*, *Thitarodes armoricanus*, *T. xiaojinensis*) epidemic in the Qinghai-Tibet Plateau have an enlarged antennal entry part and lack obvious sexual dimorphism, when compared to later lepidopterans. Comparative genomics further revealed that the ghost moth *T. xiaojinensis* expanded a specific *Or19* tandem array instead of the classic type I PR clade. Behavioral tests indicated that the ghost moth *T. xiaojinensis* exhibits similar sex roles between males and females in courtship, possibly due to their pheromonal neural architectures without sexual dimorphism and the specific *Or19* tandem array. In summary, this study uncovers a mechanism for the occurrence of functional ORs such as PRs through asymmetric divergence in Lepidoptera.

Results

Chromosome-level genome assembly of Himalaya ghost moth

A *T. xiaojinensis* larva was sequenced using Nanopore long-read technology, resulting in 319.9 Gb of clean reads. The draft genome of 3.1 Gb, comprising 1,645 contigs with a contig N50 of 5.4 Mb, was assembled using NextDenovo, corrected with minimap2 and NextPolish, and refined by removing contaminants. Utilizing Hi-C interaction data, the primary assembly was divided into 31,434 contigs, and 31,391 contigs (99.86% in length) were anchored to 32 chromosomes (Figure S1). BUSCO analysis revealed 91.8% complete genes in the final

chromosome-level genome assembly, which was subsequently employed in downstream analysis.

Evolutionary position of ancient Himalaya ghost moths based on phylogenomics analysis

We carried out a phylogenomics analysis based on genomes and transcriptomes of three ghost moth species, together with 13 Lepidoptera and two outgroups (Table S1). The separation of exoporian and ditrysian Lepidoptera occurred by the end of the Triassic Period, at around 205 million years ago. While the speciation of the *Thitarodes* moths soon followed *A. jianchuanensis*, at around 26 million years ago by the end of the Paleogene Period. Although *Ahamus* and *Thitarodes* represented an ancient moth lineage, the species within were diverged in parallel with higher moths (Figure 1A). We next asked what olfactory traits were maintained during the evolution of Himalaya ghost moths.

Nonsexual dimorphic shortened antennae and enlarged glomeruli of Himalaya ghost moths

The Himalaya ghost moths had no observable proboscis but possessed the antennae and labial palps intact (Figure 1B). Besides, we found that these moths presented the shortest antennae among 32 lepidopteran families [26] (Figure 1C) and their antennae were dominated by sensilla trichoidae (Figure 1D, Figure S2). In particular, per segment counts of long sensilla trichoidae in these ghost moths of both male and female were significantly higher than that from a *Hyphantria cunea* reference and other Lepidoptera species (Figure 1D, Table S2). This indicates that both male and female Himalaya ghost moths may keep pheromone reception with their shortened antennae.

The antennal lobe morphological atlas showed that three ghost moth species overall presented significantly less glomeruli (24 to 35), compared with other Lepidopteran moths (in general 50 to 80 glomeruli) (Figure 1E). Amongst 96 tested brains, the ordinary glomeruli arrangements in

Himalaya ghost moths were distinguishable with those in the compared species. The families of Hepialidae, Pieridae, and Plutellidae had less intra-species variations in glomerular arrangements compared to the other four higher moth families (Figure S3). The MGC consisted of 2 to 3 glomeruli in Himalaya ghost moths, and identical areas were confirmed in all tested species. Female LFGs was distinguishable in earlier species including the Himalaya ghost moths and the diamondback moth *Plutella xylostella*, comparing with the later species (Figure 1E).

Volume proportions of the MGC and LFG glomeruli across tested species were compared. It showed that Himalaya ghost moths had both the largest MGCs and LFGs (Figure 1F and G). The cumulus which represents a major area involving pheromone reception, occupied 23.8 $\pm$ 5.5% of the antennal lobes in *A. jianchuanensis*, 15.9 $\pm$ 2.0% in *T. armoricanus* and 19.5 $\pm$ 3.0% in *T. xiaojinensis*, respectively (Figure 1F, Data S1). The other species had relatively smaller MGC glomeruli in volumes, e.g., on average 9.8 + 0.7% of the cumulus occupation for Noctuidae. As for females, LFG1 in three ghost moth species were significantly larger than those within higher moths (Figure 1G, Data S1). We checked the MGCs of 16 species in terms of volumes and shapes by utilizing a principal component analysis test, and 73.8% fraction of explained variances was covered. Twelve of 16 lepidopteran species had a similar trend in MGC organizations, but the Himalaya ghost moths and *H. cunea* exhibited separated patterns, and especially *T. xiaojinensis* was totally isolated from higher Lepidoptera (Figure 1H). We wonder if these structural specificities may reflect the genomic backgrounds and receptor repertoires in the Himalaya ghost moths.

A unique large OR array on the ghost moth T. xiaojinensis *chromosome*

A total of 23 *TxiaOrs* were confirmed to be expressed from the genome and transcriptomes

of *T. xiaojinensis*, out of 57 annotations (Figure S4, Table S3 and S4). This number was higher than those of *A. jianchuanensis* (10) and *T. armoricanus* (16) (Table S4). Notably, a large array comprising 34 tandem duplications was mapped by higher moth PRs on LG14 of the chromosome-level assembly of *T. xiaojinensis*. This array, homologous to *TxiaOr1*9, contained 16 homologs (*TxiatdOrs*) and 18 pseudogenes (*TxiatdpOrs*), which maintained the largest tandem duplications reported in lepidopterans (Figure S5, Data S2). The *TxiaOr19* array was located on the same chromosome with an upstream *TxiaOr18c*, which mapped to Noctuidae homologs [27]. The Maximum-Likelihood phylogeny analysis using 272 ORs showed that the *TxiaOr19* array formed an earlier group where canonical type I PRs arose (Figure 2A, Data S3). This tree topology between the two clades was consistent when cross-checked with both Neighbor-Joining and Bayesian methods (Figure S6). A female-biased expression pattern was observed in *TxiatdOr15* and *TxiatdOr25* of the 16 *TxiaOr19* homologs (Figure 2B). The *TxiaOr19* array suggested an earlier emergence than male-biased *TxiaOr7* as it could blast to ORs in locust, aphid, soldier fly, mosquito, and flea, with homologs predicted by CLANS [28] in mosquito and flea (Figure 2C, Figure S7).

We investigated the evolution of the *TxiaOr19* array by mapping it to chromosomes of caddisflies, primitive and higher moths, and cross-checking with canonical PR mapped regions (Figure 2D). The results indicated that tandem ORs were identified in linearized regions of almost all species, except for *C. flavipennella* and *B. mori*. Some of these linearized regions did not contain ORs that could be annotated using FGENESH [29], suggesting possible evolutionary patterns similar to the duplicated *zen* family of orange swift moth *H. sylvina*, which diverged to new functional gene families *Shx* [30]. On the other hand, PRs could be traced back to a single LarmPR1 in the caddisfly, and expansions of PR tandems were consistently observed within species after the Himalaya ghost moths, except for *C. flavipennella*. Notably, regions containing *TxiaOr19* and PR

had mixed tandem patterns in the early moths succeeding *T. xiaojinensis* but tended to be separated in later species (Figure 2D). Bayesian phylogenetic analysis showed that the homologs of *TxiaOr19* underwent significant diversification from their ancestors (Figure 2E). Specifically, *TxiaOr19* array arose from the *LarmOr19-Or13a* tandem (Figure 2E). The majority of PR-mapped ORs formed a single cluster that possibly diverged from LarmPR1, with a few remaining in the TxiaOR19 mapped phyla (Figure 2E).

LarmPR1 exhibited all three motifs of PR consensus regions [12], indicating the potential emergence of canonical PRs prior to the evolution of lepidopteran insects (Figure 2F). However, most PR-mapped ORs from non-Ditrysia primitive moths did not meet the requirements of canonical PR motifs (Figure 2F). The majority of TxiaOR19-mapped ORs had two motifs that overlapped with PRs, with some having three motifs but with shifted positions (Figure 2G). Notably, motifs 4-6 from TxiaOR19 homologs showed overlaps with motifs 2-3 from PR homologs, suggesting the existence of a common ancestor for TxiaOR19 and canonical PRs at an earlier evolutionary stage (Figure 2H). In all, the genomic backgrounds and receptor repertoires are specific in *T. xiaojinensis*, which may confer to the structural specificities described above.

Transposable elements (TE) *involved in the evolution of ORs*

Tandem gene duplications and chromosome linearization patterns have been reported to be associated with TE activities [31]. To explore how a large and specific OR array was formed in the ghost moth *T. xiaojinensis*, we characterized the landscape of TEs in the genomes of above species. It showed that 2-3 TE burst events occurred in the Himalaya ghost moths. These bursts likely took place around the same time as the successive divergences of Exoporia and *Hepialus* (Figure 3A, Figure 1A). Caddisfly, primitive and modern Lepidoptera experienced more recent bursts of TE

activity compared to Himalaya ghost moths (Figure 3A and B). Specifically, various TE arrangements were observed in previously identified OR loci. The TE arrangements of the *TxiaOr19* tandem were correlated with that of the *LarmOr13a* (Figure 3C). Furthermore, the TE landscapes in the *TxiaOr18/19* homologs were found to be similar mostly with *Ors* from non-ditrysian species and later diversified in higher moths (Figure 3C).

In conclusion, our findings suggest that both the TxiaOR19 tandem and PR clusters had already emerged in caddisflies. The OR19 lineage underwent expansion within the ancestral moth lineage, leading to the formation of a large duplicated tandem in *T. xiaojinensis*. Furthermore, linkages between OR19 and PRs were observed in species predating Ditrysia. In later higher moths, PRs became predominant, while the OR19 cluster contracted through asymmetric diversifications of their homologs, as supported by the similar TE arrangements (Figure 3D).

Equal sex roles of ghost moth T. xiaojinensis *adults*

The replacement of the TxiaOR19 duplications with canonical PRs suggests possible functional drift of TxiaOR19. To confirm this assumption, we first analyzed the emissions of adult *T. xiaojinensis* using both solvent extraction and solid-phase microextraction (SPME) methods. We found that male and female adults were not distinguishable by tracing the volatile blends in abdomen tip extractions. However, SPME samples collected within the first 24 hours after female emergence exhibited a significant peak corresponding to oleamide (Figure S8A). We performed successive docking simulations using TxiaOR19 and four sex-biased TxiaORs against the identified major components. The results showed that TxiaOR19 had less binding affinity towards the panel of the ghost moth emissions, and its responding spectrum was relatively broad (Figure S8B), indicating that ghost moth *T. xiaojinensis* may show unconventional courtship behaviors

comparing to higher moths.

To confirm this speculation, we tested adult pairs of *T. xiaojinensis* in a courtship arena (Figure 4A). Unlike the female calling behaviors of higher moths with wing beats and extruded pheromone gland, calling behavior of this ghost moth involved hovering with wing beats. Females fluttered with substantial wing beats, while males fluttered by small range vibrating-like wing beats (Figure 4B). Interestingly, male and female adults exhibited similar amounts of calling behaviors and tracing velocities (Figure 4B and C). Our results indicated that the sex roles of *T. xiaojinensis* adults were different from those of higher lepidopterans during mating allocation, which fits the predicted non-PR functioning of TxiaOR19 to a non-canonical female emission and the olfactory architectural observations.

Discussion

Himalaya ghost moths offer a basal model for the study of olfaction evolution in insects due to their unique olfactory system, and possession of the largest genomes in Lepidoptera. The limited and fragmented natural distribution of the species poses challenges for conducting molecular functional studies on their populations. Our study has successfully generated the first chromosome-level assembly for this lineage, providing valuable insights into the genetic characteristics of these ghost moths. We have also discovered that both males and females of these moths possess a compact olfactory system with distinct structures involved in sexual recognition. We have discovered that the OR evolutionary pathway in ghost moth *T. xiaojinensis* parallels with that of modern moths, and have identified molecular traces that reveal the origins of the modern pheromone sensory system. Interestingly, the non-sexual dimorphic MGC/LFG and mostly non-

biased expressions of *TxiaOr19* homologs support our observation of equal sex roles in Himalaya ghost moths.

Why the lineage of Himalaya ghost moths keeps primitive may be due to their isolated habitats, uneven long life cycle for larvae (3-6 years in nature) and a brief adult stage (several days) [24,25], which greatly reduces the evolution speed of this lineage. They have developed a unique evolutionary strategy of focusing solely on reproduction in adulthood while forgiving foraging [32]. The redundancy of their giant genomes is an example of the basal genomic features possessed by the ghost moths [33]. Asymmetrically diverged duplications and frequent TE activity bursts have played a critical role in both PR formation and the emergence of other functional genes in Lepidoptera [30]. While TxiaOR19 duplications have been linked to the expansion of the moth PR clade in higher moths, OR19 duplications in these ghost moths do not appear to have enhanced functions, unlike tandem ORs in higher moths [34]. As a result, the expanded OR19 homologs in primitive species were later diversified to canonical PRs, establishing the advanced sex pheromone-based communication system.

Canonical PRs of ancient moth species were broadly characterized [13,35]. In this study, we show that the TxiaOR19 array and LarmPR1 share motifs that reflect some exons on the loci. These motifs were separated before the evolution of Lepidoptera. Considering that motif shifts accompany similar transposable element arrangements within the tested OR loci, it is likely that the first PR emerged from exonization, driven by TE activities. This effect has been commonly observed in other organisms [36]. On the other hand, the shared motif regions could be traced back to earlier dipteran species, which also had similar large OR duplications such as in *Bactrocera dorsalis* [37]. The disappearance of large tandem arrays in later lepidopterans suggests the separation of ancestral duplicated ORs. This can be supported by scattered chromosome

linearization and increased DNA transposons during TE activity bursts. The duplicated ORs themselves could reflect rapid olfactory evolution for species adaptation [38]. Although TEs may not be determining factors for the functional emergence of ORs, as shown in the clonal raider ant *Ooceraea biroi* [39], we cannot exclude possible TE involvement in PR emergence due to the horizontally diverged TE landscapes among lepidopteran ORs.

Modern moths show larger interspecies variations and increased numbers of glomeruli in antennal lobes, suggesting potential positive selection in olfactory systems, along with their distribution in different ecological niches [40]. Enlarged MGCs in the butterfly *Pieris rapae* suggest that the sexual dimorphic sex pheromone recognition system is widely used by Lepidoptera [41]. However, the sexual dimorphic olfactory neuropils are not suitable for Himalaya ghost moths, as females have also retained the enlarged LFGs. We argue that female LFGs are more likely involved in mating allocation rather than egg-laying orientation since these ghost moths spray eggs in nature, unlike the majority of modern moth egg-laying behavior on the selected locations [32,42]. Therefore, they may not require a sophisticated olfactory system for precise assessment of corresponding sites. This is supported by the contractions of OR repertoires in all observed ghost moth species. These cues also suggest the ancestral roles of the ghost moths in terms of their sex role systems.

Himalaya ghost moths retain ancestral traits of olfactory system for equal sex roles, which may be attributed to the mechanisms of asymmetric divergence and redundant genome formation. The lack of sexual dimorphism in the antennal lobes and expansion of TxiaOR19 array other than canonical PRs also contribute to the non-biased sex role differentiation within this primitive lineage. Overall, these findings highlight the unique evolutionary features of Himalaya ghost moths and shed light on the mechanisms shaping olfactory systems in insects.

Materials and Methods

Insects

Newly emerged lepidopteran species from lab colonies were sexed and 3-5 d adults were used in all tests. *A. jianchuanensis*, *T. armoricanus*, *T. xiaojinensis*, *Agrotis ipsilon*, *S. frugiperda*, *Galleria mellonella*, and *P. rapae* were obtained from Institute of Zoology, Guangdong Academy of Sciences. *H. cunea* were obtained from Chinese Academy of Forestry. *Athetis dissimilis* were obtained from Henan University of Science and Technology. *Helicoverpa armigera* and *Helicoverpa assulta* were obtained from Henan Agricultural University. *Mythimna separata*, *Cydia pomonella*, and *S. litura* were obtained from Institute of Plant Protection, Chinese Academy of Agricultural Sciences. *S. exigua* were obtained from Qingdao Agricultural University. *P. xylostella* were obtained from Shanxi Agricultural University. All lab colonies were regularly rejuvenated with natural populations.

Morphometric measurement

A total of 7 strains of Himalaya ghost moths were measured by the lengths of antennae and forewings. Three *T. xiaojinensis* strains were from lab colony mentioned above, and Xiaojin (N30.99, E102.27), Hongkou (N31.16, E103.84) field populations. The other four strains consisted of two *A. jianchuanensis* populations collected from Jiulong (N28.99, E101.51), Gongga (N29.56, E101.98), and two *T. armoricanus* populations from Yala (N30.11, E102.25), Kangding (N30.08, E101.97), respectively. Intact appendages were removed and embedded with glass slides before processed under an AXIO Imager microscope (Zeiss, Jena, Germany) equipped with an Axiocam 512 camera (Zeiss). A ZEN 2.3 software (Zeiss) was used to acquire scale bar labelled photographs of antennae and wings. Lengths of interest were manually assigned to the scale bar with ImageJ

1.53f51 (National Institute of Health, USA) and then recorded. A total 5 to 21 replicates were carried out for each strain, and means were used to develop olfactory indexes by the formula [antenna/wing]. Data of other species were referred to the previous publication [26].

Scanning electron microscopy

The antennae of 1-3 d adults were cut from base and fixed in 0.25% glutaraldehyde at 4 °C overnight. After three washes at room temperature with 0.1 M phosphate-buffered saline (PBS, pH 7.4), antennae were dehydrated through a laddered ethanol series (30, 50, 70, 80, 90, and 100%) and dried in a critical point drier (Bal-Tel CPD 030) before mounted on aluminum stubs. The mounted antennae were coated with gold spray (Bal-Tel SCD 005) and observed with SEM instrument (FEI Quanta 200). Sensillar distributions of compared species were from reported works (Table S2).

Antennal lobe atlas

Lepidopteran brains were labeled according to the previous work [19]. Newly dissected intact brains were successively processed with 4% paraformaldehyde in 0.1 M PBS for fixation (24 h), pre-incubating with 5% normal goat serum in 0.1 M PBS containing 0.5% Triton X-100 (NGS-PBST) (0.5 h), incubating with 1% SYNORF1 (Developmental Studies Hybridoma Bank, University of Iowa) in 5% NGS-PBST (72 h), and incubating with Alexa Fluor 488 goat anti-mouse (Invitrogen, Eugene, OR, USA) at 1:500 with 1% NGS-PBST (48 h). After rinsed for six times in PBS and dehydrated with laddered ethanol series, brain samples were mounted with antifade mounting medium (Beyotime, Shanghai, China) in a perforated aluminum slide which was sandwiched by two glass coverslips. Three brains of each sex from each species were prepared for imaging.

All image stacks were acquired with a confocal laser scanning microscopy system with a 10-

20x objective. Data for *A. jianchuanensis*, *T. armoricanus*, *T. xiaojinensis*, *G. mellonella*, and *A. ipsilon* were collected with FV3000 (Olympus, Tokyo, Japan). Data for *H. cunea*, *A. dissimilis*, *H. armigera*, *H. assulta*, *M. separata*, *S. litura*, and *P. xylostella* were collected with LSM 780 (Zeiss). Data for *S. frugiperda*, *P. rapae*, *C. pomonella*, and *S. exigua* were collected with A1 HD25 (Nikon, Tokyo, Japan). An argon laser at 488 nm was used to excite the Alexa Fluor. The resolution of the x-axis was 500 - 2,048 voxels and the section interval was set to 3 or 5 μm. Amira software (AMIRA 5.3, Visage Imaging, Fürth, Germany) was used as previously described to conduct segmentation, tissue statistics, and three dimensional reconstructions of the antennal lobes [19].

Genome and transcriptome sequencing

Genomic DNA of *T. xiaojinensis* larva was extracted for library establishment, and then sequenced with Nanopore PromethION platform (Oxford Nanopore Technology, Oxford, UK). After quality control, a total 319.9 Gb clean data was assembled by using NextDenovo (https://github.com/Nextomics). minimap2 was used to map separately prepared Illumina short reads onto the genome assembly which was later polished by NextPolish (https://github.com/Nextomics). Contigs from non-insect organisms were removed from the final assembly with referring to NT database.

To obtain a chromosome-level assembly, Hi-C scaffolding was further carried out with the same larval sample following reported protocols [43-45]. Specifically, samples were fixed using 2% formaldehyde to establish cross-links, followed by cell lysis and sample quality assessment through extraction. Chromatin digestion was carried out using a restriction endonuclease, with enzyme cleavage efficacy evaluated through sampling. Subsequent steps included biotin-14-dCTP (Invitrogen) labeling, blunt-end ligation, DNA purification, and Hi-C sample preparation. After passing quality control, Hi-C fragments underwent end-biotin removal, sonication, end repair, A-

tailing, and adapter ligation to form ligated products. Subsequent PCR steps were amplified to generate library enriched products. Library amplification products were sampled for Hi-C fragment junction quality control, and the entire library preparation was sequenced using Illumina HiSeq with a PE150 sequencing strategy (NextOmics Biotech. lnc., Wuhan, China). The fastp v.0.12.6 (RRID:SCR_016962) with default parameters was used to filter the raw sequences, resulting in high-quality clean reads. The sequenced Reads1 and Reads2 were separately aligned to the assembled genome sequence using bowtie2 v.2.3.2 (end-to-end alignment mode, parameters: --very-sensitive -L 30) (RRID:SCR_016368) to obtain the alignment information. For the unmapped reads after alignment, we searched for reads containing ligation junction sites, trimmed them, and performed alignment again. Finally, the alignment results were combined, and the proportion of Unique Mapped Paired-end Reads was calculated. The LACHESIS software (RRID:SCR_017644) was used to cluster the Contig sequences of the draft assembly into chromosome groups using agglomerative hierarchical clustering. The final genome was further assessed with BUSCO [46] for completeness.

Second generation genome of *T. armoricanus* was obtained from the DNA of a fourth instar larva without gut. A total of 23 different insert size libraries were constructed and 67 lanes were sequenced on Illumina HiSq2000, harvesting 1,344.5 Gb raw data and 877.7 Gb filtered data. The genome was assembled using SOAPdenovo (v2.04) [47] and SSPACE (v2.0) [48] software. We used all 549.3 Gb (180.4×) clean data of short insert size libraries to construct contigs and all 877.7 Gb (266.4×) clean data to construct scaffolds. 283.4 Gb (86.0×) data of large insert size libraries was used again to construct scaffolds by using SSPACE. Then all clean data of short insert size libraries was used to fill the gaps. TrimDup3 (Rabbit2.6) (https://github.com/gigascience/rabbit-genome-assembler) was used to remove the large redundant sequences. The final assembly

presented 3,168 Mb total length of the scaffolds, with N50 of 27.8 kb and 176.2 kb for contigs and scaffolds, respectively. RNA-seq data from 14 different developmental stages of *T. armoricanus* was assembled by Trinity v2.4.0 [49] and was mapped to the assembled genome sequence using BLAT (v. 34) [50], to check the coverage rate. The results showed that 96.8% of the sequences could be mapped to the assembly.

Respective antennae, heads, and labial palps from *A. jianchuanensis* and *T. xiaojinensis* were collected in liquid nitrogen and sequenced with Illumina according to the manufactural instructions. The transcriptomes were assembled by Trinity v2.4.0 [49] with default parameters.

Phylogenetic analysis and estimation of divergence time

To reconstruct the phylogenetic tree of 16 lepidopteran insect species with two outgroups of *Tribolium castaneum* and *D. melanogaster*. Except for three species of *A. dissimilis*, *T. xiaojinensis*, *A. jianchuanensis*, we first downloaded the genome annotations or raw data of transcriptomes for other 15 species from NCBI (Table S1). The transcripts were assembled by Trinity v2.4.0 [49] with default parameters. Subsequently, the orthologous genes of these 18 insect species were inferred from their genomes or transcriptomes by using OrthoFinder [51] with the default parameters. Single-copy orthologues from each species were selected for phylogenetic reconstruction. The protein sequences of each orthologue were independently aligned with MAFFT v7.407 [52], and the aligned results were trimmed by trimAl [53] to remove low-quality regions with the parameter "-automated1", the trimmed sequences were concatenated into a single super sequence. RAxML [54] was then used with the VT + F model, which is inferred by ProtTest v3.4.2 [55], to estimate a maximum likelihood tree starting with 1000 bootstraps followed by likelihood optimization.

We used the r8s (V1.7.1) [56] to estimate the divergence time. The phylogenetic tree

constructed by RAxML [54] was used as an input tree. A smoothing parameter of 3 was selected, which was estimated by the cross-validation approach (with parameters “cvstart=0, cvinc=1, cvnum=18”). The calibration points were: 1) the most recent common ancestor of the clade including *T. castaneum* and *P. xylostella*, constrained to be 337 Mya (million years ago); 2) the most recent common ancestor of the clade including *D. melanogaster* and *C. pomonella*, constrained to be 318 Mya; and 3) the most recent common ancestor of the clade including *P. rapae* and *S. litura*, constrained to be 125 Mya [34].

Annotation of Or *gene family*

The protein sequences of lepidopteran insect ORs were collected from NCBI. These protein sequences were then used as queries in iterative BLASTP searches with parameter “-evalue 1e-5” against the assembly of the three ghost moth species to find candidate *Or* genes. A local command line HMMER (version 3.1b2) [57] search was conducted for these candidate ORs against the Pfam-A database (http://pfam.xfam.org) to find the 7tm_6 (PF02949) or 7tm_4 (PF13853) HMM profile for ORs. FGENESH 2.6 [29] prediction of potential genes was done for contigs of interests. Annotated ORs were verified with PCRs (Table S3). Data from other species were collected according to the reported works (Table S4).

Characterizations of Ors

CDS cloning verifications were carried out targeting on annotated *TxiaOrs* using adult antennal cDNA. Gene-specific primers were designed (Table S3) and PCRs were done on a Veriti 96-well thermal cycler (Applied Biosystems, MA, USA) using High Fidelity (HiFi) PCR SuperMix (Trans, Beijing, China). Products were processed with 1% agarose (BBI, Shanghai, China) on a PowerPac electrophoresis system (Bio-Rad, CA, USA) and visualized with a GelDoc-It TS3315 imaging system (UVP, CA, USA). Multiple bands such as for *TxiaOr18* were separately collected

and purified with a gel extraction kit (GenStar, Beijing, China) before Sanger sequencing (Sangon Biotech, Shanghai, China). Later analysis was based on the longest sequenced *TxiaOrs* for each locus. *Or* expressions were showed as autoscaled heatmaps indicating the FPKM (Fragments Per Kilobase of transcript per Million mapped reads) which were calculated by RSEM [58] from head, antenna, and labial palp transcriptomes of adult ghost moths.

Phylogenetic analysis of 272 ORs were carried out with the above mentioned protocol using MAFFT [52], trimAl [53], and RAxML [54], but with the best fit estimated model of LG+F. Verifications were done to the tree topology with MEGA X [59] and MrBayes 3.2.6 [60] to establish the NJ tree based on Dayhoff model and BY tree based on Blosum62 model, respectively. Homologs of TxiaOR19 array were predicted by CLANS [28] using the blastx results against the NCBI nr database. For chromosome linearization tests, local tblastn was applied to map the selected ORs towards chromosomes of each species (Table S1) and results were visualized as circos plots by using TBtools v1.113 [61]. Evolution of mapped ORs were inferred using MrBayes 3.2.6 [60] under JTT+F+G4 model (2 parallel runs, 200,000 generations), in which the initial 25% of sampled data were discarded as burn-in. The final average standard deviation of split frequencies was 0.069772. Protein motifs were predicted with MEME Suite v5.5.2 [62].

Annotation of repeats and transposable element families

For transposable element analysis, we first performed the *de novo* predictions for each species by RepeatModeler version open-1.0.11 (https://github.com/Dfam-consortium/RepeatModeler) to generate a specific library. Then we annotated the genome assembly by RepeatMasker version open-4.0.7 with the “ncbi” search algorithm. Annotated transposable element sequences were manually verified and classified with Dfam [63]. The calcDivergenceFromAlign.pl and createRepeatLandscape.pl scripts in the RepeatMasker package were used to calculate the Kimura

divergence values and plot the repeat landscape, respectively. Estimations for transposable element burst times were based on the recently reported substitution rate of 6.19 x 10^{-10} per site per generation in arthropods [64].

Chemical analysis

Hexane extraction method was adopted from our previous works on moth pheromone identifications [65]. Abdomen tips of calling adult *T. xiaojinensis* were cut with dissection scissors and immediately put in 20 µl hexane (HPLC purity, Kermel Chemical Reagent Co., Tianjin, China) which kept at 4 °C for 1 d prior to the test. Head space SPME method was adopted from our previous works on body surface volatile emissions of insects [66]. Newly emerged male or female adults were kept in a mesh cage in separated rearing chambers for sampling. A 50/30 µm DVB/CAR/PDMS stableflex fiber (Supelco, Bellefonte, PA, USA) was penetrated into the cage for sampling at 10 °C for 24 h. The volatile blends sampled were either injected for 1 µl or subjected to an Agilent 7890B GC - 5977 MSD coupled system equipped with a HP-5MS column (0.25 µm x 30 m x 0.250 mm) (Agilent, Palo Alto, CA, USA). A 60 min oven temperature program was used following: 40 °C for 2 min, 40 °C to 150 °C at 5 °C/min, 150 °C for 2 min, 150 °C to 200 °C at 10 °C/min, 200 °C for 5 min, 200 °C to 230 °C at 5 °C/min, and 230 °C for 18 min. Raw data were analyzed with MSD ChemStation (G1701FA F. 01. 03. 2357) by searching against a NIST 17 MS library (Agilent). A total 40 individuals were tested for SPME from two stratified groups. Each hexane extraction sample included 20 individuals and at least 3 replicates were done towards each sex.

Docking simulation

TxiaOR19 and the other four sex biased OR sequences of *T. xiaojinensis* were predicted by AlphaFold2 [67] for their tertiary structures. The 3D structures of 18 ligands were downloaded from

PubChem [68]. The Molecular Operating Environment software (MOE, Chemical Computing Group ULC, Montreal, Canada) was used to dock the ligands with ORs. Briefly, ORs were prepared using MOE QuickPrep and ligands were energy minimized with the MOE Energy Minimize prior to the simulation. Triangle Matcher algorithm was selected for placement and 30 top-scoring placement poses were selected by the London dG empirical scoring function, while the rigid receptor was selected for refinement and top-scoring poses were selected by the GBVI/WSA dG empirical scoring function. The binding free energy of respective OR-ligand was estimated by using S Score function and later used for establishment of colour coded map.

Courtship arena

The assays were carried out using 1 d emerged naïve moths at peak mating hours 18 - 20 pm during sunset. One randomly chosen pair of *T. xiaojinensis* adults was placed in a paper funnel and recorded for 1 h. A total 20 pairs were tested and recorded for calling and tracing behaviors. Recorded footages were processed through the idTracker [69] pipeline to obtain the velocities of moths showing as per pixel distances per min. Fluttering behaviors were observed by manually checking each video file.

Statistics and data processing

Comparison of means was done by using either unpaired *t* test or GLM followed by multiple comparisons according to treatment sizes (SPSS 22.0.0.0, IBM Corp., Armonk, NY, USA). Simple linear regression and data plotting were done using Prism 5.01 (GraphPad software, San Diego, CA, USA). Multivariate tests were carried out with MetaboAnalyst 5.0 [70] server which integrates R statistics (https://www.r-project.org). All error bars indicate standard errors of the means otherwise indicated in the figure legends.

Author contributions

Rui Tang: Conceptualization, Methodology, Data curation, Formal analysis, Investigation, Visualization, Writing-original draft, Writing-review & editing. **Cong Huang**: Methodology, Data curation, Formal analysis, Visualization, Writing-review & editing. **Jun Yang**: Data curation, Writing-review & editing. **Zhong-Chen Rao**: Methodology, Formal analysis, Writing-review & editing. **Li Cao**: Data curation, Writing-review & editing. **Peng-Hua Bai**: Data curation, Writing-review & editing. **Xin-Cheng Zhao**: Methodology, Data curation, Writing-review & editing. **Jun-Feng Dong**: Data curation, Writing-review & editing. **Xi-Zhong Yan**: Data curation, Writing-review & editing. **Fang-Hao Wan**: Formal analysis, Writing-review & editing. **Nan-Ji Jiang**: Conceptualization, Methodology, Data curation, Investigation, Writing-original draft, Writing-review & editing. **Ri-Chou Han**: Conceptualization, Investigation, Supervision, Writing-review & editing.

Declaration of Interest

The authors declare no competing interests.

Acknowledgments

We thank Dr. Zhang Bin and Dr. Meng Xiang for supporting on insect materials. We thank Zhongkai University of Agriculture and Engineering, South China Normal University, and Institute of Zoology, CAS for sharing the research platforms to assist confocal imaging. We thank B.F.A. Yorda for the development of insect schematics.

Funding information

The current research was funded by the Major Science and Technology Project of Qinghai Province (No. 2021-SF-A4-1), GDAS Special Project of Science and Technology Development (2022GDASZH-2022010106), Guangzhou Science and Technology Project (No. 202201010039), and Guangdong Basic and Applied Basic Research Foundation (No. 2020A1515011366).

Data availability

All data are available in the main text or the supplementary materials. The whole genome sequence data of *T. xiaojinensis* reported in this paper have been deposited in NCBI (Bioproject: PRJNA1006505).

References

1 Shine, R. Ecological causes for the evolution of sexual dimorphism: a review of the evidence. The Quarterly Review of Biology 64, 419-461 (1989).

2 Ritchie, M. G. Sexual selection and speciation. Annual Review of Ecology, Evolution, and Systematics 38, 79-102 (2007).

3 Andersson, M. & Iwasa, Y. Sexual selection. Trends in Ecology & Evolution 11, 53-58 (1996).

4 Kokko, H. & Jennions, M. D. Parental investment, sexual selection and sex ratios. Journal of Evolutionary Biology 21, 919-948 (2008).

5 Datta, S. R., Vasconcelos, M. L., Ruta, V., Luo, S., Wong, A., Demir, E. et al. The *Drosophila* pheromone cVA activates a sexually dimorphic neural circuit. Nature 452, 473-477 (2008).

6 Allen, C. E., Zwaan, B. J. & Brakefield, P. M. Evolution of sexual dimorphism in the Lepidoptera. Annual Review of Entomology 56, 445-464 (2011).

7 Butenandt, v. A. Uber den sexsual-lockstoff des seidenspinners *Bombyx mori*. Reindarstellung und konstitution. Z. Naturforschg, b 14, 283 (1959).

8 Sakurai, T., Namiki, S. & Kanzaki, R. Molecular and neural mechanisms of sex pheromone reception and processing in the silkmoth *Bombyx mori*. Frontiers in Physiology 5, 125 (2014).

9 Stork, N. E. How many species of insects and other terrestrial arthropods are there on Earth? Annual Review of Entomology 63, 31-45 (2018).

10 Löfstedt, C., Wahlberg, N. & Millar, J. Evolutionary patterns of pheromone diversity in Lepidoptera. Pheromone communication in moths: evolution, behavior and application, 43-82 (University of California Press 2016).

11 Ando, T., Inomata, S. I. & Yamamoto, M. Lepidopteran sex pheromones. The chemistry of pheromones and other semiochemicals I, 51-96 (Springer, 2004).

12 Zhang, D. D. & Löfstedt, C. Moth pheromone receptors: gene sequences, function, and evolution. Frontiers in Ecology and Evolution 3, 105 (2015).

13 Yuvaraj, J. K., Andersson, M. N., Corcoran, J. A., Anderbrant, O. & Löfstedt, C. Functional characterization of odorant receptors from *Lampronia capitella* suggests a non-ditrysian origin of the lepidopteran pheromone receptor clade. Insect Biochemistry and Molecular Biology 100, 39-47 (2018).

14 Bastin-Héline, L., De Fouchier, A., Cao, S., Koutroumpa, F., Caballero-Vidal, G., Robakiewicz, S. et al. A novel lineage of candidate pheromone receptors for sex communication in moths. eLife 8, e49826 (2019).

15 Hildebrand, J. G. & Shepherd, G. M. Mechanisms of olfactory discrimination: converging evidence for common principles across phyla. Annual Review of Neuroscience 20, 595-631 (1997).

16 Zhang, J., Walker, W. B. & Wang, G. Pheromone reception in moths: from molecules to behaviors. Progress in Molecular Biology and Translational Science 130, 109-128 (2015).

17 Matsumoto, S. & Hildebrand, J. G. Olfactory interneurons in the moth *Manduca sexta*: Response characteristics and morphology of central neurons in the antennal lobes. Proceedings of the Royal Society of London. Series B. Biological Sciences 213, 249-277 (1981).

18 Rössler, W., Tolbert, L. P. & Hildebrand, J. G. Early formation of sexually dimorphic glomeruli in the developing olfactory lobe of the brain of the moth *Manduca sexta*. Journal of Comparative Neurology 396, 415-428 (1998).

19 Zhao, X. C., Ma, B. W., Berg, B. G., Xie, G. Y., Tang, Q. B. & Guo, X. R. A global-wide search for sexual dimorphism of glomeruli in the antennal lobe of female and male *Helicoverpa armigera*. Scientific Reports 6, 1-9 (2016).

20 Mallet, J. Sex roles in the ghost moth *Hepialus humuli* (L.) and a review of mating in the Hepialidae (Lepidoptera). Zoological Journal of the Linnean Society 80, 67-82 (1984).

21 Kawahara, A. Y., Plotkin, D., Espeland, M., Meusemann, K., Toussaint, E. F., Donath, A. et al. Phylogenomics reveals the evolutionary timing and pattern of butterflies and moths. Proceedings of the National Academy of Sciences 116, 22657-22663 (2019).

22 Han, R., Wu, H., Tao, H., Qiu, X., Liu, G., Rao, Z. et al. Research on Chinese cordyceps during the past 70 years in China. Chinese Journal of Applied Entomology 56, 849-883 (2019).

23 Wang, Z. & Pierce, N. E. Fine‐scale genome‐wide signature of Pleistocene glaciation in *Thitarodes* moths (Lepidoptera: Hepialidae), host of *Ophiocordyceps* fungus in the Hengduan Mountains. Molecular Ecology 32, 2695-2714 (2023).

24 Wu, H., Cao, L., He, M., Han, R. & De Clercq, P. Interspecific hybridization and complete mitochondrial genome analysis of two ghost moth species. Insects 12, 1046 (2021).

25 Tao, Z., Cao, L., Zhang, Y., Ye, Y. & Han, R. Laboratory rearing of *Thitarodes armoricanus* and *Thitarodes jianchuanensis* (Lepidoptera: Hepialidae), hosts of the Chinese medicinal fungus *Ophiocordyceps sinensis* (Hypocreales: Ophiocordycipitaceae). Journal of Economic Entomology 109, 176-181 (2016).

26 Symonds, M. R., Johnson, T. L. & Elgar, M. A. Pheromone production, male abundance, body size, and the evolution of elaborate antennae in moths. Ecology and Evolution 2, 227-246 (2012).

27 Brigaud, I., Montagné, N., Monsempes, C., François, M. C. & Jacquin‐Joly, E. Identification of an atypical insect olfactory receptor subtype highly conserved within noctuids. The FEBS Journal 276, 6537-6547 (2009).

28 Frickey, T. & Lupas, A. CLANS: a Java application for visualizing protein families based on pairwise similarity. Bioinformatics 20, 3702-3704 (2004).

29 Solovyev, V., Kosarev, P., Seledsov, I. & Vorobyev, D. Automatic annotation of eukaryotic genes, pseudogenes and promoters. Genome Biology 7, 1-12 (2006).

30 Holland, P. W., Marlétaz, F., Maeso, I., Dunwell, T. L. & Paps, J. New genes from old: asymmetric divergence of gene duplicates and the evolution of development. Philosophical Transactions of the Royal Society B: Biological Sciences 372, 20150480 (2017).

31 Krasileva, K. V. The role of transposable elements and DNA damage repair mechanisms in gene duplications and gene fusions in plant genomes. Current Opinion in Plant Biology 48, 18-25 (2019).

32 Nielsen, E. S., Robinson, G. S. & Wagner, D. L. Ghost-moths of the world: a global inventory and bibliography of the Exoporia (Mnesarchaeoidea and Hepialoidea) (Lepidoptera). Journal of Natural History 34, 823-878 (2000).

33 Cheng, R. L., Yu, Y. X., Liu, L. X., Zhang, C. X. & Fang, C. X. A draft genome of the ghost moth, *Thitarodes* (Hepialus) sp., a medicinal caterpillar fungus. Insect Science 23, 326-329 (2016).

34 Wan, F., Yin, C., Tang, R., Chen, M., Wu, Q., Huang, C. et al. A chromosome-level genome assembly of *Cydia pomonella* provides insights into chemical ecology and insecticide resistance. Nature Communications 10, 1-14 (2019).

35 Yuvaraj, J. K., Corcoran, J. A., Andersson, M. N., Newcomb, R. D., Anderbrant, O. & Löfstedt, C. Characterization of odorant receptors from a non-ditrysian moth, *Eriocrania semipurpurella* sheds light on the origin of sex pheromone receptors in Lepidoptera. Molecular Biology and Evolution 34, 2733-2746 (2017).

36 Sela, N., Kim, E. & Ast, G. The role of transposable elements in the evolution of non-mammalian vertebrates and invertebrates. Genome Biology 11, 1-13 (2010).

37 Wang, Y., Fang, G., Xu, P., Gao, B., Liu, X., Qi, X. et al. Behavioral and genomic divergence between a generalist and a specialist fly. Cell Reports 41, 111654 (2022).

38 Nei, M., Niimura, Y. & Nozawa, M. The evolution of animal chemosensory receptor gene repertoires: roles of chance and necessity. Nature Reviews Genetics 9, 951-963 (2008).

39 McKenzie, S. K. & Kronauer, D. J. The genomic architecture and molecular evolution of ant odorant receptors. Genome Research 28, 1757-1765 (2018).

40 Engsontia, P., Sangket, U., Chotigeat, W. & Satasook, C. Molecular evolution of the odorant and gustatory receptor genes in lepidopteran insects: implications for their adaptation and speciation. Journal of Molecular Evolution 79, 21-39 (2014).

41 Montgomery, S. H. & Ott, S. R. Brain composition in *Godyris zavaleta*, a diurnal butterfly, reflects an increased reliance on olfactory information. Journal of Comparative Neurology 523, 869-891 (2015).

42 Renwick, J. & Chew, F. Oviposition behavior in Lepidoptera. Annual Review of Entomology 39, 377-400 (1994).

43 Shi, J., Ma, X., Zhang, J., Zhou, Y., Liu, M., Huang, L. et al. Chromosome conformation capture resolved near complete genome assembly of broomcorn millet. Nature Communications 10, 464 (2019).

44 Servant, N., Varoquaux, N., Lajoie, B.R., Viara, E., Chen, C.-J., Vert, J.-P. et al. HiC-Pro: an optimized and flexible pipeline for Hi-C data processing. Genome Biology 16, 1-11 (2015).

45 Burton, J.N., Adey, A., Patwardhan, R.P., Qiu, R., Kitzman, J.O. & Shendure, J. Chromosome-scale scaffolding of de novo genome assemblies based on chromatin interactions. Nature Biotechnology 31, 1119-1125 (2013).

46 Simão, F.A., Waterhouse, R.M., Ioannidis, P., Kriventseva, E.V. & Zdobnov, E.M. BUSCO: assessing genome assembly and annotation completeness with single-copy orthologs. Bioinformatics 31, 3210-3212 (2015).

47 Luo, R., Liu, B., Xie, Y., Li, Z., Huang, W., Yuan, J. et al. SOAPdenovo2: an empirically improved memory-efficient short-read de novo assembler. Gigascience 1(1), 2047-217X (2012).

48 Boetzer, M., Henkel, C. V., Jansen, H. J., Butler, D. & Pirovano, W. Scaffolding pre-assembled contigs using SSPACE. Bioinformatics 27, 578-579 (2011).

49 Haas, B. J., Papanicolaou, A., Yassour, M., Grabherr, M., Blood, P. D., Bowden, J. et al. *De novo* transcript sequence reconstruction from RNA-seq using the Trinity platform for reference generation and analysis. Nature Protocols 8, 1494-1512 (2013).

50 Kent, W. J. BLAT - the BLAST-like alignment tool. Genome Research 12, 656-664 (2002).

51 Emms, D. M. & Kelly, S. OrthoFinder: phylogenetic orthology inference for comparative genomics. Genome Biology 20, 1-14 (2019).

52 Katoh, K. & Standley, D. M. MAFFT multiple sequence alignment software version 7: improvements in performance and usability. Molecular Biology and Evolution 30, 772-780 (2013).

53 Capella-Gutiérrez, S., Silla-Martínez, J. M. & Gabaldón, T. trimAl: a tool for automated alignment trimming in large-scale phylogenetic analyses. Bioinformatics 25, 1972-1973 (2009).

54 Stamatakis, A. RAxML version 8: a tool for phylogenetic analysis and post-analysis of large phylogenies. Bioinformatics 30, 1312-1313 (2014).

55 Darriba, D., Taboada, G. L., Doallo, R. & Posada, D. ProtTest 3: fast selection of best-fit models of protein evolution. Bioinformatics 27, 1164-1165 (2011).

56 Sanderson, M. J. r8s: inferring absolute rates of molecular evolution and divergence times in the absence of a molecular clock. Bioinformatics 19, 301-302 (2003).

57 Eddy, S. R. Accelerated profile HMM searches. PLoS Computational Biology 7, e1002195 (2011).

58 Li, B. & Dewey, C. N. RSEM: accurate transcript quantification from RNA-Seq data with or without a reference genome. BMC Bioinformatics 12, 1-16 (2011).

59 Kumar, S., Stecher, G., Li, M., Knyaz, C. & Tamura, K. MEGA X: molecular evolutionary genetics analysis across computing platforms. Molecular Biology and Evolution 35, 1547 (2018).

60 Ronquist, F., Teslenko, M., Van Der Mark, P., Ayres, D. L., Darling, A., Höhna, S. et al. MrBayes 3.2: efficient Bayesian phylogenetic inference and model choice across a large model space. Systematic Biology 61, 539-542 (2012).

61 Chen, C., Chen, H., Zhang, Y., Thomas, H. R., Frank, M. H., He, Y. et al. TBtools: an integrative toolkit developed for interactive analyses of big biological data. Molecular Plant 13, 1194-1202 (2020).

62 Bailey, T. L., Boden, M., Buske, F. A., Frith, M., Grant, C. E., Clementi, L. et al. MEME SUITE: tools for motif discovery and searching. Nucleic Acids Research 37, W202-W208 (2009).

63 Hubley, R., Finn, R. D., Clements, J., Eddy, S. R., Jones, T. A., Bao, W. et al. The Dfam database of repetitive DNA families. Nucleic Acids Research 44, D81-D89 (2016).

64 Shao, C., Sun, S., Liu, K., Wang, J., Li, S., Liu, Q. et al. The enormous repetitive *Antarctic krill* genome reveals environmental adaptations and population insights. Cell 186, 1279-1294 (2023).

65 Jiang, N. J., Tang, R., Wu, H., Xu, M., Ning, C., Huang, L. Q. et al. Dissecting sex pheromone communication of *Mythimna separata* (Walker) in North China from receptor molecules and antennal lobes to behavior. Insect Biochemistry and Molecular Biology 111, 103176 (2019).

66 Liu, J., Zhang, R., Tang, R., Zhang, Y., Guo, R., Xu, G. et al. The role of honey bee derived aliphatic esters in the host-finding behavior of *Varroa destructor*. Insects 14, 24 (2022).

67 Jumper, J., Evans, R., Pritzel, A., Green, T., Figurnov, M., Ronneberger, O. et al. Highly accurate protein structure prediction with AlphaFold. Nature 596, 583-589 (2021).

68 Kim, S., Chen, J., Cheng, T., Gindulyte, A., He, J., He, S. et al. PubChem 2019 update: improved access to chemical data. Nucleic Acids Research 47, D1102-D1109 (2019).

69 Pérez-Escudero, A., Vicente-Page, J., Hinz, R. C., Arganda, S. & De Polavieja, G. G. idTracker: tracking individuals in a group by automatic identification of unmarked animals. Nature Methods 11, 743-748 (2014).

70 Pang, Z., Chong, J., Zhou, G., de Lima Morais, D. A., Chang, L., Barrette, M. et al. MetaboAnalyst 5.0: narrowing the gap between raw spectra and functional insights. Nucleic Acids Research 49, W388-W396 (2021).

Figure legends

Figure 1. Phylogenomics and olfactory morphology of the ghost moths comparing with other species in Lepidoptera. **(A)** Dated evolutionary tree of Lepidoptera relationships. Two of the non-lepidopteran species were placed on outgroup branches including *D. melanogaster* and *T. castaneum*. The tree was inferred through a maximum-likelihood analysis of 634,106 amino acid sites from 1,547 strict single-copy genes employing VT + F model and 1000 bootstrap replicates. Branch lengths were optimized and node ages estimated using the penalized likelihood (PL) methods with truncated Newton (TN) algorithm in r8s [56]. Scale bar is in millions of years. Data resources were listed in Table S1. **(B)** Adult head development of Hepialidae *T. xiaojinensis* comparing to moth *S. frugiperda* and butterfly *P. rapae*. Orange arrow indicates the labial palp. Blue arrow indicates the proboscis which lacks in the ghost moth. **(C)** Antennal and forewing length comparison among Lepidoptera. O.I. indicates olfactory index which equals antenna/forewing. Data of hepialids were collected in the current work (blue), and other families were referred to published works [26]. **(D)** Antennal sensilla distribution of selected Lepidoptera by scanning electron microscope. Left box chart shows statistics by counting anterior long sensilla trichoidae on per randomly chosen middle segment. Color coded boxes indicate species which were identically marked on the upper photos. Error bars indicate data ranges. Asterisks in black indicate significant differences among species (GLM and Dunnett multiple comparison referring to Hcun. Male: $F_{3, 25} = 12.58$, $P < 0.0001$; Female: $F_{3, 27} = 63.49$, $P < 0.0001$). Color coded asterisks indicate sex difference within species (Unpaired *t* test. Hcun: $t_{10} = 10.65$, $P < 0.0001$; Ajia: $t_{14} = 7.61$, $P < 0.0001$; Tarm: $t_{14} = 4.49$, $P = 0.0005$; Txia: $t_{14} = 4.89$, $P = 0.0002$). Hcun: *H. cunea*, Ajia: *A. jianchuanensis*, Tarm: *T. armoricanus*, Txia: *T. xiaojinensis*. Arrows indicate long sensilla

trichoidae which are potentially involved in pheromone sensing. Below indicates the density distribution of sensilla trichoidae among species included in (A), with data and references provided in Table S2. **(E)** Glomerular counts of tested Lepidoptera observed by confocal laser scanning microscopy system. Red coded bars indicate predicted male MGCs, and green bars indicate female LFGs. Numbers indicate standard errors of means. Lower case letters indicate significant differences of glomerular counts among species (GLM and Tukey HSD, male: $F_{15, 32} = 48.2$, $P < 0.0001$, female: $F_{15, 32} = 30.9$, $P < 0.0001$). **(F)** Comparison of the cumulus considering volume weights in males. Lower case letters indicate significant differences among species (GLM and Duncan multiple comparison, $F_{15, 32} = 6.05$, $P < 0.0001$). **(G)** Comparison of LFG1 volume weights in females. Lower case letters indicate significant differences among species ($F_{15, 32} = 14.91$, $P < 0.0001$). **(H)** PCA plot of MGC measurements with 16 tested Lepidoptera species at 73.8% fraction of explained variance. Representative MGC glomeruli from *A. jianchuanensis*, *T. armoricanus*, and *T. xiaojinensis* were shown. Cu indicates the cumulus.

Figure 2. Evolution of sex pheromone-related odorant receptors among *T. xiaojinensis*, caddisfly, and other Lepidoptera. **(A)** Rooted Maximum Likelihood (ML) tree of 272 selected lepidopteran ORs established by RAxML [54] (Data S3). The evolutionary distances were computed using the LG+F model based on ProtTest3 [55] estimation. The bootstrap test of 1000 replicates was used to infer the final ML tree. Tested species included *A. jianchuanensis* (Ajia), *T. armoricanus* (Tarm), *T. xiaojinensis* (Txia, test), *P. xylostella* (Pxyl), *C. pomonella* (Cpom), *B. mori* (Bmor), *S. exigua* (Sexi), *S. litura* (Slitu), *S. littoralis* (Slit), *Heliothis virescens* (Hvir), *H. armigera* (Harm), *H. assulta* (Hass), *Ectropis grisescens* (Egri), *Operophtera brumata* (Obru), *Agrotis segetum* (Aseg), *Eriocrania semipurpurella* (Esem), and *Lampronia capitella* (Lcap). Tree

topology was verified also with Neighbor-Joining and Bayes approaches (Figure S6). **(B)** Volcano plot (up) shows significantly different expressed *Ors* in *T. xiaojinensis* between male and female adults. Color-coded map (down) showing expression levels of identified *Ors* in *T. xiaojinensis* adult heads, antennae, or labial palps. **(C)** Distributions of selected TxiaOR homologs across species. **(D)** Linearization of TxiaOR18c-TxiaOR19 tandem proteins with chromosomes from selected species showed by circos plot. Reported chromosome assemblies (chr) from *L. marmoratus*, *Micropterix aruncella*, *Incurvaria masculella*, *Nematopogon swammerdamellus*, *Coleophora flavipennella*, *Cydia pomonella*, *Manduca sexta*, *Bombyx mori*, and *Agrotis ipsilon* were used (Table S1). ORs mapped by TxiaOR18c or TxiaOR19 tandem were colored in blue, and those PR mapped were colored in red. **(E)** Bayes tree showing evolutionary relationships of 142 ORs. Bayesian Inference phylogenies were inferred using MrBayes 3.2.6 [60] under JTT+F+G4 model (2 parallel runs, 200,000 generations), in which the initial 25% of sampled data were discarded as burn-in. The final average standard deviation of split frequencies was 0.069772. Color indications were used as per (D). Uncolored ORs were selected according to blastp results of TxiaOR19 array against nr database. Additional species included *Athalia rosae*, *Hermetia illucens*, *Culex quinquefasciatus*, *Aphis gossypii*, *Schistocerca americana*, and *S. cancellata*. **(F)** Motif identification towards PR mapped ORs from caddisfly and moths. **(G)** Motif identification towards TxiaOR18c/19 tandem mapped ORs from caddisfly and moths. **(H)** Distribution of motifs identified in (F) and (G), showing overlaps from PR and TxiaOR18c/19 clades.

Figure 3. Genome and OR evolution reflected by landscapes of transposable elements (TEs). **(A)** Detailed TE landscapes of the ghost moths, caddisfly, and white-barred gold. Times of TE burst events were estimated according to CpG adjusted Kimura substitution levels and a reported

arthropod substitution rate of 6.19 x 10^{-10} per site per generation [64]. **(B)** Comparison of overall TE distributions among caddisfly and moths. **(C)** Aligned Correlation matrix showing arrangement patterns of TEs in TxiaOR18c/19 mapped OR loci. Screened regions for TEs included coding area and respective 5000 bp per up and down stream. Ribbons indicate neighboring of ORs on the same chromosome, and directions were from wider to narrower. Blue colored ORs are from *T. xiaojinensis*, and those in red are ORs mapped simultaneously by TxiaOR18c/19 and classic PRs. **(D)** Overview of asymmetric divergence of duplicated pheromone-related ORs from caddisfly to higher Lepidoptera. The TxiaOR18c/19 duplications predominate in caddisfly and primitive moths, but they were replaced by functional PR duplications in higher moths during evolution.

Figure 4. Resulted dual attraction in sex communications of the ghost moth adults. **(A)** Schematic shows set-up of the courtship arena of *T. xiaojinensis* adults. **(B)** Comparison of calling rates which were reflected by fluttering behaviors in both sexes (Binary test against even distribution, P = 0.33). **(C)** Left shows representative behavioral traces of male and female adults tracked by idTracker [69]. Right shows comparison of distance per min between male and female *T. xiaojinensis* adults (Mann Whitney test, U = 137, P = 0.8055).

Figure1

Click here to access/download;Figure;Figure01.tif

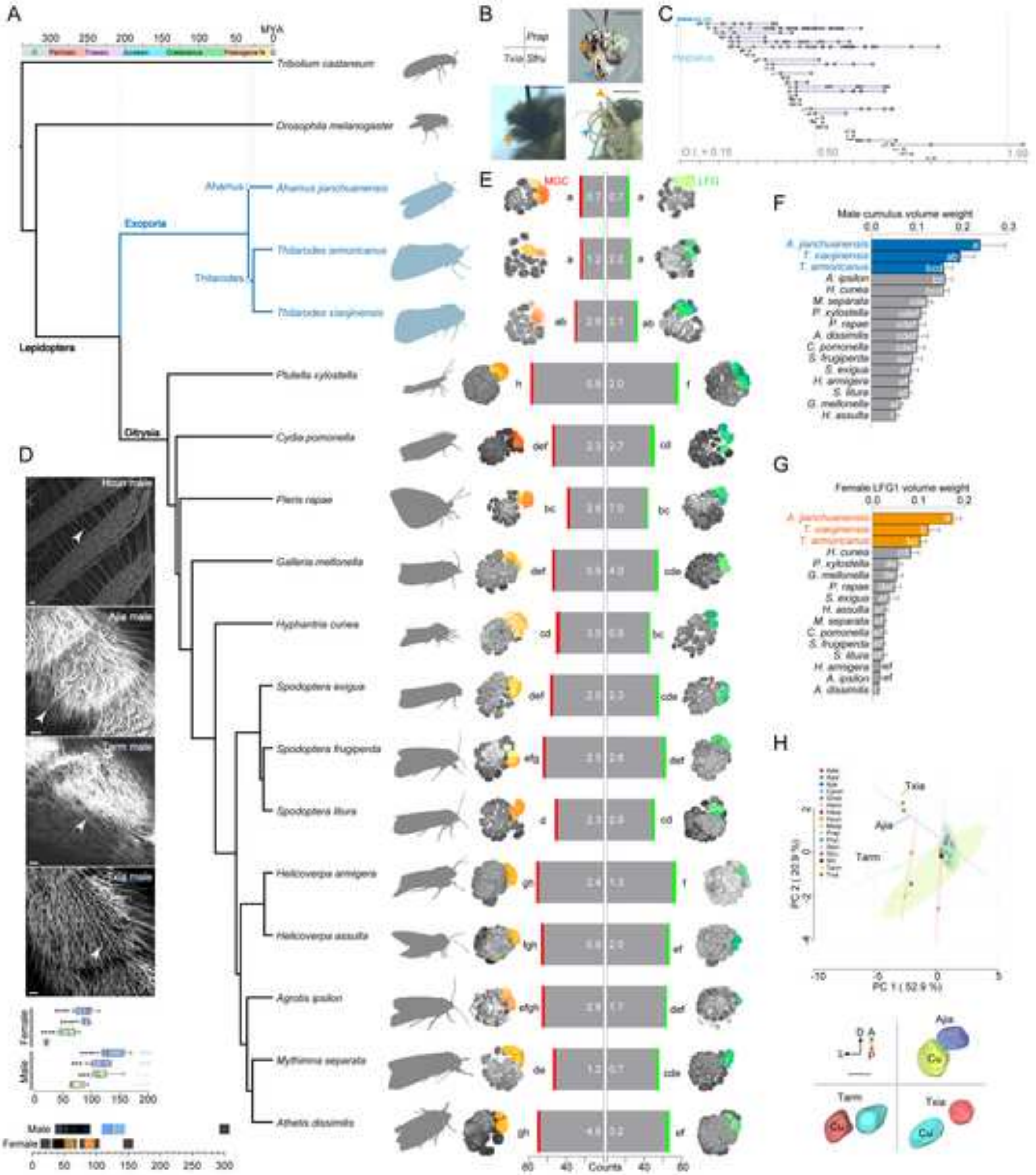

Figure2 Click here to access/download;Figure;Figure02.tif

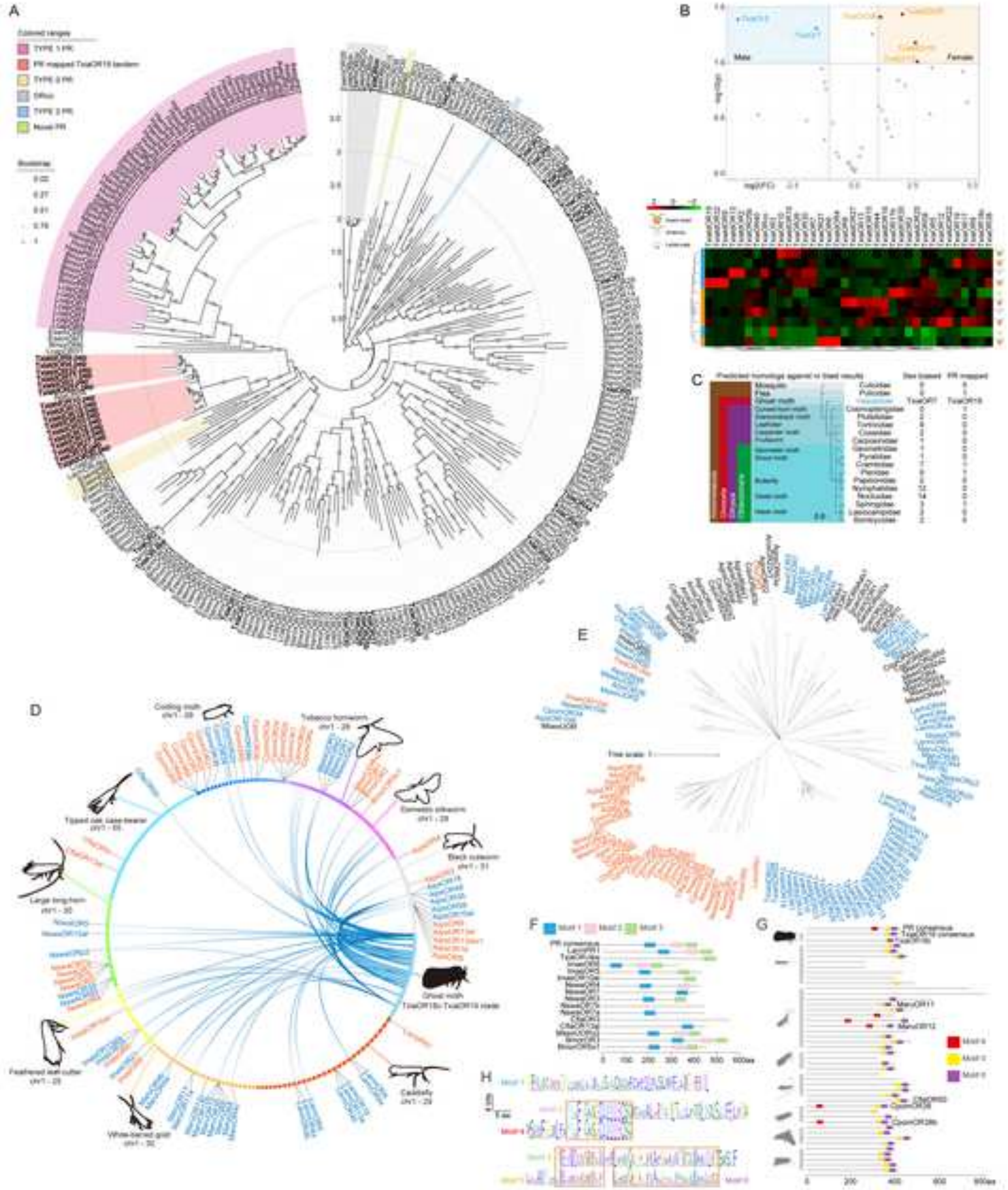

Figure3

Click here to access/download;Figure;Figure03.tif

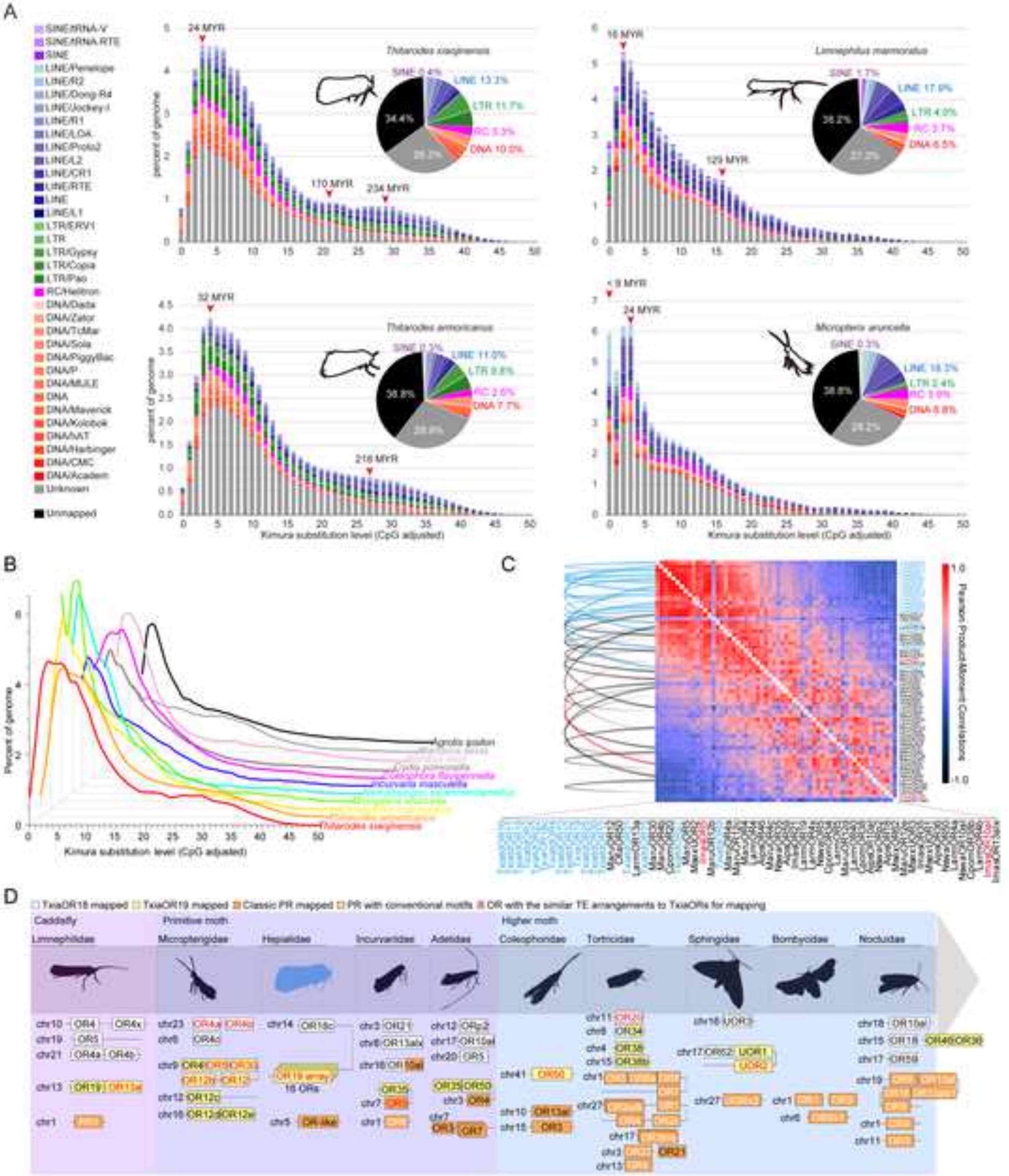

Figure4

Click here to access/download;Figure;Figure04.tif

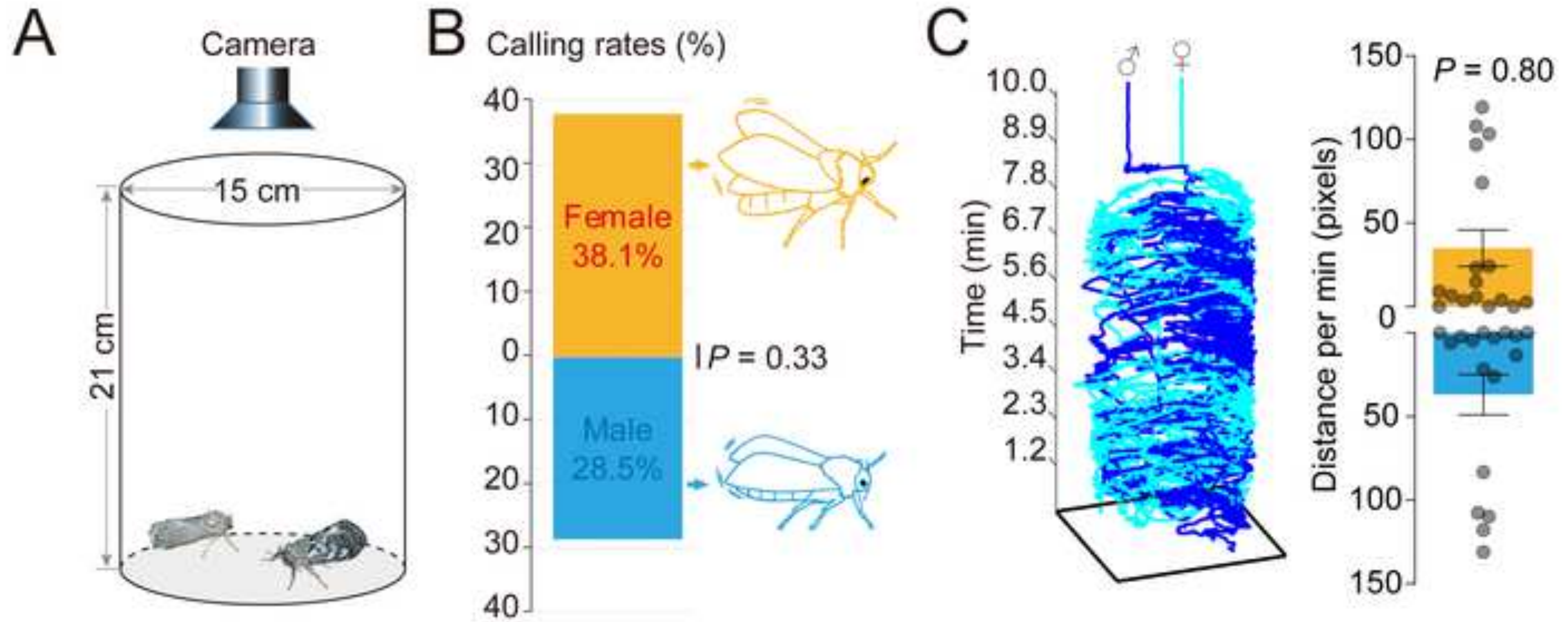

Click here to access/download
Supplementary Material
Supplementary materials.docx

Data S1

Click here to access/download
Supplementary Material
Data S1.xlsx

Data S2

Click here to access/download
Supplementary Material
Data S2.rar

Data S3

Click here to access/download
Supplementary Material
Data S3.fasta

Graphical abstract

Click here to access/download;Personal Cover;Graphical Abstract.tif

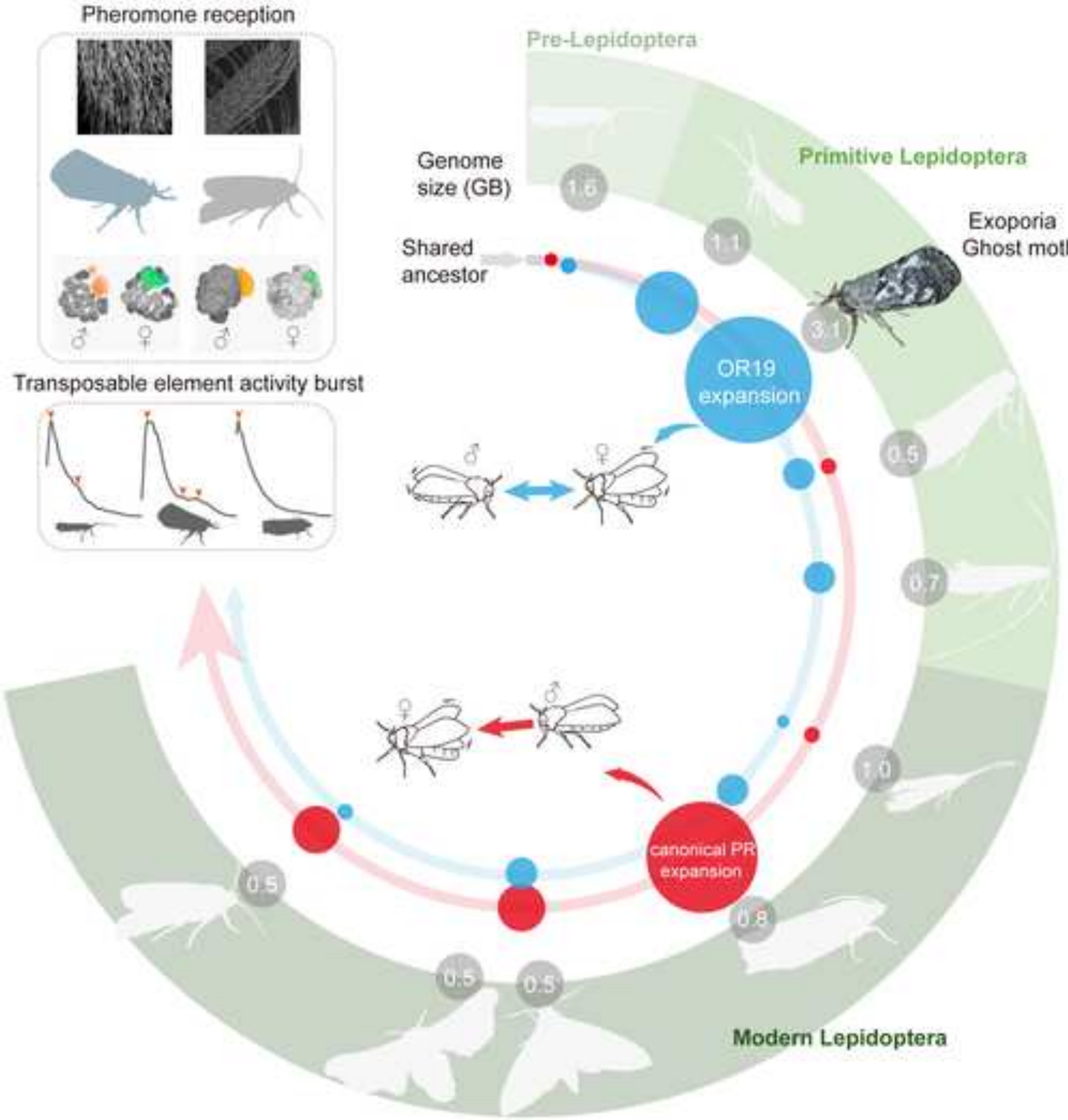

Supplement: giae044_GIGA-D-23-00252_Original_Submission [file giae044_giga-d-23-00252_original_submission.pdf]
