# Supplementary material for: A ghost moth olfactory prototype of the lepidopteran sex communication
Source: Gigascience. 2024 Jul 19;13:giae044. doi: 10.1093/gigascience/giae044 (PMC11258902; doi:10.1093/gigascience/giae044)

GigaScience

A ghost moth olfactory prototype of the lepidopteran sex communication

--Manuscript Draft--

**Manuscript Number:**	GIGA-D-23-00252R1
**Full Title:**	A ghost moth olfactory prototype of the lepidopteran sex communication
**Article Type:**	Research
**Funding Information:**	Major Science and Technology Project of Qinghai Province (2021-SF-A4-1) — Prof Ri-Chou Han<br>National Key Research and Development Program of China (2023YFC2606900) — Dr Rui Tang<br>GDAS Special Project of Science and Technology Development (2022GDASZH-2022010106) — Dr Rui Tang<br>Guangdong Basic and Applied Basic Research Foundation (2020A1515011366) — Prof Ri-Chou Han
**Abstract:**	Sex role differentiation is a widespread phenomenon. Sex pheromones are often associated with sex roles and convey sex-specific information. In Lepidoptera, females release sex pheromones to attract males, which evolve sophisticated olfactory structures to relay pheromone signals. However, in some primitive moths, sex role differentiation becomes diverged. Here, we introduce the chromosome-level genome assembly from ancestral Himalaya ghost moths, revealing a unique olfactory evolution pattern and sex role parity among Lepidoptera. These olfactory structures of the ghost moths are characterized by a dense population of trichoid sensilla, both larger male and female antennal entry parts of brains, compared to the evolutionary later Lepidoptera. Furthermore, a unique tandem of 34 odorant receptor 19 homologs in Thitarodes xiaojinensis (TxiaOr19) has been identified, which presents overlapped motifs with pheromone receptors (PRs). Interestingly, the expanded TxiaOr19 was predicted to have unconventional tuning patterns compared to canonical PRs, with non-sexual dimorphic olfactory neuropils discovered, which contributes to the observed equal sex roles in Thitarodes adults. Additionally, transposable element activity bursts have provided traceable loci landscapes where parallel diversifications occurred between TxiaOr19 and PRs, indicating that the Or19 homolog expansions were diversified to PRs during evolution and thus established the classic sex roles in higher moths. This study elucidates an olfactory prototype of intermediate sex communication from Himalaya ghost moths.
**Corresponding Author:**	Ri-Chou Han, Ph.D.<br>Guangdong Academy of Sciences<br>Guangzhou, Guangdong CHINA
**Corresponding Author Secondary Information:**	
**Corresponding Author's Institution:**	Guangdong Academy of Sciences
**Corresponding Author's Secondary Institution:**	
**First Author:**	Rui Tang, Ph.D.
**First Author Secondary Information:**	
**Order of Authors:**	Rui Tang, Ph.D.
	Cong Huang
	Jun Yang
	Zhong-Chen Rao
	Li Cao

Powered by Editorial Manager® and ProduXion Manager® from Aries Systems Corporation

	Peng-Hua Bai
	Xin-Cheng Zhao
	Jun-Feng Dong
	Xi-Zhong Yan
	Fang-Hao Wan
	Nan-Ji Jiang
	Ri-Chou Han
**Order of Authors Secondary Information:**	

Response to Reviewers:

Dear editor,
Thank you for considering our manuscript publish on the journal Gigascience. We have modified our manuscript very carefully based on both reviewers' suggestions, and it takes some time. All changes we have marked by highlight in the text. More details please see the manuscript or the response letter.
Best regards,
Ri-Chou Han

Reviewer reports:

Reviewer #1: The authors report on the early evolution of the olfactory system within Lepidoptera insects, framed upon the phenomena of intra-specific pheromone communication and subsequent reproductive behaviors, within three species of the primitive Exoporia lineage of Lepidoptera, compared to several other species of the more derived Ditrysian lineage of Lepidoptera. Within evolutionary contexts, olfactory organ morphology, olfactory neuroanatomy, olfactory receptor genes, transposon dynamics within genomic loci, and courtship behaviors are examined. The report is thus broad in scope, with an immense amount of data collected and examined, the methodology appears to be sound and are clearly described, however there are substantial concerns about the presentation of the results, in that the evidence to support claims being made is sometimes not clear or transparent, other times the conclusions drawn do not seem to be supported by the results presented. If these issues can be rectified, the report would provide a substantial contribution to our understanding of the early evolution of olfactory systems and olfactory-based sexual communication within Lepidoptera.
R: We thank you for detailed examinations, suggestions and pointing out our weakness in this manuscript. We, therefore, fully followed those suggestions and check our manuscript carefully, especially for conclusions. We have modified related parts of this manuscript accordingly (also marked by highlight in the text or see in below response letter). We feel that those suggestions do improve our manuscript considerably.

Specific comments are given for each section.

Abstract.
Line 37. "modern Lepidoptera".
What is a modern Lepidoptera? Is it meant to say derived (as compared to ancestral?)
R: We exchange the term "modern Lepidoptera" to the "evolutionary later Lepidoptera". And we hope the term is clearer than before.

Line 40. "Interestingly, the expanded TxiaOR19 does not function as canonical PRs". There were no functional assays conducted on any ORs in this study. This statement should be revised.
R: We revised this sentence as "Interestingly, the expanded TxiaOr19 was predicted to have unconventional tuning patterns compared to canonical PRs".

Introduction.
Line 55-56. "In most insects, such as the vinegar fly, Drosophila melanogaster, males usually release the specific pheromone cVA to gain an advantage in recruiting females."
This sentence seems to be written incorrectly. As it is now, it is claiming that in most insects, males release cVA. Is this what is intended? Or is it meant to say that most in

most insects, males release the specific (sex?) pheromone, such as is seen with Drosophila, with male release of cVA.
R: We apologize for this mistake. We rephrased these sentences as “In insects, sex pheromone becomes an effective investment for male to gain opportunities to mate with female successfully. One well studied example is that, in fruit fly Drosophila melanogaster, males typically release a specific pheromone called cis-11-vaccenyl acetate (cVA) to gain an advantage in mating”.

Line 79-81. "In Lepidoptera, the antennal lobe shows obvious sexual dimorphism. The male specific MGC is located at the entry of the antenna and exclusively processes pheromone signals."
While this statement is generally true, it is not "exclusively" true. There are examples within Lepidoptera where some regions of the MGC process no pheromone signals. See Trona et al., 2010 - Journal of Experimental Biology, and Namiki et al., 2008, Journal of Comparative Physiology A; both of these report on processing of non-pheromonal odorants by the MGC in respective species.
R: We accept and adjust this sentence as “The male-specific macroglomerular complex (MGC) locates at the entry of the antenna and mainly processes pheromone signals, in addition in some species e.g. Cydia pomonella and Bombyx mori, it also responds to plant volatiles (Trona et al., 2010; Namiki et al., 2008)” and cite both publications.

Line 90-92. "Moverover, ghost moths have undergone asymmetrical divergence of duplicated genes to deliver functional alterations in subsequent species, providing insights into the evolutionary process of Lepidoptera [21]."
It is not clear whether the correct reference is being cited here, or if it is indeed the correct reference, what the claim is referring to. The cited article does not mention ghost moths nor anything about asymmetric divergence of duplicated genes. So it is not clear how this statement is supported nor how the conclusion is reached.
R: We are sorry for this citation mistake. The right citation was used in revised version as “Holland, P. W., Marlétaz, F., Maeso, I., Dunwell, T. L. & Paps, J. New genes from old: asymmetric divergence of gene duplicates and the evolution of development. Philosophical Transactions of the Royal Society B: Biological Sciences 372, 20150480 (2017).”

Line 93 (and also on 102). "epidemic".
I don't think this is the correct word, I think it is meant to say "endemic"
R: We checked our manuscript and exchange “epidemic” to “endemic”.

Results.
Line 137-138. "This indicates that both male and female Himalaya ghost moths may keep pheromone reception with their shortened antennae".
Without actually testing the functional response profiles of neurons housed within the sensilla trichoidae of ghost moths, this is a speculative statement, and should not be presented in the results section, but instead in the discussion.
R: We move this sentence to the discussion part (L262-266). Please see “Interestingly, the presence of unique olfactory structures including enlarged glomeruli observed in both sexes and dominated long trichoid sensilla, mostly non-biased expressions of TxiaOr19 homologs, along with their non-feeding life traits in adulthood suggests that these ghost moths may employ a primitive pheromone sensing system to locate potential partners.”

Line 140-141. "compared with other Lepidopteran moths (in general 50 to 80 glomeruli) (Figure 1E).
Where does this data come from? In addition to Figure 1E here, it is needed to refer to the relevant supplementary data file as well.
R: We have added the glomerular counts in Data S1 and referred to this file in the text.

Line 149-150. "The cumulus which represents a major area involving pheromone reception"
A reference is needed for this statement. Furthermore, is it being suggested that his area involves pheromone processing in the ghost moth? Is anything known about the pheromones used in these species? This should be described in the appropriate section of the introduction.

Powered by Editorial Manager® and ProduXion Manager® from Aries Systems Corporation

	R: We deleted sentence of "involving pheromone reception" in results. So far, most researches about ghost moth mating or partner seeking were focus on behaviors (Kuenen et al., 1994; Allan and Wang, 2010), hence people speculate that female pheromones or male pheromone were used to attract partner in Hepialidae species. Additionally, only 3 species' male pheromones were identified including H. californicus (Kubo et al. 1985, Uchino et al. 1985), H. hecta (Schulz et al. 1990), and E. excrescens (Marukawa and Mori 2002). Hence, studies of pheromone sensing of Hepialidae species are very rare in the past. These background of ghost moth pheromone sensing was added to the manuscript. Please see "While most previous research has focused on mating behaviors and pheromone identifications in hepialids (Kuenen et al., 1994; Mallet 1984; Schulz et al., 1990; Chen et al., 2024 Journal of Economic Entomology; Allan and Wang, 2010; Kubo et al. 1985, Uchino et al. 1985; Marukawa and Mori, 2002), few studies have explored their potential pheromone-sensing neural architecture or annotated the odorant receptor family in Hepialidae species."<br>In this manuscript, we first provide evidence of male and female enlarged glomeruli (Figure 1). Considering these species showed non-feeding bio traits in their adulthoods and different sex roles, we believe that those enlarge glomeruli may function as pheromone processing in hepialids. We add more discussion as "Interestingly, the presence of unique olfactory structures including enlarged glomeruli observed in both sexes and dominated long trichoid sensilla, mostly non-biased expressions of TxiaOr19 homologs, along with their non-feeding life traits in adulthood suggests that these ghost moths may employ a primitive pheromone sensing system to locate potential partners."<br><br>Line 164. "A total of 23 TxiaORs were confirmed to be expressed from the genome and transcriptomes…"<br>Confirmed to be expressed where? In which tissues specifically?<br>R: The RT-PCR verification showed that 23 annotated ORs expressed in antennal cDNAs. The OR panel comprised with 35 annotations was obtained from genome and antennal transcriptome. The confirmed 23 ORs were used plus 34 TxiaOr19 tamdem and added up to 57 ORs as shown in the previous text. We agree that the sentence was confusing, so that we have revised this part to make it more clearly to readers as "A total of 23 TxiaOrs were confirmed to be expressed in the antennae of T. xiaojinensis via RT-PCR verification, out of annotations from genome and antennal transcriptome assembly". The original annotated ORs were listed in additional Data S2.<br><br>Line 168-170. "This array, homologous to TxiaOR19, contained 16 homologues (TxiatdORs) and 18 pseudogenes (TxiatdpORs), which maintained the largest tandem duplications reported in lepidopterans (Figure s5, data s2).<br>Figure s5 does not show an array of tandem duplications, it just shows all of the candidate genes' exon/intron structure lined up in separate rows, some of which seem to have similar patterns, but many that do not. The representations are inexplicably named "testNN" and it is not clear the relevance of these names. To make the intended point, It would be far more useful to highlight with different colors homologous exons, and show an additional panel that diagrams their relative positions in the genome on the respective chromosome. The focus on the OR19 tandem expansion of ORs is a central point in this report. So this claim needs to be properly visualized.<br>R: We have updated Figure S5 which included physical locations of TxiaOR19 homologs on chr14 and exon/intron structures for each homolog with homologous exons indicated by color. We also named all TxiaOR19 homologs.<br><br>Line 171-173. "The Maximum-Likelihood phylogeny analysis using 272 ORs showed that the TxiaOR19 array formed an earlier group where canonical type I PRs arose (Figure 2A, Data S3).<br>In looking at Figure 2A, it is not clear how well that statement is supported. The bootstrap support circle at the branchpoint between the OR19 clade and the canonical PR clade is rather small. It would be better to show the actual bootstrap support value number at this point instead of the circle representation in order to make it clearer to the reader.<br>R: We accept and updated the tree. The key bootstrap support values of the branch were indicated in the new Figure 2A. The relevant methods, results, and figure legends were revised.<br><br>Line 173-175. "This tree topology between the two clades was consistent when cross

Powered by Editorial Manager® and ProduXion Manager® from Aries Systems Corporation

checked with both Neighbor-Joining and Bayesian methods (Figure s6).
This is true, but those trees do not appear to clearly support such close relationships between the PR clade and the OR19 clade. How can these differences be reconciled? In Figure 2A the relationship between the two clades appears to be directly approximate, but in the Figure s6 trees, they do not.
R: We re-built the NJ and BY tree as the ML tree was updated, using the same batch of 387 ORs. Please see the updated Figure 2A and Figure S9. To make it clearer, we modified related sentence as "The Maximum-Likelihood phylogeny analysis using 387 ORs showed that the TxiaOr19 array joint an earlier group where canonical type I PRs arose (Figure 2A, Data S3). The earlier separation of canonical PRs and TxiaOR19 tandem was also observed when cross-checked with Neighbor-Joining and Bayes method (Figure S9)."

Line 176-178. "The TxiaOR19 array suggested an earlier emergence than male-biased TxiaOR7, as it could blast to ORs in locust, aphid, soldier flu, mosquito and flea, with homologues predicted by CLANS in mosquito and flea (Figure 2C, Figure s7).
It is not clear how Figures 2C and S7 supports the claim that any TxiaORs have any homology with ORs from mosquito and flea, and it is not clear from figure 2C what data supports this claim. It would have been better to include these ORs (from mosquito and flea) in figure 2A in order to provide better support for homologous relationships between ORs across distant insect orders. It is well known that there is little evidence for homology between ORs across distant insect orders, See Hansson and Stensmyr 2011 review in Neuron, Figure 3. Solid evidence would be needed to be shown to support the claim being made, and as currently presented, that evidence is lacking.
R: We revised this description, and updated the phylogeny tree with adding ORs from mosquito and flea by blasting using TxiaOR19 in the new Figure 2A. Please see "Differing from ORs found in evolutionarily later moths, the TxiaOR19 array could blast to ORs in locusts, aphids, soldier flies, mosquitoes, and fleas, with homologues predicted by CLAN [37] (Figure 2A and Figure S9). However, the male-biased TxiaOR7 failed to blast to any ORs from those earlier species (Figure S11, S12)."

Line 183-185. "suggesting possible evolutionary patterns similar to the duplicated zen family of orange swift moth, H. sylvina, which diverged to new functional gene families Shx"
This is a discussion point that should not be presented as results.
R: We delete this sentence in the results.

Line 185-186. "On the other hand, PRs could be traced back to a single LarmPR1 in the caddisfly."
What is this claim based on? It seems like it is being made from Figure 2D. However, it is not clear that anything shown in Figure 2D supports the claim. LarmPR1 does not seem to be connected or tracing to any other ORs in that figure.
R: We apologize for this unclearly claim and we updated Figure 2A and marked the caddisfly PR. The sentence was revised as "it showed that canonical PRs and a single LarmPR1 in the caddisfly formed a clade in the phylogeny".

Line 190-191. "Specifically, TxiaOR19 array arose from the LarmOR19-OR13a tandem (Figure 2E)."
What is the statistical support (Bootstrap values or Likelihood Ratio or other) for this claim? It does not seem to be apparent in the figure, so it is difficult to assess how well supported the phylogenetic relationships are.
R: We revised this sentence to "Specifically, TxiaOr19 array formed the same clade with LarmOr19-Or13a tandem". Please see Figure 2A.

Line 241-243. "which fits the predicted non-PR functioning of TxiaOR19 to a non-canonical female emission and the olfactory architectural observations."
What functional assays were done to show that TxiaOR19 does or does not function as a PR? Is this based on the molecular docking predictions shown in figure S8? If that is the case, it appears in that figure that the male-biased OR7 shows higher affinity to the female-specific emitted compound, Oleamide. This would seem to point to the potential for OR7 being a PR. How can the claim made here be reconciled with what is shown in Figure S8?
R: We agree that TxiaOR7 in the docking simulation could be a better hit for PRs, but we did not map this OR using canonical PRs along with the TxiaOR19. This TxiaOR7

may support the recent reports of the novel PR clade in lepidopteran species and worth further investigation. As we here in this work focus on the canonical PR clade, we revised this sentence as “Our results indicated that the sex roles of T. xiaojinensis adults were different from those of higher lepidopterans during mating allocation, which could relate to predicted unconventional TxiaOR19 tandem, the non-canonical female emission and the olfactory architectural observations.” And also in the discussion “One interesting result in our molecular docking predictions is that the male-biased OR7 shows a higher affinity to oleamide, suggesting that TxiaOR7 potentially serves as an ancestral PR of the novel PR clade in lepidopteran species. However, more experimental functional evidence needs to be provided for both TxiaOR19 and TxiaOR7 to support their evolutionary roles in lepidopteran species.”

Discussion

Line 251-253. "We have also discovered that both males and females of these moths possess a compact olfactory system with distinct structures involved in sexual recognition"

What is the basis for this claim? Which structures? It was not shown that any structures are involved in sexual recognition in the ghost moths examined in this report. I would suggest to be careful about making assumptions about homologous structures from higher moths while at the same time trying to argue in favor of great distinctions in the ancestral ghost moths compared to the higher moths. It is better to present data about the function of the structures of the ghost moth instead.

R: We accept and rephrase the sentence as “Interestingly, the presence of unique olfactory structures including enlarged glomeruli observed in both sexes and dominated long trichoid sensilla, mostly non-biased expressions of TxiaOr19 homologs, along with their non-feeding life traits in adulthood suggests that these ghost moths may employ a primitive pheromone sensing system to locate potential partners.”

Line 266-267. "OR19 duplications in these ghost moths do not appear to have enhanced functions"

What does this mean? Almost no evidence was presented demonstrating the function of these ORs.

R: Although we tried to deorphanize the TxiaOR19 by using molecular docking software, but it is still hard to show TxiaOR19’ functions. Hence, we toned down OR function in the manuscript and delete this sentence in here.

Line 294. "These cues also suggest the ancestral roles of the ghost moths in terms of their sex role systems."

It is not clear what is meant by this.

R: We rephrase this sentence as “Given their ecological traits, highly redundant genome, and a smaller number of ORs, we believe that these Himalayan ghost moths may retain pheromone sensing abilities and exhibit primitive sex roles within Lepidoptera. Further investigation into the function of ORs remains crucial for refining this perspective.”

Figures and Data

Figure S8. "or head space volatiles (blue)"

Are these blue traces both from females, or something else? It should be clarified in the legend.

R: We added the information in the legend.

Table S4. Concerning Cydia pomonella, Wan et al., 2019 reported 85 ORs, 65 GRs and 39 IRs (see page 4), so it is not clear what is being referred to here for 82 ORs, 15 IRs and 1 GR.

R: We apologize for this mistake. We then check all data in the Table S4 (the Table S2 in revision files) and we hope it is clearer now.

Reviewer #2: The manuscript entitled "A ghost moth olfactory prototype of the lepidopteran sex communication" is reviewed. This is a highly multidisciplinary work and difficult for me (and maybe to many other researchers) to review. Based on my expertise of insect genomics and molecular adaptation, I am providing my comments

mainly on the related sections (i.e. genome and transcriptome assembly, orthology analyses, and gene evolution). In general, this is publishable material from my point of view, with some revision required to improve the manuscript. Here I list two of my major concerns and some minor suggestions.
R: We appreciate your thorough examination and valuable suggestions. We have carefully incorporated all your recommendations into our manuscript and figures. In the relevant sections of the manuscript, we have highlighted the changes in yellow. We believe that these modifications have enhanced the clarity and readability of the manuscript compared to the previous version.

Main concerns:
1. For the sensilla distribution analysis (Figure 1D), I feel the result is potentially biased by the different ways of counting the sensilla trichoidae in the retrieved studies. For example, Gargi et al. were counting all the sensilla trichoidae on the entire antenna, while the authors possibly divided the number by number of flagellum segments to represent the "mean" number of sensilla trichoidae of each flagellum to make it comparable with the data matrix generated in the current study. But this is assuming that all the flagellum segments have similar number of sensilla trichoidae, which is unlikely to be true. Ideally, counting all the sensilla trichoidae is required to perform the statistic analyses. My suggestion is to not include this section in the manuscript as it is not necessary for authors to derive their conclusions.
R: We agree. The statistics for sensilla tricoidae was removed from the manuscript and we toned down related descriptions about sensilla.

2. The completeness assessment of the genome assembly of T. armoricanus is not available. So does the transcriptome of A. jianchuanensis. These are very crucial as if the assembly less complete, the subsequent analyses will be confounded. My suggestion is to run BUSCO on the genome and transcriptome assembles.
R: We added BUSCO results for both T. armoricanus genome and A. jianchuanensis transcriptome in the manuscript. Please see “BUSCO analysis indicates 90% of single-copy insect orthologs are complete. We also conducted BUSCO analysis towards the transcriptomes of A. jianchuanensis, and a completeness of 96.6% was observed”.

3. I think the figures are too complicated for most readers. Too packed figures make it unpleasant to read but the role of the figures should be helping readers to quickly get the key information. For example, Figure 1B, C, D, F, G, and H; Figure 2B, C, E, H, and G; Figure 3B and C, can be separated figures in SI.
R: We agree that visualization concerns and have followed suggestions. We, therefore, re-organized the figure panels, by moving Figure 1C, F, G, 2B, C, and 3B, C to the supplementary materials. The phylogenic tree was updated combining previous Figure 2A and D.

Minor suggestions:
1. Line 112 (results): I was expecting to see the assembly report of T. armoricanus as well, but it seems missing in the result sections.
R: We added assembly report of T. armoricanus in revised “Genome of T. armoricanus was sequenced on Illumina HiSq2000, harvesting 877.7 Gb clean data to construct scaffolds. The final assembly presented 3,168 Mb total length of the scaffolds, with N50 of 27.8 kb and 176.2 kb for contigs and scaffolds, respectively. BUSCO analysis indicates 90% of single-copy insect orthologs are complete. We also conducted BUSCO analysis towards the transcriptomes of A. jianchuanensis, and a completeness of 96.6% was observed. Genome and transcriptomes were subsequently employed in downstream analysis.”

2. Line 136: maybe address why H. cunea was selected for the comparison.
R: This comparison was removed according to Major comment 1. H. cunea is a signature species which presents significant sex dimorphism of the antennae among the tested species, so that we used this species as a calibrator to assess sensillar counts in T. xiaojinensis. Due to the counting method limitations raised in Major comment 1, we removed this part.

3. Line 165: not sure what the "57 annotations" means here.
R: We are sorry for this unclear description. The relevant part was revised and additional supplementary file was added. “A total of 23 TxiaOrs were confirmed to be

Powered by Editorial Manager® and ProduXion Manager® from Aries Systems Corporation

	expressed in the antennae of T. xiaojinensis via RT-PCR verification, out of annotations from genome and antennal transcriptome assembly" (Data S2 listed annotated TxiaORs for used in PCR verifications)<br><br>4. Line 167: what is LG14?<br>R: We revised LG14 to chr14.<br><br>5. Line359-360: please describe how the short read sequences were generated<br>R: We revised this part as "Meanwhile, short-reads library was constructed by Illumina platform with the same batch of T. xiaojinensis DNA, and 165 Gb raw data were generated. After filtering, the remaining clean reads with Q > 20 were used for minimap2 mapping onto the genome assembly which was later polished by NextPolish (https://github.com/Nextomics)."<br><br>6. Line 361-362: please provide more information about how the non-insect contigs were identified.<br>R: We added relevant information to this part. Please see "To remove the DNA pollutions from the other organisms, the polished genome was aligned against the NCBI nucleotide (NT) database, and the contigs which were aligned to the sequences from fungi, plants, or virus were removed."<br><br>7. Line384: not sure what it means by "second generation genome" since there is no previous version of genome assembly for the species.<br>R: We are sorry for the confusion description. The relevant part was revised as "Genome".<br><br>8. Line 406-407: What I know about orthofinder is to get the orthologs from protein sequences. Here it says "the orthologous genes of these 18 insect species were inferred from their genomes or transcriptomes" but those are DNA sequences not proteins.<br>R: We are sorry for the mistake. The orthofinder does refer to protein sequences. We revised this part.<br><br>9. Line425: I thought this should be protein-protein searching, but the queries are DNA, this is somewhat confusing me.<br>R: We apologize. We revised this part.<br><br>10. Line430: please provide more information about how the ORs were verified by PCRs.<br>R: The detailed methods were described in "Characterizations of Ors" section following this part. So that we removed this sentence in order not to draw any confusion.
**Additional Information:**	
**Question**	**Response**
Are you submitting this manuscript to a special series or article collection?	No
**Experimental design and statistics**<br><br>Full details of the experimental design and statistical methods used should be given in the Methods section, as detailed in our Minimum Standards Reporting Checklist. Information essential to interpreting the data presented should be made available in the figure legends.<br><br>Have you included all the information	Yes

requested in your manuscript?	
**Resources**<br><br>A description of all resources used, including antibodies, cell lines, animals and software tools, with enough information to allow them to be uniquely identified, should be included in the Methods section. Authors are strongly encouraged to cite Research Resource Identifiers (RRIDs) for antibodies, model organisms and tools, where possible.<br><br>Have you included the information requested as detailed in our Minimum Standards Reporting Checklist?	Yes
**Availability of data and materials**<br><br>All datasets and code on which the conclusions of the paper rely must be either included in your submission or deposited in publicly available repositories (where available and ethically appropriate), referencing such data using a unique identifier in the references and in the "Availability of Data and Materials" section of your manuscript.<br><br>Have you have met the above requirement as detailed in our Minimum Standards Reporting Checklist?	Yes

Research Article

A ghost moth olfactory prototype of the lepidopteran sex communication

Rui Tang[1#a], Cong Huang[23#], Jun Yang[4], Zhong-Chen Rao[1], Li Cao[1], Peng-Hua Bai[5], Xin-Cheng Zhao[6], Jun-Feng Dong[7], Xi-Zhong Yan[4], Fang-Hao Wan[23], Nan-Ji Jiang[8]*, Ri-Chou Han[1]*

1 Guangdong Key Laboratory of Animal Conservation and Resource Utilization, Guangdong Public Laboratory of Wild Animal Conservation and Utilization, Institute of Zoology, Guangdong Academy of Sciences, Guangzhou, China 510260

2 State Key Laboratory for Biology of Plant Diseases and Insect Pests, Institute of Plant Protection, Chinese Academy of Agricultural Sciences, Beijing, China 100193

3 Shenzhen Branch, Guangdong Laboratory for Lingnan Modern Agriculture, Genome Analysis Laboratory of the Ministry of Agriculture, Agricultural Genomics Institute at Shenzhen, Chinese Academy of Agricultural Sciences, Shenzhen, China 518120

4 College of Plant Protection, Shanxi Agricultural University, Taigu, Shanxi, China 030801

5 Institute of Plant Protection, Tianjin Academy of Agricultural Sciences, Tianjin, China 300384

6 Henan International Laboratory for Green Pest Control, College of Plant Protection, Henan Agricultural University, Zhengzhou, China 450046

7 Forestry College, Henan University of Science and Technology, Luoyang, China 471000

8 Department of Evolutionary Neuroethology, Max Planck Institute for Chemical Ecology, Hans-Knöll-Straße 8, Jena, Germany D-07745

*Correspondence: Nan-Ji Jiang, njiang@ice.mpg.de Hans-Knöll-Straße 8, Jena, Germany. Tel. +49 (0)3641 57-1456; Ri-Chou Han, hanrc@giz.gd.cn, 105 Xingang West Road, Haizhu District, Guangzhou. Tel. +86 020-84191089

# Equal contribution was claimed.

ORCID.ORG: a 0000-0002-9313-0802

Abstract

Sex role differentiation is a widespread phenomenon. Sex pheromones are often associated with sex roles and convey sex-specific information. In Lepidoptera, females release sex pheromones to attract males, which evolve sophisticated olfactory structures to relay pheromone signals. However, in some primitive moths, sex role differentiation becomes diverged. Here, we introduce the chromosome-level genome assembly from ancestral Himalaya ghost moths, revealing a unique olfactory evolution pattern and sex role parity among Lepidoptera. These olfactory structures of the ghost moths are characterized by a dense population of trichoid sensilla, both larger male and female antennal entry parts of brains, compared to the evolutionary later Lepidoptera. Furthermore, a unique tandem of 34 odorant receptor 19 homologs in *Thitarodes xiaojinensis* (*TxiaOr19*) has been identified, which presents overlapped motifs with pheromone receptors (PRs). Interestingly, the expanded *TxiaOr19* was predicted to have unconventional tuning patterns compared to canonical PRs, with non-sexual dimorphic olfactory neuropils discovered, which contributes to the observed equal sex roles in *Thitarodes* adults. Additionally, transposable element activity bursts have provided traceable loci landscapes where parallel diversifications occurred between *TxiaOr19* and *PRs*, indicating that the *Or19* homolog expansions were diversified to *PRs* during evolution and thus established the classic sex roles in higher moths. This study elucidates an olfactory prototype of intermediate sex communication from Himalaya ghost moths.

Keywords: Genome; Olfactory evolution; Neuroecology; Lepidoptera; Ghost moth; Sex role

Introduction

Sexual dimorphism is ubiquitous across the animal kingdom [1]. For most animals, mating by partner allocation is an indispensable process to ensure population continuity [2]. Sex roles often form under the pressure of sexual selection [3]. In general, the female exhibiting greater parental investment becomes a limiting resource for the less caring male so that the latter competes for accessing to the former [4]. In insects, sex pheromone becomes an effective investment for male to gain opportunities to mate with female successfully. One well studied example is that, in fruit fly *Drosophila melanogaster*, males typically release a specific pheromone called cis-11-vaccenyl acetate (cVA) to gain an advantage in mating [5]. However, sex roles appear to be reversed in moths [6]. Female moths invest in synthesizing and releasing sex pheromones to attract male moths, and males have evolved distinct structures for sensing pheromones [7,8]. Therefore, the study of pheromone and pheromone perception can expand our understanding of the evolution of sexual roles in animals.

One well-known animal lineage that relies on pheromone communication is Lepidoptera, comprising nearly 160,000 extant species and forming a key branch of insects [9]. Lepidoptera pheromones were well-studied in the last decade, and most can be classified into type 0, I, II, and III, according to their hydrocarbon chains, double-bond allocations, and terminal functional groups [10]. Among them, type I pheromones, consisting of straight-chain acetates, alcohols, or aldehydes with 10 to 18 carbon atoms, make up 75% of all known sex pheromones and are employed by most moth families [11]. Pheromones are detected by pheromone receptors (PRs)/odorant receptor co-receptors (ORco) on the dendrites of olfactory sensory neurons. Based on the pheromone types, the corresponding PR family can be classified into type 0, I, and II clades [12]. However, the recent discovery of *Lampronia capitella* OR6/Orco and *Spodoptera littoralis* OR5/ORco has revealed a

novel ‘PR clade’ that is distant from the type I PR clade [13,14]. This implies that the mechanisms underlying the evolutionary process of ORs for detecting pheromones in Lepidoptera need to be explored.

The neural architectures of pheromone perception appear to be conserved in moths [15]. A typical perception of type I pheromone is achieved through a label-lined olfactory coding pattern in higher moths, such as Noctuidae. Pheromones are tuned by olfactory sensory neurons housed in sensilla trichoidae on the antennae. After the PR/ORco complex has been activated by the corresponding pheromone, the potential signals are projected to the primary olfactory center, the antennal lobe [16]. In Lepidoptera, the antennal lobe shows obvious sexual dimorphism. The male-specific macroglomerular complex (MGC) locates at the entry of the antenna and mainly processes pheromone signals [17], in addition in some species e.g. *Cydia pomonella* and *Bombyx mori*, it also responds to plant volatiles [18,19]. The counterparts of the MGC in females are usually called the large female glomeruli (LFG) that process oviposition and host-choosing signals, but LFG glomeruli are not generally enlarged in size as the MGC [20,21].

The ghost moths (Hepialoidae: Hepialidae) from Exoporia are primitive Lepidoptera species and form an especially interesting lineage for studying the evolution of sex roles and pheromone communication [22]. Hepialids represent an early branch from the line leading to the heteroneuran Ditrysia, and the latter includes almost all the lepidopteran species which use typical PR-based olfaction for pheromones. Notably, the sex roles of Hepialidae species show diversity; for example, *Hepialus hecta* and *H. humuli* exhibit courtship behavior that is very different from the usual moth pattern, as males hover in groups to attract females [22]. Moreover, ghost moths have undergone asymmetrical divergence of duplicated genes to deliver functional alterations in subsequent species, providing insights into the evolutionary process of Lepidoptera [23]. *Thitarodes*, *Ahamus*,

and *Hepialus* ghost moths, as the hosts of *Ophiocordyceps sinensis* medicinal fungus, are endemic to the Qinghai-Tibet Plateau [24]. The isolated ecological habitat and prolonged life cycle of these so-called Himalaya ghost moths provide ideal opportunities for the retention of pheromone receptive characteristics from shared ancestors of Lepidoptera [25-27]. While most previous research has focused on mating behaviors and pheromone identifications in hepialids [22, 28-34], few studies have explored their potential pheromone-sensing neural architecture or annotated the odorant receptor family in Hepialidae species.

In this study, we presented the unique evolutionary position of olfaction in ghost moths, characterized by comparative neurology and phylogenomics. Our results demonstrated that the antennal lobes of both male and female of three Himalaya ghost moth species (*Ahamus jianchuanensis*, *Thitarodes armoricanus*, *T. xiaojinensis*) have an enlarged antennal entry part and lack obvious sexual dimorphism, when compared to evolutionary later Lepidopterans. Comparative genomics further revealed that the ghost moth *T. xiaojinensis* expanded a specific *Or19* tandem array instead of the classic type I PR clade. Behavioral tests indicated that the ghost moth *T. xiaojinensis* exhibits similar sex roles between males and females in courtship, possibly due to their pheromonal neural architectures without sexual dimorphism and the specific *Or19* tandem array. In summary, this study uncovers a mechanism for the occurrence of functional ORs such as PRs through asymmetric divergence in Lepidoptera.

Results

Chromosome-level genome assembly of Himalaya ghost moth

A *T. xiaojinensis* larva was sequenced using Nanopore long-read technology, resulting in 319.9 Gb of clean reads. The draft genome of 3.1 Gb, comprising 1,645 contigs with a contig N50

of 5.4 Mb, was assembled using NextDenovo, corrected with minimap2 and NextPolish, and refined by removing contaminants. Utilizing Hi-C interaction data, the primary assembly was divided into 31,434 contigs, and 31,391 contigs (99.86% in length) were anchored to 32 chromosomes (Figure S1). BUSCO analysis revealed 91.8% complete genes in the final chromosome-level genome assembly, which was subsequently employed in downstream analysis.

Genome of *T. armoricanus* was sequenced on Illumina HiSq2000, harvesting 877.7 Gb clean data to construct scaffolds. The final assembly presented 3,168 Mb total length of the scaffolds, with N50 of 27.8 kb and 176.2 kb for contigs and scaffolds, respectively. BUSCO analysis indicates 90% of single-copy insect orthologs are complete. We also conducted BUSCO analysis towards the transcriptomes of *A. jianchuanensis*, and a completeness of 96.6% was observed. Genome and transcriptomes were subsequently employed in downstream analysis.

Evolutionary position of ancient Himalaya ghost moths based on phylogenomics analysis

We carried out a phylogenomics analysis based on genomes and transcriptomes of three ghost moth species, together with 13 Lepidoptera and two outgroups (data source see Table S1). The separation of exoporian and ditrysian Lepidoptera occurred by the end of the Triassic Period, at around 205 million years ago. While the speciation of the *Thitarodes* moths soon followed *A. jianchuanensis*, at around 26 million years ago by the end of the Paleogene Period. Although *Ahamus* and *Thitarodes* represented an ancient moth lineage, the species within were diverged in parallel with higher moths (Figure 1A). We next asked what olfactory traits were maintained during the evolution of Himalaya ghost moths.

Nonsexual dimorphic shortened antennae and enlarged glomeruli of Himalaya ghost moths

The Himalaya ghost moths had no observable proboscis but possessed the antennae and labial palps intact (Figure 1B). Besides, we found that these moths presented the shortest antennae among 32 lepidopteran families [35] (Figure S2) and their antennae were dominated by sensilla trichoidae (Figure 1C, Figure S3).

The antennal lobe morphological atlas showed that three ghost moth species overall presented significantly less glomeruli (23 to 37), compared with other moths (45 to 80, Figure 1D, Data S1). Amongst 96 tested brains, the ordinary glomeruli arrangements in Himalaya ghost moths were distinguishable with those in the compared species. The families of Hepialidae, Pieridae, and Plutellidae had less intra-species variations in glomerular arrangements compared to the other four higher moth families (Figure S4). The MGC consisted of 2 to 3 glomeruli in Himalaya ghost moths, and identical areas were confirmed in all tested species. Female LFGs was distinguishable in earlier species including the Himalaya ghost moths and the diamondback moth *Plutella xylostella*, comparing with the later species (Figure 1D).

Volume proportions of the MGC and LFG glomeruli across tested species were compared. It showed that Himalaya ghost moths had both the largest MGCs and LFGs (Figure S5). The cumulus which represents a major area, occupied 23.8 ± 5.5% of the antennal lobes in *A. jianchuanensis*, 15.9 ± 2.0% in *T. armoricanus* and 19.5 ± 3.0% in *T. xiaojinensis*, respectively (Figure S5, Data S1). The other species had relatively smaller MGC glomeruli in volumes, e.g., on average 9.8 + 0.7% of the cumulus occupation for Noctuidae. As for females, LFG1 in three ghost moth species were significantly larger than those within higher moths (Figure S5, Data S1). We checked the MGCs of 16 species in terms of volumes and shapes by utilizing a principal component analysis test, and 73.8% fraction of explained variances was covered. Twelve of 16 lepidopteran species had a similar trend in MGC organizations, but the Himalaya ghost moths and *H. cunea* exhibited

separated patterns, and especially *T. xiaojinensis* was totally isolated from higher Lepidoptera (Figure S6). We wonder if these structural specificities may reflect the genomic backgrounds and receptor repertoires in the Himalaya ghost moths.

A unique large OR array on the ghost moth T. xiaojinensis *chromosome*

A total of 23 *TxiaOrs* were confirmed to be expressed in the antennae of *T. xiaojinensis* via RT-PCR verification, out of annotations from genome and antennal transcriptome assembly (Figure S7, Data S2). This number was higher than those of *A. jianchuanensis* (10) and *T. armoricanus* (16) (Table S2). Notably, a large array comprising 34 tandem duplications was mapped by higher moth PRs on chr14 of the chromosome-level assembly of *T. xiaojinensis*. This array, homologous to *TxiaOr19*, contained 16 homologs (*TxiatdOrs*) and 18 pseudogenes (*TxiatdpOrs*), which maintained the largest tandem duplications reported in lepidopterans (Figure S8, Data S2). The *TxiaOr19* array was located on the same chromosome with an upstream *TxiaOr18c*, which mapped to Noctuidae homologs [36]. The Maximum-Likelihood phylogeny analysis using 387 ORs showed that the *TxiaOr19* array joint an earlier group where canonical type I PRs arose (Figure 2A, Data S3). The earlier separation of canonical PRs and TxiaOR19 tandem was also observed when cross-checked with Neighbor-Joining and Bayes method (Figure S9). A female-biased expression pattern was observed in *TxiatdOr15* and *TxiatdOr25* of the 16 *TxiaOr19* homologs (Figure S10). Differing from ORs found in evolutionarily later moths, the TxiaOR19 array could blast to ORs in locusts, aphids, soldier flies, mosquitoes, and fleas, with homologues predicted by CLAN [37] (Figure 2A and Figure S9, S11). However, the male-biased TxiaOR7 failed to blast to any ORs from those earlier species (Figure S11, S12).

We investigated the evolution of the *TxiaOr19* array by mapping it to chromosomes of

caddisflies, primitive and higher moths, and cross-checking with canonical PR mapped regions (Figure 2B). The results indicated that tandem ORs were identified in linearized regions of almost all species, except for *C. flavipennella* and *B. mori*. Some of these linearized regions did not contain ORs that could be annotated using FGENESH [38]. Mapped ORs from linearization analysis and nr blast ORs by TxiaOR19 tandem were used in a Bayesian phylogenic analysis, and it showed that canonical PRs and a single LarmPR1 in the caddisfly formed a clade in the phylogeny (Figure 2A, Figure S13), and expansions of PR tandems were consistently observed within species after the Himalaya ghost moths, except for *C. flavipennella* (Figure 2B, Figure S13). Notably, regions containing *TxiaOr19* and PR had mixed tandem patterns in the early moths succeeding *T. xiaojinensis* but tended to be separated in later species (Figure 2B). Bayesian phylogenetic analysis showed that the homologs of *TxiaOr19* underwent significant diversification from their ancestors (Figure S13). Specifically, *TxiaOr19* array formed the same clade with *LarmOr19-Or13a* tandem (Figure 2A, Figure S13). The majority of PR-mapped ORs formed a single cluster that possibly diverged from LarmPR1, with a few remaining in the TxiaOR19 mapped phyla (Figure 2A, Figure S13).

LarmPR1 exhibited all three motifs of PR consensus regions [12], indicating the potential emergence of canonical PRs prior to the evolution of lepidopteran insects (Figure 2C). However, most PR-mapped ORs from non-Ditrysia primitive moths did not meet the requirements of canonical PR motifs (Figure 2C). The majority of TxiaOR19-mapped ORs had two motifs that overlapped with PRs, with some having three motifs but with shifted positions (Figure 2D). Notably, motifs 4-6 from TxiaOR19 homologs showed overlaps with motifs 2-3 from PR homologs, suggesting the existence of a common ancestor for TxiaOR19 and canonical PRs at an earlier evolutionary stage (Figure 2E). In all, the genomic backgrounds and receptor repertoires are

specific in *T. xiaojinensis*, which may confer to the structural specificities described above.

Transposable elements (TE) *involved in the evolution of ORs*

Tandem gene duplications and chromosome linearization patterns have been reported to be associated with TE activities [39]. To explore how a large and specific OR array was formed in the ghost moth *T. xiaojinensis*, we characterized the landscape of TEs in the genomes of above species. It showed that 2-3 TE burst events occurred in the Himalaya ghost moths. These bursts likely took place around the same time as the successive divergences of Exoporia and *Hepialus* (Figure 3A, Figure 1A). Caddisfly, primitive and modern Lepidoptera experienced more recent bursts of TE activity compared to Himalaya ghost moths (Figure 3A, Figure S14). Specifically, various TE arrangements were observed in previously identified OR loci. The TE arrangements of the *TxiaOr19* tandem were correlated with that of the *LarmOr13a* (Figure S15). Furthermore, the TE landscapes in the *TxiaOr18/19* homologs were found to be similar mostly with *Ors* from non-ditrysian species and later diversified in higher moths (Figure S15).

In conclusion, our findings suggest that both the TxiaOR19 tandem and PR clusters had already emerged in caddisflies. The OR19 lineage underwent expansion within the ancestral moth lineage, leading to the formation of a large duplicated tandem in *T. xiaojinensis*. Furthermore, linkages between OR19 and PRs were observed in species predating Ditrysia. In later higher moths, PRs became predominant, while the OR19 cluster contracted through asymmetric diversifications of their homologs, as supported by the similar TE arrangements (Figure 3B).

Equal sex roles of ghost moth T. xiaojinensis *adults*

The replacement of the TxiaOR19 duplications with canonical PRs suggests possible

functional drift of TxiaOR19. To confirm this assumption, we first analyzed the emissions of adult *T. xiaojinensis* using both solvent extraction and solid-phase microextraction (SPME) methods. We found that male and female adults were not distinguishable by tracing the volatile blends in abdomen tip extractions. However, SPME samples collected within the first 24 hours after female emergence exhibited a significant peak corresponding to oleamide (Figure S16A). We performed successive docking simulations using TxiaOR19 and four sex-biased TxiaORs against the identified major components. The results showed that TxiaOR19 had less binding affinity towards the panel of the ghost moth emissions, and its responding spectrum was relatively broad (Figure S16B), indicating that ghost moth *T. xiaojinensis* may show unconventional courtship behaviors comparing to higher moths.

To confirm this speculation, we tested adult pairs of *T. xiaojinensis* in a courtship arena (Figure 4A). Unlike the female calling behaviors of higher moths with wing beats and extruded pheromone gland, calling behavior of this ghost moth involved hovering with wing beats. Females fluttered with substantial wing beats, while males fluttered by small range vibrating-like wing beats (Figure 4B). Interestingly, male and female adults exhibited similar amounts of calling behaviors and tracing velocities (Figure 4B and C). Our results indicated that the sex roles of *T. xiaojinensis* adults differed from those of higher lepidopterans during mating allocation, which may be attributed to the predicted unconventional TxiaOR19 tandem, non-canonical female emission, and olfactory architectural observations.

Discussion

Himalaya ghost moths offer a basal model for the study of olfaction evolution in insects due to their unique olfactory system, limited distribution, and possession of the largest genomes in

Lepidoptera. Our study has successfully generated the first chromosome-level assembly for this lineage, providing valuable insights into the genetic characteristics of these ghost moths. We have discovered that the OR evolutionary pathway in ghost moth *T. xiaojinensis* parallels with that of modern moths, and have identified molecular traces that reveal the origins of the modern pheromone sensory system. Interestingly, the presence of unique olfactory structures including enlarged glomeruli observed in both sexes and dominated long trichoid sensilla, mostly non-biased expressions of *TxiaOr19* homologs, along with their non-feeding life traits in adulthood suggests that these ghost moths may employ a primitive pheromone sensing system to locate potential partners.

Why the lineage of Himalaya ghost moths keeps primitive may be due to their isolated habitats, uneven long life cycle for larvae (3-6 years in nature) and a brief adult stage (several days) [26,27], which greatly reduces the evolution speed of this lineage. They have developed a unique evolutionary strategy of focusing solely on reproduction in adulthood while forgiving foraging [40]. The redundancy of their giant genomes is an example of the basal genomic features possessed by the ghost moths [41]. Asymmetrically diverged duplications and frequent TE activity bursts have played a critical role in both PR formation and the emergence of other functional genes in Lepidoptera [23]. As a result, the expanded OR19 homologs in primitive species were later diversified to canonical PRs, establishing the advanced sex pheromone-based communication system. One interesting result in our molecular docking predictions is that the male-biased OR7 shows a higher affinity to oleamide, suggesting that TxiaOR7 potentially serves as an ancestral PR of the novel PR clade in lepidopteran species. However, more experimental functional evidence needs to be provided for both TxiaOR19 and TxiaOR7 to support their evolutionary roles in lepidopteran species.

Canonical PRs of ancient moth species were broadly characterized [13,43]. In this study, we show that the TxiaOR19 array and LarmPR1 share motifs that reflect some exons on the loci. These motifs were separated before the evolution of Lepidoptera. Considering that motif shifts accompany similar transposable element arrangements within the tested OR loci, it is likely that the first PR emerged from exonization, driven by TE activities. This effect has been commonly observed in other organisms [44]. On the other hand, the shared motif regions could be traced back to earlier dipteran species, which also had similar large OR duplications such as in *Bactrocera dorsalis* [45]. The disappearance of large tandem arrays in later lepidopterans suggests the separation of ancestral duplicated ORs. This can be supported by scattered chromosome linearization and increased DNA transposons during TE activity bursts. The duplicated ORs themselves could reflect rapid olfactory evolution for species adaptation [46]. Although TEs may not be determining factors for the functional emergence of ORs, as shown in the clonal raider ant *Ooceraea biroi* [47], we cannot exclude possible TE involvement in PR emergence due to the horizontally diverged TE landscapes among lepidopteran ORs.

Evolutionary later moths show larger interspecies variations and increased numbers of glomeruli in antennal lobes, suggesting potential positive selection in olfactory systems, along with their distribution in different ecological niches [48]. Enlarged MGCs in the butterfly *Pieris rapae* suggest that the sexual dimorphic sex pheromone recognition system is widely used by Lepidoptera [49]. However, the sexual dimorphic olfactory neuropils are not suitable for Himalaya ghost moths, as females have also retained the enlarged LFGs. We argue that female LFGs are more likely involved in mating allocation rather than egg-laying orientation since these ghost moths spray eggs in nature, unlike the majority of modern moth egg-laying behavior on the selected locations [40,50]. Therefore, they may not require a sophisticated olfactory system for precise assessment of

corresponding sites. This is also supported by our behavioral assays in the *T. xia* that both male and female were moving to find partners. Given their ecological traits, highly redundant genome, and a smaller number of ORs, we believe that these Himalayan ghost moths may retain pheromone sensing abilities and exhibit primitive sex roles within Lepidoptera. Further investigation into the function of ORs remains crucial for refining this perspective.

Himalaya ghost moths retain ancestral traits of olfactory system for equal sex roles, which may be attributed to the mechanisms of asymmetric divergence and redundant genome formation. The lack of sexual dimorphism in the antennal lobes and expansion of TxiaOR19 array other than canonical PRs also contribute to the non-biased sex role differentiation within this primitive lineage. Overall, these findings highlight the unique evolutionary features of Himalaya ghost moths and shed light on the mechanisms shaping olfactory systems in insects.

Materials and Methods

Insects

Newly emerged lepidopteran species from lab colonies were sexed and 3-5 d adults were used in all tests. *A. jianchuanensis*, *T. armoricanus*, *T. xiaojinensis*, *Agrotis ipsilon*, *S. frugiperda*, *Galleria mellonella*, and *P. rapae* were obtained from Institute of Zoology, Guangdong Academy of Sciences. *H. cunea* were obtained from Chinese Academy of Forestry. *Athetis dissimilis* were obtained from Henan University of Science and Technology. *Helicoverpa armigera* and *Helicoverpa assulta* were obtained from Henan Agricultural University. *Mythimna separata*, *C. pomonella*, and *S. litura* were obtained from Institute of Plant Protection, Chinese Academy of Agricultural Sciences. *S. exigua* were obtained from Qingdao Agricultural University. *P. xylostella*

were obtained from Shanxi Agricultural University. All lab colonies were regularly rejuvenated with natural populations.

Morphometric measurement

A total of 7 strains of Himalaya ghost moths were measured by the lengths of antennae and forewings. Three *T. xiaojinensis* strains were from lab colony mentioned above, and Xiaojin (N30.99, E102.27), Hongkou (N31.16, E103.84) field populations. The other four strains consisted of two *A. jianchuanensis* populations collected from Jiulong (N28.99, E101.51), Gongga (N29.56, E101.98), and two *T. armoricanus* populations from Yala (N30.11, E102.25), Kangding (N30.08, E101.97), respectively. Intact appendages were removed and embedded with glass slides before processed under an AXIO Imager microscope (Zeiss, Jena, Germany) equipped with an Axiocam 512 camera (Zeiss). A ZEN 2.3 software (Zeiss) was used to acquire scale bar labelled photographs of antennae and wings. Lengths of interest were manually assigned to the scale bar with ImageJ 1.53f51 (National Institute of Health, USA) and then recorded. A total 5 to 21 replicates were carried out for each strain, and means were used to develop olfactory indexes by the formula [antenna/wing]. Data of other species were referred to the previous publication [35].

Scanning electron microscopy

The antennae of 1-3 d adults were cut from base and fixed in 0.25% glutaraldehyde at 4 °C overnight. After three washes at room temperature with 0.1 M phosphate-buffered saline (PBS, pH 7.4), antennae were dehydrated through a laddered ethanol series (30, 50, 70, 80, 90, and 100%) and dried in a critical point drier (Bal-Tel CPD 030) before mounted on aluminum stubs. The mounted antennae were coated with gold spray (Bal-Tel SCD 005) and observed with SEM instrument (FEI Quanta 200).

Antennal lobe atlas

Lepidopteran brains were labeled according to the previous work [21]. Newly dissected intact brains were successively processed with 4% paraformaldehyde in 0.1 M PBS for fixation (24 h), pre-incubating with 5% normal goat serum in 0.1 M PBS containing 0.5% Triton X-100 (NGS-PBST) (0.5 h), incubating with 1% SYNORF1 (Developmental Studies Hybridoma Bank, University of Iowa) in 5% NGS-PBST (72 h), and incubating with Alexa Fluor 488 goat anti-mouse (Invitrogen, Eugene, OR, USA) at 1:500 with 1% NGS-PBST (48 h). After rinsed for six times in PBS and dehydrated with laddered ethanol series, brain samples were mounted with antifade mounting medium (Beyotime, Shanghai, China) in a perforated aluminum slide which was sandwiched by two glass coverslips. Three brains of each sex from each species were prepared for imaging.

All image stacks were acquired with a confocal laser scanning microscopy system with a 10-20x objective. Data for *A. jianchuanensis*, *T. armoricanus*, *T. xiaojinensis*, *G. mellonella*, and *A. ipsilon* were collected with FV3000 (Olympus, Tokyo, Japan). Data for *H. cunea*, *A. dissimilis*, *H. armigera*, *H. assulta*, *M. separata*, *S. litura*, and *P. xylostella* were collected with LSM 780 (Zeiss). Data for *S. frugiperda*, *P. rapae*, *C. pomonella*, and *S. exigua* were collected with A1 HD25 (Nikon, Tokyo, Japan). An argon laser at 488 nm was used to excite the Alexa Fluor. The resolution of the x-axis was 500 - 2,048 voxels and the section interval was set to 3 or 5 μm. Amira software (AMIRA 5.3, Visage Imaging, Fürth, Germany) was used as previously described to conduct segmentation, tissue statistics, and three dimensional reconstructions of the antennal lobes [21].

Genome and transcriptome sequencing

Genomic DNA of *T. xiaojinensis* larva was extracted for library establishment, and then sequenced with Nanopore PromethION platform (Oxford Nanopore Technology, Oxford, UK). After quality control, a total 319.9 Gb clean data was assembled by using NextDenovo

(https://github.com/Nextomics). Meanwhile, short-reads library was constructed by Illumina platform with the same batch of *T. xiaojinensis* DNA, and 165 Gb raw data were generated. After filtering, the remaining clean reads with Q > 20 were used for minimap2 mapping onto the genome assembly which was later polished by NextPolish (https://github.com/Nextomics). To remove the DNA pollutions from the other organisms, the polished genome was aligned against the NCBI nucleotide (NT) database, and the contigs which were aligned to the sequences from fungi, plants, or virus were removed.

To obtain a chromosome-level assembly, Hi-C scaffolding was further carried out with the same larval sample following reported protocols [51-53]. Specifically, samples were fixed using 2% formaldehyde to establish cross-links, followed by cell lysis and sample quality assessment through extraction. Chromatin digestion was carried out using a restriction endonuclease, with enzyme cleavage efficacy evaluated through sampling. Subsequent steps included biotin-14-dCTP (Invitrogen) labeling, blunt-end ligation, DNA purification, and Hi-C sample preparation. After passing quality control, Hi-C fragments underwent end-biotin removal, sonication, end repair, A-tailing, and adapter ligation to form ligated products. Subsequent PCR steps were amplified to generate library enriched products. Library amplification products were sampled for Hi-C fragment junction quality control, and the entire library preparation was sequenced using Illumina HiSeq with a PE150 sequencing strategy (NextOmics Biotech. lnc., Wuhan, China). The fastp v.0.12.6 (RRID:SCR_016962) with default parameters was used to filter the raw sequences, resulting in high-quality clean reads. The sequenced Reads1 and Reads2 were separately aligned to the assembled genome sequence using bowtie2 v.2.3.2 (end-to-end alignment mode, parameters: --very-sensitive -L 30) (RRID:SCR_016368) to obtain the alignment information. For the unmapped reads after alignment, we searched for reads containing ligation junction sites, trimmed them, and

performed alignment again. Finally, the alignment results were combined, and the proportion of Unique Mapped Paired-end Reads was calculated. The LACHESIS software (RRID:SCR_017644) was used to cluster the Contig sequences of the draft assembly into chromosome groups using agglomerative hierarchical clustering. The final genome was further assessed with BUSCO [54] for completeness.

Genome of *T. armoricanus* was obtained from the DNA of a fourth instar larva without gut. A total of 23 different insert size libraries were constructed and 67 lanes were sequenced on Illumina HiSq2000, harvesting 1,344.5 Gb raw data and 877.7 Gb filtered data. The genome was assembled using SOAPdenovo (v2.04) [55] and SSPACE (v2.0) [56] software. We used all 549.3 Gb (180.4×) clean data of short insert size libraries to construct contigs and all 877.7 Gb (266.4×) clean data to construct scaffolds. 283.4 Gb (86.0×) data of large insert size libraries was used again to construct scaffolds by using SSPACE. Then all clean data of short insert size libraries was used to fill the gaps. TrimDup3 (Rabbit2.6) (https://github.com/gigascience/rabbit-genome-assembler) was used to remove the large redundant sequences. RNA-seq data from 14 different developmental stages of *T. armoricanus* was assembled by Trinity v2.4.0 [57] and was mapped to the assembled genome sequence using BLAT (v. 34) [58], to check the coverage rate. The results showed that 96.8% of the sequences could be mapped to the assembly.

Respective antennae, heads, and labial palps from *A. jianchuanensis* and *T. xiaojinensis* were collected in liquid nitrogen and sequenced with Illumina according to the manufactural instructions. The transcriptomes were assembled by Trinity v2.4.0 [57] with default parameters.

Phylogenetic analysis and estimation of divergence time

To reconstruct the phylogenetic tree of 16 lepidopteran insect species with two outgroups of *Tribolium castaneum* and *D. melanogaster*. Except for three species of *A. dissimilis*, *T.*

xiaojinensis, *A. jianchuanensis*, we first downloaded the genome annotations or raw data of transcriptomes for other 15 species from NCBI (Table S1). The transcripts were assembled by Trinity v2.4.0 [57] with default parameters. Subsequently, the orthologs of these 18 insect species were inferred from their genomic or transcriptomic protein annotations by using OrthoFinder [59] with the default parameters. Single-copy orthologues from each species were selected for phylogenetic reconstruction. The protein sequences of each orthologue were independently aligned with MAFFT v7.407 [60], and the aligned results were trimmed by trimAl [61] to remove low-quality regions with the parameter "-automated1", the trimmed sequences were concatenated into a single super sequence. RAxML [62] was then used with the VT + F model, which is inferred by ProtTest v3.4.2 [63], to estimate a maximum likelihood tree starting with 1000 bootstraps followed by likelihood optimization.

We used the r8s (V1.7.1) [64] to estimate the divergence time. The phylogenetic tree constructed by RAxML [62] was used as an input tree. A smoothing parameter of 3 was selected, which was estimated by the cross-validation approach (with parameters "cvstart=0, cvinc=1, cvnum=18"). The calibration points were: 1) the most recent common ancestor of the clade including *T. castaneum* and *P. xylostella*, constrained to be 337 Mya (million years ago); 2) the most recent common ancestor of the clade including *D. melanogaster* and *C. pomonella*, constrained to be 318 Mya; and 3) the most recent common ancestor of the clade including *P. rapae* and *S. litura*, constrained to be 125 Mya [42].

Annotation of Or *gene family*

The protein sequences of lepidopteran insect ORs were collected from NCBI. These protein sequences were then used as queries in iterative TBLASTN searches with parameter "-evalue 1e-5" against the assembly of the three ghost moth species to find candidate *Or* genes. A local

command line HMMER (version 3.1b2) [65] search was conducted for these candidate ORs against the Pfam-A database (http://pfam.xfam.org) to find the 7tm_6 (PF02949) or 7tm_4 (PF13853) HMM profile for ORs. FGENESH 2.6 [38] prediction of potential genes was done for contigs of interests. Data from other species were collected according to the reported works (Table S1).

Characterizations of Ors

CDS cloning verifications were carried out targeting on annotated *TxiaOrs* using adult antennal cDNA. Gene-specific primers were designed (Table S3) and PCRs were done on a Veriti 96-well thermal cycler (Applied Biosystems, MA, USA) using High Fidelity (HiFi) PCR SuperMix (Trans, Beijing, China). Products were processed with 1% agarose (BBI, Shanghai, China) on a PowerPac electrophoresis system (Bio-Rad, CA, USA) and visualized with a GelDoc-It TS3315 imaging system (UVP, CA, USA). Multiple bands such as for *TxiaOr18* were separately collected and purified with a gel extraction kit (GenStar, Beijing, China) before Sanger sequencing (Sangon Biotech, Shanghai, China). Later analysis was based on the longest sequenced *TxiaOrs* for each locus. *Or* expressions were showed as autoscaled heatmaps indicating the FPKM (Fragments Per Kilobase of transcript per Million mapped reads) which were calculated by RSEM [66] from head, antenna, and labial palp transcriptomes of adult ghost moths.

Phylogenetic analysis of 387 ORs were carried out with the above mentioned protocol using MAFFT [60], trimAl [61], and IQ-TREE [67] using 'Auto' option for model, with 1000 ultrafast [68] bootstraps, as well as the Shimodaira-Hasegawa-like approximate likelihood-ratio test [69]. Verifications were done to the tree topology with MEGA X [70] and MrBayes 3.2.6 [71] to establish the NJ tree based on Dayhoff model and BY tree based on Blosum62 model, respectively. Homologs of TxiaOR19 array were predicted by CLANS [37] using the blastx results against the NCBI nr database. For chromosome linearization tests, local tblastn was applied to map the selected

ORs towards chromosomes of each species (Table S1) and results were visualized as circos plots by using TBtools v1.113 [72]. Evolution of mapped ORs were inferred using MrBayes 3.2.6 [71] under JTT+F+G4 model (2 parallel runs, 200,000 generations), in which the initial 25% of sampled data were discarded as burn-in. The final average standard deviation of split frequencies was 0.069772. Protein motifs were predicted with MEME Suite v5.5.2 [73].

Annotation of repeats and transposable element families

For transposable element analysis, we first performed the *de novo* predictions for each species by RepeatModeler version open-1.0.11 (https://github.com/Dfam-consortium/RepeatModeler) to generate a specific library. Then we annotated the genome assembly by RepeatMasker version open-4.0.7 with the "ncbi" search algorithm. Annotated transposable element sequences were manually verified and classified with Dfam [74]. The calcDivergenceFromAlign.pl and createRepeatLandscape.pl scripts in the RepeatMasker package were used to calculate the Kimura divergence values and plot the repeat landscape, respectively. Estimations for transposable element burst times were based on the recently reported substitution rate of 6.19 x 10^{-10} per site per generation in arthropods [75].

Chemical analysis

Hexane extraction method was adopted from our previous works on moth pheromone identifications [76]. Abdomen tips of calling adult *T. xiaojinensis* were cut with dissection scissors and immediately put in 20 μl hexane (HPLC purity, Kermel Chemical Reagent Co., Tianjin, China) which kept at 4 ^{o}C for 1 d prior to the test. Head space SPME method was adopted from our previous works on body surface volatile emissions of insects [77]. Newly emerged male or female adults were kept in a mesh cage in separated rearing chambers for sampling. A 50/30 μm DVB/CAR/PDMS stableflex fiber (Supelco, Bellefonte, PA, USA) was penetrated into the cage

for sampling at 10 ºC for 24 h. The volatile blends sampled were either injected for 1 µl or subjected to an Agilent 7890B GC - 5977 MSD coupled system equipped with a HP-5MS column (0.25 µm x 30 m x 0.250 mm) (Agilent, Palo Alto, CA, USA). A 60 min oven temperature program was used following: 40 ºC for 2 min, 40 ºC to 150 ºC at 5 ºC/min, 150 ºC for 2 min, 150 ºC to 200 ºC at 10 ºC/min, 200 ºC for 5 min, 200 ºC to 230 ºC at 5 ºC/min, and 230 ºC for 18 min. Raw data were analyzed with MSD ChemStation (G1701FA F. 01. 03. 2357) by searching against a NIST 17 MS library (Agilent). A total 40 individuals were tested for SPME from two stratified groups. Each hexane extraction sample included 20 individuals and at least 3 replicates were done towards each sex.

Docking simulation

TxiaOR19 and the other four sex biased OR sequences of *T. xiaojinensis* were predicted by AlphaFold2 [78] for their tertiary structures. The 3D structures of 18 ligands were downloaded from PubChem [79]. The Molecular Operating Environment software (MOE, Chemical Computing Group ULC, Montreal, Canada) was used to dock the ligands with ORs. Briefly, ORs were prepared using MOE QuickPrep and ligands were energy minimized with the MOE Energy Minimize prior to the simulation. Triangle Matcher algorithm was selected for placement and 30 top-scoring placement poses were selected by the London dG empirical scoring function, while the rigid receptor was selected for refinement and top-scoring poses were selected by the GBVI/WSA dG empirical scoring function. The binding free energy of respective OR-ligand was estimated by using S Score function and later used for establishment of colour coded map.

Courtship arena

The assays were carried out using 1 d emerged naïve moths at peak mating hours 18 - 20 pm during sunset. One randomly chosen pair of *T. xiaojinensis* adults was placed in a paper funnel and

recorded for 1 h. A total 20 pairs were tested and recorded for calling and tracing behaviors. Recorded footages were processed through the idTracker [80] pipeline to obtain the velocities of moths showing as per pixel distances per min. Fluttering behaviors were observed by manually checking each video file.

Statistics and data processing

Comparison of means was done by using either unpaired *t* test or GLM followed by multiple comparisons according to treatment sizes (SPSS 22.0.0.0, IBM Corp., Armonk, NY, USA). Simple linear regression and data plotting were done using Prism 5.01 (GraphPad software, San Diego, CA, USA). Multivariate tests were carried out with MetaboAnalyst 5.0 [81] server which integrates R statistics (https://www.r-project.org). All error bars indicate standard errors of the means otherwise indicated in the figure legends.

Author contributions

Rui Tang: Conceptualization, Methodology, Data curation, Formal analysis, Investigation, Visualization, Writing-original draft, Writing-review & editing. **Cong Huang**: Methodology, Data curation, Formal analysis, Visualization, Writing-review & editing. **Jun Yang**: Data curation, Writing-review & editing. **Zhong-Chen Rao**: Methodology, Formal analysis, Writing-review & editing. **Li Cao**: Data curation, Writing-review & editing. **Peng-Hua Bai**: Data curation, Writing-review & editing. **Xin-Cheng Zhao**: Methodology, Data curation, Writing-review & editing. **Jun-Feng Dong**: Data curation, Writing-review & editing. **Xi-Zhong Yan**: Data curation, Writing-review & editing. **Fang-Hao Wan**: Formal analysis, Writing-review & editing. **Nan-Ji Jiang**: Conceptualization, Methodology, Data curation, Investigation, Writing-original draft, Writing-

review & editing. **Ri-Chou Han**: Conceptualization, Investigation, Supervision, Writing-review & editing.

Declaration of Interest

The authors declare no competing interests.

Acknowledgments

We thank Dr. Zhang Bin and Dr. Meng Xiang for supporting on insect materials. We thank Min-Jun Huang for providing technical support on bioinformatics. We thank Zhongkai University of Agriculture and Engineering, South China Normal University, and Institute of Zoology, CAS for sharing the research platforms to assist confocal imaging. We thank B.F.A. Yorda for the development of insect schematics.

Funding information

The current research was funded by the Major Science and Technology Project of Qinghai Province (No. 2021-SF-A4-1), National Key Research and Development Program of China (2023YFC2606900), GDAS Special Project of Science and Technology Development (2022GDASZH-2022010106), and Guangdong Basic and Applied Basic Research Foundation (No. 2020A1515011366).

Data availability

All data are available in the main text or the supplementary materials. The whole genome sequence data of *T. xiaojinensis* reported in this paper have been deposited in NCBI (Bioproject: PRJNA1006505).

References

1. Shine, R. Ecological causes for the evolution of sexual dimorphism: a review of the evidence. The Quarterly Review of Biology 64, 419-461 (1989).
2. Ritchie, M. G. Sexual selection and speciation. Annual Review of Ecology, Evolution, and Systematics 38, 79-102 (2007).
3. Andersson, M. & Iwasa, Y. Sexual selection. Trends in Ecology & Evolution 11, 53-58 (1996).
4. Kokko, H. & Jennions, M. D. Parental investment, sexual selection and sex ratios. Journal of Evolutionary Biology 21, 919-948 (2008).
5. Datta, S. R., Vasconcelos, M. L., Ruta, V., Luo, S., Wong, A., Demir, E. et al. The *Drosophila* pheromone cVA activates a sexually dimorphic neural circuit. Nature 452, 473-477 (2008).
6. Allen, C. E., Zwaan, B. J. & Brakefield, P. M. Evolution of sexual dimorphism in the Lepidoptera. Annual Review of Entomology 56, 445-464 (2011).
7. Butenandt, v. A. Uber den sexsual-lockstoff des seidenspinners *Bombyx mori*. Reindarstellung und konstitution. Z. Naturforschg, b 14, 283 (1959).
8. Sakurai, T., Namiki, S. & Kanzaki, R. Molecular and neural mechanisms of sex pheromone reception and processing in the silkmoth *Bombyx mori*. Frontiers in Physiology 5, 125 (2014).
9. Stork, N. E. How many species of insects and other terrestrial arthropods are there on Earth? Annual Review of Entomology 63, 31-45 (2018).
10. Löfstedt, C., Wahlberg, N. & Millar, J. Evolutionary patterns of pheromone diversity in Lepidoptera. Pheromone communication in moths: evolution, behavior and application, 43-82 (University of California Press 2016).

11. Ando, T., Inomata, S. I. & Yamamoto, M. Lepidopteran sex pheromones. The chemistry of pheromones and other semiochemicals I, 51-96 (Springer, 2004).

12. Zhang, D. D. & Löfstedt, C. Moth pheromone receptors: gene sequences, function, and evolution. Frontiers in Ecology and Evolution 3, 105 (2015).

13. Yuvaraj, J. K., Andersson, M. N., Corcoran, J. A., Anderbrant, O. & Löfstedt, C. Functional characterization of odorant receptors from *Lampronia capitella* suggests a non-ditrysian origin of the lepidopteran pheromone receptor clade. Insect Biochemistry and Molecular Biology 100, 39-47 (2018).

14. Bastin-Héline, L., De Fouchier, A., Cao, S., Koutroumpa, F., Caballero-Vidal, G., Robakiewicz, S. et al. A novel lineage of candidate pheromone receptors for sex communication in moths. eLife 8, e49826 (2019).

15. Hildebrand, J. G. & Shepherd, G. M. Mechanisms of olfactory discrimination: converging evidence for common principles across phyla. Annual Review of Neuroscience 20, 595-631 (1997).

16. Zhang, J., Walker, W. B. & Wang, G. Pheromone reception in moths: from molecules to behaviors. Progress in Molecular Biology and Translational Science 130, 109-128 (2015).

17. Matsumoto, S. & Hildebrand, J. G. Olfactory interneurons in the moth *Manduca sexta*: Response characteristics and morphology of central neurons in the antennal lobes. Proceedings of the Royal Society of London. Series B. Biological Sciences 213, 249-277 (1981).

18. Trona, F., Anfora, G., Bengtsson, M., Witzgall, P., & Ignell, R. Coding and interaction of sex pheromone and plant volatile signals in the antennal lobe of the codling moth *Cydia pomonella*. Journal of Experimental Biology, 213(24), 4291-4303 (2010).

19. Namiki, S., Iwabuchi, S., & Kanzaki, R. Representation of a mixture of pheromone and host plant odor by antennal lobe projection neurons of the silkmoth *Bombyx mori*. Journal of Comparative Physiology A, 194, 501-515 (2008).

20. Rössler, W., Tolbert, L. P. & Hildebrand, J. G. Early formation of sexually dimorphic glomeruli in the developing olfactory lobe of the brain of the moth *Manduca sexta*. Journal of Comparative Neurology 396, 415-428 (1998).

21. Zhao, X. C., Ma, B. W., Berg, B. G., Xie, G. Y., Tang, Q. B. & Guo, X. R. A global-wide search for sexual dimorphism of glomeruli in the antennal lobe of female and male *Helicoverpa armigera*. Scientific Reports 6, 1-9 (2016).

22. Mallet, J. Sex roles in the ghost moth *Hepialus humuli* (L.) and a review of mating in the Hepialidae (Lepidoptera). Zoological Journal of the Linnean Society 80, 67-82 (1984).

23. Holland, P. W., Marlétaz, F., Maeso, I., Dunwell, T. L. & Paps, J. New genes from old: asymmetric divergence of gene duplicates and the evolution of development. Philosophical Transactions of the Royal Society B: Biological Sciences 372, 20150480 (2017).

24. Han, R., Wu, H., Tao, H., Qiu, X., Liu, G., Rao, Z. et al. Research on Chinese cordyceps during the past 70 years in China. Chinese Journal of Applied Entomology 56, 849-883 (2019).

25. Wang, Z. & Pierce, N. E. Fine‐scale genome‐wide signature of Pleistocene glaciation in *Thitarodes* moths (Lepidoptera: Hepialidae), host of *Ophiocordyceps* fungus in the Hengduan Mountains. Molecular Ecology 32, 2695-2714 (2023).

26. Wu, H., Cao, L., He, M., Han, R. & De Clercq, P. Interspecific hybridization and complete mitochondrial genome analysis of two ghost moth species. Insects 12, 1046 (2021).

27. Tao, Z., Cao, L., Zhang, Y., Ye, Y. & Han, R. Laboratory rearing of *Thitarodes armoricanus* and *Thitarodes jianchuanensis* (Lepidoptera: Hepialidae), hosts of the Chinese medicinal fungus *Ophiocordyceps sinensis* (Hypocreales: Ophiocordycipitaceae). Journal of Economic Entomology 109, 176-181 (2016).

28. Kuenen, L. P. S., Wagner, D. L., Wallner, W. E. & Cardé, R. T. Female sex pheromone in *Korscheltellus gracilis* (Grote) (Lepidoptera: Hepialidae). The Canadian Entomologist, 126(1), 31-41 (1994).

29. Schulz, S., Francke, W., König, W. A., Schurig, V., Mori, K., Kittmann, R. et al. Male pheromone of swift moth, *Hepialus hecta* L.(Lepidoptera: Hepialidae). Journal of Chemical Ecology, 16, 3511-3521 (1990).

30. Chen, X., Su, X., Qiu, Z., Xu, Y., Yang, Z. & Hu, P. Courtship and mating behavior of *Endoclita signifer* (Hepialidae: Lepidoptera) and the male sex pheromones in hairbrushes. Journal of Economic Entomology, 117(1), 218-229 (2024).

31. Allan, R. A. & Wang, Q. Mating behaviour, and evidence for a female - released sex pheromone, in *Wiseana copularis* (Meyrick) (Lepidoptera: Hepialidae). New Zealand Journal of Zoology, 28(3), 257-262 (2001).
32. Kubo, I., Matsumoto, T., Wagner, D. L. & Shoolery, J. N. Isolation and structure of hepialone; principal component from male sex scales of *Hepialus californicus* (Lepidoptera). Tetrahedron Letters, 26(5), 563-566 (1985).
33. Uchino, K., Yamagiwa, Y., Kamikawa, T. & Kubo, I. Synthesis of hepialone; principal component from male sex scales of *Hepialus californicus* (Lepidoptera). Tetrahedron Letters, 26(10), 1319-1320 (1985).
34. Marukawa, K. & Mori, K. Synthesis of (1R, 3S, 5S)-1, 3, 8-trimethyl-2, 9-dioxabicyclo [3.3. 1] non-7-ene, the male pheromone of a Hepialid Moth, *Endoclita excrescens*, and its enantiomer. European Journal of Organic Chemistry, 2002(23), 3974-3978 (2002).
35. Symonds, M. R., Johnson, T. L. & Elgar, M. A. Pheromone production, male abundance, body size, and the evolution of elaborate antennae in moths. Ecology and Evolution 2, 227-246 (2012).
36. Brigaud, I., Montagné, N., Monsempes, C., François, M. C. & Jacquin - Joly, E. Identification of an atypical insect olfactory receptor subtype highly conserved within noctuids. The FEBS Journal 276, 6537-6547 (2009).
37. Frickey, T. & Lupas, A. CLANS: a Java application for visualizing protein families based on pairwise similarity. Bioinformatics 20, 3702-3704 (2004).
38. Solovyev, V., Kosarev, P., Seledsov, I. & Vorobyev, D. Automatic annotation of eukaryotic genes, pseudogenes and promoters. Genome Biology 7, 1-12 (2006).
39. Krasileva, K. V. The role of transposable elements and DNA damage repair mechanisms in gene duplications and gene fusions in plant genomes. Current Opinion in Plant Biology 48, 18-25 (2019).
40. Nielsen, E. S., Robinson, G. S. & Wagner, D. L. Ghost-moths of the world: a global inventory and bibliography of the Exoporia (Mnesarchaeoidea and Hepialoidea) (Lepidoptera). Journal of Natural History 34, 823-878 (2000).
41. Cheng, R. L., Yu, Y. X., Liu, L. X., Zhang, C. X. & Fang, C. X. A draft genome of the ghost moth, *Thitarodes* (Hepialus) sp., a medicinal caterpillar fungus. Insect Science 23, 326-329 (2016).

42. Wan, F., Yin, C., Tang, R., Chen, M., Wu, Q., Huang, C. et al. A chromosome-level genome assembly of *Cydia pomonella* provides insights into chemical ecology and insecticide resistance. Nature Communications 10, 1-14 (2019).

43. Yuvaraj, J. K., Corcoran, J. A., Andersson, M. N., Newcomb, R. D., Anderbrant, O. & Löfstedt, C. Characterization of odorant receptors from a non-ditrysian moth, *Eriocrania semipurpurella* sheds light on the origin of sex pheromone receptors in Lepidoptera. Molecular Biology and Evolution 34, 2733-2746 (2017).

44. Sela, N., Kim, E. & Ast, G. The role of transposable elements in the evolution of non-mammalian vertebrates and invertebrates. Genome Biology 11, 1-13 (2010).

45. Wang, Y., Fang, G., Xu, P., Gao, B., Liu, X., Qi, X. et al. Behavioral and genomic divergence between a generalist and a specialist fly. Cell Reports 41, 111654 (2022).

46. Nei, M., Niimura, Y. & Nozawa, M. The evolution of animal chemosensory receptor gene repertoires: roles of chance and necessity. Nature Reviews Genetics 9, 951-963 (2008).

47. McKenzie, S. K. & Kronauer, D. J. The genomic architecture and molecular evolution of ant odorant receptors. Genome Research 28, 1757-1765 (2018).

48. Engsontia, P., Sangket, U., Chotigeat, W. & Satasook, C. Molecular evolution of the odorant and gustatory receptor genes in lepidopteran insects: implications for their adaptation and speciation. Journal of Molecular Evolution 79, 21-39 (2014).

49. Montgomery, S. H. & Ott, S. R. Brain composition in *Godyris zavaleta*, a diurnal butterfly, reflects an increased reliance on olfactory information. Journal of Comparative Neurology 523, 869-891 (2015).

50. Renwick, J. & Chew, F. Oviposition behavior in Lepidoptera. Annual Review of Entomology 39, 377-400 (1994).

51. Shi, J., Ma, X., Zhang, J., Zhou, Y., Liu, M., Huang, L. et al. Chromosome conformation capture resolved near complete genome assembly of broomcorn millet. Nature Communications 10, 464 (2019).

52. Servant, N., Varoquaux, N., Lajoie, B.R., Viara, E., Chen, C.-J., Vert, J.-P. et al. HiC-Pro: an optimized and flexible pipeline for Hi-C data processing. Genome Biology 16, 1-11 (2015).

53. Burton, J.N., Adey, A., Patwardhan, R.P., Qiu, R., Kitzman, J.O. & Shendure, J. Chromosome-scale scaffolding of de novo genome assemblies based on chromatin interactions. Nature Biotechnology 31, 1119-1125 (2013).
54. Simão, F.A., Waterhouse, R.M., Ioannidis, P., Kriventseva, E.V. & Zdobnov, E.M. BUSCO: assessing genome assembly and annotation completeness with single-copy orthologs. Bioinformatics 31, 3210-3212 (2015).
55. Luo, R., Liu, B., Xie, Y., Li, Z., Huang, W., Yuan, J. et al. SOAPdenovo2: an empirically improved memory-efficient short-read de novo assembler. Gigascience 1(1), 2047-217X (2012).
56. Boetzer, M., Henkel, C. V., Jansen, H. J., Butler, D. & Pirovano, W. Scaffolding pre-assembled contigs using SSPACE. Bioinformatics 27, 578-579 (2011).
57. Haas, B. J., Papanicolaou, A., Yassour, M., Grabherr, M., Blood, P. D., Bowden, J. et al. *De novo* transcript sequence reconstruction from RNA-seq using the Trinity platform for reference generation and analysis. Nature Protocols 8, 1494-1512 (2013).
58. Kent, W. J. BLAT - the BLAST-like alignment tool. Genome Research 12, 656-664 (2002).
59. Emms, D. M. & Kelly, S. OrthoFinder: phylogenetic orthology inference for comparative genomics. Genome Biology 20, 1-14 (2019).
60. Katoh, K. & Standley, D. M. MAFFT multiple sequence alignment software version 7: improvements in performance and usability. Molecular Biology and Evolution 30, 772-780 (2013).
61. Capella-Gutiérrez, S., Silla-Martínez, J. M. & Gabaldón, T. trimAl: a tool for automated alignment trimming in large-scale phylogenetic analyses. Bioinformatics 25, 1972-1973 (2009).
62. Stamatakis, A. RAxML version 8: a tool for phylogenetic analysis and post-analysis of large phylogenies. Bioinformatics 30, 1312-1313 (2014).
63. Darriba, D., Taboada, G. L., Doallo, R. & Posada, D. ProtTest 3: fast selection of best-fit models of protein evolution. Bioinformatics 27, 1164-1165 (2011).
64. Sanderson, M. J. r8s: inferring absolute rates of molecular evolution and divergence times in the absence of a molecular clock. Bioinformatics 19, 301-302 (2003).

65. Eddy, S. R. Accelerated profile HMM searches. PLoS Computational Biology 7, e1002195 (2011).
66. Li, B. & Dewey, C. N. RSEM: accurate transcript quantification from RNA-Seq data with or without a reference genome. BMC Bioinformatics 12, 1-16 (2011).
67. Nguyen, L. T., Schmidt, H. A., von Haeseler, A. & Minh, B. Q. IQ-TREE: a fast and effective stochastic algorithm for estimating maximum-likelihood phylogenies. Molecular Biology and Evolution 32, 268-274 (2015).
68. Minh, B. Q., Nguyen, M. A. & von Haeseler, A. Ultrafast approximation for phylogenetic bootstrap. Molecular Biology and Evolution 30, 1188-1195 (2013).
69. Guindon, S., Dufayard, J. F., Lefort, V., Anisimova, M., Hordijk, W. & Gascuel, O. New algorithms and methods to estimate maximum-likelihood phylogenies: assessing the performance of PhyML 3.0. Systematic Biology 59, 307-321 (2010).
70. Kumar, S., Stecher, G., Li, M., Knyaz, C. & Tamura, K. MEGA X: molecular evolutionary genetics analysis across computing platforms. Molecular Biology and Evolution 35, 1547 (2018).
71. Ronquist, F., Teslenko, M., Van Der Mark, P., Ayres, D. L., Darling, A., Höhna, S. et al. MrBayes 3.2: efficient Bayesian phylogenetic inference and model choice across a large model space. Systematic Biology 61, 539-542 (2012).
72. Chen, C., Chen, H., Zhang, Y., Thomas, H. R., Frank, M. H., He, Y. et al. TBtools: an integrative toolkit developed for interactive analyses of big biological data. Molecular Plant 13, 1194-1202 (2020).
73. Bailey, T. L., Boden, M., Buske, F. A., Frith, M., Grant, C. E., Clementi, L. et al. MEME SUITE: tools for motif discovery and searching. Nucleic Acids Research 37, W202-W208 (2009).
74. Hubley, R., Finn, R. D., Clements, J., Eddy, S. R., Jones, T. A., Bao, W. et al. The Dfam database of repetitive DNA families. Nucleic Acids Research 44, D81-D89 (2016).
75. Shao, C., Sun, S., Liu, K., Wang, J., Li, S., Liu, Q. et al. The enormous repetitive *Antarctic krill* genome reveals environmental adaptations and population insights. Cell 186, 1279-1294 (2023).

76. Jiang, N. J., Tang, R., Wu, H., Xu, M., Ning, C., Huang, L. Q. et al. Dissecting sex pheromone communication of *Mythimna separata* (Walker) in North China from receptor molecules and antennal lobes to behavior. Insect Biochemistry and Molecular Biology 111, 103176 (2019).

77. Liu, J., Zhang, R., Tang, R., Zhang, Y., Guo, R., Xu, G. et al. The role of honey bee derived aliphatic esters in the host-finding behavior of *Varroa destructor*. Insects 14, 24 (2022).

78. Jumper, J., Evans, R., Pritzel, A., Green, T., Figurnov, M., Ronneberger, O. et al. Highly accurate protein structure prediction with AlphaFold. Nature 596, 583-589 (2021).

79. Kim, S., Chen, J., Cheng, T., Gindulyte, A., He, J., He, S. et al. PubChem 2019 update: improved access to chemical data. Nucleic Acids Research 47, D1102-D1109 (2019).

80. Pérez-Escudero, A., Vicente-Page, J., Hinz, R. C., Arganda, S. & De Polavieja, G. G. idTracker: tracking individuals in a group by automatic identification of unmarked animals. Nature Methods 11, 743-748 (2014).

81. Pang, Z., Chong, J., Zhou, G., de Lima Morais, D. A., Chang, L., Barrette, M. et al. MetaboAnalyst 5.0: narrowing the gap between raw spectra and functional insights. Nucleic Acids Research 49, W388-W396 (2021).

Figure legends

Figure 1. Phylogenomics and olfactory morphology of the ghost moths comparing with other species in Lepidoptera. **(A)** Dated evolutionary tree of Lepidoptera relationships. Two of the non-lepidopteran species were placed on outgroup branches including *D. melanogaster* and *T. castaneum*. The tree was inferred through a maximum-likelihood analysis of 634,106 amino acid sites from 1,547 strict single-copy genes employing VT + F model and 1000 bootstrap replicates. Branch lengths were optimized and node ages estimated using the penalized likelihood (PL) methods with truncated Newton (TN) algorithm in r8s [56]. Scale bar is in millions of years. Data resources were listed in Table S1. **(B)** Adult head development of Hepialidae *T. xiaojinensis* comparing to moth *S. frugiperda* and butterfly *P. rapae*. Orange arrow indicates the labial palp. Blue arrow indicates the proboscis which lacks in the ghost moth. **(C)** Antennal sensilla morphology of selected Lepidoptera by scanning electron microscope. **(D)** Glomerular counts of tested Lepidoptera observed by confocal laser scanning microscopy system. Red coded bars indicate predicted male MGCs, and green bars indicate female LFGs. Numbers indicate standard errors of means. Lower case letters indicate significant differences of glomerular counts among species (GLM and Tukey HSD, male: $F_{15, 32} = 48.2$, $P < 0.0001$, female: $F_{15, 32} = 30.9$, $P < 0.0001$).

Figure 2. Evolution of sex pheromone-related odorant receptors among *T. xiaojinensis*, caddisfly, and other Lepidoptera. **(A)** Rooted Maximum Likelihood (ML) tree of 387 selected lepidopteran ORs which included reported ORs of moths, mapped ORs in the linearization analysis in (B) by TxiaOR19 tandem, and ORs obtained from pre-lepidopteran species by blasting with TxiaOR19 tandem against nr database (Data S4). The evolutionary distances were computed using

the 'Auto' option in IQ-TREE [67] with ultrafast [68] 1000 bootstraps and the Shimodaira-Hasegawa-like approximate likelihood-ratio test [69]. Tested species included *A. jianchuanensis* (Ajia), *T. armoricanus* (Tarm), *T. xiaojinensis* (Txia), *P. xylostella* (Pxyl), *C. pomonella* (Cpom), *B. mori* (Bmor), *S. exigua* (Sexi), *S. litura* (Slitu), *S. littoralis* (Slit), *Heliothis virescens* (Hvir), *H. armigera* (Harm), *H. assulta* (Hass), *Ectropis grisescens* (Egri), *Operophtera brumata* (Obru), *Agrotis segetum* (Aseg), *Eriocrania semipurpurella* (Esem), and *Lampronia capitella* (Lcap), *Limnephilus marmoratus* (Larm), *Micropterix aruncella* (Maru), *Incurvaria masculella* (Imas), *Nematopogon swammerdamellus* (Nswa), *Coleophora flavipennella* (Cfla), *Manduca sexta* (Msex), *A. ipsilon* (Aips), *Athalia rosae* (Aros), *Hhermetia illucens* (Hill), *Culex quinquefasciatus* (Cqui), *Aphis gossypii* (Agos), *Schistocerca americana* (Same), and *S. cancellata* (Scan). Tree topology was cross-checked with Neighbor-Joining and Bayes approaches (Figure S9). Red arrows indicate key bootstrap values related to type I PRs and TxiaOR19 tandem. Blue arrows indicate ancestral ORs from caddisflies related to type I PRs and TxiaOR19 tandem. **(B)** Linearization of TxiaOR18c-TxiaOR19 tandem proteins with chromosomes from selected species showed by circos plot. Reported chromosome assemblies (chr) from *L. marmoratus*, *M. aruncella*, *I. masculella*, *N. swammerdamellus*, *C. flavipennella*, *C. pomonella*, *M. sexta*, *B. mori*, and *A. ipsilon* were used (Table S1). ORs mapped by TxiaOR18c or TxiaOR19 tandem were colored in blue, and those PR mapped were colored in red. **(C)** Motif identification towards PR mapped ORs from caddisfly and moths. **(D)** Motif identification towards TxiaOR18c/19 tandem mapped ORs from caddisfly and moths. **(E)** Distribution of motifs identified in (C) and (D), showing overlaps from PR and TxiaOR18c/19 clades.

Figure 3. Genome and OR evolution reflected by landscapes of transposable elements (TEs). (A) Detailed TE landscapes of the ghost moths, caddisfly, and white-barred gold. Times of TE burst events were estimated according to CpG adjusted Kimura substitution levels and a reported arthropod substitution rate of 6.19 x 10^{-10} per site per generation [75]. **(B)** Overview of asymmetric divergence of duplicated pheromone-related ORs from caddisfly to higher Lepidoptera. The TxiaOR18c/19 duplications predominate in caddisfly and primitive moths, but they were replaced by functional PR duplications in higher moths during evolution.

Figure 4. Resulted dual attraction in sex communications of the ghost moth adults. **(A)** Schematic shows set-up of the courtship arena of *T. xiaojinensis* adults. **(B)** Comparison of calling rates which were reflected by fluttering behaviors in both sexes (Binary test against even distribution, $P = 0.33$). **(C)** Left shows representative behavioral traces of male and female adults tracked by idTracker [80]. Right shows comparison of distance per min between male and female *T. xiaojinensis* adults (Mann Whitney test, $U = 137$, $P = 0.8055$).

Click here to access/download;Figure;Figure01.tif

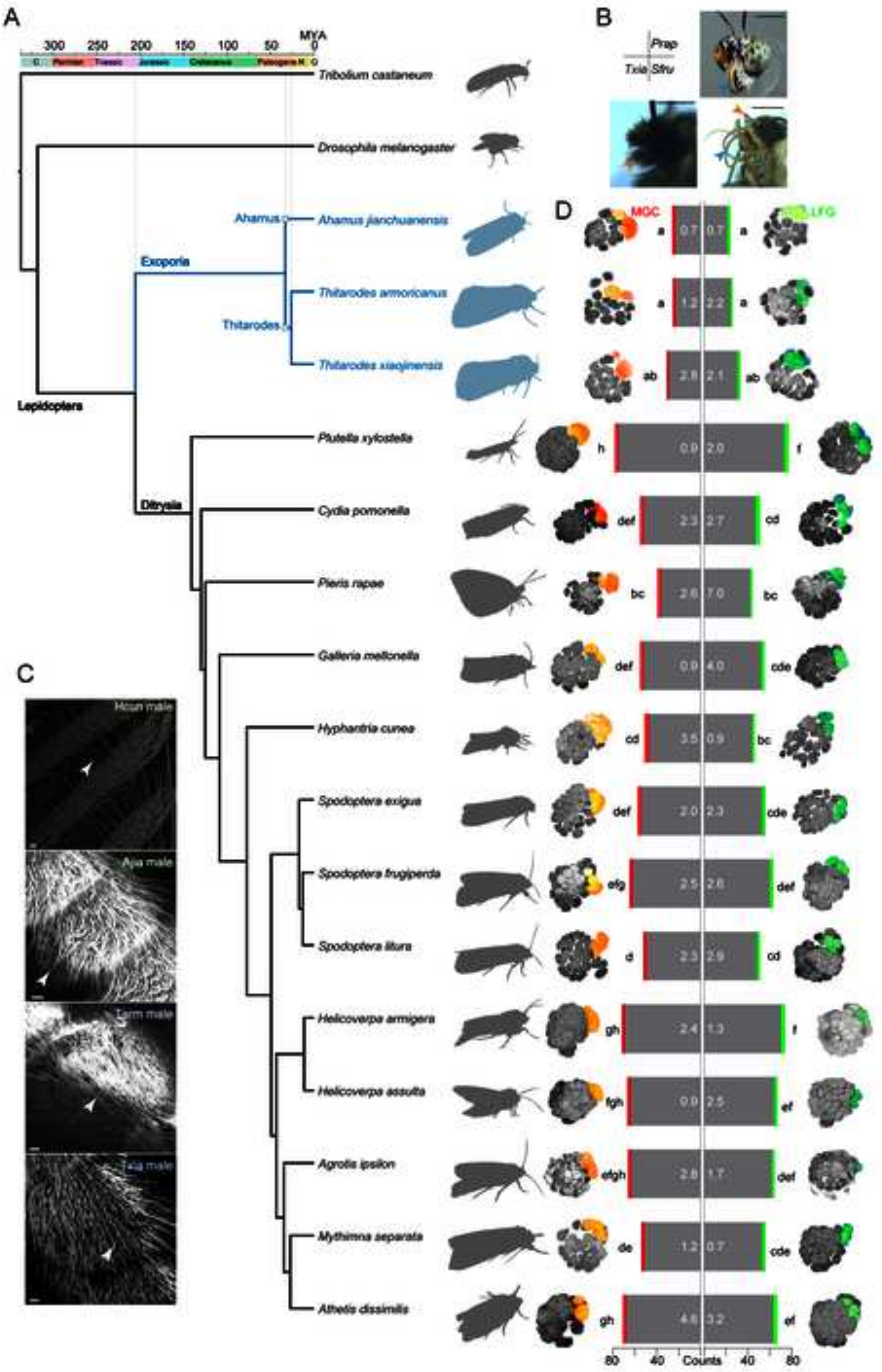

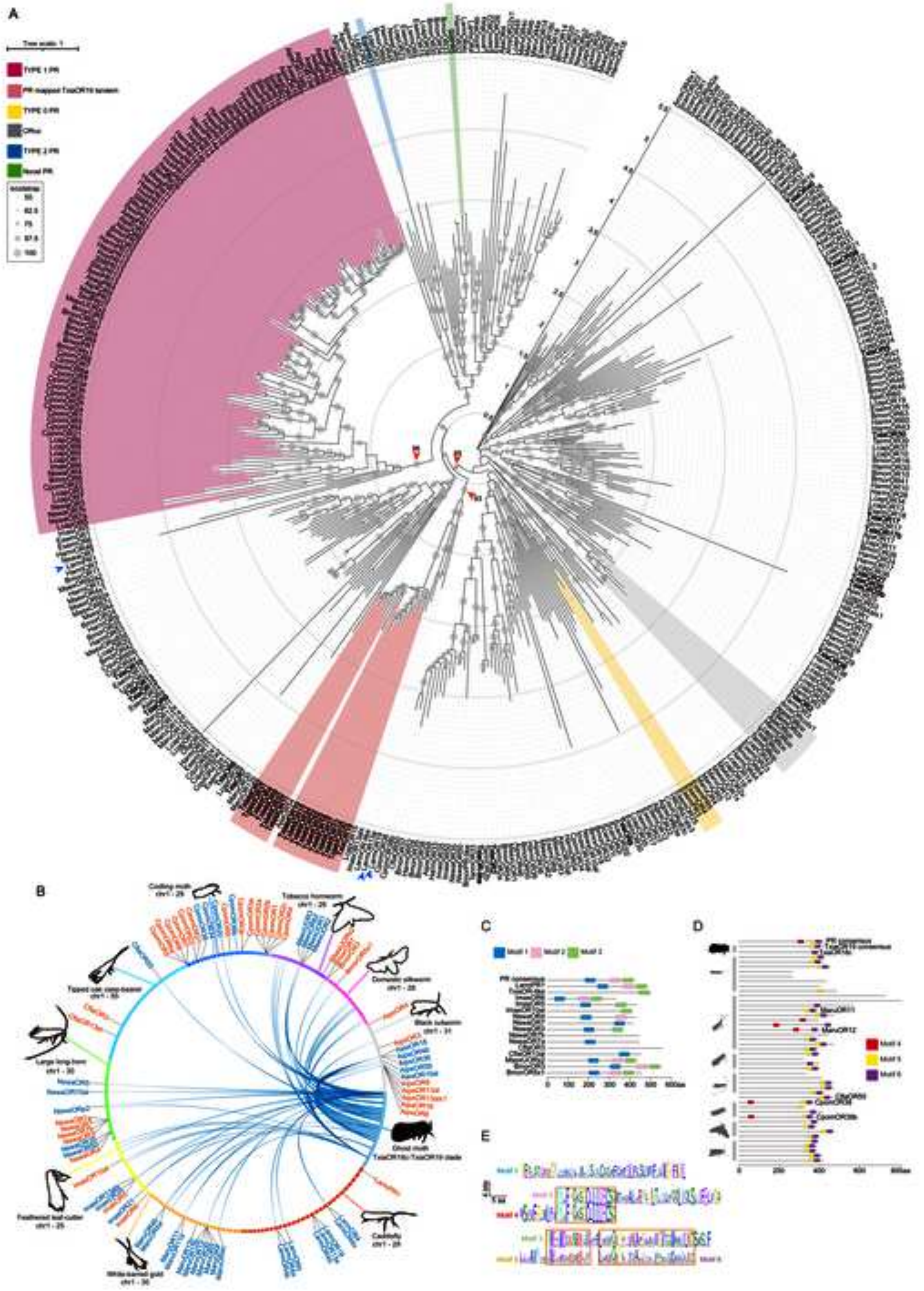
A
TYPE 1 PR
TYPE 0 PR
TYPE 2 PR
Novel PR
B
C
D
E

Figure3

Click here to access/download;Figure;Figure03.tif

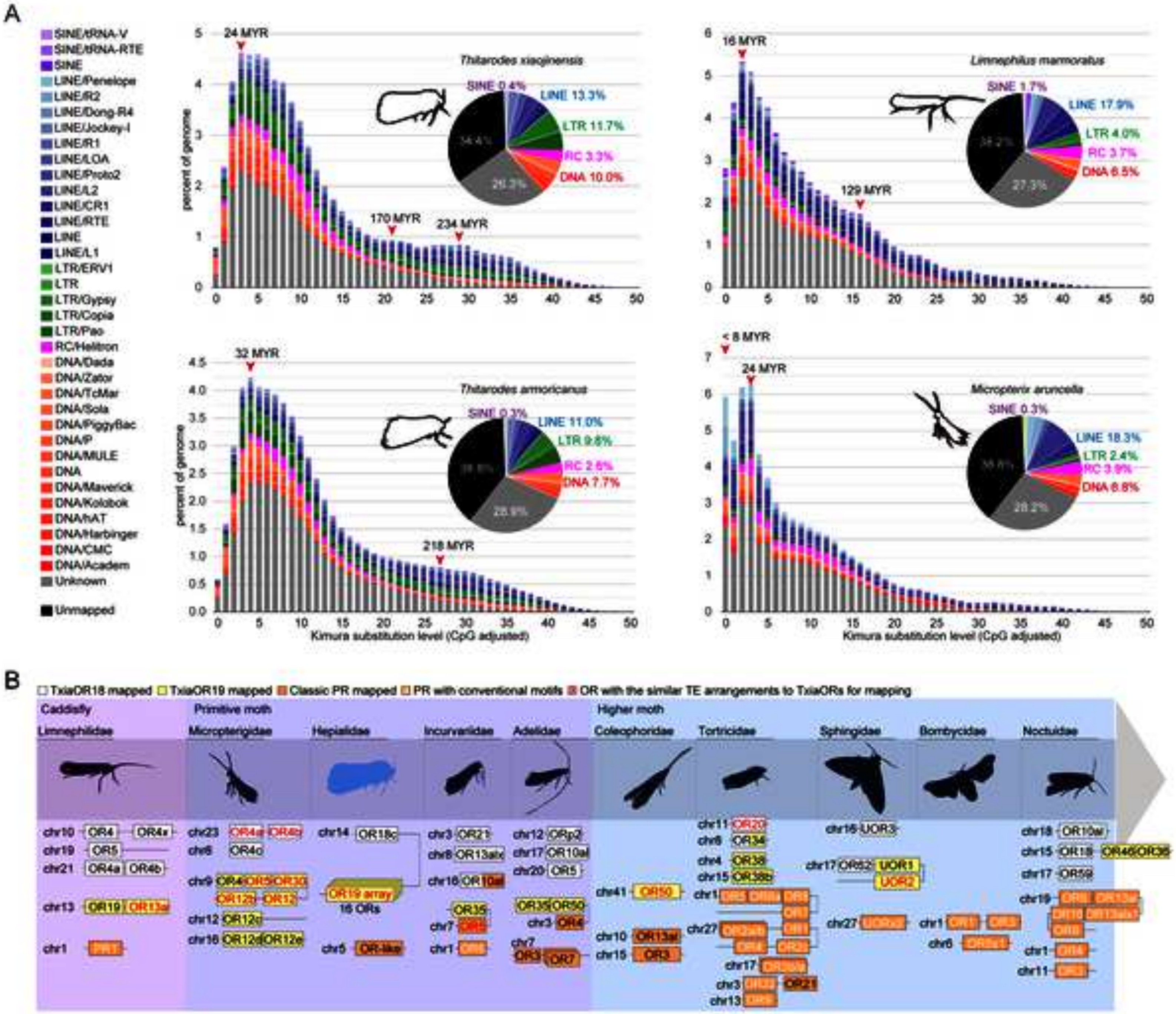

Figure4

Click here to access/download;Figure;Figure04.tif

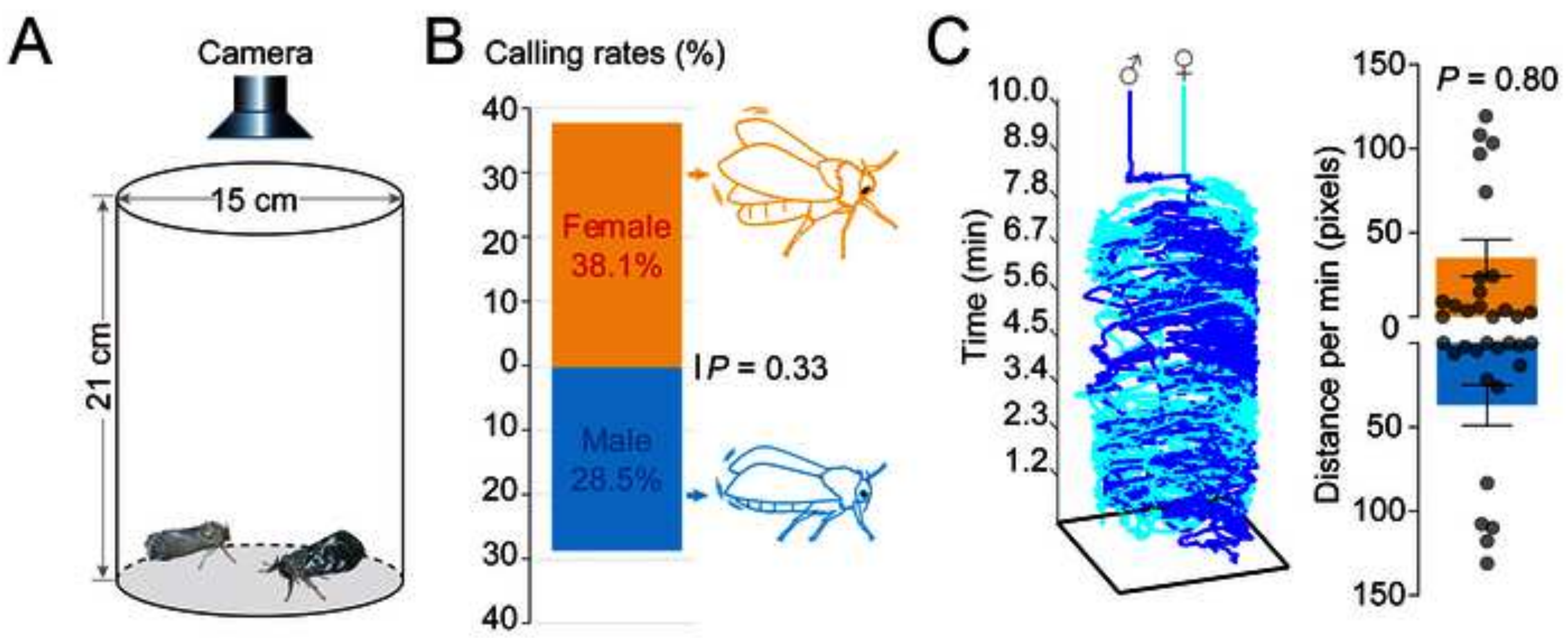

Supplementary Material main file

Click here to access/download

Supplementary Material

Supplementary materials_clear.docx

Data S1

Click here to access/download
Supplementary Material
Data S1.xlsx

Data S2

Click here to access/download
Supplementary Material
Data S2.fasta

Data S3

Click here to access/download
Supplementary Material
Data S3.rar

Data S4

Click here to access/download
Supplementary Material
Data S4.fas

Letter of responses R1 Click here to access/download;Personal Cover;Letter_of_response_clear.docx

Dear editor,

Thank you for considering our manuscript publish on the journal *Gigascience*. We have modified our manuscript very carefully based on both reviewers' suggestions, and it takes some time. All changes we have marked by highlight in the text. More details please see the manuscript or the response letter.

Best regards,

Ri-Chou Han

Reviewer reports:

Reviewer #1: The authors report on the early evolution of the olfactory system within Lepidoptera insects, framed upon the phenomena of intra-specific pheromone communication and subsequent reproductive behaviors, within three species of the primitive Exoporia lineage of Lepidoptera, compared to several other species of the more derived Ditrysian lineage of Lepidoptera. Within evolutionary contexts, olfactory organ morphology, olfactory neuroanatomy, olfactory receptor genes, transposon dynamics within genomic loci, and courtship behaviors are examined. The report is thus broad in scope, with an immense amount of data collected and examined, the methodology appears to be sound and are clearly described, however there are substantial concerns about the presentation of the results, in that the evidence to support claims being made is sometimes not clear or transparent, other times the conclusions drawn do not seem to be supported by the results presented. If these issues can be rectified, the report would provide a substantial contribution to our understanding of the early evolution of olfactory systems and olfactory-based sexual communication within Lepidoptera.

R: We thank you for detailed examinations, suggestions and pointing out our weakness in this manuscript. We, therefore, fully followed those suggestions and check our manuscript carefully, especially for conclusions. We have modified related parts of this manuscript accordingly (also marked by highlight in the text or see in below response letter). We feel that those suggestions do improve our manuscript considerably.

Specific comments are given for each section.

Abstract.

Line 37. "modern Lepidoptera".

What is a modern Lepidoptera? Is it meant to say derived (as compared to ancestral?)

R: We exchange the term "modern Lepidoptera" to the "evolutionary later Lepidoptera". And we hope the term is clearer than before.

Line 40. "Interestingly, the expanded TxiaOR19 does not function as canonical PRs". There were no functional assays conducted on any ORs in this study. This statement should be revised.

R: We revised this sentence as "Interestingly, the expanded TxiaOr19 was predicted to have unconventional tuning patterns compared to canonical PRs".

Introduction.

Line 55-56. "In most insects, such as the vinegar fly, Drosophila melanogaster, males usually release the specific pheromone cVA to gain an advantage in recruiting females." This sentence seems to be written incorrectly. As it is now, it is claiming that in most insects, males release cVA. Is this what is intended? Or is it meant to say that most in most insects, males release the specific (sex?) pheromone, such as is seen with Drosophila, with male release of cVA.

R: We apologize for this mistake. We rephrased these sentences as "In insects, sex pheromone becomes an effective investment for male to gain opportunities to mate with female successfully. One well studied example is that, in fruit fly *Drosophila melanogaster*, males typically release a specific pheromone called cis-11-vaccenyl acetate (cVA) to gain an advantage in mating".

Line 79-81. "In Lepidoptera, the antennal lobe shows obvious sexual dimorphism. The male specific MGC is located at the entry of the antenna and exclusively processes

pheromone signals."

While this statement is generally true, it is not "exclusively" true. There are examples within Lepidoptera where some regions of the MGC process no pheromone signals. See Trona et al., 2010 - Journal of Experimental Biology, and Namiki et al., 2008, Journal of Comparative Physiology A; both of these report on processing of non-pheromonal odorants by the MGC in respective species.

R: We accept and adjust this sentence as "The male-specific macroglomerular complex (MGC) locates at the entry of the antenna and mainly processes pheromone signals, in addition in some species e.g. *Cydia pomonella* and *Bombyx mori*, it also responds to plant volatiles (Trona et al., 2010; Namiki et al., 2008)" and cite both publications.

Line 90-92. "Moverover, ghost moths have undergone asymmetrical divergence of duplicated genes to deliver functional alterations in subsequent species, providing insights into the evolutionary process of Lepidoptera [21]."

It is not clear whether the correct reference is being cited here, or if it is indeed the correct reference, what the claim is referring to. The cited article does not mention ghost moths nor anything about asymmetric divergence of duplicated genes. So it is not clear how this statement is supported nor how the conclusion is reached.

R: We are sorry for this citation mistake. The right citation was used in revised version as "Holland, P. W., Marlétaz, F., Maeso, I., Dunwell, T. L. & Paps, J. New genes from old: asymmetric divergence of gene duplicates and the evolution of development. Philosophical Transactions of the Royal Society B: Biological Sciences 372, 20150480 (2017)."

Line 93 (and also on 102). "epidemic".

I don't think this is the correct word, I think it is meant to say "endemic"

R: We checked our manuscript and exchange "epidemic" to "endemic".

Results.

Line 137-138. "This indicates that both male and female Himalaya ghost moths may

keep pheromone reception with their shortened antennae".

Without actually testing the functional response profiles of neurons housed within the sensilla trichoidae of ghost moths, this is a speculative statement, and should not be presented in the results section, but instead in the discussion.

R: We move this sentence to the discussion part (L262-266). Please see "Interestingly, the presence of unique olfactory structures including enlarged glomeruli observed in both sexes and dominated long trichoid sensilla, mostly non-biased expressions of *TxiaOr19* homologs, along with their non-feeding life traits in adulthood suggests that these ghost moths may employ a primitive pheromone sensing system to locate potential partners."

Line 140-141. "compared with other Lepidopteran moths (in general 50 to 80 glomeruli) (Figure 1E).

Where does this data come from? In addition to Figure 1E here, it is needed to refer to the relevant supplementary data file as well.

R: We have added the glomerular counts in Data S1 and referred to this file in the text.

Line 149-150. "The cumulus which represents a major area involving pheromone reception"

A reference is needed for this statement. Furthermore, is it being suggested that his area involves pheromone processing in the ghost moth? Is anything known about the pheromones used in these species? This should be described in the appropriate section of the introduction.

R: We deleted sentence of "involving pheromone reception" in results. So far, most researches about ghost moth mating or partner seeking were focus on behaviors (Kuenen et al., 1994; Allan and Wang, 2010), hence people speculate that female pheromones or male pheromone were used to attract partner in Hepialidae species. Additionally, only 3 species' male pheromones were identified including *H. californicus* (Kubo et al. 1985, Uchino et al. 1985), *H. hecta* (Schulz et al. 1990), and *E. excrescens* (Marukawa and Mori 2002). Hence, studies of pheromone sensing of

Hepialidae species are very rare in the past. These background of ghost moth pheromone sensing was added to the manuscript. Please see "While most previous research has focused on mating behaviors and pheromone identifications in hepialids (Kuenen et al., 1994; Mallet 1984; Schulz et al., 1990; Chen et al., 2024 Journal of Economic Entomology; Allan and Wang, 2010; Kubo et al. 1985, Uchino et al. 1985; Marukawa and Mori, 2002), few studies have explored their potential pheromone-sensing neural architecture or annotated the odorant receptor family in Hepialidae species."

In this manuscript, we first provide evidence of male and female enlarged glomeruli (Figure 1). Considering these species showed non-feeding bio traits in their adulthoods and different sex roles, we believe that those enlarge glomeruli may function as pheromone processing in hepialids. We add more discussion as "Interestingly, the presence of unique olfactory structures including enlarged glomeruli observed in both sexes and dominated long trichoid sensilla, mostly non-biased expressions of *TxiaOr19* homologs, along with their non-feeding life traits in adulthood suggests that these ghost moths may employ a primitive pheromone sensing system to locate potential partners."

Line 164. "A total of 23 TxiaORs were confirmed to be expressed from the genome and transcriptomes…"

Confirmed to be expressed where? In which tissues specifically?

R: The RT-PCR verification showed that 23 annotated ORs expressed in antennal cDNAs. The OR panel comprised with 35 annotations was obtained from genome and antennal transcriptome. The confirmed 23 ORs were used plus 34 TxiaOr19 tamdem and added up to 57 ORs as shown in the previous text. We agree that the sentence was confusing, so that we have revised this part to make it more clearly to readers as "A total of 23 TxiaOrs were confirmed to be expressed in the antennae of *T. xiaojinensis* via RT-PCR verification, out of annotations from genome and antennal transcriptome assembly". The original annotated ORs were listed in additional Data S2.

Line 168-170. "This array, homologous to TxiaOR19, contained 16 homologues

(TxiatdORs) and 18 pseudogenes (TxiatdpORs), which maintained the largest tandem duplications reported in lepidopterans (Figure s5, data s2).

Figure s5 does not show an array of tandem duplications, it just shows all of the candidate genes' exon/intron structure lined up in separate rows, some of which seem to have similar patterns, but many that do not. The representations are inexplicably named "testNN" and it is not clear the relevance of these names. To make the intended point, It would be far more useful to highlight with different colors homologous exons, and show an additional panel that diagrams their relative positions in the genome on the respective chromosome. The focus on the OR19 tandem expansion of ORs is a central point in this report. So this claim needs to be properly visualized.

R: We have updated Figure S5 which included physical locations of TxiaOR19 homologs on chr14 and exon/intron structures for each homolog with homologous exons indicated by color. We also named all TxiaOR19 homologs.

Line 171-173. "The Maximum-Likelihood phylogeny analysis using 272 ORs showed that the TxiaOR19 array formed an earlier group where canonical type I PRs arose (Figure 2A, Data S3).

In looking at Figure 2A, it is not clear how well that statement is supported. The bootstrap support circle at the branchpoint between the OR19 clade and the canonical PR clade is rather small. It would be better to show the actual bootstrap support value number at this point instead of the circle representation in order to make it clearer to the reader.

R: We accept and updated the tree. The key bootstrap support values of the branch were indicated in the new Figure 2A. The relevant methods, results, and figure legends were revised.

Line 173-175. "This tree topology between the two clades was consistent when cross checked with both Neighbor-Joining and Bayesian methods (Figure s6).

This is true, but those trees do not appear to clearly support such close relationships between the PR clade and the OR19 clade. How can these differences be reconciled?

In Figure 2A the relationship between the two clades appears to be directly approximate, but in the Figure s6 trees, they do not.

R: We re-built the NJ and BY tree as the ML tree was updated, using the same batch of 387 ORs. Please see the updated Figure 2A and Figure S9. To make it clearer, we modified related sentence as "The Maximum-Likelihood phylogeny analysis using 387 ORs showed that the TxiaOr19 array joint an earlier group where canonical type I PRs arose (Figure 2A, Data S3). The earlier separation of canonical PRs and TxiaOR19 tandem was also observed when cross-checked with Neighbor-Joining and Bayes method (Figure S9)."

Line 176-178. "The TxiaOR19 array suggested an earlier emergence than male-biased TxiaOR7, as it could blast to ORs in locust, aphid, soldier flu, mosquito and flea, with homologues predicted by CLANS in mosquito and flea (Figure 2C, Figure s7).

It is not clear how Figures 2C and S7 supports the claim that any TxiaORs have any homology with ORs from mosquito and flea, and it is not clear from figure 2C what data supports this claim. It would have been better to include these ORs (from mosquito and flea) in figure 2A in order to provide better support for homologous relationships between ORs across distant insect orders. It is well known that there is little evidence for homology between ORs across distant insect orders, See Hansson and Stensmyr 2011 review in Neuron, Figure 3. Solid evidence would be needed to be shown to support the claim being made, and as currently presented, that evidence is lacking.

R: We revised this description, and updated the phylogeny tree with adding ORs from mosquito and flea by blasting using TxiaOR19 in the new Figure 2A. Please see "Differing from ORs found in evolutionarily later moths, the TxiaOR19 array could blast to ORs in locusts, aphids, soldier flies, mosquitoes, and fleas, with homologues predicted by CLAN [37] (Figure 2A and Figure S9). However, the male-biased TxiaOR7 failed to blast to any ORs from those earlier species (Figure S11, S12)."

Line 183-185. "suggesting possible evolutionary patterns similar to the duplicated zen family of orange swift moth, H. sylvina, which diverged to new functional gene families

Shx"

This is a discussion point that should not be presented as results.

R: We delete this sentence in the results.

Line 185-186. "On the other hand, PRs could be traced back to a single LarmPR1 in the caddisfly."

What is this claim based on? It seems like it is being made from Figure 2D. However, it is not clear that anything shown in Figure 2D supports the claim. LarmPR1 does not seem to be connected or tracing to any other ORs in that figure.

R: We apologize for this unclearly claim and we updated Figure 2A and marked the caddisfly PR. The sentence was revised as "it showed that canonical PRs and a single LarmPR1 in the caddisfly formed a clade in the phylogeny".

Line 190-191. "Specifically, TxiaOR19 array arose from the LarmOR19-OR13a tandem (Figure 2E)."

What is the statistical support (Bootstrap values or Likelihood Ratio or other) for this claim? It does not seem to be apparent in the figure, so it is difficult to assess how well supported the phylogenetic relationships are.

R: We revised this sentence to "Specifically, *TxiaOr19* array formed the same clade with *LarmOr19-Or13a* tandem". Please see Figure 2A.

Line 241-243. "which fits the predicted non-PR functioning of TxiaOR19 to a non-canonical female emission and the olfactory architectural observations."

What functional assays were done to show that TxiaOR19 does or does not function as a PR? Is this based on the molecular docking predictions shown in figure S8? If that is the case, it appears in that figure that the male-biased OR7 shows higher affinity to the female-specific emitted compound, Oleamide. This would seem to point to the potential for OR7 being a PR. How can the claim made here be reconciled with what is shown in Figure S8?

R: We agree that TxiaOR7 in the docking simulation could be a better hit for PRs, but

we did not map this OR using canonical PRs along with the TxiaOR19. This TxiaOR7 may support the recent reports of the novel PR clade in lepidopteran species and worth further investigation. As we here in this work focus on the canonical PR clade, we revised this sentence as "Our results indicated that the sex roles of *T. xiaojinensis* adults were different from those of higher lepidopterans during mating allocation, which could relate to predicted unconventional TxiaOR19 tandem, the non-canonical female emission and the olfactory architectural observations." And also in the discussion "One interesting result in our molecular docking predictions is that the male-biased OR7 shows a higher affinity to oleamide, suggesting that TxiaOR7 potentially serves as an ancestral PR of the novel PR clade in lepidopteran species. However, more experimental functional evidence needs to be provided for both TxiaOR19 and TxiaOR7 to support their evolutionary roles in lepidopteran species."

Discussion

Line 251-253. "We have also discovered that both males and females of these moths possess a compact olfactory system with distinct structures involved in sexual recognition"

What is the basis for this claim? Which structures? It was not shown that any structures are involved in sexual recognition in the ghost moths examined in this report. I would suggest to be careful about making assumptions about homologous structures from higher moths while at the same time trying to argue in favor of great distinctions in the ancestral ghost moths compared to the higher moths. It is better to present data about the function of the structures of the ghost moth instead.

R: We accept and rephrase the sentence as "Interestingly, the presence of unique olfactory structures including enlarged glomeruli observed in both sexes and dominated long trichoid sensilla, mostly non-biased expressions of *TxiaOr19* homologs, along with their non-feeding life traits in adulthood suggests that these ghost moths may employ a primitive pheromone sensing system to locate potential partners."

Line 266-267. "OR19 duplications in these ghost moths do not appear to have enhanced

functions"

What does this mean? Almost no evidence was presented demonstrating the function of these ORs.

R: Although we tried to deorphanize the TxiaOR19 by using molecular docking software, but it is still hard to show TxiaOR19' functions. Hence, we toned down OR function in the manuscript and delete this sentence in here.

Line 294. "These cues also suggest the ancestral roles of the ghost moths in terms of their sex role systems."

It is not clear what is meant by this.

R: We rephrase this sentence as "Given their ecological traits, highly redundant genome, and a smaller number of ORs, we believe that these Himalayan ghost moths may retain pheromone sensing abilities and exhibit primitive sex roles within Lepidoptera. Further investigation into the function of ORs remains crucial for refining this perspective."

Figures and Data

Figure S8. "or head space volatiles (blue)"

Are these blue traces both from females, or something else? It should be clarified in the legend.

R: We added the information in the legend.

Table S4. Concerning Cydia pomonella, Wan et al., 2019 reported 85 ORs, 65 GRs and 39 IRs (see page 4), so it is not clear what is being referred to here for 82 ORs, 15 IRs and 1 GR.

R: We apologize for this mistake. We then check all data in the Table S4 (the Table S2 in revision files) and we hope it is clearer now.

Reviewer #2: The manuscript entitled "A ghost moth olfactory prototype of the

lepidopteran sex communication" is reviewed. This is a highly multidisciplinary work and difficult for me (and maybe to many other researchers) to review. Based on my expertise of insect genomics and molecular adaptation, I am providing my comments mainly on the related sections (i.e. genome and transcriptome assembly, orthology analyses, and gene evolution). In general, this is publishable material from my point of view, with some revision required to improve the manuscript. Here I list two of my major concerns and some minor suggestions.

R: We appreciate your thorough examination and valuable suggestions. We have carefully incorporated all your recommendations into our manuscript and figures. In the relevant sections of the manuscript, we have highlighted the changes in yellow. We believe that these modifications have enhanced the clarity and readability of the manuscript compared to the previous version.

Main concerns:

1. For the sensilla distribution analysis (Figure 1D), I feel the result is potentially biased by the different ways of counting the sensilla trichoidae in the retrieved studies. For example, Gargi et al. were counting all the sensilla trichoidae on the entire antenna, while the authors possibly divided the number by number of flagellum segments to represent the "mean" number of sensilla trichoidae of each flagellum to make it comparable with the data matrix generated in the current study. But this is assuming that all the flagellum segments have similar number of sensilla trichoidae, which is unlikely to be true. Ideally, counting all the sensilla trichoidae is required to perform the statistic analyses. My suggestion is to not include this section in the manuscript as it is not necessary for authors to derive their conclusions.

R: We agree. The statistics for sensilla tricoidae was removed from the manuscript and we toned down related descriptions about sensilla.

2. The completeness assessment of the genome assembly of T. armoricanus is not available. So does the transcriptome of A. jianchuanensis. These are very crucial as if the assembly less complete, the subsequent analyses will be confounded. My

suggestion is to run BUSCO on the genome and transcriptome assembles.

R: We added BUSCO results for both *T. armoricanus* genome and *A. jianchuanensis* transcriptome in the manuscript. Please see "BUSCO analysis indicates 90% of single-copy insect orthologs are complete. We also conducted BUSCO analysis towards the transcriptomes of *A. jianchuanensis*, and a completeness of 96.6% was observed".

3. I think the figures are too complicated for most readers. Too packed figures make it unpleasant to read but the role of the figures should be helping readers to quickly get the key information. For example, Figure 1B, C, D, F, G, and H; Figure 2B, C, E, H, and G; Figure 3B and C, can be separated figures in SI.

R: We agree that visualization concerns and have followed suggestions. We, therefore, re-organized the figure panels, by moving Figure 1C, F, G, 2B, C, and 3B, C to the supplementary materials. The phylogenic tree was updated combining previous Figure 2A and D.

Minor suggestions:

1. Line 112 (results): I was expecting to see the assembly report of T. armoricanus as well, but it seems missing in the result sections.

R: We added assembly report of *T. armoricanus* in revised "Genome of *T. armoricanus* was sequenced on Illumina HiSq2000, harvesting 877.7 Gb clean data to construct scaffolds. The final assembly presented 3,168 Mb total length of the scaffolds, with N50 of 27.8 kb and 176.2 kb for contigs and scaffolds, respectively. BUSCO analysis indicates 90% of single-copy insect orthologs are complete. We also conducted BUSCO analysis towards the transcriptomes of *A. jianchuanensis*, and a completeness of 96.6% was observed. Genome and transcriptomes were subsequently employed in downstream analysis."

2. Line 136: maybe address why H. cunea was selected for the comparison.

R: This comparison was removed according to Major comment 1. *H. cunea* is a signature species which presents significant sex dimorphism of the antennae among the

tested species, so that we used this species as a calibrator to assess sensillar counts in *T. xiaojinensis*. Due to the counting method limitations raised in Major comment 1, we removed this part.

3. Line 165: not sure what the "57 annotations" means here.

R: We are sorry for this unclear description. The relevant part was revised and additional supplementary file was added. "A total of 23 *TxiaOrs* were confirmed to be expressed in the antennae of *T. xiaojinensis* via RT-PCR verification, out of annotations from genome and antennal transcriptome assembly" (Data S2 listed annotated TxiaORs for used in PCR verifications)

4. Line 167: what is LG14?

R: We revised LG14 to chr14.

5. Line359-360: please describe how the short read sequences were generated

R: We revised this part as "Meanwhile, short-reads library was constructed by Illumina platform with the same batch of *T. xiaojinensis* DNA, and 165 Gb raw data were generated. After filtering, the remaining clean reads with Q > 20 were used for minimap2 mapping onto the genome assembly which was later polished by NextPolish (https://github.com/Nextomics)."

6. Line 361-362: please provide more information about how the non-insect contigs were identified.

R: We added relevant information to this part. Please see "To remove the DNA pollutions from the other organisms, the polished genome was aligned against the NCBI nucleotide (NT) database, and the contigs which were aligned to the sequences from fungi, plants, or virus were removed."

7. Line384: not sure what it means by "second generation genome" since there is no previous version of genome assembly for the species.

R: We are sorry for the confusion description. The relevant part was revised as "Genome".

8. Line 406-407: What I know about orthofinder is to get the orthologs from protein sequences. Here it says "the orthologous genes of these 18 insect species were inferred from their genomes or transcriptomes" but those are DNA sequences not proteins.

R: We are sorry for the mistake. The orthofinder does refer to protein sequences. We revised this part.

9. Line425: I thought this should be protein-protein searching, but the queries are DNA, this is somewhat confusing me.

R: We apologize. We revised this part.

10. Line430: please provide more information about how the ORs were verified by PCRs.

R: The detailed methods were described in "Characterizations of Ors" section following this part. So that we removed this sentence in order not to draw any confusion.

Graphical abstract

Click here to access/download;Graphical Abstract;Graphical Abstract.tif

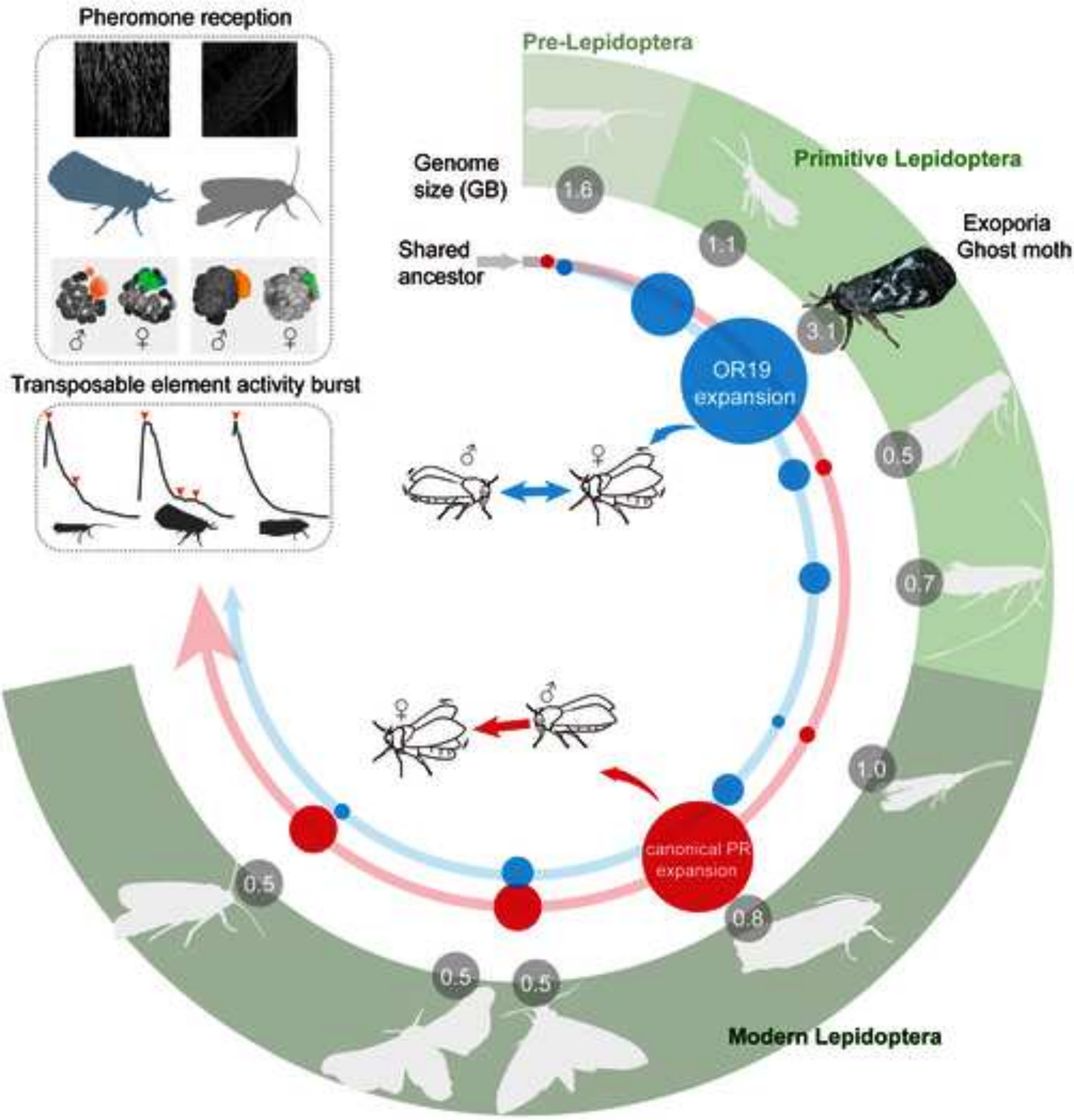

Supplement: giae044_GIGA-D-23-00252_Revision_1 [file giae044_giga-d-23-00252_revision_1.pdf]
