# Supplementary material for: A ghost moth olfactory prototype of the lepidopteran sex communication
Source: Gigascience. 2024 Jul 19;13:giae044. doi: 10.1093/gigascience/giae044 (PMC11258902; doi:10.1093/gigascience/giae044)

GigaScience

A ghost moth olfactory prototype of the lepidopteran sex communication

--Manuscript Draft--

**Manuscript Number:**	GIGA-D-23-00252R2
**Full Title:**	A ghost moth olfactory prototype of the lepidopteran sex communication
**Article Type:**	Research
**Funding Information:**	Major Science and Technology Project of Qinghai Province (2021-SF-A4-1) — Prof Ri-Chou Han<br>GDAS Special Project of Science and Technology Development (2022GDASZH-2022010106) — Dr Rui Tang<br>Guangdong Basic and Applied Basic Research Foundation (2020A1515011366) — Prof Ri-Chou Han<br>National Key R & D Program of China (2023YFC2606900) — Dr Rui Tang
**Abstract:**	Sex role differentiation is a widespread phenomenon. Sex pheromones are often associated with sex roles and convey sex-specific information. In Lepidoptera, females release sex pheromones to attract males, which evolve sophisticated olfactory structures to relay pheromone signals. However, in some primitive moths, sex role differentiation becomes diverged. Here, we introduce the chromosome-level genome assembly from ancestral Himalaya ghost moths, revealing a unique olfactory evolution pattern and sex role parity among Lepidoptera. These olfactory structures of the ghost moths are characterized by a dense population of trichoid sensilla, both larger male and female antennal entry parts of brains, compared to the evolutionary later Lepidoptera. Furthermore, a unique tandem of 34 odorant receptor 19 homologs in Thitarodes xiaojinensis (TxiaOr19) has been identified, which presents overlapped motifs with pheromone receptors (PRs). Interestingly, the expanded TxiaOr19 was predicted to have unconventional tuning patterns compared to canonical PRs, with non-sexual dimorphic olfactory neuropils discovered, which contributes to the observed equal sex roles in Thitarodes adults. Additionally, transposable element activity bursts have provided traceable loci landscapes where parallel diversifications occurred between TxiaOr19 and PRs, indicating that the Or19 homolog expansions were diversified to PRs during evolution and thus established the classic sex roles in higher moths. This study elucidates an olfactory prototype of intermediate sex communication from Himalaya ghost moths.
**Corresponding Author:**	Ri-Chou Han, Ph.D.<br>Guangdong Academy of Sciences<br>Guangzhou, Guangdong CHINA
**Corresponding Author Secondary Information:**	
**Corresponding Author's Institution:**	Guangdong Academy of Sciences
**Corresponding Author's Secondary Institution:**	
**First Author:**	Rui Tang, Ph.D.
**First Author Secondary Information:**	
**Order of Authors:**	Rui Tang, Ph.D.<br>Cong Huang<br>Jun Yang<br>Zhong-Chen Rao<br>Li Cao

Powered by Editorial Manager® and ProduXion Manager® from Aries Systems Corporation

	Peng-Hua Bai
	Xin-Cheng Zhao
	Jun-Feng Dong
	Xi-Zhong Yan
	Fang-Hao Wan
	Nan-Ji Jiang
	Ri-Chou Han
**Order of Authors Secondary Information:**	
**Response to Reviewers:**	GIGA-D-23-00252R1<br>A ghost moth olfactory prototype of the lepidopteran sex communication<br>Rui Tang; Cong Huang; Jun Yang; Zhong-Chen Rao; Li Cao; Peng-Hua Bai; Xin-Cheng Zhao; Jun-Feng Dong; Xi-Zhong Yan; Fang-Hao Wan; Nan-Ji Jiang; Ri-Chou Han<br>GigaScience<br><br>Dear Prof Han,<br><br>Your manuscript "A ghost moth olfactory prototype of the lepidopteran sex communication" (GIGA-D-23-00252R1) has been assessed by our reviewers. Based on these reports, and my own assessment as Editor, I am pleased to inform you that it is potentially acceptable for publication in GigaScience, once you have carried out some essential revisions suggested by our reviewers.<br><br>Their reports, together with any other comments, are below. Please also take a moment to check our website at https://www.editorialmanager.com/giga/ for any additional comments that were saved as attachments.<br><br>Once you have made the necessary corrections, please submit a revised manuscript online at:<br><br>https://www.editorialmanager.com/giga/<br><br>If you have forgotten your username or password please use the "Send Login Details" link to get your login information. For security reasons, your password will be reset.<br><br>Please include a point-by-point within the 'Response to Reviewers' box in the submission system. Please ensure you describe additional experiments that were carried out and include a detailed rebuttal of any criticisms or requested revisions that you disagreed with. Please also ensure that your revised manuscript conforms to the journal style, which can be found in the Instructions for Authors on the journal homepage. On top of these, we suggest you find a copy editing company or friendly native English speaker to polish the grammar.<br><br>The due date for submitting the revised version of your article is 04 Aug 2024.<br><br>We look forward to receiving your revised manuscript soon.<br><br>Best wishes,<br><br>Hongfang Zhang<br>GigaScience<br>www.gigasciencejournal.com<br><br>Dear editor,<br>Thank you for considering publishing our manuscript in Gigascience. We have carefully revised our manuscript based on all suggestions from the reviewer and have answered the reviewer’s questions point-by-point in the following letter. Furthermore, we have edited the language and fixed some grammatical errors, and we believe the current version is more readable. All changes have been marked by highlighting them (in

green) in the text in the R2 submission files. We truly hope that the R2 revised manuscript is in good format and scientific quality to meet the journal's publishing standards. For more details, please see the manuscript or the response letter.
Best regards,
Ri-Chou Han

Reviewer reports:
Reviewer #1: The manuscript is substantially improved after the previous round of revisions and most of the reviewer comments have been addressed. However, reading the updated manuscript in this new light has brought to the forefront several other minor concerns that must be addressed before the manuscript should be published. Furthermore, while the manuscript is generally written very well, numerous small language errors are noticeable throughout the text. It is recommended to ensure the manuscript is revised for language after all scientific edits are made.

R: We appreciate your detailed assessments and suggestions. We followed each of the suggestions and revised our manuscript carefully. Accordingly, we have modified related parts in the manuscript (please see the green highlights in the text or in the response letter below). After addressing these concerns and fixing grammatical errors, we feel that your suggestions have indeed helped us improve the quality of this manuscript and make it more readable.

Minor revisions are suggested as follows:
Introduction
Line 94-96. "Moreover, ghost moths have undergone asymmetrical divergence of duplicated genes to deliver functional alterations in subsequent species, proving insights into the evolutionary process of Lepidoptera."
Which genes? Is this a widespread phenomena across all different kinds of genes, or is this referring to specific genes or gene families? Please clarify.

R: The duplication genes mentioned in here are zen gene. And the asymmetrical divergence is a widespread phenomenon in all genes during evolution. In the manuscript, we clarified as “Moreover, ghost moths have undergone asymmetrical divergence of duplicated gene, e.g. zen gene, to deliver functional alterations in subsequent species, providing insights into the evolutionary process of Lepidoptera”.

Results
Line 211-212. "In all, the genomic backgrounds and receptor repertoires are specific in T. xiaojinensis, which may confer to the structural specificities described above."
It is not clear what this statement means. How are these features specific in T. xia, and what structural specificities are being referred to.

R: We deleted this statement, as it is confusing in the current version of manuscript since we removed some functional observations from the text.

Line 234-235. "possible functional drift of TxiaOR19."
Please describe what is meant here more clearly with this term. Functional drift in what manner, or relative to what?

R: We delete the term “functional drift” and change the sentence as “The replacement of the TxiaOR19 duplications with canonical PRs suggests possible different tuning characteristics of TxiaOR19 from a PR that narrowly tunes to sex pheromone components”

Line 241-244. "The results showed that TxiaOR19 had less binding affinity towards the panel of the ghost moth emissions, and its responding spectrum were relatively broad, indicating that ghost moth T. xiaojinensis may show unconventional courtship behaviors comparing to higher moths."
Might this result also suggest that other ORs, especially within the OR19 tandem array may be instead tuned to ghost moth emissions? Why aren't docking profiles examined for other OR19 sub-family receptors? In any case, this kind of statement seems more like a discussion statement than a results statement. I understand it is used to transition to the next paragraph, however, the logic for looking at behavior in males and females (as it turns out, non-dimorphic behavior), could just as well follow from the lack

of sexual dimorphism in the antennal lobe, perhaps much better, as compared to the hypothetical ligand docking studies, which are far from complete.

R: We redocked all TxiaOR19 array ORs to ligands, and the results showed that all tested TxiaORs are not specifically tuned to the female-specific compound oleamide (see Figure S16), which supports our idea that the TxiaOR19 array has different tuning characteristics compared to the narrowly tuned PRs. In the manuscript, we have toned down our statements regarding OR functional docking, as this aspect is not our main focus in discussing the evolution of moth pheromone receptors. Please see the following statement: “The results showed that the TxiaOR19 array had less binding affinity towards the panel of ghost moth emissions, and the responding spectrum was relatively broad for all 16 tandem ORs.”

Regarding the transition from molecular to behavioral aspects in the manuscript, we followed your suggestion by initially discussing the lack of sexual dimorphism in the antennal lobe before transitioning to the discussion of non-dimorphic behavior. See the “Considering ghost moths, including T. xiaojinensis, lack sexual dimorphism in their antennal lobe (Figure 1D), we hypothesized that T. xiaojinensis may also exhibit unconventional calling and mating behaviors compared to higher moths.”

Discussion.
Line 256. "Himalaya ghost moths offer a basal model for the study of olfaction evolution in insects"
I am not sure it is correct to say "insects" here. It may be more correct instead to say "Lepidoptera" or at the very most, the "Lepidoptera/Trichoptera" sister clades, since caddisflies are also included….but not more generally insects.

R: We adjusted “insect” to “Lepidoptera”.

Line 278-279. "However, more experimental functional evidence needs to be provided for both TxiaOR19 and TxiaOR7…"
Again, here, shouldn't this refer to the TxiaOR19 sub-family/array ORs more generally, instead of only TxiaOR19?

R: We re-docked the TxiaOR array to ligands, please see the Figure S16. And we also adjust the “TxiaOR19” to “TxiaOR19 array” in the manuscript.

Line 282. "the TxiaOR19 array and LarmPR1 share motifs that reflect some exons on the loci"
It's not clear what this means. How do the motifs reflect exons? Which exons and which loci?

R: We removed “that reflect some exons on the loci” as there was no hard evidence to support this statement.

Line 282-283. "These motifs were separated before the evolution of Lepidoptera."
This is a vague statement. Do you mean before Lepidoptera and Trichoptera diverged from each other, or before the ghost moths split off from the more recently evolved moths? Please clarify.

R: We apologize for this vague statement. We clarified as “These motifs were likely to be already separated before Lepidoptera and Trichoptera diverged from each other”.

Line 286-287. "On the other hand, the shared motif regions could be traced back to earlier dipteran species"
Which shared motif regions? Are you referring to the ones in the PRs as shown in Fig 2C/2D/2E? Or motifs present in other organisms, as mentioned in the previous sentence?

R: We hope to compare the motifs of PRs to the TxiaOR19. We revised this sentence as “On the other hand, PRs shared motif regions with TxiaOR19 and the later could be blasted to ORs in dipteran species where large OR duplications existed such as in Bactrocera dorsalis”.

Powered by Editorial Manager® and ProduXion Manager® from Aries Systems Corporation

Line 297-298. "Enlarged MGCs in butterfly Piers rapae suggest that the sexual dimorphic sex pheromone recognition system is widely used by Lepidoptera"
Please check the species, as Piers rapae is not at all mentioned in the cited article, no. 49. I would also further caution against making this statement about the sexually dimorphic sex pheromone recognition system, as the authors of the cited article emphasize that their butterfly MGC is not implied to be homologous to the moth MGC.

R: We apologize for the inconsistent citation and unclearly statement. We revised this sentence as “The enlarged MGCs in the butterflies, such as Pieris rapae and Godyris zavaleta [49], suggest that the sexual dimorphic brain organizations are widely presented by Lepidoptera, however, the link between morphology and pheromone recognition in butterflies requires further examination”.

Materials and Methods.
Section on Scanning electron Microscopy.
For the washes, dehydration and drying steps, please specify the length of times for each of these steps.
R: We revised the paragraph by adding these essential information as “The antennae of 1-3 d adults were cut from base and fixed in 0.25% glutaraldehyde at 4 °C overnight. After three washes at room temperature with 0.1 M phosphate-buffered saline (PBS, pH 7.4) for 15 min each, antennae were dehydrated through a laddered ethanol series (30, 50, 70, 80, 90, and 100%) for 15 min each and dried for 15 min in a critical point drier (Bal-Tel CPD 030) before mounted on aluminum stubs. The mounted antennae were coated with gold spray (Bal-Tel SCD 005) and observed with SEM instrument (FEI Quanta 200,).”

Line 376. Change pollutions to contamination.
R: Revised.

Figures and Tables
Figure 2, Panels C-E. It is unclear why Motifs 1-3 are shown in 2C, while Motifs 4-6 are shown in 2D. And in 2E, motif 2 appears very similar to Motif 4, while Motif 3 appears very similar to Motifs 5 and 6. Based on this last point, the text from lines 204-211 is confusing, especially considering that in 2C and 2D, for the PR consensus representations, either Motifs 1-3 are shown in 2C or Motifs 4-6 are shown in 2D. If this is the PR consensus, and it is the same representation for 2C and 2D, shouldn't Motifs 1-6 be shown for both? Please try and re-write the relevant text in the main manuscript and figure legend so that this concept is clearer.

R: The Motifs 1-3 are highly conserved motifs of PRs, reviewed by Zhang and Löfstedt in 2015 (doi.org/10.3389/fevo.2015.00105). We firstly used the MEME suite to check the signature Motifs of the PR clade, and confirmed them according to published works. The same approach was used then to test TxiaOR19 mapped ORs, and we thus named the later motifs as Motif 4-6. As the two clades were separately investigated, they had some overlaps but should not be placed together. We are sorry for this mistake and removed PR consensus from Figure 2D in order not to draw any confusions. The descriptions in the manuscript as “Using MEME suite, we found that LarmPR1 exhibited all three signature motifs of PR consensus regions as reported before” and also as “On the other hand, the majority of TxiaOR19-mapped ORs had two motifs, with some having three motifs (named as motif 4-6) upon checking with the same approach”.

Figure 3, Panel A. It is not clear where the burst events are. Are they marked by the red arrowheads? This should be written out clearly in the figure legend.
R: The red arrows indicate TE burst. We have added this information in the figure legend of Figure 3. See “Red arrowheads indicate TE burst events”

Figure S2. What do the black squares and grey bars represent? Please indicate their meaning in the figure legend.
R: We added explanations in the figure legend. Please see “Where square dots indicate each measurement of individual sample, grey bars indicate the ranges of O.I. from the species”

Figure S3. It appears that different sensilla types are present in the various different

Powered by Editorial Manager® and ProduXion Manager® from Aries Systems Corporation

	panels. Please indicate, with arrows and notations, in each panel, which sensilla type are the sensilla trichodea, as it is not obvious to an unfamiliar reader.<br>R: We added arrowheads to indicate sensilla trichodae in each panel, with figure legend revised “Arrowheads indicate representative sensilla trichodae morphology in each panel”.<br><br>Figure S8. It appears that only 18 genes are shown here. It should be made clear in the figure legend that these are the intact homologues and the pseudogenes are excluded, if that is indeed the case.<br>R: Yes, it is the case. We added relevant clarification to the figure legend as “Tandem TxiaOr19 intact Or duplications exclude pseudogenes”.<br><br>Table S2. The title of this table has been updated to say "comparison of chemosensory genes in head among the tested Lepidoptera. Is this referring to head only? Or is it an indication of head/antennal expression? Furthermore, in the previous round of revision, it was mentioned that the numbers for C. pomonella were inaccurate and they have been updated to reflect all ORs/GRs/IRs identified in the genome, as reported by Wan et al., 2019. However, now that the title has changed to reflect chemosensory genes in head, the numbers for C. pomonella are again inaccurate, as they reflect presence of these genes in the genome, not in the head.<br>R: We are sorry for this. The correct title for this table is revised to “Table S2. Comparison of chemosensory gene annotations among the tested or reported Lepidoptera.”<br><br>Reviewer #2: My comments to the manuscript have been properly addressed. I have no further concerns.<br>R: Thank you again for your time.
**Additional Information:**	
**Question**	**Response**
Are you submitting this manuscript to a special series or article collection?	No
**Experimental design and statistics**<br><br>Full details of the experimental design and statistical methods used should be given in the Methods section, as detailed in our Minimum Standards Reporting Checklist. Information essential to interpreting the data presented should be made available in the figure legends.<br><br>Have you included all the information requested in your manuscript?	Yes
**Resources**<br><br>A description of all resources used, including antibodies, cell lines, animals and software tools, with enough information to allow them to be uniquely identified, should be included in the Methods section. Authors are strongly	Yes

Powered by Editorial Manager® and ProduXion Manager® from Aries Systems Corporation

encouraged to cite Research Resource Identifiers (RRIDs) for antibodies, model organisms and tools, where possible.<br><br>Have you included the information requested as detailed in our Minimum Standards Reporting Checklist?	
**Availability of data and materials**<br><br>All datasets and code on which the conclusions of the paper rely must be either included in your submission or deposited in publicly available repositories (where available and ethically appropriate), referencing such data using a unique identifier in the references and in the "Availability of Data and Materials" section of your manuscript.<br><br>Have you have met the above requirement as detailed in our Minimum Standards Reporting Checklist?	Yes

Manuscript

Click here to access/download;Manuscript;manuscript_R2_June 21_autInputs.docx

A ghost moth olfactory prototype of the lepidopteran sex communication

Rui Tang[1#a], Cong Huang[23#], Jun Yang[4], Zhong-Chen Rao[1], Li Cao[1], Peng-Hua Bai[5], Xin-Cheng Zhao[6], Jun-Feng Dong[7], Xi-Zhong Yan[4], Fang-Hao Wan[23], Nan-Ji Jiang[8]*, Ri-Chou Han[1]*

1 Guangdong Key Laboratory of Animal Conservation and Resource Utilization, Guangdong Public Laboratory of Wild Animal Conservation and Utilization, Institute of Zoology, Guangdong Academy of Sciences, Guangzhou, China 510260

2 State Key Laboratory for Biology of Plant Diseases and Insect Pests, Institute of Plant Protection, Chinese Academy of Agricultural Sciences, Beijing, China 100193

3 Shenzhen Branch, Guangdong Laboratory for Lingnan Modern Agriculture, Genome Analysis Laboratory of the Ministry of Agriculture, Agricultural Genomics Institute at Shenzhen, Chinese Academy of Agricultural Sciences, Shenzhen, China 518120

4 College of Plant Protection, Shanxi Agricultural University, Taigu, Shanxi, China 030801

5 Institute of Plant Protection, Tianjin Academy of Agricultural Sciences, Tianjin, China 300384

6 Henan International Laboratory for Green Pest Control, College of Plant Protection, Henan Agricultural University, Zhengzhou, China 450046

7 Forestry College, Henan University of Science and Technology, Luoyang, China 471000

8 Department of Evolutionary Neuroethology, Max Planck Institute for Chemical Ecology, Hans-Knöll-Straße 8, Jena, Germany D-07745

*Correspondence: Nan-Ji Jiang, njiang@ice.mpg.de Hans-Knöll-Straße 8, Jena, Germany. Tel. +49 (0)3641 57-1456; Ri-Chou Han, hanrc@giz.gd.cn, 105 Xingang West Road, Haizhu District, Guangzhou. Tel. +86 020-84191089

# Equal contribution was claimed.

Rui Tang [0000-0002-9313-0802]; Cong Huang [0000-0001-6149-4989]; Jun Yang; Zhong-Chen Rao; Li Cao [0000-0001-5936-4966]; Peng-Hua Bai; Xin-Cheng Zhao; Jun-Feng Dong; Xi-Zhong Yan; Fang-Hao Wan [0000-0002-4275-8632]; Nan-Ji Jiang [0000-0003-4251-2795]; Ri-Chou Han [0000-0002-4120-3803]

Abstract

Sex role differentiation is a widespread phenomenon. Sex pheromones are often associated with sex roles and convey sex-specific information. In Lepidoptera, females release sex pheromones to attract males, which evolve sophisticated olfactory structures to relay pheromone signals. However, in some primitive moths, sex role differentiation becomes diverged. Here, we introduce the chromosome-level genome assembly from ancestral Himalaya ghost moths, revealing a unique olfactory evolution pattern and sex role parity among Lepidoptera. These olfactory structures of the ghost moths are characterized by a dense population of trichoid sensilla, both larger male and female antennal entry parts of brains, compared to the evolutionary later Lepidoptera. Furthermore, a unique tandem of 34 odorant receptor 19 homologs in *Thitarodes xiaojinensis* (*TxiaOr19*) has been identified, which presents overlapped motifs with pheromone receptors (PRs). Interestingly, the expanded *TxiaOr19* was predicted to have unconventional tuning patterns compared to canonical PRs, with non-sexual dimorphic olfactory neuropils discovered, which contributes to the observed equal sex roles in *Thitarodes* adults. Additionally, transposable element activity bursts have provided traceable loci landscapes where parallel diversifications occurred between *TxiaOr19* and *PRs*, indicating that the *Or19* homolog expansions were diversified to *PRs* during evolution and thus established the classic sex roles in higher moths. This study elucidates an olfactory prototype of intermediate sex communication from Himalaya ghost moths.

Keywords: Genome; Olfactory evolution; Neuroecology; Lepidoptera; Ghost moth; Sex role

Introduction

Sexual dimorphism is ubiquitous across the animal kingdom [1]. For most animals, mating by partner allocation is an indispensable process to ensure population continuity [2]. Sex roles often form under the pressure of sexual selection [3]. In general, the female exhibiting greater parental investment becomes a limiting resource for the less caring male so that the latter competes for accessing to the former [4]. In insects, sex pheromone becomes an effective investment for male to gain opportunities to mate with female successfully. One well studied example is that, in fruit fly *Drosophila melanogaster*, males typically release a specific pheromone called cis-11-vaccenyl acetate (cVA) to gain an advantage in mating [5]. However, sex roles appear to be reversed in moths [6]. Female moths invest in synthesizing and releasing sex pheromones to attract male moths, and males have evolved distinct structures for sensing pheromones [7,8]. Therefore, the study of pheromone and pheromone perception can expand our understanding of the evolution of sexual roles in animals.

One well-known animal lineage that relies on pheromone communication is Lepidoptera, comprising nearly 160,000 extant species and forming a key branch of insects [9]. Lepidoptera pheromones were well-studied in the last decade, and most can be classified into type 0, I, II, and III, according to their hydrocarbon chains, double-bond allocations, and terminal functional groups [10]. Among them, type I pheromones, consisting of straight-chain acetates, alcohols, or aldehydes with 10 to 18 carbon atoms, make up 75% of all known sex pheromones and are employed by most moth families [11]. Pheromones are detected by pheromone receptors (PRs)/odorant receptor co-receptors (ORco) on the dendrites of olfactory sensory neurons. Based on the pheromone types, the corresponding PR family can be classified into type 0, I, and II clades [12]. However, the recent discovery of *Lampronia capitella* OR6/Orco and *Spodoptera littoralis* OR5/ORco has revealed a

novel ‘PR clade’ that is distant from the type I PR clade [13,14]. This implies that the mechanisms underlying the evolutionary process of ORs for detecting pheromones in Lepidoptera need to be explored.

The neural architectures of pheromone perception appear to be conserved in moths [15]. A typical perception of type I pheromone is achieved through a label-lined olfactory coding pattern in higher moths, such as Noctuidae. Pheromones are tuned by olfactory sensory neurons housed in sensilla trichoidae on the antennae. After the PR/ORco complex has been activated by the corresponding pheromone, the potential signals are projected to the primary olfactory center, the antennal lobe [16]. In Lepidoptera, the antennal lobe shows obvious sexual dimorphism. The male-specific macroglomerular complex (MGC) locates at the entry of the antenna and mainly processes pheromone signals [17], in addition in some species e.g. *Cydia pomonella* and *Bombyx mori*, it also responds to plant volatiles [18,19]. The counterparts of the MGC in females are usually called the large female glomeruli (LFG) that process oviposition and host-choosing signals, but LFG glomeruli are not generally enlarged in size as the MGC [20,21].

The ghost moths (Hepialoidae: Hepialidae) from Exoporia are primitive Lepidoptera species and form an especially interesting lineage for studying the evolution of sex roles and pheromone communication [22]. Hepialids represent an early branch from the line leading to the heteroneuran Ditrysia, and the latter includes almost all the lepidopteran species which use typical PR-based olfaction for pheromones. Notably, the sex roles of Hepialidae species show diversity; for example, *Hepialus hecta* and *H. humuli* exhibit courtship behavior that is very different from the usual moth pattern, as males hover in groups to attract females [22]. Moreover, ghost moths have undergone asymmetrical divergence of duplicated gene, e.g. *zen* gene, to deliver functional alterations in subsequent species, providing insights into the evolutionary process of Lepidoptera [23]. *Thitarodes*,

Ahamus, and *Hepialus* ghost moths, as the hosts of *Ophiocordyceps sinensis* medicinal fungus, are endemic to the Qinghai-Tibet Plateau [24]. The isolated ecological habitat and prolonged life cycle of these so-called Himalaya ghost moths provide ideal opportunities for the retention of pheromone receptive characteristics from shared ancestors of Lepidoptera [25-27]. While most previous research has focused on mating behaviors and pheromone identifications in hepialids [22, 28-34], few studies have explored their potential pheromone-sensing neural architecture or annotated the odorant receptor family in Hepialidae species.

In this study, we presented the unique evolutionary position of olfaction in ghost moths, characterized by comparative neurology and phylogenomics. Our results demonstrated that the antennal lobes of both male and female of three Himalaya ghost moth species (*Ahamus jianchuanensis*, *Thitarodes armoricanus*, *T. xiaojinensis*) have an enlarged antennal entry part and lack obvious sexual dimorphism, when compared to evolutionary later Lepidopterans. Comparative genomics further revealed that the ghost moth *T. xiaojinensis* expanded a specific *Or19* tandem array instead of the classic type I PR clade. Behavioral tests indicated that the ghost moth *T. xiaojinensis* exhibits similar sex roles between males and females in courtship, possibly due to their pheromonal neural architectures without sexual dimorphism and the specific *Or19* tandem array. In summary, this study uncovers a mechanism for the occurrence of functional ORs such as PRs through asymmetric divergence in Lepidoptera.

Results

Chromosome-level genome assembly of Himalaya ghost moth

A *T. xiaojinensis* (NCBI:txid1589740) larva was sequenced using Nanopore long-read technology, resulting in 319.9 Gb of clean reads. The draft genome of 3.1 Gb, comprising 1,645

contigs with a contig N50 of 5.4 Mb, was assembled using NextDenovo, corrected with minimap2 and NextPolish, and refined by removing contaminants. Utilizing Hi-C interaction data, the primary assembly was divided into 31,434 contigs, and 31,391 contigs (99.86% in length) were anchored to 32 chromosomes (Figure S1). BUSCO analysis revealed 91.8% complete genes in the final chromosome-level genome assembly, which was subsequently employed in downstream analysis.

The genome of *T. armoricanus* (NCBI:txid92013) was sequenced on Illumina HiSeq2000, yielding 877.7 Gb clean data for scaffolding. The final assembly presented a total length of 3,168 Mb for the scaffolds, with N50 of 27.8 kb and 176.2 kb for contigs and scaffolds, respectively. BUSCO analysis indicates 90% of single-copy insect orthologs were complete. We also conducted BUSCO analysis towards the transcriptomes of *A. jianchuanensis* (NCBI:txid92022), and a completeness of 96.6% was observed. Genome and transcriptomes were subsequently employed in downstream analysis.

Evolutionary position of ancient Himalaya ghost moths based on phylogenomics analysis

We carried out a phylogenomics analysis based on genomes and transcriptomes of three ghost moth species, together with 13 Lepidoptera and two outgroups (data source see Table S1). The separation of exoporian and ditrysian Lepidoptera occurred by the end of the Triassic Period, around 205 million years ago. While the speciation of the *Thitarodes* moths soon followed *A. jianchuanensis*, approximately 26 million years ago by the end of the Paleogene Period. Although *Ahamus* and *Thitarodes* represented an ancient moth lineage, the species within were diverged in parallel with higher moths (Figure 1A). We next asked what olfactory traits were maintained during the evolution of Himalaya ghost moths.

Nonsexual dimorphic shortened antennae and enlarged glomeruli of Himalaya ghost moths

The Himalaya ghost moths had no observable proboscis but possessed intact antennae and labial palps (Figure 1B). Additionally, we found that these moths had the shortest antennae among 32 lepidopteran families [35] (Figure S2) and their antennae were dominated by sensilla trichoidae (Figure 1C, Figure S3).

The antennal lobe morphological atlas showed that three ghost moth species overall presented significantly less glomeruli (23 to 37), compared with other moths (45 to 80, Figure 1D, Data S1). Amongst 96 tested brains, the ordinary glomeruli arrangements in Himalaya ghost moths were distinguishable from those in the compared species. The families of Hepialidae, Pieridae, and Plutellidae had less intra-species variations in glomerular arrangements compared to the other four higher moth families (Figure S4). The MGC consisted of 2 to 3 glomeruli in Himalaya ghost moths, and identical areas were confirmed in all tested species. Female LFGs was distinguishable in earlier species including the Himalaya ghost moths and the diamondback moth *Plutella xylostella*, comparing with the later species (Figure 1D).

Volume proportions of the MGC and LFG glomeruli across tested species were compared. It showed that Himalaya ghost moths had both the largest MGCs and LFGs (Figure S5). The cumulus which represents a major area, occupied 23.8 ± 5.5% of the antennal lobes in *A. jianchuanensis*, 15.9 ± 2.0% in *T. armoricanus* and 19.5 ± 3.0% in *T. xiaojinensis*, respectively (Figure S5, Data S1). The other species had relatively smaller MGC glomeruli in volumes, e.g., on average 9.8 + 0.7% of the cumulus occupation for Noctuidae. As for females, LFG1 in three ghost moth species were significantly larger than those within higher moths (Figure S5, Data S1). We checked the MGCs of 16 species in terms of volumes and shapes by utilizing a principal component analysis

test, covering 73.8% for explained variances. Twelve of 16 lepidopteran species had a similar trend in MGC organizations, but the Himalaya ghost moths and *H. cunea* exhibited separated patterns, and especially *T. xiaojinensis* was totally isolated from higher Lepidoptera (Figure S6). We wonder if these structural specificities may reflect the genomic backgrounds and receptor repertoires in the Himalaya ghost moths.

A unique large OR array on the ghost moth T. xiaojinensis *chromosome*

A total of 23 *TxiaOrs* were confirmed to be expressed in the antennae of *T. xiaojinensis* via RT-PCR verification, out of annotations from genome and antennal transcriptome assembly (Figure S7, Data S2). This number was higher than those found in *A. jianchuanensis* (10) and *T. armoricanus* (16) (Table S2). Notably, a large array comprising 34 tandem duplications was mapped by higher moth PRs on chr14 of the chromosome-level assembly of *T. xiaojinensis*. This array, homologous to *TxiaOr19*, contained 16 homologs (*TxiatdOrs*) and 18 pseudogenes (*TxiatdpOrs*), which maintained the largest tandem duplications reported in lepidopterans (Figure S8, Data S2). The *TxiaOr19* array was located on the same chromosome with an upstream *TxiaOr18c*, which mapped to Noctuidae homologs [36]. Maximum-Likelihood phylogeny analysis using 387 ORs showed that the *TxiaOr19* array joint an earlier group where canonical type I PRs arose (Figure 2A, Data S3). The earlier separation of canonical PRs and TxiaOR19 tandem was also observed when cross-checked with Neighbor-Joining and Bayes method (Figure S9). A female-biased expression pattern was observed in *TxiatdOr15* and *TxiatdOr25* of the 16 *TxiaOr19* homologs (Figure S10). Differing from ORs found in evolutionarily later moths, the TxiaOR19 array could blast to ORs in locusts, aphids, soldier flies, mosquitoes, and fleas, with homologues predicted by CLAN [37] (Figure 2A and Figure S9, S11). However, the male-biased TxiaOR7 failed

to blast to any ORs from those earlier species (Figure S11, S12).

We investigated the evolution of the *TxiaOr19* array by mapping it to chromosomes of caddisflies, primitive and higher moths, and cross-checking with canonical PR mapped regions (Figure 2B). The results indicated that tandem ORs were identified in linearized regions of almost all species, except for *C. flavipennella* and *B. mori*. Some of these linearized regions did not contain ORs that could be annotated using FGENESH [38]. Mapped ORs from linearization analysis and nr blast ORs by TxiaOR19 tandem were used in a Bayesian phylogenic analysis, and it showed that canonical PRs and a single LarmPR1 in the caddisfly formed a clade in the phylogeny (Figure 2A, Figure S13), and expansions of PR tandems were consistently observed within species after the Himalaya ghost moths, except for *C. flavipennella* (Figure 2B, Figure S13). Notably, regions containing *TxiaOr19* and PR had mixed tandem patterns in the early moths succeeding *T. xiaojinensis* but tended to be separated in later species (Figure 2B). Bayesian phylogenetic analysis showed that the homologs of *TxiaOr19* underwent significant diversification from their ancestors (Figure S13). Specifically, *TxiaOr19* array formed the same clade with *LarmOr19-Or13a* tandem (Figure 2A, Figure S13). The majority of PR-mapped ORs formed a single cluster that possibly diverged from LarmPR1, with a few remaining in the TxiaOR19 mapped phyla (Figure 2A, Figure S13).

Using MEME suite, we found that LarmPR1 exhibited all three signature motifs of PR consensus regions as reported before [12], indicating the potential emergence of canonical PRs prior to the evolution of lepidopteran insects (Figure 2C). However, most PR-mapped ORs from non-Ditrysia primitive moths did not meet the requirements of canonical PR motifs (Figure 2C). On the other hand, the majority of TxiaOR19-mapped ORs had two motifs, with some having three motifs (named as motif 4-6) upon checking with the same approach (Figure 2D). Notably, motifs 4-6 from

TxiaOR19 homologs showed overlaps with motifs 2-3 from PR homologs, suggesting the existence of a common ancestor for TxiaOR19 and canonical PRs at an earlier evolutionary stage (Figure 2E).

Transposable elements (TE) *involved in the evolution of ORs*

Tandem gene duplications and chromosome linearization patterns have been reported to be associated with TE activities [39]. To explore how a large and specific OR array was formed in the ghost moth *T. xiaojinensis*, we characterized the landscape of TEs in the genomes of above species. It showed that 2-3 TE burst events occurred in the Himalaya ghost moths. These bursts likely took place around the same time as the successive divergences of Exoporia and *Hepialus* (Figure 3A, Figure 1A). Caddisfly, primitive and modern Lepidoptera experienced more recent bursts of TE activity compared to Himalaya ghost moths (Figure 3A, Figure S14). Specifically, various TE arrangements were observed in previously identified OR loci. The TE arrangements of the *TxiaOr19* tandem were correlated with that of the *LarmOr13a* (Figure S15). Furthermore, the TE landscapes in the *TxiaOr18/19* homologs were found to be similar mostly with *Ors* from non-ditrysian species and later diversified in higher moths (Figure S15).

In conclusion, our findings suggest that both the TxiaOR19 tandem and PR clusters had already emerged in caddisflies. The OR19 lineage underwent expansion within the ancestral moth lineage, leading to the formation of a large duplicated tandem in *T. xiaojinensis*. Furthermore, linkages between OR19 and PRs were observed in species predating Ditrysia. In later higher moths, PRs became predominant, while the OR19 cluster contracted through asymmetric diversifications of their homologs, as supported by the similar TE arrangements (Figure 3B).

Equal sex roles of ghost moth T. xiaojinensis *adults*

The replacement of the TxiaOR19 duplications with canonical PRs suggests possible different tuning characteristics of TxiaOR19 from a PR that narrowly tunes to sex pheromone components. To confirm this assumption, we first analyzed the emissions of adult *T. xiaojinensis* using both solvent extraction and solid-phase microextraction (SPME) methods. We found that male and female adults were not distinguishable by tracing the volatile blends in abdomen tip extractions. However, SPME samples collected within the first 24 hours after female emergence exhibited a significant peak corresponding to oleamide (Figure S16A). We performed successive docking simulations using TxiaOR19 array and four sex-biased TxiaORs against the identified major components. The results showed that the TxiaOR19 array had less binding affinity towards the panel of ghost moth emissions, and the responding spectrum was relatively broad for all 16 tandem ORs (Figure S16B).

Considering ghost moths, including *T. xiaojinensis*, lack sexual dimorphism in their antennal lobe (Figure 1D), we hypothesized that *T. xiaojinensis* may also exhibit unconventional calling and mating behaviors compared to higher moths. We then tested adult pairs of *T. xiaojinensis* in a courtship arena (Figure 4A). Unlike the female calling behaviors of higher moths with wing beats and extruded pheromone gland, calling behavior of this ghost moth involved hovering with wing beats. Females fluttered with substantial wing beats, while males fluttered by small range vibrating-like wing beats (Figure 4B). Interestingly, male and female adults exhibited similar amounts of calling behaviors and tracing velocities (Figure 4B and C). Our results indicated that the sex roles of *T. xiaojinensis* adults differed from those of higher lepidopterans during mating allocation, which may be attributed to the predicted unconventional TxiaOR19 tandem, non-canonical female emission, and olfactory architectural observations.

Discussion

Himalaya ghost moths offer a basal model for the study of olfaction evolution in Lepidoptera due to their unique olfactory system, limited distribution, and possession of the largest genomes in Lepidoptera. Our study has successfully generated the first chromosome-level assembly for this lineage, providing valuable insights into the genetic characteristics of these ghost moths. We have discovered that the OR evolutionary pathway in ghost moth *T. xiaojinensis* parallels with that of modern moths, and have identified molecular traces that reveal the origins of the modern pheromone sensory system. Interestingly, the presence of unique olfactory structures including enlarged glomeruli observed in both sexes and dominated long trichoid sensilla, mostly non-biased expressions of *TxiaOr19* homologs, along with their non-feeding life traits in adulthood suggests that these ghost moths may employ a primitive pheromone sensing system to locate potential partners.

Why the lineage of Himalaya ghost moths keeps primitive may be due to their isolated habitats, uneven long life cycle for larvae (3-6 years in nature) and a brief adult stage (several days) [26,27], which greatly reduces the evolution speed of this lineage. They have developed a unique evolutionary strategy of focusing solely on reproduction in adulthood while forgiving foraging [40]. The redundancy of their giant genomes is an example of the basal genomic features possessed by the ghost moths [41]. Asymmetrically diverged duplications and frequent TE activity bursts have played a critical role in both PR formation and the emergence of other functional genes in Lepidoptera [23]. As a result, the expanded OR19 homologs in primitive species were later diversified into canonical PRs, establishing the advanced sex pheromone-based communication system. One interesting result in our molecular docking predictions is that the male-biased OR7

shows a higher affinity to oleamide, suggesting that TxiaOR7 potentially serves as an ancestral PR of the novel PR clade in lepidopteran species. However, more experimental functional evidence needs to be provided for both TxiaOR19 array and TxiaOR7 to support their evolutionary roles in lepidopteran species.

Canonical PRs of ancient moth species were broadly characterized [13,43]. In this study, we showed that the TxiaOR19 array and LarmPR1 share motifs. These motifs were likely to be already separated before Lepidoptera and Trichoptera diverged from each other. Considering that motif shifts accompany similar transposable element arrangements within the tested OR loci, it is likely that the first PR emerged from exonization, driven by TE activities. This effect has been commonly observed in other organisms [44]. On the other hand, PRs shared motif regions with TxiaOR19 and the later could be blasted to ORs in dipteran species where large OR duplications existed such as in *Bactrocera dorsalis* [45]. The disappearance of large tandem arrays in later lepidopterans suggests the separation of ancestral duplicated ORs. This can be supported by scattered chromosome linearization and increased DNA transposons during TE activity bursts. The duplicated ORs themselves could reflect rapid olfactory evolution for species adaptation [46]. Although TEs may not be determining factors for the functional emergence of ORs, as shown in the clonal raider ant *Ooceraea biroi* [47], we cannot exclude possible TE involvement in PR emergence due to the horizontally diverged TE landscapes among lepidopteran ORs.

Evolutionary later moths show larger interspecies variations and increased numbers of glomeruli in antennal lobes, suggesting potential positive selection in olfactory systems, along with their distribution in different ecological niches [48]. The enlarged MGCs in the butterflies, such as *Pieris rapae* and *Godyris zavaleta* [49], suggest that the sexual dimorphic brain organizations are widely presented by Lepidoptera, however, the link between morphology and pheromone

recognition in butterflies requires further examination. On the contrary, the sexual dimorphic olfactory neuropils are not suitable for Himalaya ghost moths, as females have also retained the enlarged LFGs. We argue that female LFGs are more likely involved in mating allocation rather than egg-laying orientation since these ghost moths spray eggs in nature, unlike the majority of modern moth egg-laying behavior in the selected locations [40,50]. Therefore, they may not require a sophisticated olfactory system for precise assessment of corresponding sites. This is also supported by our behavioral assays in the *T. xia* that both male and female were moving to find partners. Given their ecological traits, highly redundant genome, and a smaller number of ORs, we believe that these Himalayan ghost moths may retain pheromone sensing abilities and exhibit primitive sex roles within Lepidoptera. Further investigation into the function of ORs remains crucial for refining this perspective.

Himalaya ghost moths retain ancestral traits of olfactory system for equal sex roles, which may be attributed to the mechanisms of asymmetric divergence and redundant genome formation. The lack of sexual dimorphism in the antennal lobes and expansion of TxiaOR19 array other than canonical PRs also contribute to the non-biased sex role differentiation within this primitive lineage. Overall, these findings highlight the unique evolutionary features of Himalaya ghost moths and shed light on the mechanisms shaping olfactory systems in insects.

Materials and Methods

Insects

Newly emerged lepidopteran species from lab colonies were sexed, and 3 to 5-day-old adults were used in all tests. *A. jianchuanensis, T. armoricanus, T. xiaojinensis, Agrotis ipsilon, S.*

frugiperda, *Galleria mellonella*, and *P. rapae* were obtained from Institute of Zoology, Guangdong Academy of Sciences. *H. cunea* was obtained from Chinese Academy of Forestry. *Athetis dissimilis* was obtained from Henan University of Science and Technology. *Helicoverpa armigera* and *Helicoverpa assulta* were obtained from Henan Agricultural University. *Mythimna separata*, *C. pomonella*, and *S. litura* were obtained from Institute of Plant Protection, Chinese Academy of Agricultural Sciences. *S. exigua* were obtained from Qingdao Agricultural University. *P. xylostella* were obtained from Shanxi Agricultural University. All lab colonies were regularly rejuvenated with natural populations.

Morphometric measurement

A total of 7 strains of Himalaya ghost moths were measured for the lengths of antennae and forewings. Three *T. xiaojinensis* strains were from lab colony mentioned above, and Xiaojin (N30.99, E102.27), Hongkou (N31.16, E103.84) field populations. The other four strains consisted of two *A. jianchuanensis* populations collected from Jiulong (N28.99, E101.51), Gongga (N29.56, E101.98), and two *T. armoricanus* populations from Yala (N30.11, E102.25), Kangding (N30.08, E101.97), respectively. Intact appendages were removed and embedded on glass slides before being processed under an AXIO Imager microscope (Zeiss, Jena, Germany) equipped with an Axiocam 512 camera (Zeiss). A ZEN 2.3 software (Zeiss) was used to acquire scale bar labelled photographs of antennae and wings. Lengths of interest were manually assigned to the scale bar using ImageJ 1.53f51 (National Institute of Health, USA) and then recorded. A total 5 to 21 replicates were carried out for each strain, and means were used to develop olfactory indexes using the formula [antenna/wing]. Data from other species were referred to the previous publication [35].

Scanning electron microscopy

The antennae of 1-3 d adults were cut from the base and fixed in 0.25% glutaraldehyde at 4

°C overnight. After three washes at room temperature with 0.1 M phosphate-buffered saline (PBS, pH 7.4) for 15 min each, the antennae were dehydrated through a laddered ethanol series (30, 50, 70, 80, 90, and 100%) for 15 min each and dried for 15 min in a critical point drier (Bal-Tel CPD 030) before being mounted on aluminum stubs. The mounted antennae were coated with gold spray (Bal-Tel SCD 005) and observed with an SEM instrument (FEI Quanta 200).

Antennal lobe atlas

Lepidopteran brains were labeled according to previous work [21]. Newly dissected intact brains were successively processed with 4% paraformaldehyde in 0.1 M PBS for fixation (24 h), pre-incubating with 5% normal goat serum in 0.1 M PBS containing 0.5% Triton X-100 (NGS-PBST) (0.5 h), incubating with 1% SYNORF1 (Developmental Studies Hybridoma Bank, University of Iowa) in 5% NGS-PBST (72 h), and incubating with Alexa Fluor 488 goat anti-mouse (Invitrogen, Eugene, OR, USA) at 1:500 with 1% NGS-PBST (48 h). After being rinsed six times in PBS and dehydrated with a laddered ethanol series, brain samples were mounted with antifade mounting medium (Beyotime, Shanghai, China) in perforated aluminum slides which was sandwiched by two glass coverslips. Three brains of each sex from each species were prepared for imaging.

All image stacks were acquired with a confocal laser scanning microscopy system with a 10-20× objective. Data for *A. jianchuanensis*, *T. armoricanus*, *T. xiaojinensis*, *G. mellonella*, and *A. ipsilon* were collected with FV3000 (Olympus, Tokyo, Japan). Data for *H. cunea*, *A. dissimilis*, *H. armigera*, *H. assulta*, *M. separata*, *S. litura*, and *P. xylostella* were collected with LSM 780 (Zeiss). Data for *S. frugiperda*, *P. rapae*, *C. pomonella*, and *S. exigua* were collected with A1 HD25 (Nikon, Tokyo, Japan). An argon laser at 488 nm was used to excite the Alexa Fluor. The resolution of the x-axis was set to 500-2,048 voxels and the section interval was set to 3 or 5 μm. Amira software

(AMIRA 5.3, Visage Imaging, Fürth, Germany) was used as previously described to conduct segmentation, tissue statistics, and three dimensional reconstructions of the antennal lobes [21].

Genome and transcriptome sequencing

Genomic DNA of *T. xiaojinensis* larva was extracted for library establishment, and then sequenced with Nanopore PromethION platform (Oxford Nanopore Technology, Oxford, UK). After quality control, a total 319.9 Gb clean data was assembled by using NextDenovo (RRID:SCR_025033). Meanwhile, short-reads library was constructed by Illumina platform with the same batch of *T. xiaojinensis* DNA, and 165 Gb raw data were generated. After filtering, the remaining clean reads with Q > 20 were used for minimap2 mapping onto the genome assembly which was later polished by NextPolish (RRID:SCR_025232). To remove the DNA contamination from the other organisms, the polished genome was aligned against the NCBI nucleotide (NT) database, and the contigs which were aligned to the sequences from fungi, plants, or virus were removed.

To obtain a chromosome-level assembly, Hi-C scaffolding was further carried out with the same larval sample following reported protocols [51-53]. Specifically, samples were fixed using 2% formaldehyde to establish cross-links, followed by cell lysis and sample quality assessment through extraction. Chromatin digestion was carried out using a restriction endonuclease, with enzyme cleavage efficacy evaluated through sampling. Subsequent steps included biotin-14-dCTP (Invitrogen) labeling, blunt-end ligation, DNA purification, and Hi-C sample preparation. After passing quality control, Hi-C fragments underwent end-biotin removal, sonication, end repair, A-tailing, and adapter ligation to form ligated products. Subsequent PCR steps were amplified to generate library enriched products. Library amplification products were sampled for Hi-C fragment junction quality control, and the entire library preparation was sequenced using Illumina HiSeq

with a PE150 sequencing strategy (NextOmics Biotech. lnc., Wuhan, China). The fastp v.0.12.6 (RRID:SCR_016962) with default parameters was used to filter the raw sequences, resulting in high-quality clean reads. The sequenced Reads1 and Reads2 were separately aligned to the assembled genome sequence using bowtie2 v.2.3.2 (end-to-end alignment mode, parameters: --very-sensitive -L 30) (RRID:SCR_016368) to obtain the alignment information. For the unmapped reads after alignment, we searched for reads containing ligation junction sites, trimmed them, and performed alignment again. Finally, the alignment results were combined, and the proportion of Unique Mapped Paired-end Reads was calculated. The LACHESIS software (RRID:SCR_017644) was used to cluster the Contig sequences of the draft assembly into chromosome groups using agglomerative hierarchical clustering. The final genome was further assessed with BUSCO [54] for completeness.

Genome of *T. armoricanus* was obtained from the DNA of a fourth instar larva without gut. A total of 23 different insert size libraries were constructed and 67 lanes were sequenced on Illumina HiSeq2000 platform (RRID:SCR_020130), resulting in 1,344.5 Gb raw data and 877.7 Gb filtered data. The genome was assembled using SOAPdenovo (RRID:SCR_010752) (v2.04) [55] and SSPACE (RRID:SCR_005056) (v2.0) [56] software. We used all 549.3 Gb (180.4×) clean data of short insert size libraries to construct contigs and all 877.7 Gb (266.4×) clean data to construct scaffolds. 283.4 Gb (86.0×) data of large insert size libraries was used again to construct scaffolds by using SSPACE. Then all clean data of short insert size libraries was used to fill the gaps. TrimDup3 (Rabbit2.6) [57] was used to remove the large redundant sequences. RNA-seq data from 14 different developmental stages of *T. armoricanus* was assembled by Trinity (RRID:SCR_013048) v2.4.0 [58] and was mapped to the assembled genome sequence using BLAT (RRID:SCR_011919) (v. 34) [59], to check the coverage rate. The results showed that 96.8% of the

sequences could be mapped to the assembly.

Respective antennae, heads, and labial palps from *A. jianchuanensis* and *T. xiaojinensis* were collected in liquid nitrogen and sequenced with Illumina according to the manufactural instructions. The transcriptomes were assembled by Trinity v2.4.0 [58] with default parameters.

Phylogenetic analysis and estimation of divergence time

To reconstruct the phylogenetic tree of 16 lepidopteran insect species with two outgroups of *Tribolium castaneum* and *D. melanogaster*, we first downloaded the genome annotations or raw data of transcriptomes for other 15 species from NCBI (Table S1). The transcripts were assembled by Trinity v2.4.0 [58] with default parameters. Subsequently, the orthologs of these 18 insect species were inferred from their genomic or transcriptomic protein annotations by using OrthoFinder (RRID:SCR_017118) [60] with the default parameters. Single-copy orthologues from each species were selected for phylogenetic reconstruction. The protein sequences of each orthologue were independently aligned with MAFFT (RRID:SCR_011811) v7.407 [61], and the aligned results were trimmed by trimAl (RRID:SCR_017334) [62] to remove low-quality regions with the parameter "-automated1", the trimmed sequences were concatenated into a single super sequence. RAxML (RRID:SCR_006086) [63] was then used with the VT + F model, which is inferred by ProtTest (RRID:SCR_014628) v3.4.2 [64], to estimate a maximum likelihood tree starting with 1000 bootstraps followed by likelihood optimization.

We used r8s (RRID:SCR_021161) (V1.7.1) [65] to estimate the divergence time. The phylogenetic tree constructed by RAxML [63] was used as an input tree. A smoothing parameter of 3 was selected, which was estimated by the cross-validation approach (with parameters "cvstart=0, cvinc=1, cvnum=18"). The calibration points were: 1) the most recent common ancestor of the clade including *T. castaneum* and *P. xylostella*, constrained to be 337 Mya (million years ago); 2)

the most recent common ancestor of the clade including *D. melanogaster* and *C. pomonella*, constrained to be 318 Mya; and 3) the most recent common ancestor of the clade including *P. rapae* and *S. litura*, constrained to be 125 Mya [42].

Annotation of Or *gene family*

The protein sequences of lepidopteran insect ORs were collected from NCBI. These protein sequences were then used as queries in iterative TBLASTN searches with the parameter "-evalue 1e-5" against the assembly of the three ghost moth species to find candidate *Or* genes. A local command line HMMER (RRID:SCR_005305) (version 3.1b2) [66] search was conducted for these candidate ORs against the Pfam-A database (RRID:SCR_004726) to find the 7tm_6 (PF02949) or 7tm_4 (PF13853) HMM profiles for ORs. FGENESH 2.6 [38] prediction of potential genes was performed for contigs of interests. Data from other species were collected according to the reported works (Table S1).

Characterizations of Ors

CDS cloning verifications were carried out targeting on annotated *TxiaOrs* using adult antennal cDNA. Gene-specific primers were designed (Table S3) and PCRs were done on a Veriti 96-well thermal cycler (Applied Biosystems, MA, USA) using High Fidelity (HiFi) PCR SuperMix (Trans, Beijing, China). Products were processed with 1% agarose (BBI, Shanghai, China) on a PowerPac electrophoresis system (Bio-Rad, CA, USA) and visualized with a GelDoc-It TS3315 imaging system (UVP, CA, USA). Multiple bands such as for *TxiaOr18* were separately collected and purified with a gel extraction kit (GenStar, Beijing, China) before Sanger sequencing (Sangon Biotech, Shanghai, China). Later analysis was based on the longest sequenced *TxiaOrs* for each locus. *Or* expressions were showed as autoscaled heatmaps indicating the FPKM (Fragments Per Kilobase of transcript per Million mapped reads) which were calculated by RSEM [67] from head,

antenna, and labial palp transcriptomes of adult ghost moths.

Phylogenetic analysis of 387 ORs was carried out with the above mentioned protocol using MAFFT [61], trimAl [62], and IQ-TREE (RRID:SCR_017254) [68] using 'Auto' option for model, with 1000 ultrafast [69] bootstraps, as well as the Shimodaira-Hasegawa-like approximate likelihood-ratio test [70]. Verifications were done to the tree topology with MEGA X [71] and MrBayes (RRID:SCR_012067) 3.2.6 [72] to establish the NJ tree based on Dayhoff model and BY tree based on Blosum62 model, respectively. Homologs of TxiaOR19 array were predicted using CLANS [37] using the blastx results against the NCBI nr database. For chromosome linearization tests, local tblastn was applied to map the selected ORs towards chromosomes of each species (Table S1) and results were visualized as circos plots by using TBtools (RRID:SCR_023018) v1.113 [73]. Evolution of mapped ORs were inferred using MrBayes 3.2.6 [72] under JTT+F+G4 model (2 parallel runs, 200,000 generations), in which the initial 25% of sampled data were discarded as burn-in. The final average standard deviation of split frequencies was 0.069772. Protein motifs were predicted using MEME Suite (RRID:SCR_001783) v5.5.2 [74].

Annotation of repeats and transposable element families

For transposable element analysis, we first performed the *de novo* predictions for each species by RepeatModeler (RRID:SCR_015027) version open-1.0.11 to generate a specific library. Then we annotated the genome assembly by RepeatMasker (RRID:SCR_012954) version open-4.0.7 with the "ncbi" search algorithm. Annotated transposable element sequences were manually verified and classified with Dfam (RRID:SCR_021168) [75]. The calcDivergenceFromAlign.pl and createRepeatLandscape.pl scripts in the RepeatMasker package were used to calculate the Kimura divergence values and plot the repeat landscape, respectively. Estimations for transposable element burst times were based on the recently reported substitution rate of 6.19×10^{-10} per site per

generation in arthropods [76].

Chemical analysis

Hexane extraction method was adopted from our previous works on moth pheromone identifications [77]. Abdomen tips of calling adult *T. xiaojinensis* were cut with dissection scissors and immediately put in 20 µl hexane (HPLC purity, Kermel Chemical Reagent Co., Tianjin, China) which was kept at 4 °C for 1 d prior to the test. Head space SPME method was adopted from our previous works on body surface volatile emissions of insects [78]. Newly emerged male or female adults were kept in a mesh cage in separated rearing chambers for sampling. A 50/30 µm DVB/CAR/PDMS stableflex fiber (Supelco, Bellefonte, PA, USA) was penetrated into the cage for sampling at 10 °C for 24 h. The volatile blends sampled were either injected for 1 µl or subjected to an Agilent 7890B GC - 5977 MSD coupled system equipped with a HP-5MS column (0.25 µm x 30 m x 0.250 mm) (Agilent, Palo Alto, CA, USA). A 60 min oven temperature program was used following: 40 °C for 2 min, 40 °C to 150 °C at 5 °C/min, 150 °C for 2 min, 150 °C to 200 °C at 10 °C/min, 200 °C for 5 min, 200 °C to 230 °C at 5 °C/min, and 230 °C for 18 min. Raw data were analyzed with MSD ChemStation (G1701FA F. 01. 03. 2357) by searching against a NIST 17 MS library (Agilent). A total 40 individuals were tested for SPME from two stratified groups. Each hexane extraction sample included 20 individuals and at least 3 replicates were done towards each sex.

Docking simulation

TxiaOR19 and the other four sex biased OR sequences of *T. xiaojinensis* were predicted by AlphaFold2 [79] for their tertiary structures. The 3D structures of 18 ligands were downloaded from PubChem [80]. The Molecular Operating Environment software (MOE, Chemical Computing Group ULC, Montreal, Canada) was used to dock the ligands with ORs. Briefly, ORs were

prepared using MOE QuickPrep and ligands were energy minimized with the MOE Energy Minimize prior to the simulation. Triangle Matcher algorithm was selected for placement and 30 top-scoring placement poses were selected by the London dG empirical scoring function, while the rigid receptor was selected for refinement and top-scoring poses were selected by the GBVI/WSA dG empirical scoring function. The binding free energy of respective OR-ligand was estimated by using S Score function and later used for establishment of colour coded map.

Courtship arena

The assays were carried out using 1 d emerged naïve moths at peak mating hours 18 - 20 pm during sunset. One randomly chosen pair of *T. xiaojinensis* adults was placed in a paper funnel and recorded for 1 h. A total of 20 pairs were tested and recorded for calling and tracing behaviors. Recorded footages were processed through the idTracker [81] pipeline to obtain the velocities of moths shown as per pixel distances per min. Fluttering behaviors were observed by manually checking each video file.

Statistics and data processing

Comparison of means was done by using either unpaired *t* test or GLM followed by multiple comparisons according to treatment sizes (SPSS 22.0.0.0, IBM Corp., Armonk, NY, USA). Simple linear regression and data plotting were done using Prism 5.01 (GraphPad software, San Diego, CA, USA). Multivariate tests were carried out with MetaboAnalyst (RRID:SCR_015539) 5.0 [82] server which integrates R statistics (RRID:SCR_001905). All error bars indicate standard errors of the means otherwise indicated in the figure legends.

Author contributions

Rui Tang: Conceptualization, Methodology, Data curation, Formal analysis, Investigation, Visualization, Writing-original draft, Writing-review & editing. **Cong Huang**: Methodology, Data curation, Formal analysis, Visualization, Writing-review & editing. **Jun Yang**: Data curation, Writing-review & editing. **Zhong-Chen Rao**: Methodology, Formal analysis, Writing-review & editing. **Li Cao**: Data curation, Writing-review & editing. **Peng-Hua Bai**: Data curation, Writing-review & editing. **Xin-Cheng Zhao**: Methodology, Data curation, Writing-review & editing. **Jun-Feng Dong**: Data curation, Writing-review & editing. **Xi-Zhong Yan**: Data curation, Writing-review & editing. **Fang-Hao Wan**: Formal analysis, Writing-review & editing. **Nan-Ji Jiang**: Conceptualization, Methodology, Data curation, Investigation, Writing-original draft, Writing-review & editing. **Ri-Chou Han**: Conceptualization, Investigation, Supervision, Writing-review & editing.

Declaration of Interest

The authors declare no competing interests.

Acknowledgments

We thank Dr. Zhang Bin and Dr. Meng Xiang for supporting on insect materials. We thank Min-Jun Huang for providing technical support on bioinformatics. We thank Zhongkai University of Agriculture and Engineering, South China Normal University, and Institute of Zoology, CAS for sharing the research platforms to assist confocal imaging. We thank B.F.A. Yorda for the development of insect schematics.

Funding information

The current research was funded by the Major Science and Technology Project of Qinghai Province (No. 2021-SF-A4-1), National Key R & D Program of China (2023YFC2606900), GDAS Special Project of Science and Technology Development (2022GDASZH-2022010106), and Guangdong Basic and Applied Basic Research Foundation (No. 2020A1515011366).

Data availability

The whole genome sequence data of *T. xiaojinensis* reported in this paper have been deposited in NCBI (Bioproject: PRJNA1006505). All additional supporting data are available in the *GigaScience* repository, GigaDB [83].

References

1. Shine R. Ecological causes for the evolution of sexual dimorphism: a review of the evidence. Q Rev Biol. 1989; 64: 419-461
2. Ritchie MG. Sexual selection and speciation. Annu Rev Ecol Evol Syst. 2007; 38: 79-102
3. Andersson M, Iwasa Y. Sexual selection. Trends Ecol Evol. 1996; 11: 53-58
4. Kokko H, Jennions MD. Parental investment, sexual selection and sex ratios. J Evol Biol. 2008; 21: 919-948
5. Datta SR, Vasconcelos ML, Ruta V, Luo S, Wong A, Demir E, et al. The *Drosophila* pheromone cVA activates a sexually dimorphic neural circuit. Nature. 2008; 452: 473-477
6. Allen CE, Zwaan BJ, Brakefield PM. Evolution of sexual dimorphism in the Lepidoptera. Annu Rev Entomol. 2011; 56: 445-464
7. Butenandt A. Uber den sexsual-lockstoff des seidenspinners *Bombyx mori*. Reindarstellung und konstitution. 1959; Z Naturforschg 14b: 283-284 (1959).

8. Sakurai T, Namiki S, Kanzaki R. Molecular and neural mechanisms of sex pheromone reception and processing in the silkmoth *Bombyx mori*. Front Physiol. 2014; 5: 125
9. Stork NE. How many species of insects and other terrestrial arthropods are there on Earth? Annu Rev Entomol. 2018; 63: 31-45
10. Löfstedt C, Wahlberg N, Millar J. Evolutionary patterns of pheromone diversity in Lepidoptera. In: Allison JD, CardŽ RT, editors. Pheromone communication in moths: evolution, behavior and application. Oakland: University of California Press; 2016. p. 43-82
11. Ando T, Inomata SI, Yamamoto M. Lepidopteran sex pheromones. In: Schulz S, editor. The chemistry of pheromones and other semiochemicals I. Berlin: Springer; 2004. p. 51-96
12. Zhang DD, Löfstedt C. Moth pheromone receptors: gene sequences, function, and evolution. Front Ecol Evol. 2015; 3: 105
13. Yuvaraj JK, Andersson M N, Corcoran JA, Anderbrant O, Löfstedt C. Functional characterization of odorant receptors from *Lampronia capitella* suggests a non-ditrysian origin of the lepidopteran pheromone receptor clade. Insect Biochem Mol Biol. 2018; 100: 39-47
14. Bastin-Héline L, De Fouchier A, Cao S, Koutroumpa F, Caballero-Vidal G, Robakiewicz S, et al. A novel lineage of candidate pheromone receptors for sex communication in moths. eLife. 2019; 8: e49826
15. Hildebrand JG, Shepherd GM. Mechanisms of olfactory discrimination: converging evidence for common principles across phyla. Annu Rev Neurosci. 1997; 20: 595-631
16. Zhang J, Walker WB, Wang G. Pheromone reception in moths: from molecules to behaviors. Prog Mol Biol Transl Sci. 2015; 130: 109-128
17. Matsumoto S, Hildebrand JG. Olfactory interneurons in the moth *Manduca sexta*: Response characteristics and morphology of central neurons in the antennal lobes. Proc R Soc Lond B Biol Sci. 1981; 213: 249-277
18. Trona F, Anfora G, Bengtsson M, Witzgall P, Ignell R. Coding and interaction of sex pheromone and plant volatile signals in the antennal lobe of the codling moth *Cydia pomonella*. J Exp Biol. 2010; 213, 24: 4291-4303

19. Namiki S, Iwabuchi S, Kanzaki R. Representation of a mixture of pheromone and host plant odor by antennal lobe projection neurons of the silkmoth *Bombyx mori*. J Comp Physiol A. 2008; 194: 501-515

20. Rössler W, Tolbert LP, Hildebrand JG. Early formation of sexually dimorphic glomeruli in the developing olfactory lobe of the brain of the moth *Manduca sexta*. J Comp Neurol. 1998; 396: 415-428

21. Zhao XC, Ma BW, Berg BG, Xie GY, Tang QB, Guo XR. A global-wide search for sexual dimorphism of glomeruli in the antennal lobe of female and male *Helicoverpa armigera*. Sci Rep. 2016; 6: 1-9

22. Mallet J. Sex roles in the ghost moth *Hepialus humuli* (L.) and a review of mating in the Hepialidae (Lepidoptera). Zool J Linn Soc. 1984; 80: 67-82

23. Holland PW, Marlétaz F, Maeso I, Dunwell TL, Paps J. New genes from old: asymmetric divergence of gene duplicates and the evolution of development. Philos Trans R Soc Lond B Biol Sci. 2017; 372: 20150480

24. Han R, Wu H, Tao H, Qiu X, Liu G, Rao Z, et al. Research on Chinese cordyceps during the past 70 years in China. Chin J Appl Entomol. 2019; 56: 849-883

25. Wang Z, Pierce NE. Fine - scale genome - wide signature of Pleistocene glaciation in *Thitarodes* moths (Lepidoptera: Hepialidae), host of *Ophiocordyceps* fungus in the Hengduan Mountains. Mol Ecol. 2023; 32: 2695-2714

26. Wu H, Cao L, He M, Han R, De Clercq P. Interspecific hybridization and complete mitochondrial genome analysis of two ghost moth species. Insects. 2021; 12: 1046

27. Tao Z, Cao L, Zhang Y, Ye Y, Han R. Laboratory rearing of *Thitarodes armoricanus* and *Thitarodes jianchuanensis* (Lepidoptera: Hepialidae), hosts of the Chinese medicinal fungus *Ophiocordyceps sinensis* (Hypocreales: Ophiocordycipitaceae). J Econ Entomol. 2016; 109: 176-181

28. Kuenen LPS, Wagner DL, Wallner WE, Cardé RT. Female sex pheromone in *Korscheltellus gracilis* (Grote) (Lepidoptera: Hepialidae). Can Entomol. 1994; 126, 1: 31-41

29. Schulz S, Francke W, König WA, Schurig V, Mori K, Kittmann R, et al. Male pheromone of swift moth, *Hepialus hecta* L.(Lepidoptera: Hepialidae). J Chem Ecol. 1990; 16: 3511-3521

30. Chen X, Su X, Qiu Z, Xu Y, Yang Z, Hu P. Courtship and mating behavior of *Endoclita signifer* (Hepialidae: Lepidoptera) and the male sex pheromones in hairbrushes. J Econ Entomol. 2024; 117, 1: 218-229
31. Allan RA, Wang Q. Mating behaviour, and evidence for a female - released sex pheromone, in *Wiseana copularis* (Meyrick) (Lepidoptera: Hepialidae). N Z J Zool. 2001; 28, 3: 257-262
32. Kubo I, Matsumoto T, Wagner DL, Shoolery JN. Isolation and structure of hepialone; principal component from male sex scales of *Hepialus californicus* (Lepidoptera). Tetrahedron Lett. 1985; 26, 5: 563-566
33. Uchino K, Yamagiwa Y, Kamikawa T, Kubo I. Synthesis of hepialone; principal component from male sex scales of *Hepialus californicus* (Lepidoptera). Tetrahedron Lett. 1985; 26, 10: 1319-1320
34. Marukawa K, Mori K. Synthesis of (1R, 3S, 5S)-1, 3, 8-trimethyl-2, 9-dioxabicyclo [3.3. 1] non-7-ene, the male pheromone of a Hepialid Moth, *Endoclita excrescens*, and its enantiomer. European J Org Chem. 2002; 23: 3974-3978
35. Symonds MR, Johnson TL, Elgar MA. Pheromone production, male abundance, body size, and the evolution of elaborate antennae in moths. Ecol Evol. 2012; 2: 227-246
36. Brigaud I, Montagné N, Monsempes C, François MC, Jacquin−Joly E. Identification of an atypical insect olfactory receptor subtype highly conserved within noctuids. FEBS J. 2009; 276: 6537-6547
37. Frickey T, Lupas A. CLANS: a Java application for visualizing protein families based on pairwise similarity. Bioinformatics. 2004; 20: 3702-3704
38. Solovyev V, Kosarev P, Seledsov I, Vorobyev D. Automatic annotation of eukaryotic genes, pseudogenes and promoters. Genome Biol. 2006; 7: 1-12
39. Krasileva KV. The role of transposable elements and DNA damage repair mechanisms in gene duplications and gene fusions in plant genomes. Curr Opin Plant Biol. 2019; 48: 18-25
40. Nielsen ES, Robinson GS, Wagner DL. Ghost-moths of the world: a global inventory and bibliography of the Exoporia (Mnesarchaeoidea and Hepialoidea) (Lepidoptera). J Nat Hist. 2000; 34: 823-878
41. Cheng RL, Yu YX, Liu LX, Zhang CX, Fang CX. A draft genome of the ghost moth, *Thitarodes* (Hepialus) sp., a medicinal caterpillar fungus. Insect Sci. 2016; 23: 326-329

42. Wan F, Yin C, Tang R, Chen M, Wu Q, Huang C, et al. A chromosome-level genome assembly of *Cydia pomonella* provides insights into chemical ecology and insecticide resistance. Nat Commun. 2019; 10: 1-14
43. Yuvaraj JK, Corcoran JA, Andersson MN, Newcomb RD, Anderbrant O, Löfstedt C. Characterization of odorant receptors from a non-ditrysian moth, *Eriocrania semipurpurella* sheds light on the origin of sex pheromone receptors in Lepidoptera. Mol Biol Evol. 2017; 34: 2733-2746
44. Sela N, Kim E, Ast G. The role of transposable elements in the evolution of non-mammalian vertebrates and invertebrates. Genome Biol. 2010; 11: 1-13
45. Wang Y, Fang G, Xu P, Gao B, Liu X, Qi X, et al. Behavioral and genomic divergence between a generalist and a specialist fly. Cell Rep. 2022; 41: 111654
46. Nei M, Niimura Y, Nozawa M. The evolution of animal chemosensory receptor gene repertoires: roles of chance and necessity. Nat Rev Genet. 2008; 9: 951-963
47. McKenzie SK, Kronauer DJ. The genomic architecture and molecular evolution of ant odorant receptors. Genome Res. 2018; 28: 1757-1765
48. Engsontia P, Sangket U, Chotigeat W, Satasook C. Molecular evolution of the odorant and gustatory receptor genes in lepidopteran insects: implications for their adaptation and speciation. J Mol Evol. 2014; 79: 21-39
49. Montgomery SH, Ott SR. Brain composition in *Godyris zavaleta*, a diurnal butterfly, reflects an increased reliance on olfactory information. J Comp Neurol. 2015; 523: 869-891
50. Renwick J, Chew F. Oviposition behavior in Lepidoptera. Annu Rev Entomol. 1994; 39: 377-400
51. Shi J, Ma X, Zhang J, Zhou Y, Liu M, Huang L, et al. Chromosome conformation capture resolved near complete genome assembly of broomcorn millet. Nat Commun. 2019; 10: 464
52. Servant N, Varoquaux N, Lajoie BR, Viara E, Chen CJ, Vert JP, et al. HiC-Pro: an optimized and flexible pipeline for Hi-C data processing. Genome Biol. 2015; 16: 1-11
53. Burton JN, Adey A, Patwardhan RP, Qiu R, Kitzman JO, Shendure J. Chromosome-scale scaffolding of de novo genome assemblies based on chromatin interactions. Nat Biotech. 2013; 31: 1119-1125

54. Simão FA, Waterhouse RM, Ioannidis P, Kriventseva EV, Zdobnov EM. BUSCO: assessing genome assembly and annotation completeness with single-copy orthologs. Bioinformatics. 2015; 31: 3210-3212
55. Luo R, Liu B, Xie Y, Li Z, Huang W, Yuan J, et al. SOAPdenovo2: an empirically improved memory-efficient short-read de novo assembler. Gigascience. 2012; 1, 1: 2047-217X
56. Boetzer M, Henkel CV, Jansen HJ, Butler D, Pirovano W. Scaffolding pre-assembled contigs using SSPACE. Bioinformatics. 2011; 27: 578-579
57. Rabbit genome assembler. https://github.com/gigascience/rabbit-genome-assembler/. Accessed 20 Jun 2024.
58. Haas BJ, Papanicolaou A, Yassour M, Grabherr M, Blood PD, Bowden J, et al. *De novo* transcript sequence reconstruction from RNA-seq using the Trinity platform for reference generation and analysis. Nat Protoc. 2013; 8: 1494-1512
59. Kent WJ. BLAT - the BLAST-like alignment tool. Genome Res. 2002; 12: 656-664
60. Emms DM, Kelly S. OrthoFinder: phylogenetic orthology inference for comparative genomics. Genome Biol. 2019; 20: 1-14
61. Katoh K, Standley DM. MAFFT multiple sequence alignment software version 7: improvements in performance and usability. Mol Biol Evol. 2013; 30: 772-780
62. Capella-Gutiérrez S, Silla-Martínez JM, Gabaldón T. trimAl: a tool for automated alignment trimming in large-scale phylogenetic analyses. Bioinformatics. 2009; 25: 1972-1973
63. Stamatakis A. RAxML version 8: a tool for phylogenetic analysis and post-analysis of large phylogenies. Bioinformatics. 2014; 30: 1312-1313
64. Darriba D, Taboada GL, Doallo R, Posada D. ProtTest 3: fast selection of best-fit models of protein evolution. Bioinformatics. 2011; 27: 1164-1165
65. Sanderson MJ. r8s: inferring absolute rates of molecular evolution and divergence times in the absence of a molecular clock. Bioinformatics. 2003; 19: 301-302
66. Eddy SR. Accelerated profile HMM searches. PLoS Comput Biol. 2011; 7: e1002195

67. Li B, Dewey CN. RSEM: accurate transcript quantification from RNA-Seq data with or without a reference genome. BMC Bioinformaticsl. 2011; 12: 1-16

68. Nguyen LT, Schmidt HA, von Haeseler A, Minh BQ. IQ-TREE: a fast and effective stochastic algorithm for estimating maximum-likelihood phylogenies. Mol Biol Evol. 2015; 32: 268-274

69. Minh BQ, Nguyen MA, von Haeseler A. Ultrafast approximation for phylogenetic bootstrap. Mol Biol Evol. 2013; 30: 1188-1195

70. Guindon S, Dufayard JF, Lefort V, Anisimova M, Hordijk W, Gascuel O. New algorithms and methods to estimate maximum-likelihood phylogenies: assessing the performance of PhyML 3.0. Syst Biol. 2010; 59: 307-321

71. Kumar S, Stecher G, Li M, Knyaz C, Tamura K. MEGA X: molecular evolutionary genetics analysis across computing platforms. Mol Biol Evol. 2018; 35: 1547

72. Ronquist F, Teslenko M, Van Der Mark P, Ayres DL, Darling A, Höhna S, et al. MrBayes 3.2: efficient Bayesian phylogenetic inference and model choice across a large model space. Syst Biol. 2012; 61: 539-542

73. Chen C, Chen H, Zhang Y, Thomas HR, Frank MH, He Y, et al. TBtools: an integrative toolkit developed for interactive analyses of big biological data. Mol Plant. 2020; 13: 1194-1202

74. Bailey TL, Boden M, Buske FA, Frith M, Grant CE, Clementi L, et al. MEME SUITE: tools for motif discovery and searching. Nucleic Acids Res. 2009; 37: W202-W208

75. Hubley R, Finn RD, Clements J, Eddy SR, Jones TA, Bao W, et al. The Dfam database of repetitive DNA families. Nucleic Acids Res. 2016; 44: D81-D89

76. Shao C, Sun S, Liu K, Wang J, Li S, Liu Q, et al. The enormous repetitive *Antarctic krill* genome reveals environmental adaptations and population insights. Cell. 2023; 186: 1279-1294

77. Jiang NJ, Tang R, Wu H, Xu M, Ning C, Huang LQ, et al. Dissecting sex pheromone communication of *Mythimna separata* (Walker) in North China from receptor molecules and antennal lobes to behavior. Insect Biochem Mol Biol. 2019; 111: 103176

78. Liu J, Zhang R, Tang R, Zhang Y, Guo R, Xu G, et al. The role of honey bee derived aliphatic esters in the host-finding behavior of *Varroa destructor*. Insects. 2022; 14: 24
79. Jumper J, Evans R, Pritzel A, Green T, Figurnov M, Ronneberger O, et al. Highly accurate protein structure prediction with AlphaFold. Nature. 2021; 596: 583-589
80. Kim S, Chen J, Cheng T, Gindulyte A, He J, He S, et al. PubChem 2019 update: improved access to chemical data. Nucleic Acids Res. 2019; 47: D1102-D1109
81. Pérez-Escudero A, Vicente-Page J, Hinz RC, Arganda S, De Polavieja GG. idTracker: tracking individuals in a group by automatic identification of unmarked animals. Nat Methods. 2014; 11: 743-748
82. Pang Z, Chong J, Zhou G, de Lima Morais DA, Chang L, Barrette M, et al. MetaboAnalyst 5.0: narrowing the gap between raw spectra and functional insights. Nucleic Acids Res. 2021; 49: W388-W396
83. Tang R, Huang C, Yang J, Rao Z, Cao L, Bai P, et al. Supporting data for "A ghost moth olfactory prototype of the lepidopteran sex communication". GigaScience Database. 2024; https://doi.org/10.5524/102540

Figure legends

Figure 1. Phylogenomics and olfactory morphology of the ghost moths comparing with other species in Lepidoptera. **(A)** Dated evolutionary tree of Lepidoptera relationships. Two of the non-lepidopteran species were placed on outgroup branches including *D. melanogaster* and *T. castaneum*. The tree was inferred through a maximum-likelihood analysis of 634,106 amino acid sites from 1,547 strict single-copy genes employing VT + F model and 1000 bootstrap replicates. Branch lengths were optimized and node ages estimated using the penalized likelihood (PL) methods with truncated Newton (TN) algorithm in r8s [56]. Scale bar is in millions of years. Data resources were listed in Table S1. **(B)** Adult head development of Hepialidae *T. xiaojinensis* comparing to moth *S. frugiperda* and butterfly *P. rapae*. Orange arrow indicates the labial palp. Blue arrow indicates the proboscis which lacks in the ghost moth. **(C)** Antennal sensilla morphology of selected Lepidoptera by scanning electron microscope. **(D)** Glomerular counts of tested Lepidoptera observed by confocal laser scanning microscopy system. Red coded bars indicate predicted male MGCs, and green bars indicate female LFGs. Numbers indicate standard errors of means. Lower case letters indicate significant differences of glomerular counts among species (GLM and Tukey HSD, male: $F_{15, 32} = 48.2$, $P < 0.0001$, female: $F_{15, 32} = 30.9$, $P < 0.0001$).

Figure 2. Evolution of sex pheromone-related odorant receptors among *T. xiaojinensis*, caddisfly, and other Lepidoptera. **(A)** Rooted Maximum Likelihood (ML) tree of 387 selected lepidopteran ORs which included reported ORs of moths, mapped ORs in the linearization analysis in (B) by TxiaOR19 tandem, and ORs obtained from pre-lepidopteran species by blasting with TxiaOR19 tandem against nr database (Data S4). The evolutionary distances were computed using

the 'Auto' option in IQ-TREE [68] with ultrafast [69] 1000 bootstraps and the Shimodaira-Hasegawa-like approximate likelihood-ratio test [70]. Tested species included *A. jianchuanensis* (Ajia), *T. armoricanus* (Tarm), *T. xiaojinensis* (Txia), *P. xylostella* (Pxyl), *C. pomonella* (Cpom), *B. mori* (Bmor), *S. exigua* (Sexi), *S. litura* (Slitu), *S. littoralis* (Slit), *Heliothis virescens* (Hvir), *H. armigera* (Harm), *H. assulta* (Hass), *Ectropis grisescens* (Egri), *Operophtera brumata* (Obru), *Agrotis segetum* (Aseg), *Eriocrania semipurpurella* (Esem), and *Lampronia capitella* (Lcap), *Limnephilus marmoratus* (Larm), *Micropterix aruncella* (Maru), *Incurvaria masculella* (Imas), *Nematopogon swammerdamellus* (Nswa), *Coleophora flavipennella* (Cfla), *Manduca sexta* (Msex), *A. ipsilon* (Aips), *Athalia rosae* (Aros), *Hhermetia illucens* (Hill), *Culex quinquefasciatus* (Cqui), *Aphis gossypii* (Agos), *Schistocerca americana* (Same), and *S. cancellata* (Scan). Tree topology was cross-checked with Neighbor-Joining and Bayes approaches (Figure S9). Red arrows indicate key bootstrap values related to type I PRs and TxiaOR19 tandem. Blue arrows indicate ancestral ORs from caddisflies related to type I PRs and TxiaOR19 tandem. **(B)** Linearization of TxiaOR18c-TxiaOR19 tandem proteins with chromosomes from selected species showed by circos plot. Reported chromosome assemblies (chr) from *L. marmoratus*, *M. aruncella*, *I. masculella*, *N. swammerdamellus*, *C. flavipennella*, *C. pomonella*, *M. sexta*, *B. mori*, and *A. ipsilon* were used (Table S1). ORs mapped by TxiaOR18c or TxiaOR19 tandem were colored in blue, and those PR mapped were colored in red. **(C)** Motif identification towards PR mapped ORs from caddisfly and moths. **(D)** Motif identification towards TxiaOR18c/19 tandem mapped ORs from caddisfly and moths. **(E)** Distribution of motifs identified in (C) and (D), showing overlaps from PR and TxiaOR18c/19 clades.

Figure 3. Genome and OR evolution reflected by landscapes of transposable elements (TEs). (A) Detailed TE landscapes of the ghost moths, caddisfly, and white-barred gold. Times of TE burst events were estimated according to CpG adjusted Kimura substitution levels and a reported arthropod substitution rate of 6.19 x 10^{-10} per site per generation [76]. Red arrowheads indicate TE burst events. **(B)** Overview of asymmetric divergence of duplicated pheromone-related ORs from caddisfly to higher Lepidoptera. The TxiaOR18c/19 duplications predominate in caddisfly and primitive moths, but they were replaced by functional PR duplications in higher moths during evolution.

Figure 4. Resulted dual attraction in sex communications of the ghost moth adults. **(A)** Schematic shows set-up of the courtship arena of *T. xiaojinensis* adults. **(B)** Comparison of calling rates which were reflected by fluttering behaviors in both sexes (Binary test against even distribution, $P = 0.33$). **(C)** Left shows representative behavioral traces of male and female adults tracked by idTracker [81]. Right shows comparison of distance per min between male and female *T. xiaojinensis* adults (Mann Whitney test, U = 137, $P = 0.8055$).

Figure1

Click here to access/download;Figure;Figure01.tif

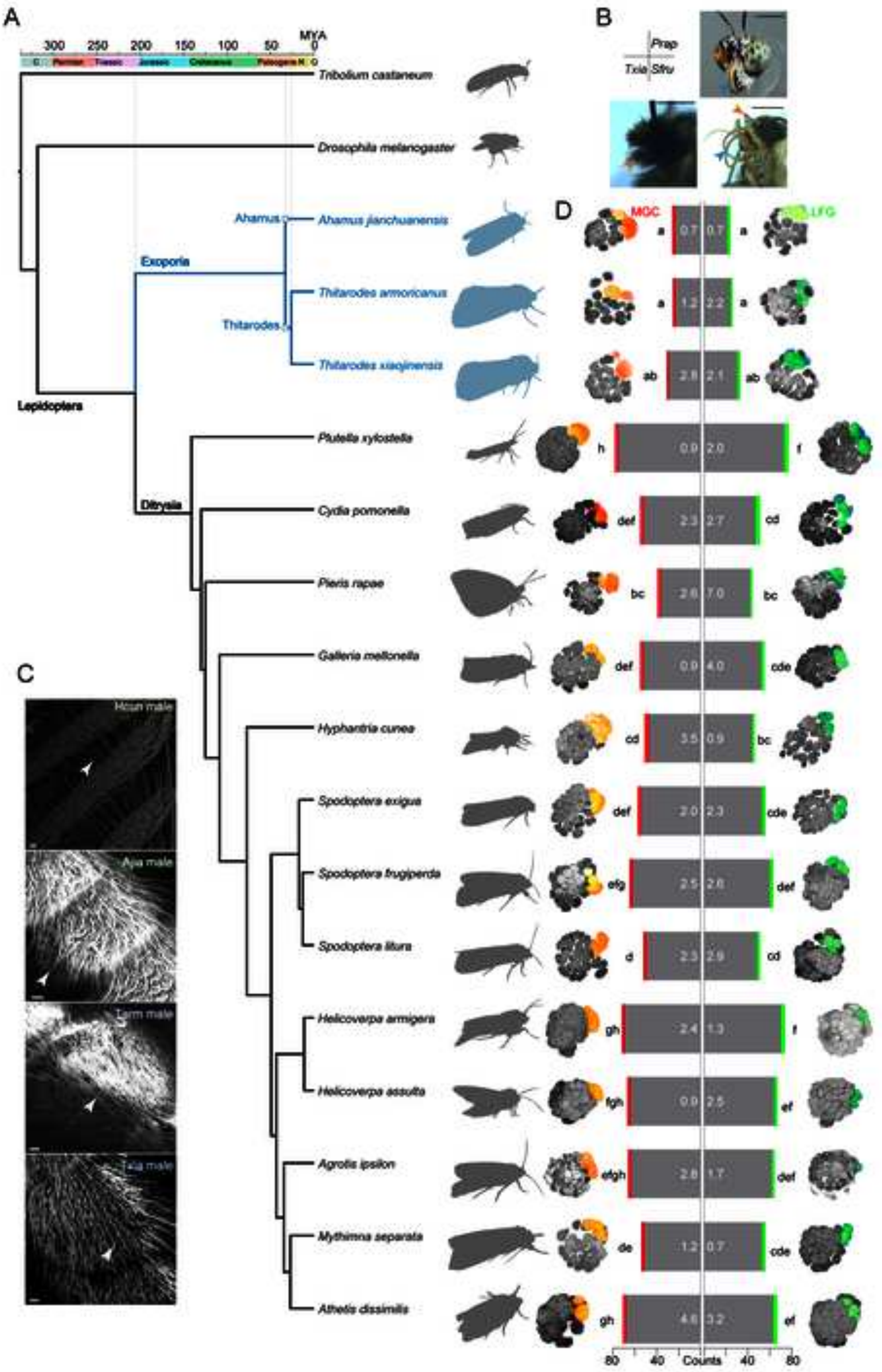

Figure2_revision2

Click here to access/download;Figure;Figure02.png

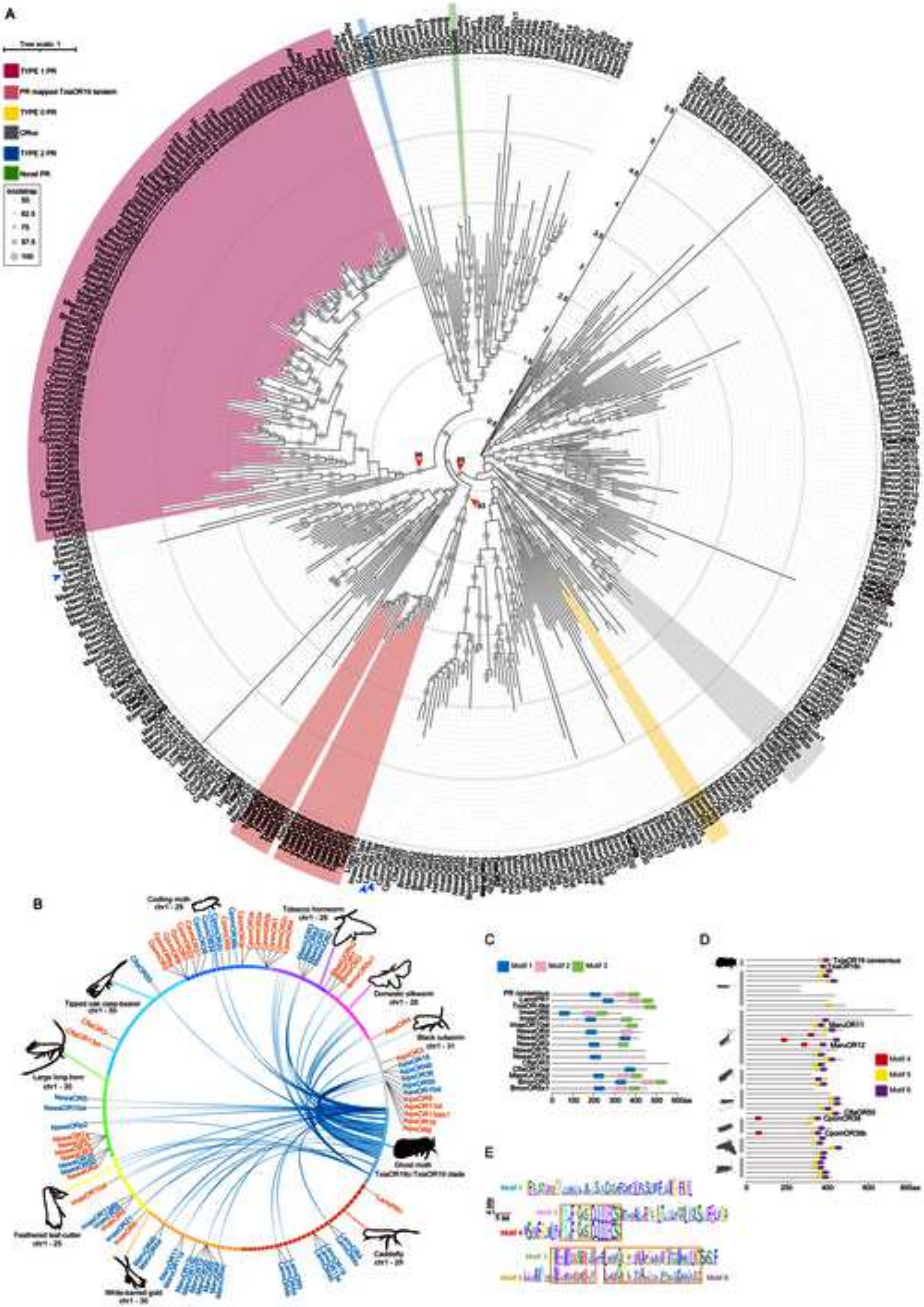

Click here to access/download;Figure;Figure03.tif

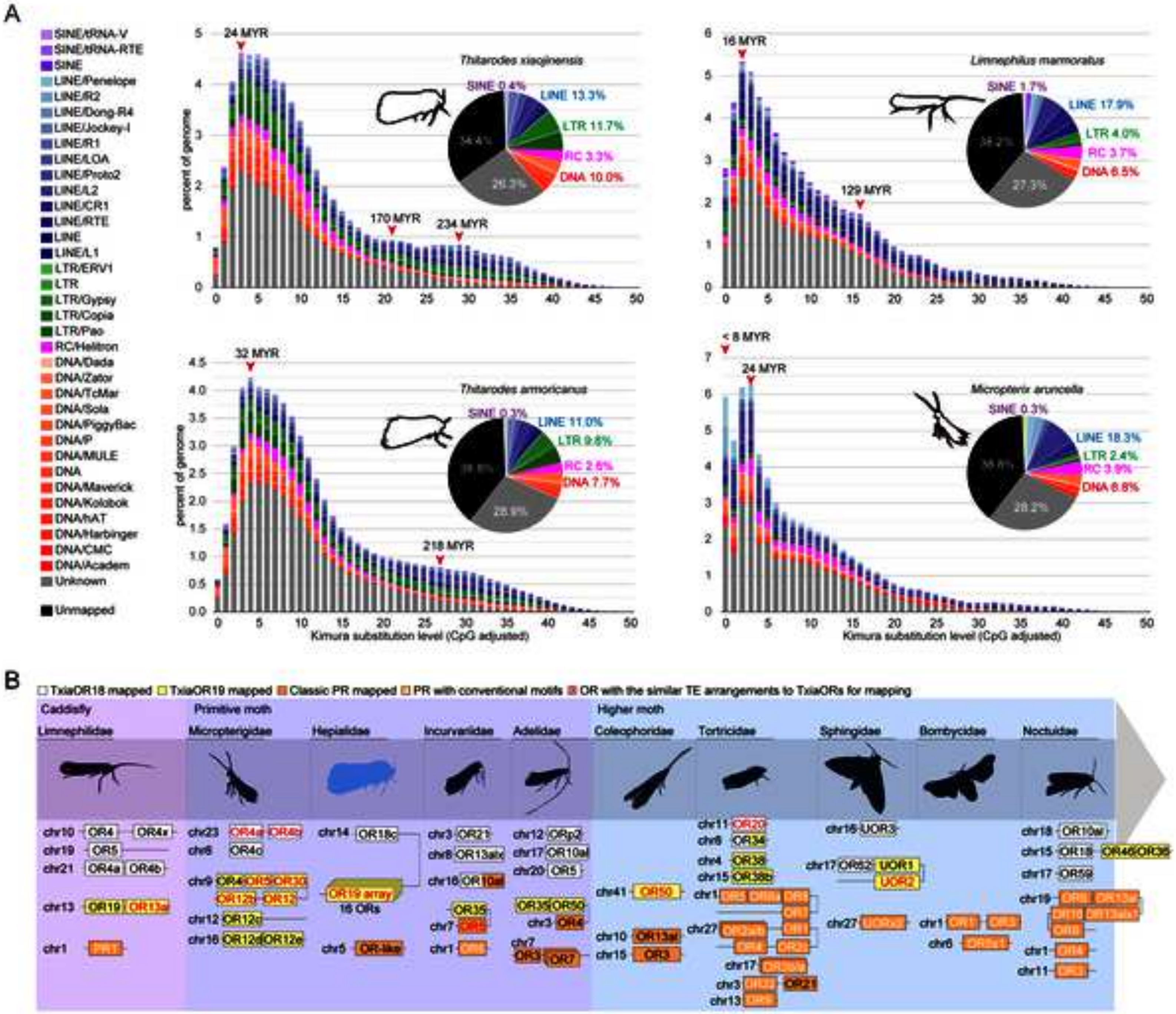

Figure4

Click here to access/download;Figure;Figure04.tif

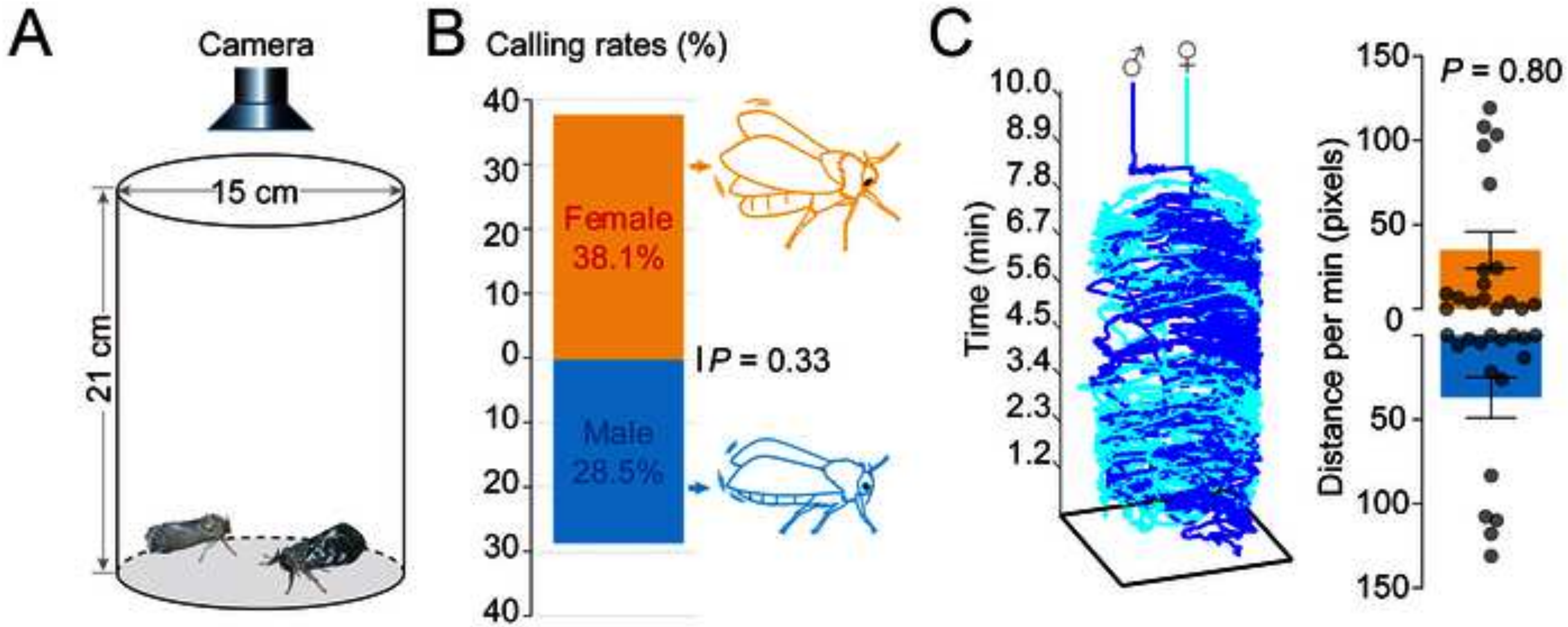

Click here to access/download
Supplementary Material
Supplementary materials_R2_submit.docx

Data S1

Click here to access/download
Supplementary Material
Data S1.xlsx

Data S2

Click here to access/download
Supplementary Material
Data S2.fasta

Data S3

Click here to access/download
Supplementary Material
Data S3.rar

Data S4

Click here to access/download
Supplementary Material
Data S4.fas

Click here to access/download;Personal Cover;Letter_of_response_R2_submit.docx

GIGA-D-23-00252R1
A ghost moth olfactory prototype of the lepidopteran sex communication
Rui Tang; Cong Huang; Jun Yang; Zhong-Chen Rao; Li Cao; Peng-Hua Bai; Xin-Cheng Zhao; Jun-Feng Dong; Xi-Zhong Yan; Fang-Hao Wan; Nan-Ji Jiang; Ri-Chou Han
GigaScience

Dear Prof Han,

Your manuscript "A ghost moth olfactory prototype of the lepidopteran sex communication" (GIGA-D-23-00252R1) has been assessed by our reviewers. Based on these reports, and my own assessment as Editor, I am pleased to inform you that it is potentially acceptable for publication in GigaScience, once you have carried out some essential revisions suggested by our reviewers.

Their reports, together with any other comments, are below. Please also take a moment to check our website at https://www.editorialmanager.com/giga/ for any additional comments that were saved as attachments.

Once you have made the necessary corrections, please submit a revised manuscript online at:

https://www.editorialmanager.com/giga/

If you have forgotten your username or password please use the "Send Login Details" link to get your login information. For security reasons, your password will be reset.

Please include a point-by-point within the 'Response to Reviewers' box in the submission system. Please ensure you describe additional experiments that were carried out and include a detailed rebuttal of any criticisms or requested revisions that you disagreed with. Please also ensure that your revised manuscript conforms to the journal style, which can be found in the Instructions for Authors on the journal homepage. On top of these, we suggest you find a copy editing company or friendly native English speaker to polish the grammar.

The due date for submitting the revised version of your article is 04 Aug 2024.

We look forward to receiving your revised manuscript soon.

Best wishes,

Hongfang Zhang
GigaScience
www.gigasciencejournal.com

Dear editor,

Thank you for considering publishing our manuscript in *Gigascience*. We have carefully revised our manuscript based on all suggestions from the reviewer and have answered the reviewer's questions point-by-point in the following letter. Furthermore, we have edited the language and fixed some grammatical errors, and we believe the current version is more readable. All changes have been marked by highlighting them (in green) in the text in the R2 submission files. We truly hope that the R2 revised manuscript is in good format and scientific quality to meet the journal's publishing standards. For more details, please see the manuscript or the response letter.

Best regards,

Ri-Chou Han

Reviewer reports:

Reviewer #1: The manuscript is substantially improved after the previous round of revisions and most of the reviewer comments have been addressed. However, reading the updated manuscript in this new light has brought to the forefront several other minor concerns that must be addressed before the manuscript should be published. Furthermore, while the manuscript is generally written very well, numerous small language errors are noticeable throughout the text. It is recommended to ensure the manuscript is revised for language after all scientific edits are made.

R: We appreciate your detailed assessments and suggestions. We followed each of the suggestions and revised our manuscript carefully. Accordingly, we have modified related parts in the manuscript (please see the green highlights in the text or in the response letter below). After addressing these concerns and fixing grammatical errors, we feel that your suggestions have indeed helped us improve the quality of this manuscript and make it more readable.

Minor revisions are suggested as follows:

Introduction

Line 94-96. "Moreover, ghost moths have undergone asymmetrical divergence of duplicated genes to deliver functional alterations in subsequent species, proving insights into the evolutionary process of Lepidoptera."

Which genes? Is this a widespread phenomena across all different kinds of genes, or is this referring to specific genes or gene families? Please clarify.

R: The duplication genes mentioned in here are *zen* gene. And the asymmetrical divergence is a widespread phenomenon in all genes during evolution. In the manuscript, we clarified as "Moreover, ghost moths have undergone asymmetrical divergence of duplicated gene, e.g. *zen* gene, to deliver functional alterations in subsequent species, providing insights into the evolutionary process of Lepidoptera".

Results

Line 211-212. "In all, the genomic backgrounds and receptor repertoires are specific in T. xiaojinensis, which may confer to the structural specificities described above."
It is not clear what this statement means. How are these features specific in T. xia, and what structural specificities are being referred to.

R: We deleted this statement, as it is confusing in the current version of manuscript since we removed some functional observations from the text.

Line 234-235. "possible functional drift of TxiaOR19."
Please describe what is meant here more clearly with this term. Functional drift in what manner, or relative to what?

R: We delete the term "functional drift" and change the sentence as "The replacement of the TxiaOR19 duplications with canonical PRs suggests possible different tuning characteristics of TxiaOR19 from a PR that narrowly tunes to sex pheromone components"

Line 241-244. "The results showed that TxiaOR19 had less binding affinity towards the panel of the ghost moth emissions, and its responding spectrum were relatively broad, indicating that ghost moth T. xiaojinensis may show unconventional courtship behaviors comparing to higher moths."
Might this result also suggest that other ORs, especially within the OR19 tandem array may be instead tuned to ghost moth emissions? Why aren't docking profiles examined for other OR19 sub-family receptors? In any case, this kind of statement seems more like a discussion statement than a results statement. I understand it is used to transition to the next paragraph, however, the logic for looking at behavior in males and females (as it turns out, non-dimorphic behavior), could just as well follow from the lack of sexual dimorphism in the antennal lobe, perhaps much better, as compared to the hypothetical ligand docking studies, which are far from complete.

R: We redocked all TxiaOR19 array ORs to ligands, and the results showed that all tested TxiaORs are not specifically tuned to the female-specific compound oleamide (see Figure S16), which supports our idea that the TxiaOR19 array has different tuning characteristics compared to the narrowly tuned PRs. In the manuscript, we have toned down our statements regarding OR functional docking, as this aspect is not our main focus in discussing the evolution of moth pheromone receptors. Please see the following statement: "The results showed that the TxiaOR19 array had less binding affinity towards the panel of ghost moth emissions, and the responding spectrum was relatively broad for all 16 tandem ORs."

Regarding the transition from molecular to behavioral aspects in the manuscript, we followed your suggestion by initially discussing the lack of sexual dimorphism in the antennal lobe before transitioning to the discussion of non-dimorphic behavior. See the "Considering ghost moths, including *T. xiaojinensis*, lack sexual dimorphism in their antennal lobe (Figure 1D), we hypothesized that *T. xiaojinensis* may also exhibit

unconventional calling and mating behaviors compared to higher moths."

Discussion.

Line 256. "Himalaya ghost moths offer a basal model for the study of olfaction evolution in insects"

I am not sure it is correct to say "insects" here. It may be more correct instead to say "Lepidoptera" or at the very most, the "Lepidoptera/Trichoptera" sister clades, since caddisflies are also included….but not more generally insects.

R: We adjusted "insect" to "Lepidoptera".

Line 278-279. "However, more experimental functional evidence needs to be provided for both TxiaOR19 and TxiaOR7…"

Again, here, shouldn't this refer to the TxiaOR19 sub-family/array ORs more generally, instead of only TxiaOR19?

R: We re-docked the TxiaOR array to ligands, please see the Figure S16. And we also adjust the "TxiaOR19" to "TxiaOR19 array" in the manuscript.

Line 282. "the TxiaOR19 array and LarmPR1 share motifs that reflect some exons on the loci"

It's not clear what this means. How do the motifs reflect exons? Which exons and which loci?

R: We removed "that reflect some exons on the loci" as there was no hard evidence to support this statement.

Line 282-283. "These motifs were separated before the evolution of Lepidoptera."

This is a vague statement. Do you mean before Lepidoptera and Trichoptera diverged from each other, or before the ghost moths split off from the more recently evolved moths? Please clarify.

R: We apologize for this vague statement. We clarified as "These motifs were likely to be already separated before Lepidoptera and Trichoptera diverged from each other".

Line 286-287. "On the other hand, the shared motif regions could be traced back to earlier dipteran species"

Which shared motif regions? Are you referring to the ones in the PRs as shown in Fig 2C/2D/2E? Or motifs present in other organisms, as mentioned in the previous sentence?

R: We hope to compare the motifs of PRs to the TxiaOR19. We revised this sentence as "On the other hand, PRs shared motif regions with TxiaOR19 and the later could be blasted to ORs in dipteran species where large OR duplications existed such as in *Bactrocera dorsalis*".

Line 297-298. "Enlarged MGCs in butterfly Piers rapae suggest that the sexual dimorphic sex pheromone recognition system is widely used by Lepidoptera"
Please check the species, as Piers rapae is not at all mentioned in the cited article, no. 49. I would also further caution against making this statement about the sexually dimorphic sex pheromone recognition system, as the authors of the cited article emphasize that their butterfly MGC is not implied to be homologous to the moth MGC.

R: We apologize for the inconsistent citation and unclearly statement. We revised this sentence as "The enlarged MGCs in the butterflies, such as *Pieris rapae* and *Godyris zavaleta* [49], suggest that the sexual dimorphic brain organizations are widely presented by Lepidoptera, however, the link between morphology and pheromone recognition in butterflies requires further examination".

Materials and Methods.
Section on Scanning electron Microscopy.
For the washes, dehydration and drying steps, please specify the length of times for each of these steps.
R: We revised the paragraph by adding these essential information as "The antennae of 1-3 d adults were cut from base and fixed in 0.25% glutaraldehyde at 4 °C overnight. After three washes at room temperature with 0.1 M phosphate-buffered saline (PBS, pH 7.4) for 15 min each, antennae were dehydrated through a laddered ethanol series (30, 50, 70, 80, 90, and 100%) for 15 min each and dried for 15 min in a critical point drier (Bal-Tel CPD 030) before mounted on aluminum stubs. The mounted antennae were coated with gold spray (Bal-Tel SCD 005) and observed with SEM instrument (FEI Quanta 200,)."

Line 376. Change pollutions to contamination.
R: Revised.

Figures and Tables
Figure 2, Panels C-E. It is unclear why Motifs 1-3 are shown in 2C, while Motifs 4-6 are shown in 2D. And in 2E, motif 2 appears very similar to Motif 4, while Motif 3 appears very similar to Motifs 5 and 6. Based on this last point, the text from lines 204-211 is confusing, especially considering that in 2C and 2D, for the PR consensus representations, either Motifs 1-3 are shown in 2C or Motifs 4-6 are shown in 2D. If this is the PR consensus, and it is the same representation for 2C and 2D, shouldn't Motifs 1-6 be shown for both? Please try and re-write the relevant text in the main manuscript and figure legend so that this concept is clearer.

R: The Motifs 1-3 are highly conserved motifs of PRs, reviewed by Zhang and Löfstedt in 2015 (doi.org/10.3389/fevo.2015.00105). We firstly used the MEME suite to check the signature Motifs of the PR clade, and confirmed them according to published works. The same approach was used then to test TxiaOR19 mapped ORs, and we thus named the later motifs as Motif 4-6. As the two clades were separately investigated, they had some

overlaps but should not be placed together. We are sorry for this mistake and removed PR consensus from Figure 2D in order not to draw any confusions. The descriptions in the manuscript as "Using MEME suite, we found that LarmPR1 exhibited all three signature motifs of PR consensus regions as reported before" and also as "On the other hand, the majority of TxiaOR19-mapped ORs had two motifs, with some having three motifs (named as motif 4-6) upon checking with the same approach".

Figure 3, Panel A. It is not clear where the burst events are. Are they marked by the red arrowheads? This should be written out clearly in the figure legend.
R: The red arrows indicate TE burst. We have added this information in the figure legend of Figure 3. See "Red arrowheads indicate TE burst events"

Figure S2. What do the black squares and grey bars represent? Please indicate their meaning in the figure legend.
R: We added explanations in the figure legend. Please see "Where square dots indicate each measurement of individual sample, grey bars indicate the ranges of O.I. from the species"

Figure S3. It appears that different sensilla types are present in the various different panels. Please indicate, with arrows and notations, in each panel, which sensilla type are the sensilla trichodea, as it is not obvious to an unfamiliar reader.
R: We added arrowheads to indicate sensilla trichodae in each panel, with figure legend revised "Arrowheads indicate representative sensilla trichodae morphology in each panel".

Figure S8. It appears that only 18 genes are shown here. It should be made clear in the figure legend that these are the intact homologues and the pseudogenes are excluded, if that is indeed the case.
R: Yes, it is the case. We added relevant clarification to the figure legend as "Tandem *TxiaOr19* intact *Or* duplications exclude pseudogenes".

Table S2. The title of this table has been updated to say "comparison of chemosensory genes in head among the tested Lepidoptera. Is this referring to head only? Or is it an indication of head/antennal expression? Furthermore, in the previous round of revision, it was mentioned that the numbers for C. pomonella were inaccurate and they have been updated to reflect all ORs/GRs/IRs identified in the genome, as reported by Wan et al., 2019. However, now that the title has changed to reflect chemosensory genes in head, the numbers for C. pomonella are again inaccurate, as they reflect presence of these genes in the genome, not in the head.
R: We are sorry for this. The correct title for this table is revised to "Table S2. Comparison of chemosensory gene annotations among the tested or reported Lepidoptera."

Reviewer #2: My comments to the manuscript have been properly addressed. I have no further concerns.
R: Thank you again for your time.

Graphical abstract

Click here to access/download;Graphical Abstract;Graphical Abstract.tif

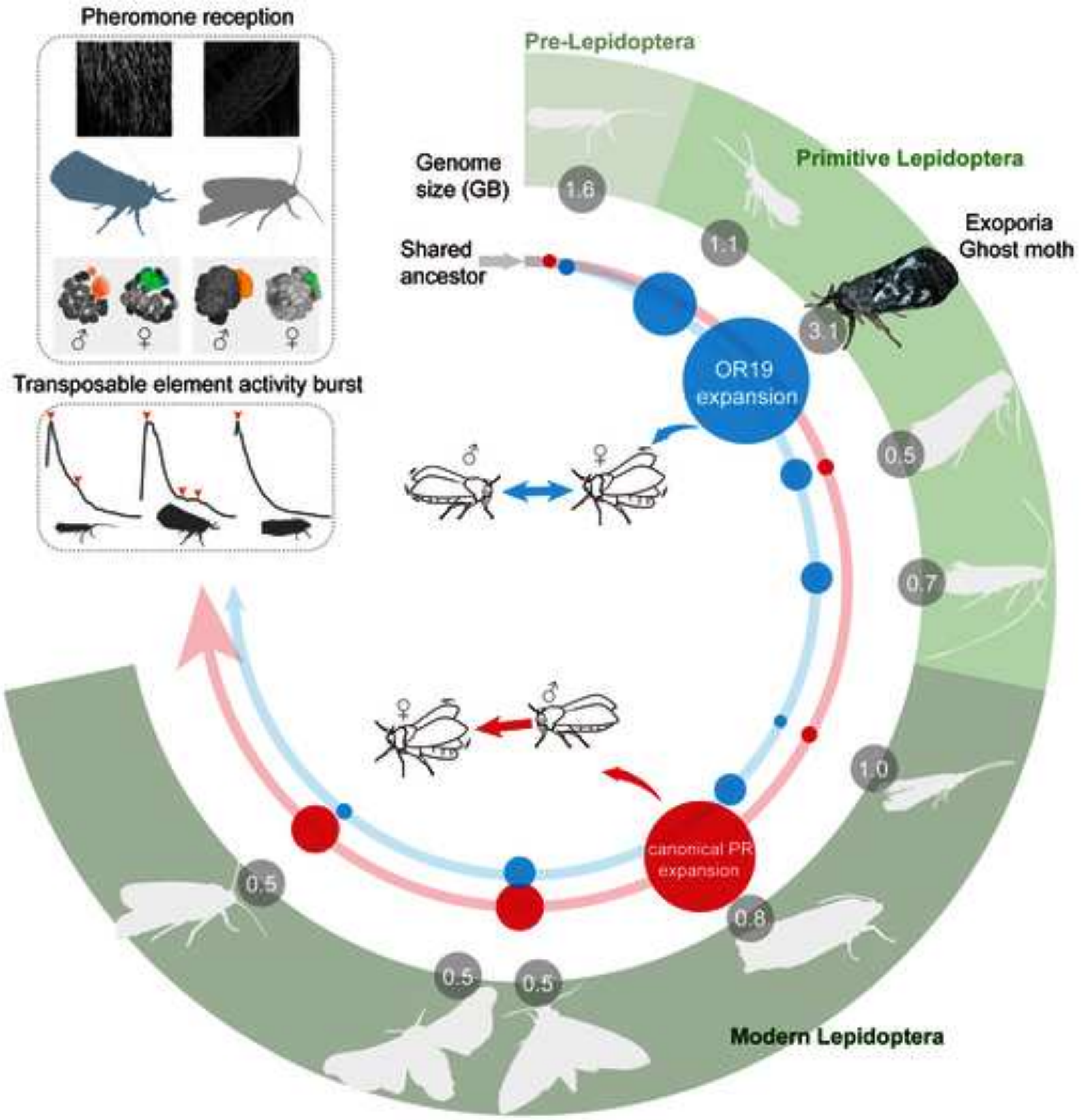

Supplement: giae044_GIGA-D-23-00252_Revision_2 [file giae044_giga-d-23-00252_revision_2.pdf]
