# Supplementary material for: A ghost moth olfactory prototype of the lepidopteran sex communication
Source: Gigascience. 2024 Jul 19;13:giae044. doi: 10.1093/gigascience/giae044 (PMC11258902; doi:10.1093/gigascience/giae044)
Supplement: giae044_Supplemental_Files [file giae044_supplemental_files.zip › Supplementary materials_R2_submit.docx]

**Supplementary materials for**

**A ghost moth olfactory prototype of the lepidopteran sex communication**

Rui Tang^1#a^, Cong Huang^23#^, Jun Yang^4^, Zhong-Chen Rao^1^, Li Cao^1^, Peng-Hua Bai^5^, Xin-Cheng Zhao^6^, Jun-Feng Dong^7^, Xi-Zhong Yan^4^, Fang-Hao Wan^23^, Nan-Ji Jiang^8^*, Ri-Chou Han^1^*

1 Guangdong Key Laboratory of Animal Conservation and Resource Utilization, Guangdong Public Laboratory of Wild Animal Conservation and Utilization, Institute of Zoology, Guangdong Academy of Sciences, Guangzhou, China 510260

2 State Key Laboratory for Biology of Plant Diseases and Insect Pests, Institute of Plant Protection, Chinese Academy of Agricultural Sciences, Beijing, China 100193

3 Shenzhen Branch, Guangdong Laboratory for Lingnan Modern Agriculture, Genome Analysis Laboratory of the Ministry of Agriculture, Agricultural Genomics Institute at Shenzhen, Chinese Academy of Agricultural Sciences, Shenzhen, China 518120

4 College of Plant Protection, Shanxi Agricultural University, Taigu, Shanxi, China 030801

5 Institute of Plant Protection, Tianjin Academy of Agricultural Sciences, Tianjin, China 300384

6 Henan International Laboratory for Green Pest Control, College of Plant Protection, Henan Agricultural University, Zhengzhou, China 450046

7 Forestry College, Henan University of Science and Technology, Luoyang, China 471000

8 Department of Evolutionary Neuroethology, Max Planck Institute for Chemical Ecology, Hans-Knöll-Straße 8, Jena, Germany D-07745

*Correspondence:

Nan-Ji Jiang, njiang@ice.mpg.de Hans-Knöll-Straße 8, Jena, Germany. Tel. +49 (0)3641 57-1456.

Ri-Chou Han, hanrc@giz.gd.cn, 105 Xingang West Road, Haizhu District, Guangzhou. Tel. +86 020-84191089.

# Equal contribution was claimed.

**This file includes:**

1. **Figure S1**. Strength of chromosomal interactions within intervals.

2. **Figure S2**. Antennal and forewing length comparison among Lepidoptera.

3. **Figure S3**. Representative SEM photos of selected moth species.

4. **Figure S4**. Evolution of ordinary glomerular morphology in Lepidoptera.

5. **Figure S5**. Comparison of the cumulus/LFG1 considering volume weights.

6. **Figure S6**. PCA plot of MGC measurements with Lepidoptera species.

7. **Figure S7**. PCR verifications for annotated *TxiaOrs*.

8. **Figure S8**. Localization and exon-intron arrangements of *TxiaOr19* duplications.

9. **Figure S9**. Verifications of ML tree structures with NJ and BY methods.

10. **Figure S10**. Expression profiles of *TxiaOrs*.

11. **Figure S11**. Distributions of selected TxiaOR homologs across species.

12. **Figure S12**. Clustering of blasted ORs by TxiaOR19 lineage.

13. **Figure S13**. Bayes tree showing evolutionary relationships of mapped and nr blasted ORs related to TxiaOR19 tandem.

14. **Figure S14**. Comparison of overall TE distributions among caddisfly and moths.

15. **Figure S15**. Aligned Correlation matrix showing arrangement patterns of TEs in TxiaOR18c/19 mapped OR loci.

16. **Figure S16**. Adult emissions and predicted non-PR functioning of TxiaOR19.

17. **Table S1**. Data resources for the phylogenomics and comparative genomic analysis.

18. **Table S2**. Comparison of OR repertoires among the tested Lepidoptera.

19. **Table S3**. List of PCR primers used in the study.

**Other supplementary datasets for this manuscript include the followings:**

1. **Data S1 (separate file)**. Relative volumes of all glomeruli within the 16 Lepidoptera.

2. **Data S2 (separate file)**. Annotated TxiaOrs for RT-PCR verifications.

3. **Data S3 (separate file)**. Annotated *TxiaOr19* duplications.

4. **Data S4 (separate file)**. Select 387 insect ORs for establishment of phylogeny.


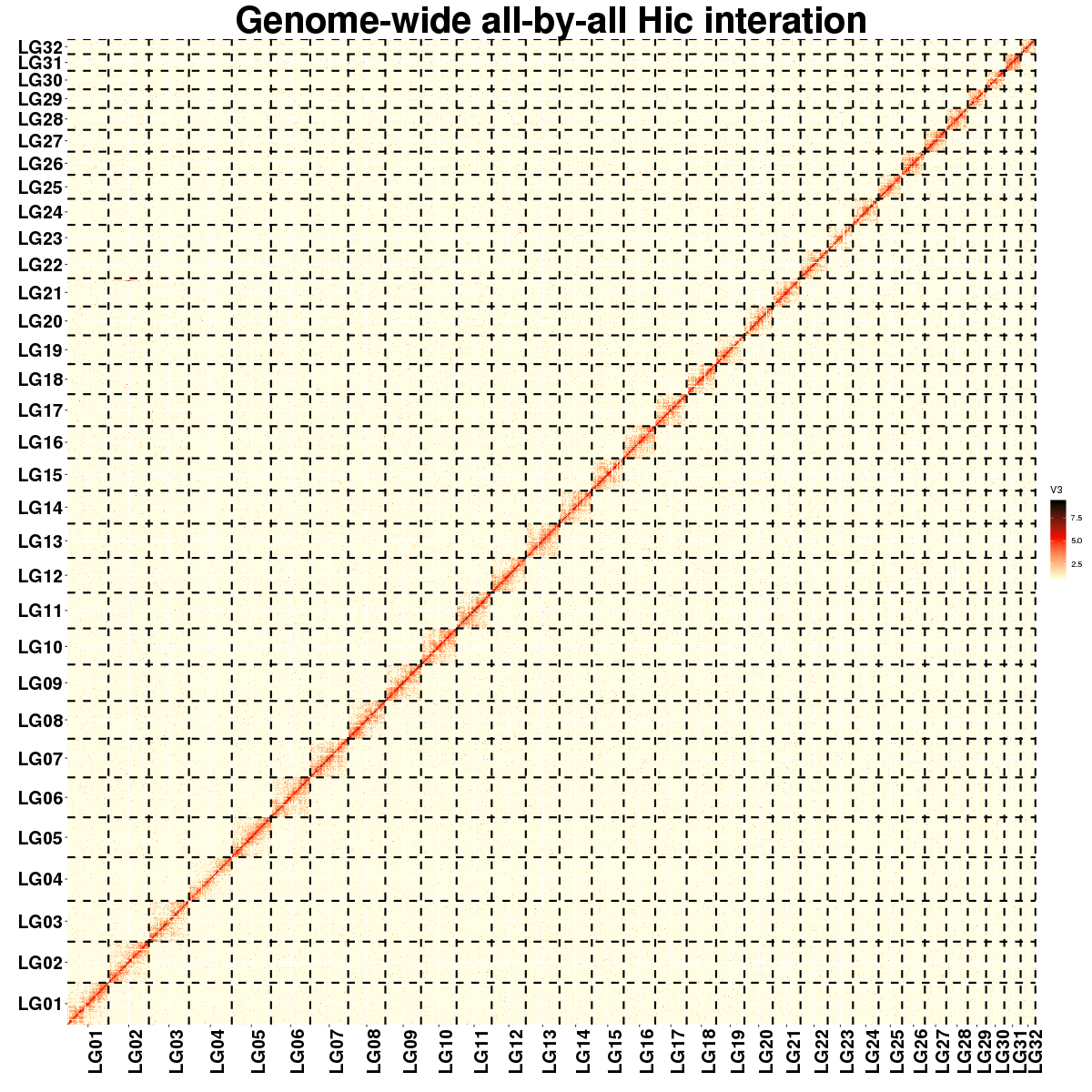


**Figure S1. Strength of chromosomal interactions within intervals**. Based on the strength signals of interactions between Contig sequences with their final determined order and orientation, we generated a Hi-C interaction heatmap for the chromosomes. The heatmap coordinates represent chromosomes, and the color of each point corresponds to the log value of the interaction strength of the respective genome bin pair, ranging from red to black indicating increasing interaction strength. Notably, the 32 chromosomal groups are clearly distinguishable. Within each group, interactions along the diagonal are stronger than those off the diagonal, indicating higher interaction strength between adjacent sequences (diagonal positions) in the Hi-C assembled chromosomes, consistent with the principles of Hi-C-assisted genome assembly. There is minimal noise (strong interaction strength) outside the diagonal, affirming the effectiveness of the genome assembly.

**
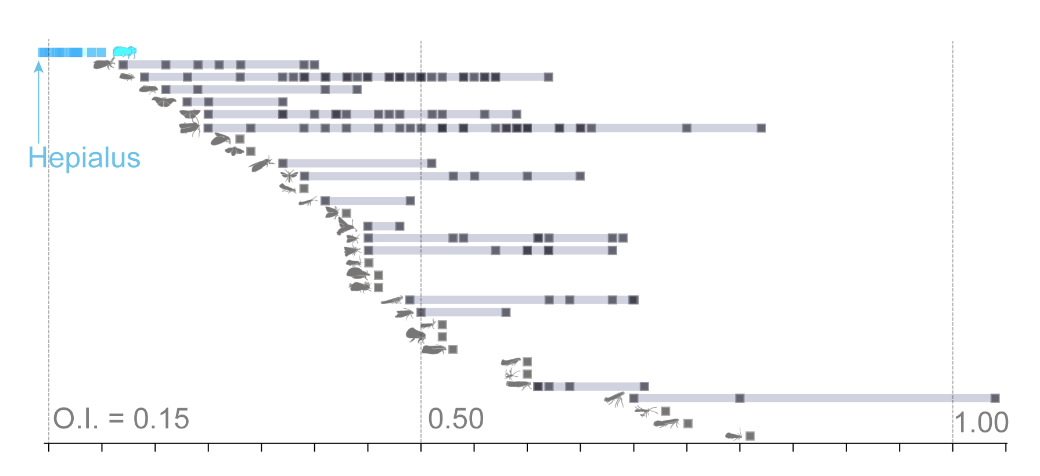
**

**Figure S2. Antennal and forewing length comparison among Lepidoptera**. Where square dots indicate each measurement of individual sample, grey bars indicate the ranges of O.I. from the species. O.I. indicates olfactory index which equals antenna/forewing. Data of hepialids were collected in the current work (blue), and other families were referred to published works [26].


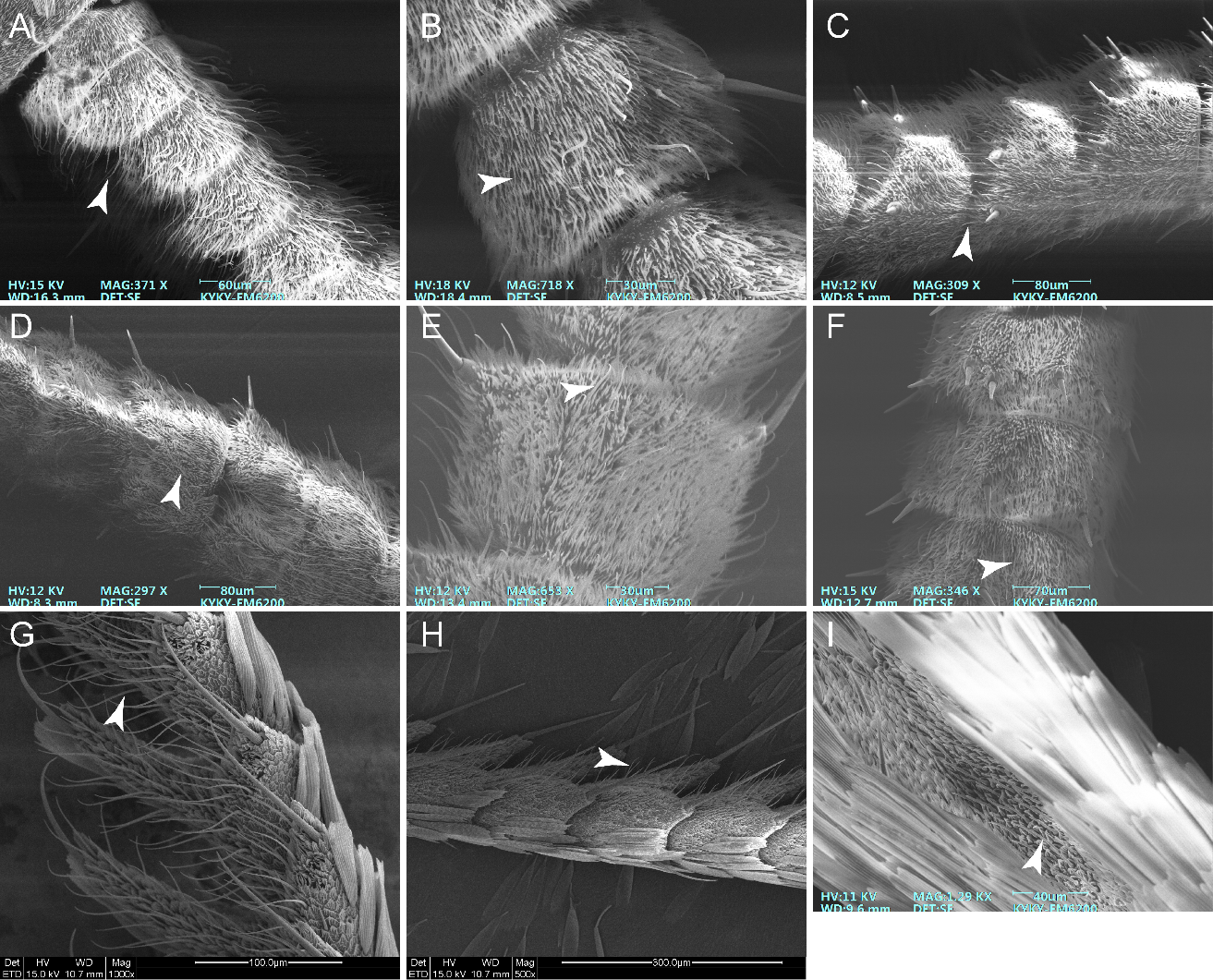


**Figure S3. Representative SEM photos of selected moth species**. Arrowheads indicate representative sensilla trichodae morphology in each panel. **(A)** *A. jianchuanensis* male. **(B)** *A. jianchuanensis* female. **(C)** *T. armoricanus* male. **(D)** *T. armoricanus* female. **(E)** *T. xiaojinensis* male. **(F)** *T. xiaojinensis* female. **(G)** *H. cunea* male. **(H)** *H. cunea* female. **(I)** *P. rapae* male showing only distributions of sensilla trichoidae in the “sacculus”.


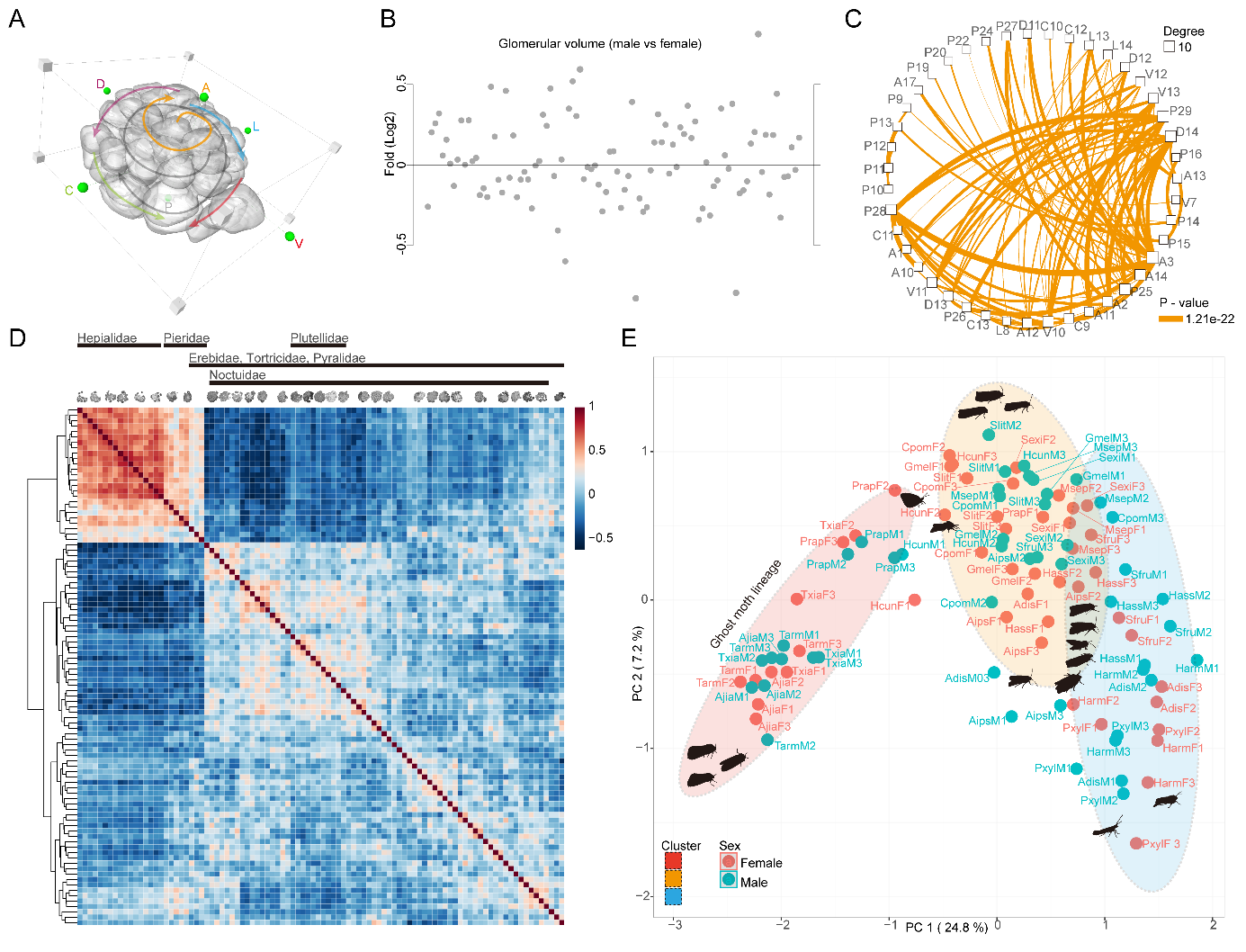


**Figure S4. Evolution of ordinary glomerular morphology in Lepidoptera.** **(A)** Schematic shows registration of glomerular organizations among species. Ordinary glomeruli were assigned according to their spatial locations as A: anterior, D: dorsal, V: ventral, C: central, L: lateral, and P: posterior. Arrows indicate numerical order of each spatial cluster. **(B)** Comparison of annotated glomerular volumes between male and female Lepidoptera. The analysis includes 101 assigned glomeruli. Fold change threshold was 2.0. **(C)** Debiased Sparse Partial Correlation (DSPC) network concerning volume relationships of selected 43 glomeruli across species. Glomerular volume was assigned as 0 when they were not observed in a species. Square size indicates correlation degree of each glomerulus. Line weight indicates the *P* value between each pair. **(D)** Correlation map of all glomeruli across tested 18 species. Representative MGC/LFG-free ALs of the species were shown on top, together with family ranges indicated. **(E)** Self Organizing Map (SOM) cluster analysis of tested species according to ordinary glomeruli.


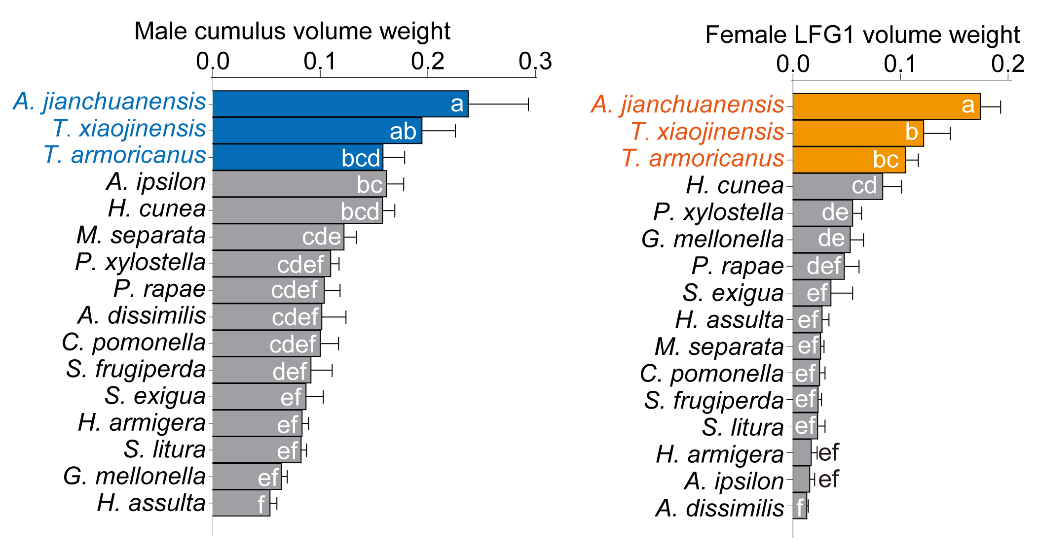


**Figure S5. Comparison of the cumulus considering volume weights in males (left) and LFG1 in females (right)**. Lower case letters indicate significant differences among species (GLM and Duncan multiple comparison, cumulus: *F*_15, 32_ = 6.05, *P* < 0.0001; LFG1: *F*_15, 32_ = 14.91, *P* < 0.0001).

**
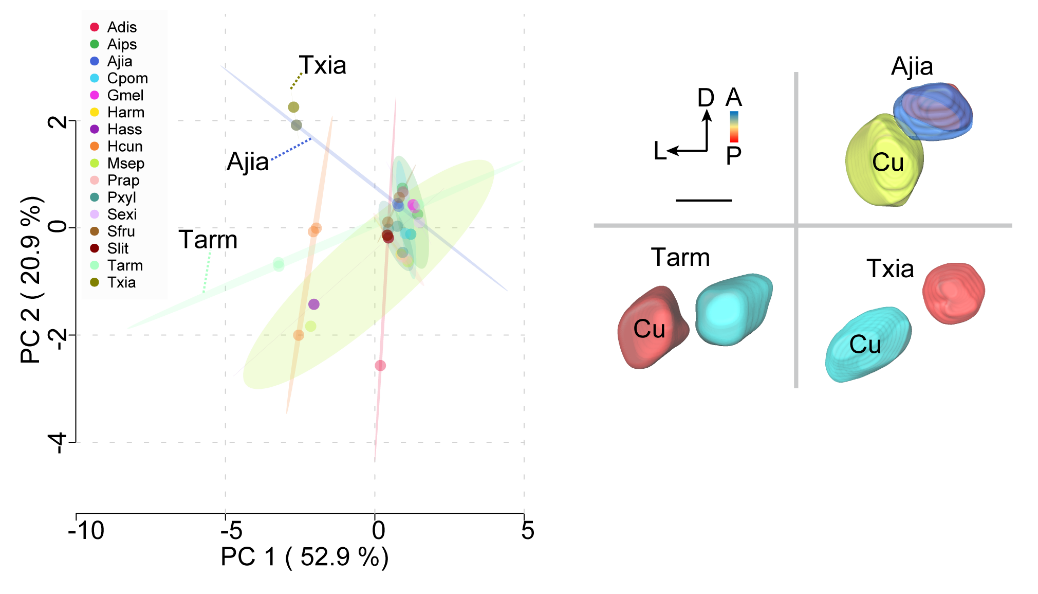
**

**Figure S6.** **PCA plot of MGC measurements with 16 tested Lepidoptera species at 73.8% fraction of explained variance**. Representative MGC glomeruli from *A. jianchuanensis*, *T. armoricanus*, and *T. xiaojinensis* were shown. Cu indicates the cumulus.


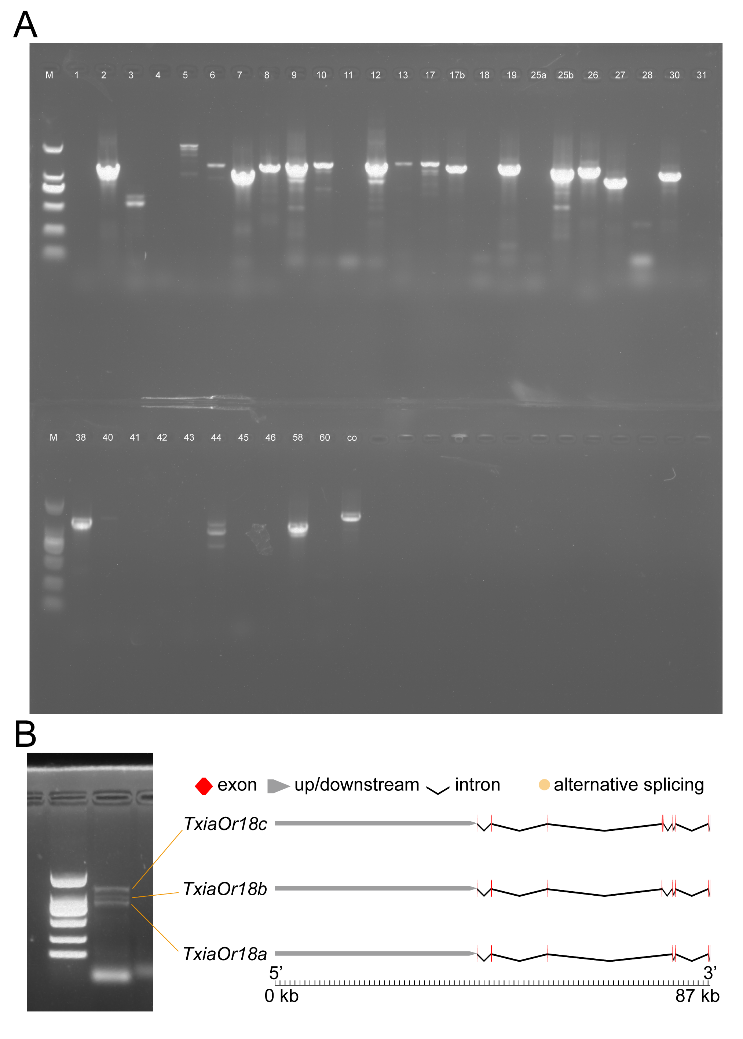


**Figure S7. PCR verifications for annotated *TxiaOrs***. **(A)** Cloned PCR products of respective *TxiaOrs* from antennal cDNA. M: DNA 2000D ladder. Captions indicate corresponding *TxiaOrs* according to the primers in Table S2. *TxiaOr18* was not cloned in this test, but we harvested its clones in another run using optimized primers [*TxiaOr18new*]. **(B)** PCR verifications and alternative splicing of *TxiaOr18*. The longest correspondence TxiaOR18c was used in all successive analysis.


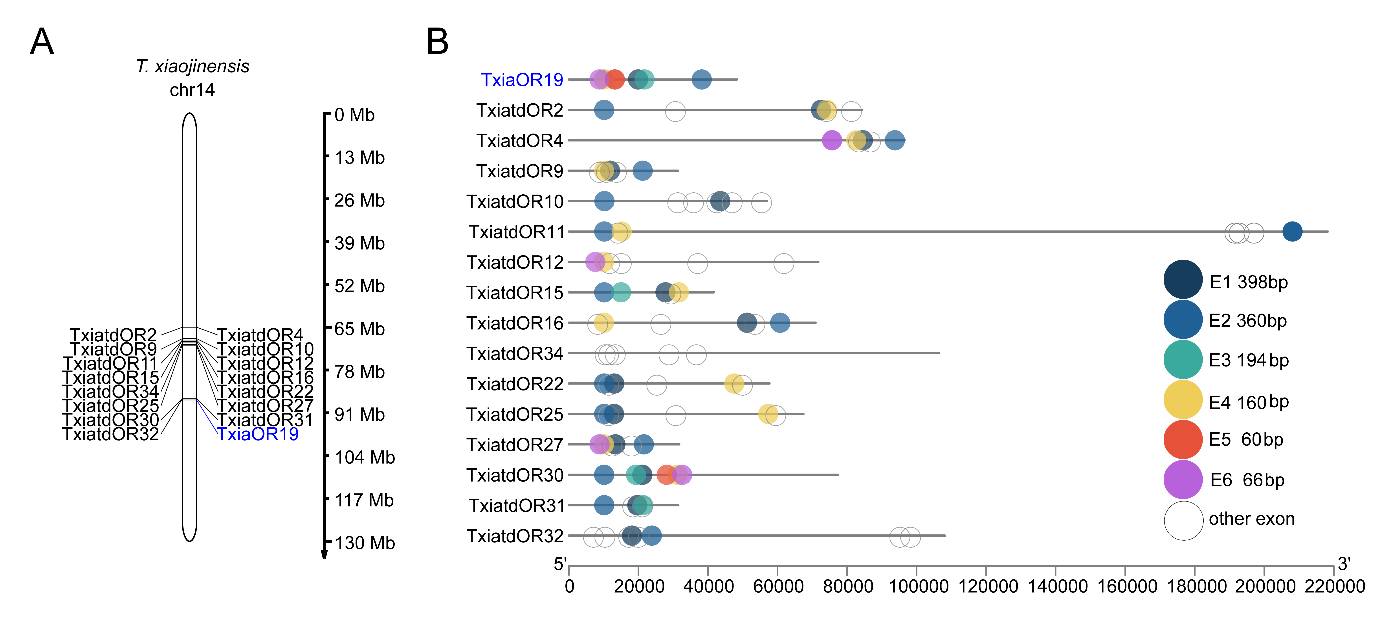


**Figure S8. Tandem *TxiaOr19* intact *Or* duplications exclude pseudogenes**. (**A**) Gene locations of the *TxiaOr19* tandem on chr14 of *T. xiaojinensis*. (**B**) Exon-intron arrangements of the *TxiaOr19* tandem.


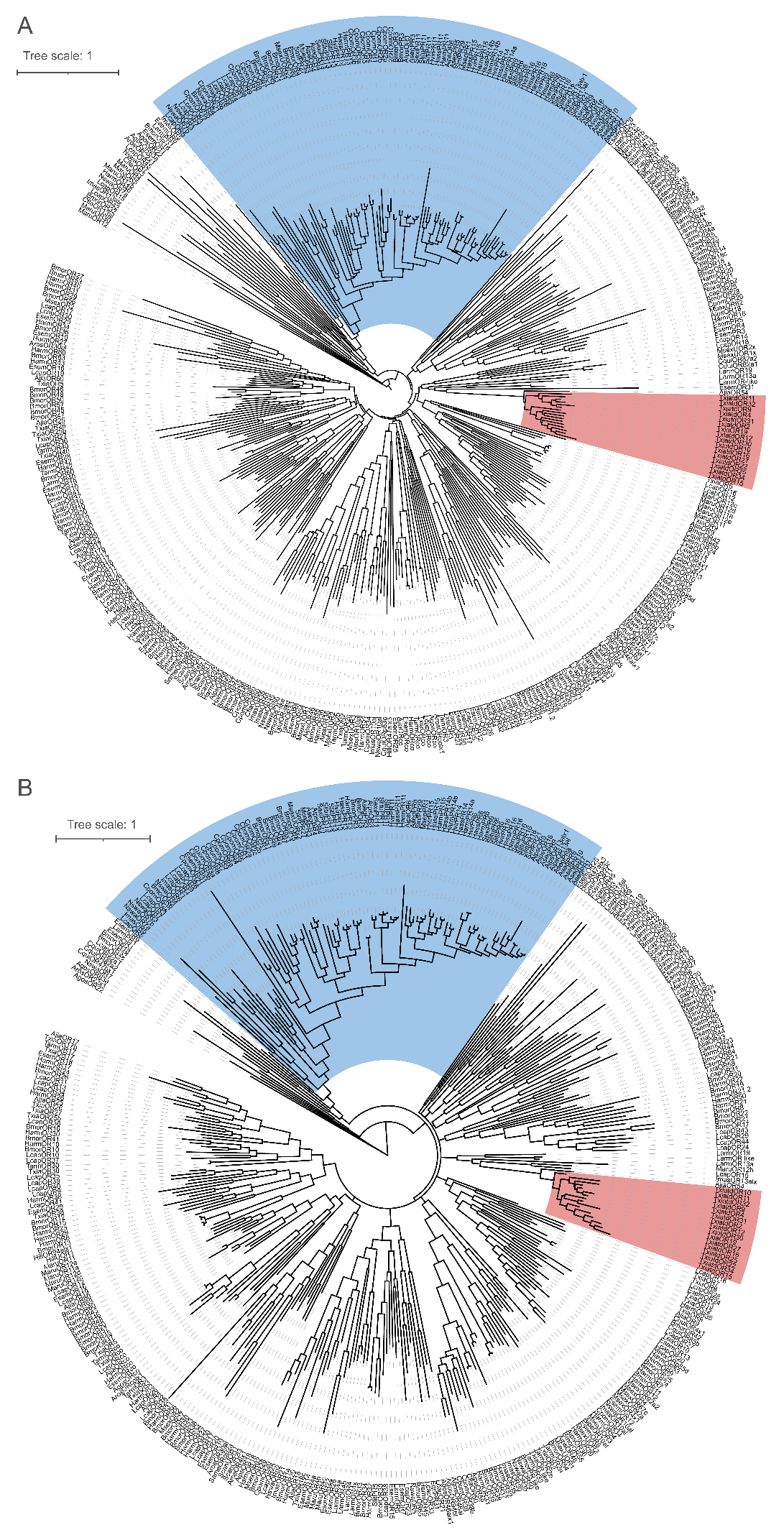


**Figure S9.** **Verifications of ML tree structures with NJ and BY methods**. The same batch of 387 ORs were used to establish the phylogeny as used in Figure 2A. Where red highlighted TxiaOR19 array ORs, and blue indicates canonical PRs. **(A)** NJ tree was established using MEGA X with the Dayhoff matrix based method. **(B)** Bayes tree was established by MrBayes using Blosum62 model. A total 200,000 generations were run and a relative burnin of 25% were used for diagnostics. The final average standard deviation of split frequencies was 0.12.


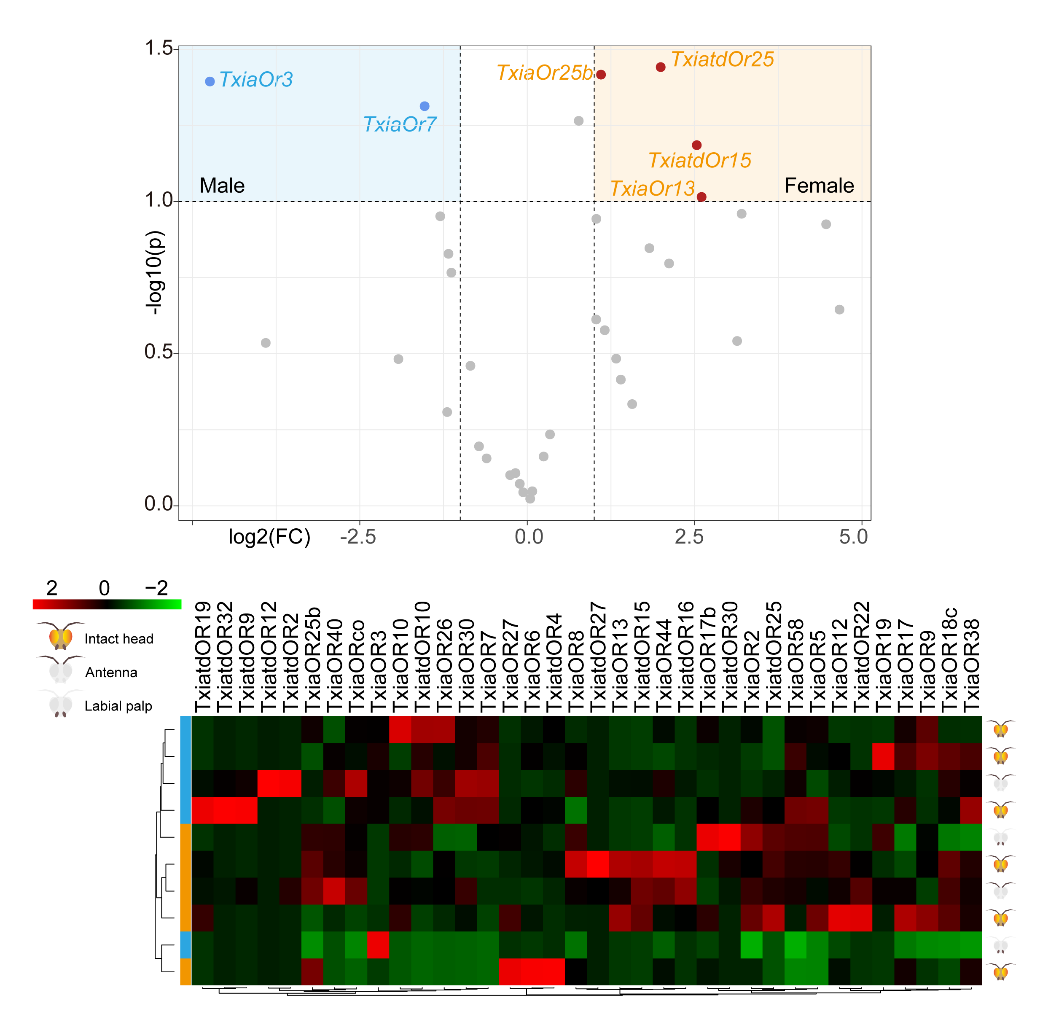


**Figure S10. Expression profiles of *TxiaOrs*.** Volcano plot (up) shows significantly different expressed *Ors* in *T. xiaojinensis* between male and female adults. Color-coded map (down) showing expression levels of identified *Ors* in *T. xiaojinensis* adult heads, antennae, or labial palps.


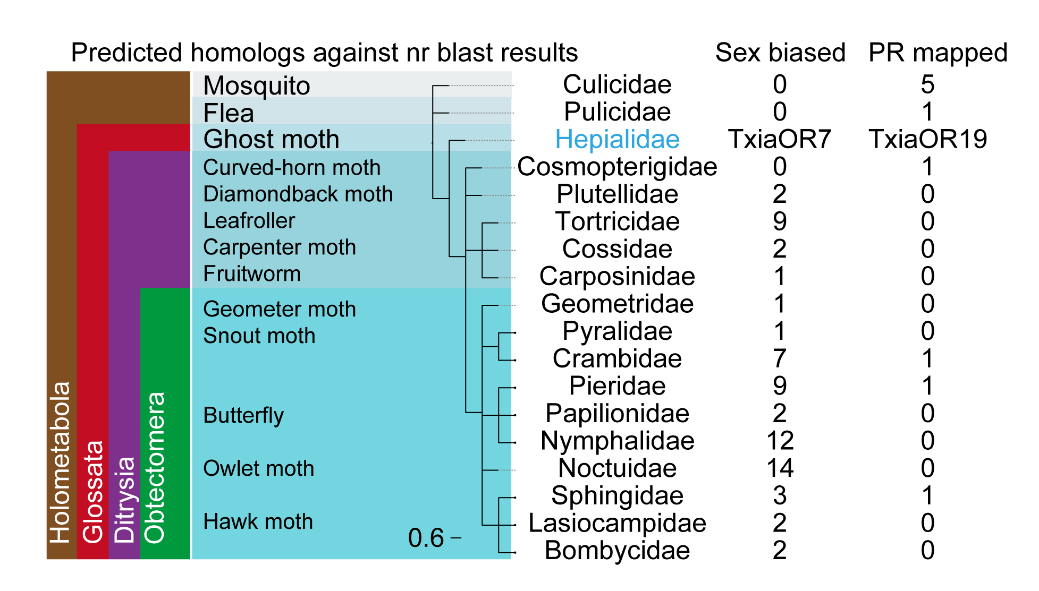


**Figure S11. Distributions of predicted TxiaOR homologs across species by nr blasting**.


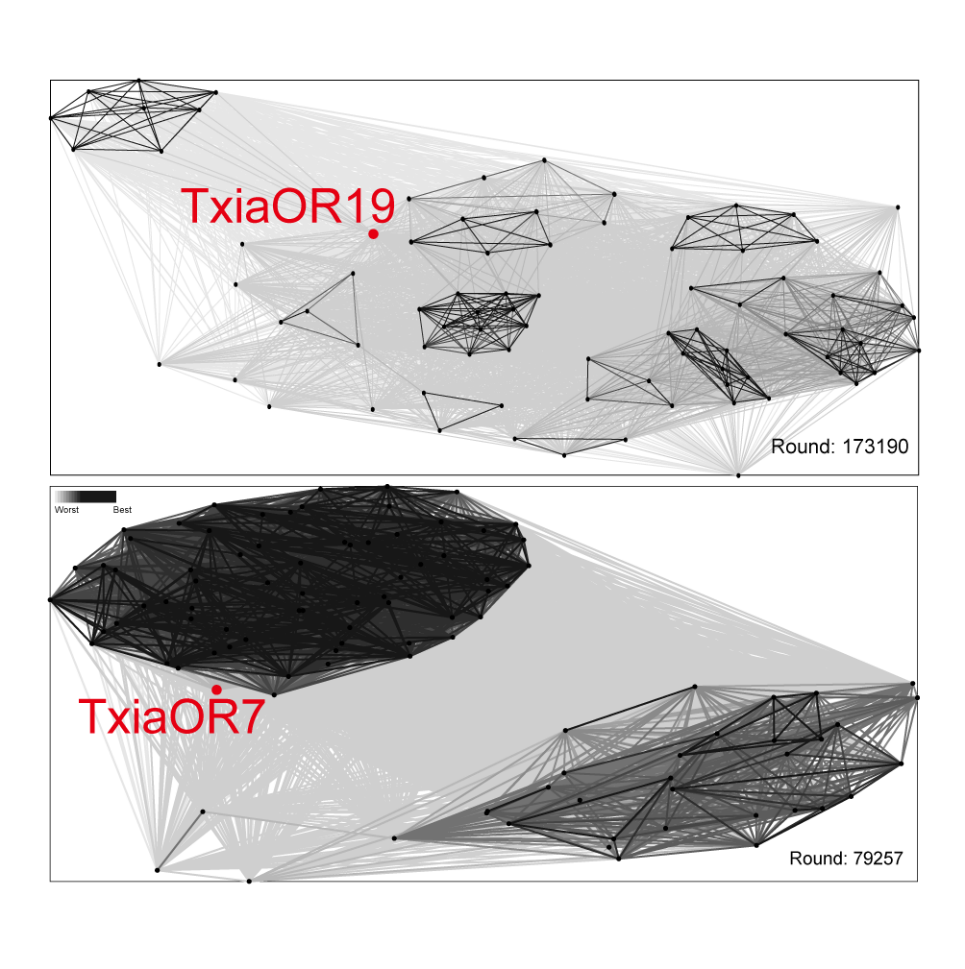


**Figure S12. Clustering of blasted ORs by TxiaOR19 lineage**. CLANS results indicate clustering of all identified ORs by blasting against nr protein library with TxiaOR19 and reference TxiaOR7, respectively. CLANS was conducted using default parameters.


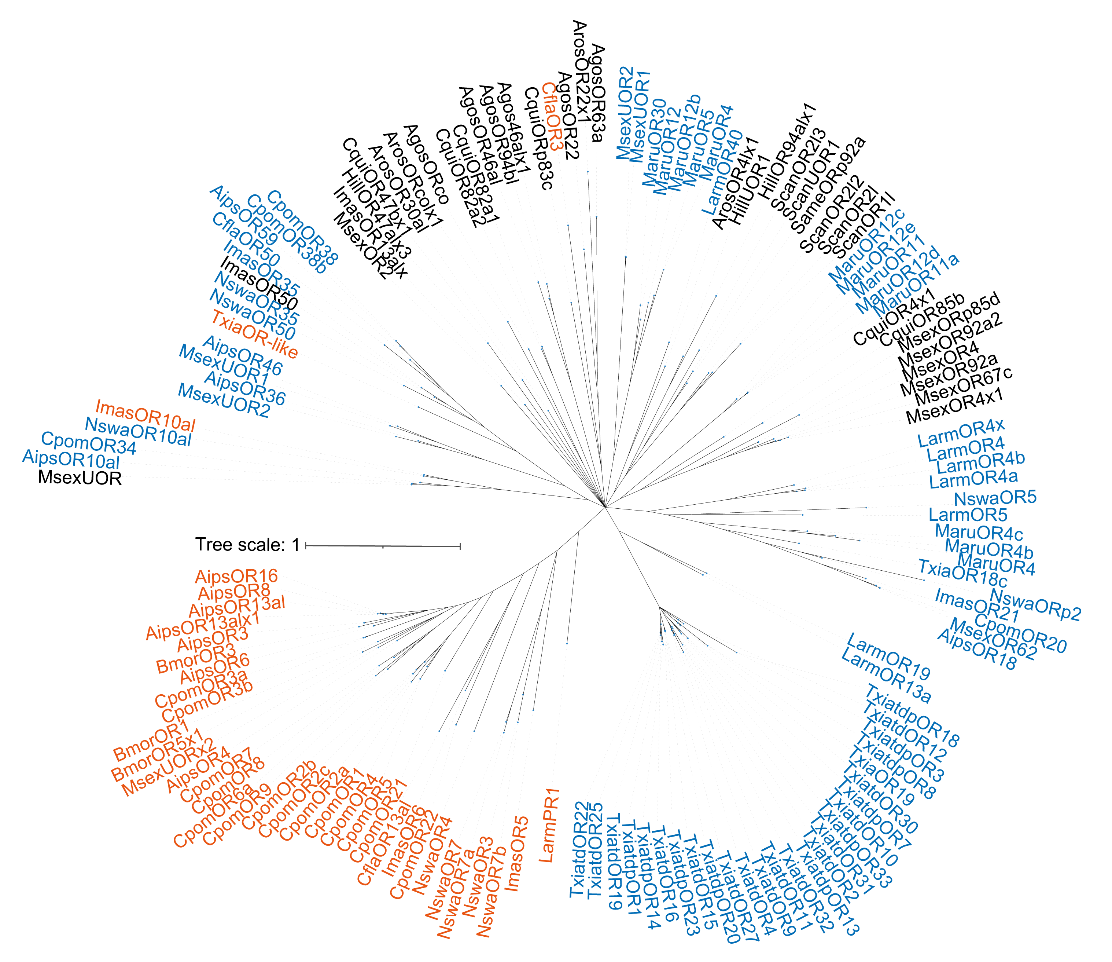


**Figure S13. Bayes tree showing evolutionary relationships of ORs mapped from linearization analysis and nr blast with TxiaOR19 tandem**. Bayesian Inference phylogenies were inferred using MrBayes 3.2.6 under JTT+F+G4 model (2 parallel runs, 200,000 generations), in which the initial 25% of sampled data were discarded as burn-in. The final average standard deviation of split frequencies was 0.069772. Color indications were used as per Figure 2B. Uncolored ORs were selected according to blastp results of TxiaOR19 array against nr database. Species included *T. xiaojinensis*, *C. pomonella*, *L. marmoratus*, *M. aruncella*, *I. masculella*, *N. swammerdamellus*, *C. flavipennella*, *M. sexta*, *B. mori*, *A. ipsilon*, *A. rosae*, *H. illucens*, *C. quinquefasciatus*, *A. gossypii*, *S. americana*, and *S. cancellata*.


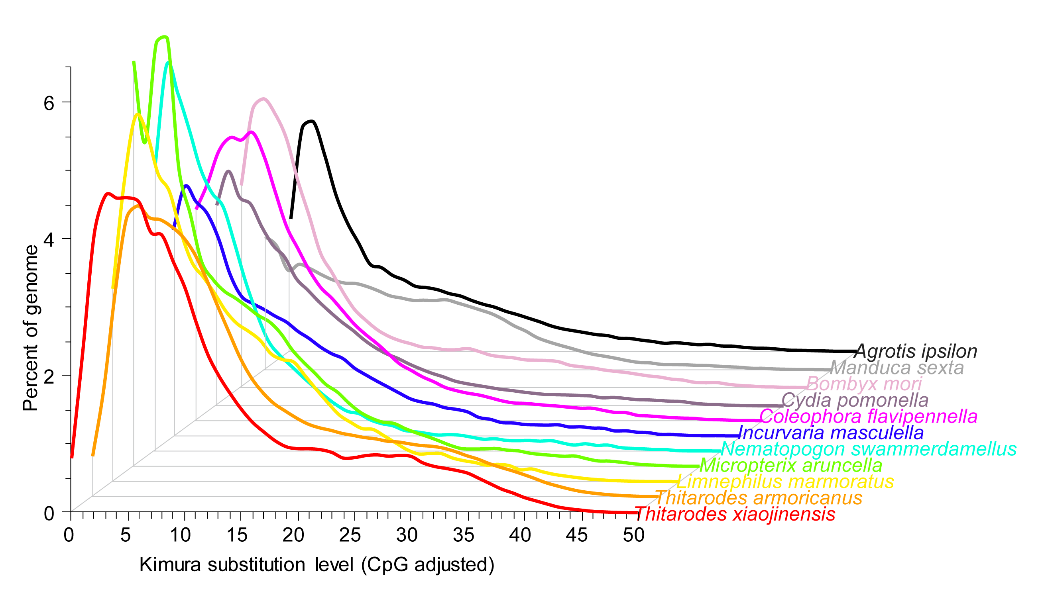


**Figure S14.** **Comparison of overall TE distributions among caddisfly and moths**.


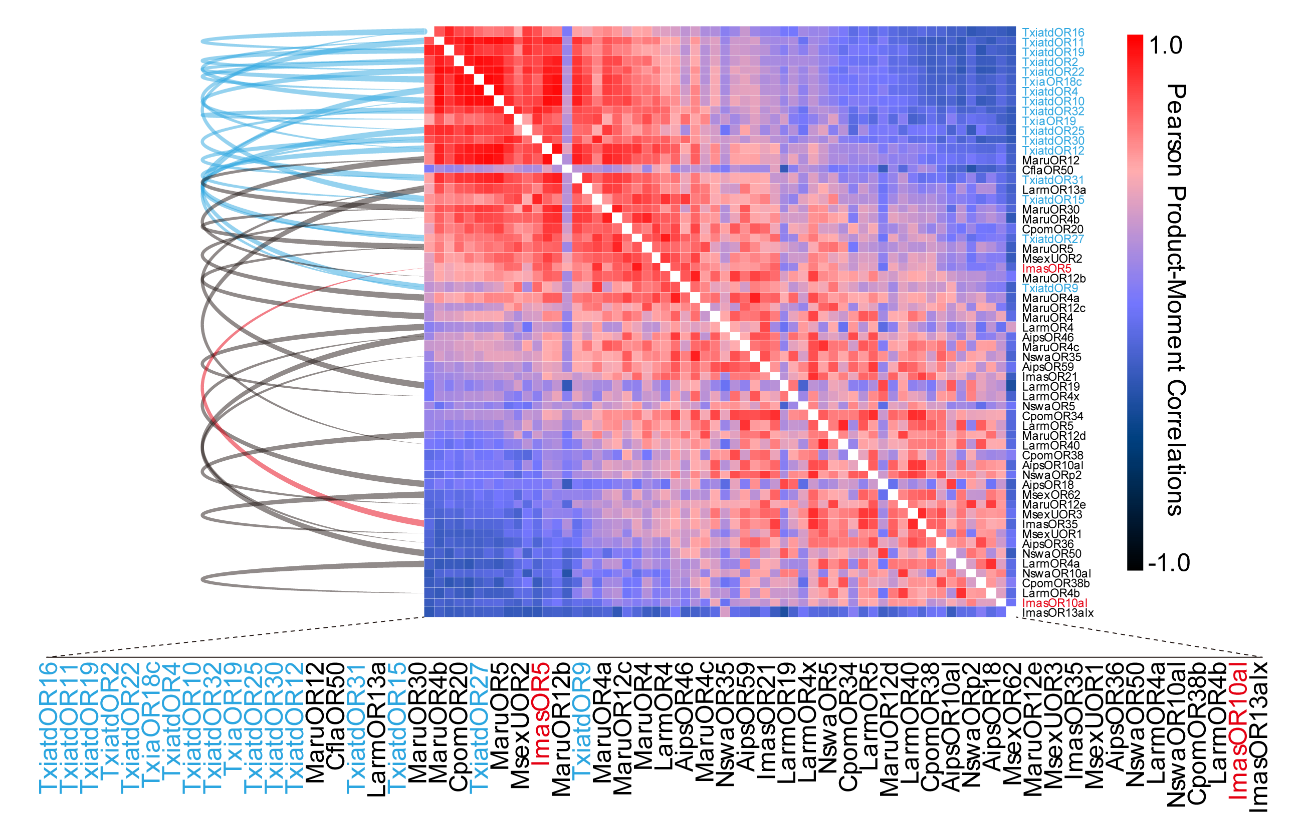


**Figure S15. Aligned Correlation matrix showing arrangement patterns of TEs in TxiaOR18c/19 mapped OR loci**. Screened regions for TEs included coding area and respective 5000 bp per up and down stream. Ribbons indicate neighboring of ORs on the same chromosome, and directions were from wider to narrower. Blue colored ORs are from *T. xiaojinensis*, and those in red are ORs mapped simultaneously by TxiaOR18c/19 and classic PRs.


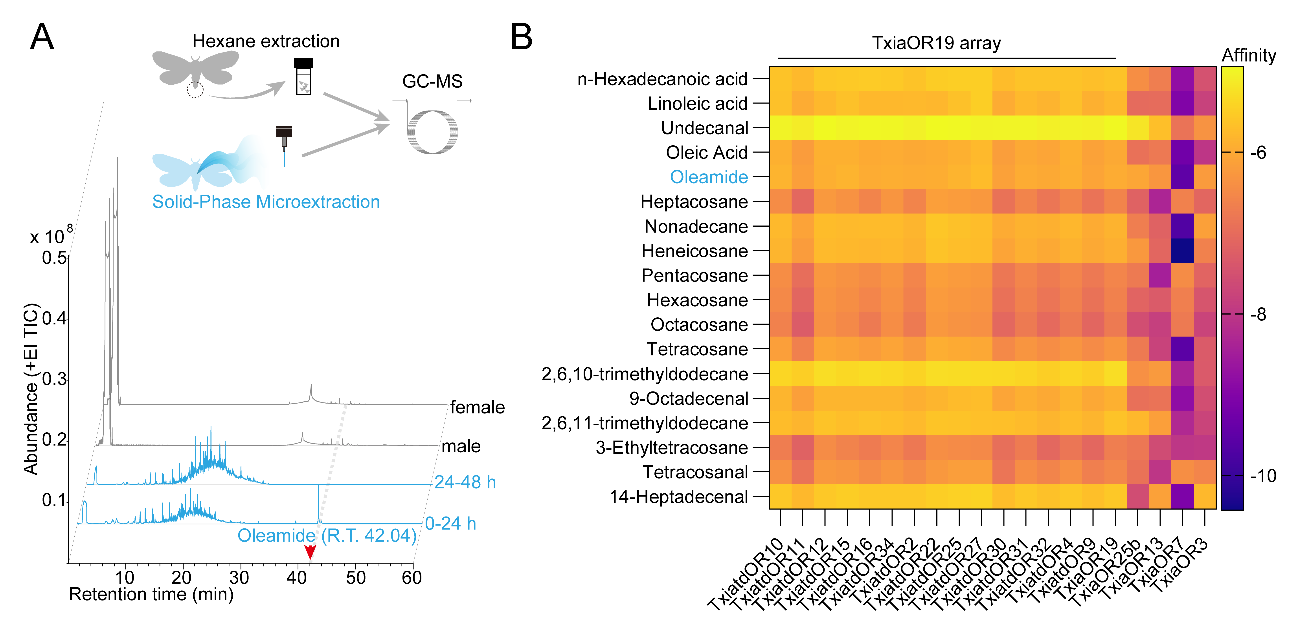


**Figure S16**. Adult emissions and predicted non-PR functioning of TxiaOR19. **(A)** GC-MS traces of abdomen tip extracts (grey) or head space volatiles (blue) from *T. xiaojinensis* adults (n = 20). Blue traces indicate chemicals collected by SPME from the same female after emerge 0 - 24 h and 24 - 48 h, respectively. **(B)** Color-coded map indicates results from docking simulations of TxiaOR19 array and the other four sex biased TxiaORs against tested ligands.

**Table S1**. Data resources for the phylogenomics and comparative genomic analysis.

| **Order** | **Family** | **Species** | **Abbreviation** | **Data Type** | **Data Resources/Accession version** |
| --- | --- | --- | --- | --- | --- |
| Lepidoptera | Erebidae | *Hyphantria cunea* | Hcun | Transcriptome | SRR6433091.1 |
| Lepidoptera | Noctuidae | *Agrotis ipsilon* | Aips | Genome | https://doi.org/10.6084/m9.figshare.16682062.v1 |
| Lepidoptera | Noctuidae | *Athetis dissimilis* | Adis | Transcriptome | Private communications |
| Lepidoptera | Noctuidae | *Mythimna separata* | Msep | Transcriptome | SRR13742171.1 |
| Lepidoptera | Noctuidae | *Spodoptera frugiperda* | Sfru | Genome | corn variant OGS6.1  (https://bipaa.genouest.org/sp/spodoptera_frugiperda_pub/) |
| Lepidoptera | Noctuidae | *Spodoptera litura* | Slit | Genome | GCF_002706865.1 |
| Lepidoptera | Noctuidae | *Helicoverpa armigera* | Harm | Genome | GCF_002156985.1 |
| Lepidoptera | Noctuidae | *Helicoverpa assulta* | Hass | Transcriptome | SRR1582443.1 |
| Lepidoptera | Pieridae | *Pieris rapae* | Prap | Genome | GCF_001856805.1 |
| Lepidoptera | Plutellidae | *Plutella xylostella* | Pxyl | Genome | GCF_000330985.1 |
| Lepidoptera | Tortricidae | *Cydia pomonella* | Cpom | Genome | http://www.insect-genome.com/cydia/ |
| Lepidoptera | Pyralidae | *Galleria mellonella* | Gmel | Genome | GCF_003640425.1 |
| Lepidoptera | Hepialidae | *Thitarodes armoricanus* | Tarm | Transcriptome | SRR12459645.1 |
| Lepidoptera | Hepialidae | *Thitarodes xiaojinensis* | Txia | Genome | GWHDOEY00000000 |
| Lepidoptera | Hepialidae | *Ahamus jianchuanensis* | Ajia | Transcriptome | Private communications |
| Lepidoptera | Noctuidae | *Spodoptera exigua* | Sexi | Genome | https://figshare.com/projects/Genome_assembly_and_annotations_  of_the_Spodoptera_exigua_beet_armyworm_/100319 |
| Coleoptera | Tenebrionidae | *Tribolium castaneum* | Tcas | Genome | GCF_000002335.3_Tcas5.2 |
| Diptera | Drosophilidae | *Drosophila melanogaster* | Dmel | Genome | GCF_000001215.4_Release_6_plus_ISO1_MT |
| Trichoptera | Limnephilidae | *Limnephilus marmoratus* | Larm | Chromosome | GCA_917880885.1 |
| Lepidoptera | Micropterigidae | *Micropterix aruncella* | Maru | Chromosome | GCA_944548615.1 |
| Lepidoptera | Incurvarioidea | *Incurvaria masculella* | Imas | Chromosome | GCA_946894095.1 |
| Lepidoptera | Incurvarioidea | *Nematopogon swammerdamellus* | Nswa | Chromosome | GCA_946902875.1 |
| Lepidoptera | Coleophoridae | *Coleophora flavipennella* | Cfla | Chromosome | GCA_947284805.1 |
| Lepidoptera | Sphingidae | *Manduca sexta* | Msex | Chromosome | GCA_014839805.1 |
| Lepidoptera | Bombycidae | *Bombyx mori* | Bmor | Chromosome | GCA_014905235.2 |
| Lepidoptera | Noctuidae | *Agrotis ipsilon* | Aips | Chromosome | GCA_028554685.1 |

**Table S2**. Comparison of chemosensory gene annotations among the tested or reported Lepidoptera.

| Species | OR | IR | GR | Reference |
| --- | --- | --- | --- | --- |
| *Ahamus jianchuanensis* | 10 | 7 | 0 | This work |
| *Thitarodes armoricanus* | 16 | 32 | 11 | This work |
| *Thitarodes xiaojinensis* | 23 | 29 | 13 | This work |
| *Plutella xylostella* | 54 | 16 | 7 | Yang et al, 2017 |
| *Cydia pomonella* | 85 | 39 | 65 | Wan et al, 2019 |
| *Pieris rapae* | 60 | 34 | 39 | Yang et al., 2021; Wang et al., 2023 |
| *Galleria mellonella* | 46 | 25 | - | Zhao et al., 2019 |
| *Hyphantria cunea* | 52 | 14 | 9 | Zhang et al., 2016 |
| *Spodoptera exigua* | 51 | 20 | 7 | Du et al., 2018 |
| *Spodoptera frugiperda* | 29 | 10 | 9 | Qiu et al., 2020; Dong et al., 2023 |
| *Spodoptera litura* | 26 | 9 | - | Feng et al., 2015 |
| *Helicoverpa armigera* | 60 | 21 | 10 | Liu et al., 2014 |
| *Helicoverpa assulta* | 44 | 24 | 18 | Xu et al., 2015 |
| *Agrotis ipsilon* | 42 | 24 | 1 | Gu et al., 2014 |
| *Mythimna separata* | 62 | 20 | 16 | Du et al., 2018 |
| *Athetis dissimilis* | 60 | 12 | 18 | Dong et al., 2016 |

-: not reported.

**Table S3**. List of PCR primers used in the study.

| Primer name | Primer sequence |
| --- | --- |
| *TxiaOr46* 5' | ATGGAACCTAAAGAAAAACAA |
| *TxiaOr46* 3' | CTATGTGGAAACCAAACTTGA |
| *TxiaOr1* 5' | ATGCTGTCGATACAGCTTGAAGAC |
| *TxiaOr1* 3' | TTAAACGTTCTTCGCGTTAACAG |
| *TxiaOr2* 5' | ATGGCGTCACTTGTTCCTTTG |
| *TxiaOr2* 3' | TCAACCCCTAGCATTAAACGT |
| *TxiaOr19* 5' | ATGACTAAAAATCTAACCAA |
| *TxiaOr19* 3' | ATTTTTCTCCCCGTTTTCTG |
| *TxiaOr13* 5' | ATGCAAGCGGTTGCCAGTC |
| *TxiaOr13* 3' | TTAGTGTTTCATGGCTGAATTGG |
| *TxiaOr12* 5' | ATGATGGAGCTCGAATCATCTGAC |
| *TxiaOr12* 3' | CTAGTGTGAGGGGTCGTACATGGT |
| *TxiaOr10* 5' | ATGACCATGGAATTCGTTGAC |
| *TxiaOr10* 3' | CTACTTAGGTTCGAAGATGGTCATA |
| *TxiaOr5* 5' | ATGGGTTTAATCAAGAATTCG |
| *TxiaOr5* 3' | CCGGCTGTTTACGAAGG |
| *TxiaOrco* 5' | ATGATGACCAAATTCAAAGTG |
| *TxiaOrco* 3' | CTTGAGTTGAATTAACACCATG |
| *TxiaOr42* 5' | ATGGAGCCCGACGGT |
| *TxiaOr42* 3' | CTAAGACTGCGATTGTATCAC |
| *TxiaOr17* 5' | ATGCAATATTTCAAAGAAGGGA |
| *TxiaOr17* 3' | CTAAGACCTTGTACTATGCAGC |
| *TxiaOr26* 5' | ATGGAAACTATACGTACGAATAGGAG |
| *TxiaOr26* 3' | TCATCATGTAGAGTATGCAGAATGG |
| *TxiaOr27* 5' | ATGACGGAGTCGGTGTACACGAGC |
| *TxiaOr27* 3' | TTCGTCTGACTCATCTGGAATGCTGT |
| *TxiaOr25a* 5' | ATGGAAACTATAAACTCTAACACTAGCTTGAA |
| *TxiaOr25a* 3' | GTGTGCTCTTCCGATCCCGTA |
| *TxiaOr11* 5' | ATGTCTGGTATGGAGAAATCAAA |
| *TxiaOr11* 3' | CTACGCCTTCCTGTTCCTTA |
| *TxiaOr43* 5' | ATGGACGCAACATCGCC |
| *TxiaOr43* 3' | TTAATCCATGCTGGTCATCAGA |
| *TxiaOr25b* 5' | ATGGAAACTATACGCACGAGC |
| *TxiaOr25b* 3' | AGATCGATCCAGCTTTGACAGTG |
| *TxiaOr8* 5' | ATGTTAAGCAAAGAAGTTAACGC |
| *TxiaOr8* 3' | TTAGTTCTCGTACATTGTGCTCAG |
| *TxiaOr7* 5' | ATGTCGCATATAGACAAAATAAAACG |
| *TxiaOr7* 3' | GCAATTCGTGTTTGTATTGGAAT |
| *TxiaOr40* 5' | ATGATATCGAACGAGTATTTTAAAA |
| *TxiaOr40* 3' | TTATTCTCTCCCGGAACTTA |
| *TxiaOr6* 5' | ATGCAGCCATACATTCCATTCAA |
| *TxiaOr6* 3' | CTAGATCTTGCTCTCGAGGAATCC |
| *TxiaOr38* 5' | ATGAATCGTGGTATAAAAGGTAATC |
| *TxiaOr38* 3' | TTATTTCATCAAACTCTGTATAACAGC |
| *TxiaOr3* 5' | ATGGATTTCAACAAAAAGTATACT |
| *TxiaOr3* 3' | TTATTTCGCTGTTAAAACTGTG |
| *TxiaOr9* 5' | ATGGACACAAACATTCCGCC |
| *TxiaOr9* 3' | CTAGTTGCTATGGACAGAGGGG |
| *TxiaOr58* 5' | ATGTACGCGAAGTCGGAGACA |
| *TxiaOr58* 3' | TTAAACGAACAAAGCGAACGAAGAG |
| *TxiaOr41* 5' | ATGGATATACCTGACCACTATTTCT |
| *TxiaOr41* 3' | TTAAGCAGTTTGATTCAACACC |
| *TxiaOr30* 5' | TGGCTGAAGCACTGTACTT |
| *TxiaOr30* 3' | TCACAGAGCAGTTTTCTTATG |
| *TxiaOr18* 5' | ATGGCTGCCGCCAACGCTTCCGGCGCCTCTCTATTG |
| *TxiaOr18* 3' | TCATATTCATATTTTACCCTTCTGCGAACCAAAAAATAA |
| *TxiaOr45* 5' | ATGGGAATGTGGCATTATTTCCT |
| *TxiaOr45* 3' | TAGTTCCTTGTTTGACTTAACATGTTGA |
| *TxiaOr60* 5' | TGGCTTGGGAAACAATTGAGGAA |
| *TxiaOr60* 3' | TAACGCATATGATGAGCAGCGATAC |
| *TxiaOr31* 5' | ATGACTCCGGTGCAGAATTG |
| *TxiaOr31* 3' | CTACGCAGCCATTTGATTCAG |
| *TxiaOr4* 5' | ATGGTTTTTCAATTCGATAAACA |
| *TxiaOr4* 3' | TCAATAGTAAATACGTCTATCCAATG |
| *TxiaOr28* 5' | ATGACAGAAGTGGTTCACTCGAGA |
| *TxiaOr28* 3' | TATTGTTCTATCTCATGGGTACTTTGC |
| *TxiaOr44* 5' | ATGTCGCATACAGGATTGTTCCTTA |
| *TxiaOr44* 3' | TTACGTAAAACTTGTCAGAAGTG |
| *TxiaOr17b* 5' | ATGCCAGAAAAGTTGGAAATAT |
| *TxiaOr17b* 3' | CTTGGTTTGTCTCAGGACTGC |
| *TxiaOr18new* 5' | ATGGCTGCCGCCAACG |
| *TxiaOr18new* 3' | TTATTTTTTGGTTCGCAGAAGGGTA |

**Supplementary material references:**

Dayhoff, M., Schwartz, R. & Orcutt, B. C. Matrices for detecting distant relationships. in *Atlas of Protein sequence and Structure* *5* (Suppl 3), 353-358 (National Biomedical Research Foundation 1978).

Dong, J.-F., Hu, Z., Dong, B.-X., Tian, C.-H. A mouthpart transcriptome for *Spodoptera frugiperda* adults: identification of candidate chemoreceptors and investigation of expression patterns. *Frontiers in Physiology* 14:1193085 (2023).

Dong, J., Song, Y., Li, W., Shi, J. & Wang, Z. Identification of putative chemosensory receptor genes from the *Athetis dissimilis* antennal transcriptome. PLoS One 11, e0147768 (2016).

Du, L. X., Liu, Y., Zhang, J., Gao, X. W., Wang, B., & Wang, G. R. Identification and characterization of chemosensory genes in the antennal transcriptome of *Spodoptera exigua*. Comparative Biochemistry and Physiology Part D: Genomics and Proteomics 27, 54-65 (2018).

Du, L., Zhao, X., Liang, X., Gao, X., Liu, Y. & Wang, G. Identification of candidate chemosensory genes in *Mythimna separata* by transcriptomic analysis. BMC Genomics 19, 1-14 (2018).

Feng, B., Lin, X., Zheng, K., Qian, K., Chang, Y. & Du, Y. Transcriptome and expression profiling analysis link patterns of gene expression to antennal responses in *Spodoptera litura*. BMC genomics 16, 1-12 (2015).

Frickey, T. & Lupas, A. CLANS: a Java application for visualizing protein families based on pairwise similarity. Bioinformatics 20(18), 3702-3704 (2004).

Gu, S. H., Sun, L., Yang, R. N., Wu, K. M., Guo, Y. Y., Li, X. C. et al. Molecular characterization and differential expression of olfactory genes in the antennae of the black cutworm moth *Agrotis ipsilon*. PloS One 9, e103420 (2014).

Kalyaanamoorthy, S., Minh, B. Q., Wong, T. K., Von Haeseler, A. & Jermiin, L. S. ModelFinder: fast model selection for accurate phylogenetic estimates. Nature Methods 14(6), 587-589 (2017).

Koh, Y. H., Park, K. C. & Boo, K. S. Antennal sensilla in adult *Helicoverpa assulta* (Lepidoptera: Noctuidae): Morphology, distribution, and ultrastructure. Annals of the Entomological Society of America 88(4), 519-530 (1995).

Kumar, S., Stecher, G., Li, M., Knyaz, C. & Tamura, K. MEGA X: Molecular Evolutionary Genetics Analysis across computing platforms. Molecular Biology and Evolution 35:1547-1549 (2018).

Liu, N. Y., Xu, W., Papanicolaou, A., Dong, S. L. & Anderson, A. Identification and characterization of three chemosensory receptor families in the cotton bollworm *Helicoverpa armigera*. BMC Genomics 15, 1-13 (2014).

Qiu, L., He, L., Tan, X., Zhang, Z., Wang, Y., Li, X. et al. Identification and phylogenetics of *Spodoptera frugiperda* chemosensory proteins based on antennal transcriptome data. Comparative Biochemistry and Physiology Part D: Genomics and Proteomics 34, 100680 (2020).

Ronquist, F., Teslenko, M., Van Der Mark, P., Ayres, D. L., Darling, A., Höhna, S. et al. MrBayes 3.2: efficient Bayesian phylogenetic inference and model choice across a large model space. Systematic Biology 61(3), 539-542 (2012).

Saitou, N. & Nei, M. The neighbor-joining method: a new method for reconstructing phylogenetic trees. Molecular Biology and Evolution 4(4), 406-425 (1987).

Wan, F., Yin, C., Tang, R., Chen, M., Wu, Q., Huang, C. et al. A chromosome-level genome assembly of *Cydia pomonella* provides insights into chemical ecology and insecticide resistance. Nature Communications 10(1), 4237 (2019).

Wang, Q., Dicke, M., Haverkamp, A. (2023) Sympatric *Pieris* butterfly species exhibit a high conservation of chemoreceptors. *Frontiers in Cellular Neuroscience* 17: 1155405 (2023).

Xu, W., Papanicolaou, A., Liu, N. Y., Dong, S. L. & Anderson, A. Chemosensory receptor genes in the Oriental tobacco budworm *Helicoverpa assulta*. *Insect Molecular Biology* 24(2), 253-263 (2015).

Yang, J., Guo, H., Jiang, N. J., Tang, R., Li, G. C., Huang, L. Q. et al. Identification of a gustatory receptor tuned to sinigrin in the cabbage butterfly *Pieris rapae*. PLoS Genetics 17(7), e1009527 (2021).

Yang, S., Cao, D., Wang, G. & Liu, Y. Identification of genes involved in chemoreception in *Plutella xyllostella* by antennal transcriptome analysis. Scientific Reports 7(1), 1-16 (2017).

Zhang, L. W., Kang, K., Jiang, S. C., Zhang, Y. N., Wang, T. T., Zhang, J. et al. Analysis of the antennal transcriptome and insights into olfactory genes in *Hyphantria cunea* (Drury). PLoS One 11(10), e0164729 (2016).

Zhao, H. X., Xiao, W. Y., Ji, C. H., Ren, Q., Xia, X. S., Zhang, X. F. et al. Candidate chemosensory genes identified from the greater wax moth, *Galleria mellonella*, through a transcriptomic analysis. Scientific Reports 9(1), 1-12 (2019).

Zhang, D., Gao, F., Jakovlić, I., Zou, H., Zhang, J., Li, W. X. et al. PhyloSuite: An integrated and scalable desktop platform for streamlined molecular sequence data management and evolutionary phylogenetics studies. Molecular Ecology Resources 20(1), 348-355 (2020).
