# Supplementary material for: A ghost moth olfactory prototype of the lepidopteran sex communication
Source: Gigascience. 2024 Jul 19;13:giae044. doi: 10.1093/gigascience/giae044 (PMC11258902; doi:10.1093/gigascience/giae044)
Supplement: giae044_Response_to_Reviewer_Comments_Original_Submission [file giae044_response_to_reviewer_comments_original_submission.pdf]

Author's Response To Reviewer Comments

Dear editor,
Thank you for considering our manuscript publish on the journal Gigascience. We have modified our manuscript very carefully based on both reviewers' suggestions, and it takes some time. All changes we have marked by highlight in the text. More details please see the manuscript or the response letter.
Best regards,
Ri-Chou Han

Reviewer reports:

Reviewer #1: The authors report on the early evolution of the olfactory system within Lepidoptera insects, framed upon the phenomena of intra-specific pheromone communication and subsequent reproductive behaviors, within three species of the primitive Exoporia lineage of Lepidoptera, compared to several other species of the more derived Ditrysian lineage of Lepidoptera. Within evolutionary contexts, olfactory organ morphology, olfactory neuroanatomy, olfactory receptor genes, transposon dynamics within genomic loci, and courtship behaviors are examined. The report is thus broad in scope, with an immense amount of data collected and examined, the methodology appears to be sound and are clearly described, however there are substantial concerns about the presentation of the results, in that the evidence to support claims being made is sometimes not clear or transparent, other times the conclusions drawn do not seem to be supported by the results presented. If these issues can be rectified, the report would provide a substantial contribution to our understanding of the early evolution of olfactory systems and olfactory-based sexual communication within Lepidoptera.
R: We thank you for detailed examinations, suggestions and pointing out our weakness in this manuscript. We, therefore, fully followed those suggestions and check our manuscript carefully, especially for conclusions. We have modified related parts of this manuscript accordingly (also marked by highlight in the text or see in below response letter). We feel that those suggestions do improve our manuscript considerably.

Specific comments are given for each section.

Abstract.
Line 37. "modern Lepidoptera".
What is a modern Lepidoptera? Is it meant to say derived (as compared to ancestral?)
R: We exchange the term "modern Lepidoptera" to the "evolutionary later Lepidoptera". And we hope the term is clearer than before.

Line 40. "Interestingly, the expanded TxiaOR19 does not function as canonical PRs".
There were no functional assays conducted on any ORs in this study. This statement should be revised.
R: We revised this sentence as "Interestingly, the expanded TxiaOr19 was predicted to have unconventional tuning patterns compared to canonical PRs".

Introduction.
Line 55-56. "In most insects, such as the vinegar fly, Drosophila melanogaster, males usually release the specific pheromone cVA to gain an advantage in recruiting females."
This sentence seems to be written incorrectly. As it is now, it is claiming that in most insects, males release cVA. Is this what is intended? Or is it meant to say that most in most insects, males release the specific (sex?) pheromone, such as is seen with Drosophila, with male release of cVA.
R: We apologize for this mistake. We rephrased these sentences as "In insects, sex pheromone becomes an effective investment for male to gain opportunities to mate with female successfully. One well studied example is that, in fruit fly Drosophila melanogaster, males typically release a specific pheromone called cis-11-vaccenyl acetate (cVA) to gain an advantage in mating".

Line 79-81. "In Lepidoptera, the antennal lobe shows obvious sexual dimorphism. The male specific MGC is located at the entry of the antenna and exclusively processes pheromone signals."

While this statement is generally true, it is not "exclusively" true. There are examples within Lepidoptera where some regions of the MGC process no pheromone signals. See Trona et al., 2010 - Journal of Experimental Biology, and Namiki et al., 2008, Journal of Comparative Physiology A; both of these report on processing of non-pheromonal odorants by the MGC in respective species.
R: We accept and adjust this sentence as "The male-specific macroglomerular complex (MGC) locates at the entry of the antenna and mainly processes pheromone signals, in addition in some species e.g. Cydia pomonella and Bombyx mori, it also responds to plant volatiles (Trona et al., 2010; Namiki et al., 2008)" and cite both publications.

Line 90-92. "Moverover, ghost moths have undergone asymmetrical divergence of duplicated genes to deliver functional alterations in subsequent species, providing insights into the evolutionary process of Lepidoptera [21]."
It is not clear whether the correct reference is being cited here, or if it is indeed the correct reference, what the claim is referring to. The cited article does not mention ghost moths nor anything about asymmetric divergence of duplicated genes. So it is not clear how this statement is supported nor how the conclusion is reached.
R: We are sorry for this citation mistake. The right citation was used in revised version as "Holland, P. W., Marlétaz, F., Maeso, I., Dunwell, T. L. & Paps, J. New genes from old: asymmetric divergence of gene duplicates and the evolution of development. Philosophical Transactions of the Royal Society B: Biological Sciences 372, 20150480 (2017)."

Line 93 (and also on 102). "epidemic".
I don't think this is the correct word, I think it is meant to say "endemic"
R: We checked our manuscript and exchange "epidemic" to "endemic".

Results.
Line 137-138. "This indicates that both male and female Himalaya ghost moths may keep pheromone reception with their shortened antennae".
Without actually testing the functional response profiles of neurons housed within the sensilla trichoidae of ghost moths, this is a speculative statement, and should not be presented in the results section, but instead in the discussion.
R: We move this sentence to the discussion part (L262-266). Please see "Interestingly, the presence of unique olfactory structures including enlarged glomeruli observed in both sexes and dominated long trichoid sensilla, mostly non-biased expressions of TxiaOr19 homologs, along with their non-feeding life traits in adulthood suggests that these ghost moths may employ a primitive pheromone sensing system to locate potential partners."

Line 140-141. "compared with other Lepidopteran moths (in general 50 to 80 glomeruli) (Figure 1E).
Where does this data come from? In addition to Figure 1E here, it is needed to refer to the relevant supplementary data file as well.
R: We have added the glomerular counts in Data S1 and referred to this file in the text.

Line 149-150. "The cumulus which represents a major area involving pheromone reception"
A reference is needed for this statement. Furthermore, is it being suggested that his area involves pheromone processing in the ghost moth? Is anything known about the pheromones used in these species? This should be described in the appropriate section of the introduction.
R: We deleted sentence of "involving pheromone reception" in results. So far, most researches about ghost moth mating or partner seeking were focus on behaviors (Kuenen et al., 1994; Allan and Wang, 2010), hence people speculate that female pheromones or male pheromone were used to attract partner in Hepialidae species. Additionally, only 3 species' male pheromones were identified including H. californicus (Kubo et al. 1985, Uchino et al. 1985), H. hecta (Schulz et al. 1990), and E. excrescens (Marukawa and Mori 2002). Hence, studies of pheromone sensing of Hepialidae species are very rare in the past. These background of ghost moth pheromone sensing was added to the manuscript. Please see "While most previous research has focused on mating behaviors and pheromone identifications in hepialids (Kuenen et al., 1994; Mallet 1984; Schulz et al., 1990; Chen et al., 2024 Journal of Economic Entomology; Allan and Wang, 2010; Kubo et al. 1985, Uchino et al. 1985; Marukawa and Mori, 2002), few studies have explored their potential pheromone-sensing neural architecture or annotated the odorant receptor family in

Hepialidae species."
In this manuscript, we first provide evidence of male and female enlarged glomeruli (Figure 1). Considering these species showed non-feeding bio traits in their adulthoods and different sex roles, we believe that those enlarge glomeruli may function as pheromone processing in hepialids. We add more discussion as "Interestingly, the presence of unique olfactory structures including enlarged glomeruli observed in both sexes and dominated long trichoid sensilla, mostly non-biased expressions of TxiaOr19 homologs, along with their non-feeding life traits in adulthood suggests that these ghost moths may employ a primitive pheromone sensing system to locate potential partners."

Line 164. "A total of 23 TxiaORs were confirmed to be expressed from the genome and transcriptomes…" Confirmed to be expressed where? In which tissues specifically?
R: The RT-PCR verification showed that 23 annotated ORs expressed in antennal cDNAs. The OR panel comprised with 35 annotations was obtained from genome and antennal transcriptome. The confirmed 23 ORs were used plus 34 TxiaOr19 tamdem and added up to 57 ORs as shown in the previous text. We agree that the sentence was confusing, so that we have revised this part to make it more clearly to readers as "A total of 23 TxiaOrs were confirmed to be expressed in the antennae of T. xiaojinensis via RT-PCR verification, out of annotations from genome and antennal transcriptome assembly". The original annotated ORs were listed in additional Data S2.

Line 168-170. "This array, homologous to TxiaOR19, contained 16 homologues (TxiatdORs) and 18 pseudogenes (TxiatdpORs), which maintained the largest tandem duplications reported in lepidopterans (Figure s5, data s2).
Figure s5 does not show an array of tandem duplications, it just shows all of the candidate genes' exon/intron structure lined up in separate rows, some of which seem to have similar patterns, but many that do not. The representations are inexplicably named "testNN" and it is not clear the relevance of these names. To make the intended point, It would be far more useful to highlight with different colors homologous exons, and show an additional panel that diagrams their relative positions in the genome on the respective chromosome. The focus on the OR19 tandem expansion of ORs is a central point in this report. So this claim needs to be properly visualized.
R: We have updated Figure S5 which included physical locations of TxiaOR19 homologs on chr14 and exon/intron structures for each homolog with homologous exons indicated by color. We also named all TxiaOR19 homologs.

Line 171-173. "The Maximum-Likelihood phylogeny analysis using 272 ORs showed that the TxiaOR19 array formed an earlier group where canonical type I PRs arose (Figure 2A, Data S3).
In looking at Figure 2A, it is not clear how well that statement is supported. The bootstrap support circle at the branchpoint between the OR19 clade and the canonical PR clade is rather small. It would be better to show the actual bootstrap support value number at this point instead of the circle representation in order to make it clearer to the reader.
R: We accept and updated the tree. The key bootstrap support values of the branch were indicated in the new Figure 2A. The relevant methods, results, and figure legends were revised.

Line 173-175. "This tree topology between the two clades was consistent when cross checked with both Neighbor-Joining and Bayesian methods (Figure s6).
This is true, but those trees do not appear to clearly support such close relationships between the PR clade and the OR19 clade. How can these differences be reconciled? In Figure 2A the relationship between the two clades appears to be directly approximate, but in the Figure s6 trees, they do not.
R: We re-built the NJ and BY tree as the ML tree was updated, using the same batch of 387 ORs. Please see the updated Figure 2A and Figure S9. To make it clearer, we modified related sentence as "The Maximum-Likelihood phylogeny analysis using 387 ORs showed that the TxiaOr19 array joint an earlier group where canonical type I PRs arose (Figure 2A, Data S3). The earlier separation of canonical PRs and TxiaOR19 tandem was also observed when cross-checked with Neighbor-Joining and Bayes method (Figure S9)."

Line 176-178. "The TxiaOR19 array suggested an earlier emergence than male-biased TxiaOR7, as it could blast to ORs in locust, aphid, soldier flu, mosquito and flea, with homologues predicted by CLANS in mosquito and flea (Figure 2C, Figure s7).

It is not clear how Figures 2C and S7 supports the claim that any TxiaORs have any homology with ORs from mosquito and flea, and it is not clear from figure 2C what data supports this claim. It would have been better to include these ORs (from mosquito and flea) in figure 2A in order to provide better support for homologous relationships between ORs across distant insect orders. It is well known that there is little evidence for homology between ORs across distant insect orders, See Hansson and Stensmyr 2011 review in Neuron, Figure 3. Solid evidence would be needed to be shown to support the claim being made, and as currently presented, that evidence is lacking.
R: We revised this description, and updated the phylogeny tree with adding ORs from mosquito and flea by blasting using TxiaOR19 in the new Figure 2A. Please see "Differing from ORs found in evolutionarily later moths, the TxiaOR19 array could blast to ORs in locusts, aphids, soldier flies, mosquitoes, and fleas, with homologues predicted by CLAN [37] (Figure 2A and Figure S9). However, the male-biased TxiaOR7 failed to blast to any ORs from those earlier species (Figure S11, S12)."

Line 183-185. "suggesting possible evolutionary patterns similar to the duplicated zen family of orange swift moth, H. sylvina, which diverged to new functional gene families Shx"
This is a discussion point that should not be presented as results.
R: We delete this sentence in the results.

Line 185-186. "On the other hand, PRs could be traced back to a single LarmPR1 in the caddisfly."
What is this claim based on? It seems like it is being made from Figure 2D. However, it is not clear that anything shown in Figure 2D supports the claim. LarmPR1 does not seem to be connected or tracing to any other ORs in that figure.
R: We apologize for this unclearly claim and we updated Figure 2A and marked the caddisfly PR. The sentence was revised as "it showed that canonical PRs and a single LarmPR1 in the caddisfly formed a clade in the phylogeny".

Line 190-191. "Specifically, TxiaOR19 array arose from the LarmOR19-OR13a tandem (Figure 2E)."
What is the statistical support (Bootstrap values or Likelihood Ratio or other) for this claim? It does not seem to be apparent in the figure, so it is difficult to assess how well supported the phylogenetic relationships are.
R: We revised this sentence to "Specifically, TxiaOr19 array formed the same clade with LarmOr19-Or13a tandem". Please see Figure 2A.

Line 241-243. "which fits the predicted non-PR functioning of TxiaOR19 to a non-canonical female emission and the olfactory architectural observations."
What functional assays were done to show that TxiaOR19 does or does not function as a PR? Is this based on the molecular docking predictions shown in figure S8? If that is the case, it appears in that figure that the male-biased OR7 shows higher affinity to the female-specific emitted compound, Oleamide. This would seem to point to the potential for OR7 being a PR. How can the claim made here be reconciled with what is shown in Figure S8?
R: We agree that TxiaOR7 in the docking simulation could be a better hit for PRs, but we did not map this OR using canonical PRs along with the TxiaOR19. This TxiaOR7 may support the recent reports of the novel PR clade in lepidopteran species and worth further investigation. As we here in this work focus on the canonical PR clade, we revised this sentence as "Our results indicated that the sex roles of T. xiaojinensis adults were different from those of higher lepidopterans during mating allocation, which could relate to predicted unconventional TxiaOR19 tandem, the non-canonical female emission and the olfactory architectural observations." And also in the discussion "One interesting result in our molecular docking predictions is that the male-biased OR7 shows a higher affinity to oleamide, suggesting that TxiaOR7 potentially serves as an ancestral PR of the novel PR clade in lepidopteran species. However, more experimental functional evidence needs to be provided for both TxiaOR19 and TxiaOR7 to support their evolutionary roles in lepidopteran species."

Discussion
Line 251-253. "We have also discovered that both males and females of these moths possess a compact olfactory system with distinct structures involved in sexual recognition"
What is the basis for this claim? Which structures? It was not shown that any structures are involved in sexual recognition in the ghost moths examined in this report. I would suggest to be careful about making

assumptions about homologous structures from higher moths while at the same time trying to argue in favor of great distinctions in the ancestral ghost moths compared to the higher moths. It is better to present data about the function of the structures of the ghost moth instead.
R: We accept and rephrase the sentence as "Interestingly, the presence of unique olfactory structures including enlarged glomeruli observed in both sexes and dominated long trichoid sensilla, mostly non-biased expressions of TxiaOr19 homologs, along with their non-feeding life traits in adulthood suggests that these ghost moths may employ a primitive pheromone sensing system to locate potential partners."

Line 266-267. "OR19 duplications in these ghost moths do not appear to have enhanced functions"
What does this mean? Almost no evidence was presented demonstrating the function of these ORs.
R: Although we tried to deorphanize the TxiaOR19 by using molecular docking software, but it is still hard to show TxiaOR19' functions. Hence, we toned down OR function in the manuscript and delete this sentence in here.

Line 294. "These cues also suggest the ancestral roles of the ghost moths in terms of their sex role systems."
It is not clear what is meant by this.
R: We rephrase this sentence as "Given their ecological traits, highly redundant genome, and a smaller number of ORs, we believe that these Himalayan ghost moths may retain pheromone sensing abilities and exhibit primitive sex roles within Lepidoptera. Further investigation into the function of ORs remains crucial for refining this perspective."

Figures and Data

Figure S8. "or head space volatiles (blue)"
Are these blue traces both from females, or something else? It should be clarified in the legend.
R: We added the information in the legend.

Table S4. Concerning Cydia pomonella, Wan et al., 2019 reported 85 ORs, 65 GRs and 39 IRs (see page 4), so it is not clear what is being referred to here for 82 ORs, 15 IRs and 1 GR.
R: We apologize for this mistake. We then check all data in the Table S4 (the Table S2 in revision files) and we hope it is clearer now.

Reviewer #2: The manuscript entitled "A ghost moth olfactory prototype of the lepidopteran sex communication" is reviewed. This is a highly multidisciplinary work and difficult for me (and maybe to many other researchers) to review. Based on my expertise of insect genomics and molecular adaptation, I am providing my comments mainly on the related sections (i.e. genome and transcriptome assembly, orthology analyses, and gene evolution). In general, this is publishable material from my point of view, with some revision required to improve the manuscript. Here I list two of my major concerns and some minor suggestions.
R: We appreciate your thorough examination and valuable suggestions. We have carefully incorporated all your recommendations into our manuscript and figures. In the relevant sections of the manuscript, we have highlighted the changes in yellow. We believe that these modifications have enhanced the clarity and readability of the manuscript compared to the previous version.

Main concerns:
1. For the sensilla distribution analysis (Figure 1D), I feel the result is potentially biased by the different ways of counting the sensilla trichoidae in the retrieved studies. For example, Gargi et al. were counting all the sensilla trichoidae on the entire antenna, while the authors possibly divided the number by number of flagellum segments to represent the "mean" number of sensilla trichoidae of each flagellum to make it comparable with the data matrix generated in the current study. But this is assuming that all the flagellum segments have similar number of sensilla trichoidae, which is unlikely to be true. Ideally, counting all the sensilla trichoidae is required to perform the statistic analyses. My suggestion is to not include this section in the manuscript as it is not necessary for authors to derive their conclusions.
R: We agree. The statistics for sensilla tricoidae was removed from the manuscript and we toned down related descriptions about sensilla.

2. The completeness assessment of the genome assembly of T. armoricanus is not available. So does the transcriptome of A. jianchuanensis. These are very crucial as if the assembly less complete, the subsequent analyses will be confounded. My suggestion is to run BUSCO on the genome and transcriptome assembles.
R: We added BUSCO results for both T. armoricanus genome and A. jianchuanensis transcriptome in the manuscript. Please see "BUSCO analysis indicates 90% of single-copy insect orthologs are complete. We also conducted BUSCO analysis towards the transcriptomes of A. jianchuanensis, and a completeness of 96.6% was observed".

3. I think the figures are too complicated for most readers. Too packed figures make it unpleasant to read but the role of the figures should be helping readers to quickly get the key information. For example, Figure 1B, C, D, F, G, and H; Figure 2B, C, E, H, and G; Figure 3B and C, can be separated figures in SI.
R: We agree that visualization concerns and have followed suggestions. We, therefore, re-organized the figure panels, by moving Figure 1C, F, G, 2B, C, and 3B, C to the supplementary materials. The phylogenic tree was updated combining previous Figure 2A and D.

Minor suggestions:
1. Line 112 (results): I was expecting to see the assembly report of T. armoricanus as well, but it seems missing in the result sections.
R: We added assembly report of T. armoricanus in revised "Genome of T. armoricanus was sequenced on Illumina HiSq2000, harvesting 877.7 Gb clean data to construct scaffolds. The final assembly presented 3,168 Mb total length of the scaffolds, with N50 of 27.8 kb and 176.2 kb for contigs and scaffolds, respectively. BUSCO analysis indicates 90% of single-copy insect orthologs are complete. We also conducted BUSCO analysis towards the transcriptomes of A. jianchuanensis, and a completeness of 96.6% was observed. Genome and transcriptomes were subsequently employed in downstream analysis."

2. Line 136: maybe address why H. cunea was selected for the comparison.
R: This comparison was removed according to Major comment 1. H. cunea is a signature species which presents significant sex dimorphism of the antennae among the tested species, so that we used this species as a calibrator to assess sensillar counts in T. xiaojinensis. Due to the counting method limitations raised in Major comment 1, we removed this part.

3. Line 165: not sure what the "57 annotations" means here.
R: We are sorry for this unclear description. The relevant part was revised and additional supplementary file was added. "A total of 23 TxiaOrs were confirmed to be expressed in the antennae of T. xiaojinensis via RT-PCR verification, out of annotations from genome and antennal transcriptome assembly" (Data S2 listed annotated TxiaORs for used in PCR verifications)

4. Line 167: what is LG14?
R: We revised LG14 to chr14.

5. Line359-360: please describe how the short read sequences were generated
R: We revised this part as "Meanwhile, short-reads library was constructed by Illumina platform with the same batch of T. xiaojinensis DNA, and 165 Gb raw data were generated. After filtering, the remaining clean reads with Q > 20 were used for minimap2 mapping onto the genome assembly which was later polished by NextPolish (https://github.com/Nextomics)."

6. Line 361-362: please provide more information about how the non-insect contigs were identified.
R: We added relevant information to this part. Please see "To remove the DNA pollutions from the other organisms, the polished genome was aligned against the NCBI nucleotide (NT) database, and the contigs which were aligned to the sequences from fungi, plants, or virus were removed."

7. Line384: not sure what it means by "second generation genome" since there is no previous version of genome assembly for the species.
R: We are sorry for the confusion description. The relevant part was revised as "Genome".

8. Line 406-407: What I know about orthofinder is to get the orthologs from protein sequences. Here it

says "the orthologous genes of these 18 insect species were inferred from their genomes or transcriptomes" but those are DNA sequences not proteins.
R: We are sorry for the mistake. The orthofinder does refer to protein sequences. We revised this part.

9. Line425: I thought this should be protein-protein searching, but the queries are DNA, this is somewhat confusing me.
R: We apologize. We revised this part.

10. Line430: please provide more information about how the ORs were verified by PCRs.
R: The detailed methods were described in “Characterizations of Ors” section following this part. So that we removed this sentence in order not to draw any confusion.
