# Supplementary material for: A ghost moth olfactory prototype of the lepidopteran sex communication
Source: Gigascience. 2024 Jul 19;13:giae044. doi: 10.1093/gigascience/giae044 (PMC11258902; doi:10.1093/gigascience/giae044)
Supplement: giae044_Response_to_Reviewer_Comments_Revision_1 [file giae044_response_to_reviewer_comments_revision_1.pdf]

Author's Response To Reviewer Comments

GIGA-D-23-00252R1
A ghost moth olfactory prototype of the lepidopteran sex communication
Rui Tang; Cong Huang; Jun Yang; Zhong-Chen Rao; Li Cao; Peng-Hua Bai; Xin-Cheng Zhao; Jun-Feng Dong; Xi-Zhong Yan; Fang-Hao Wan; Nan-Ji Jiang; Ri-Chou Han
GigaScience

Dear Prof Han,

Your manuscript "A ghost moth olfactory prototype of the lepidopteran sex communication" (GIGA-D-23-00252R1) has been assessed by our reviewers. Based on these reports, and my own assessment as Editor, I am pleased to inform you that it is potentially acceptable for publication in GigaScience, once you have carried out some essential revisions suggested by our reviewers.

Their reports, together with any other comments, are below. Please also take a moment to check our website at https://www.editorialmanager.com/giga/ for any additional comments that were saved as attachments.

Once you have made the necessary corrections, please submit a revised manuscript online at:

https://www.editorialmanager.com/giga/

If you have forgotten your username or password please use the "Send Login Details" link to get your login information. For security reasons, your password will be reset.

Please include a point-by-point within the 'Response to Reviewers' box in the submission system. Please ensure you describe additional experiments that were carried out and include a detailed rebuttal of any criticisms or requested revisions that you disagreed with. Please also ensure that your revised manuscript conforms to the journal style, which can be found in the Instructions for Authors on the journal homepage. On top of these, we suggest you find a copy editing company or friendly native English speaker to polish the grammar.

The due date for submitting the revised version of your article is 04 Aug 2024.

We look forward to receiving your revised manuscript soon.

Best wishes,

Hongfang Zhang
GigaScience
www.gigasciencejournal.com

Dear editor,
Thank you for considering publishing our manuscript in Gigascience. We have carefully revised our manuscript based on all suggestions from the reviewer and have answered the reviewer’s questions point-by-point in the following letter. Furthermore, we have edited the language and fixed some grammatical errors, and we believe the current version is more readable. All changes have been marked by highlighting them (in green) in the text in the R2 submission files. We truly hope that the R2 revised manuscript is in good format and scientific quality to meet the journal's publishing standards. For more details, please see the manuscript or the response letter.
Best regards,
Ri-Chou Han

Reviewer reports:

Reviewer #1: The manuscript is substantially improved after the previous round of revisions and most of the reviewer comments have been addressed. However, reading the updated manuscript in this new light has brought to the forefront several other minor concerns that must be addressed before the manuscript should be published. Furthermore, while the manuscript is generally written very well, numerous small language errors are noticeable throughout the text. It is recommended to ensure the manuscript is revised for language after all scientific edits are made.

R: We appreciate your detailed assessments and suggestions. We followed each of the suggestions and revised our manuscript carefully. Accordingly, we have modified related parts in the manuscript (please see the green highlights in the text or in the response letter below). After addressing these concerns and fixing grammatical errors, we feel that your suggestions have indeed helped us improve the quality of this manuscript and make it more readable.

Minor revisions are suggested as follows:
Introduction
Line 94-96. "Moreover, ghost moths have undergone asymmetrical divergence of duplicated genes to deliver functional alterations in subsequent species, proving insights into the evolutionary process of Lepidoptera."
Which genes? Is this a widespread phenomena across all different kinds of genes, or is this referring to specific genes or gene families? Please clarify.

R: The duplication genes mentioned in here are zen gene. And the asymmetrical divergence is a widespread phenomenon in all genes during evolution. In the manuscript, we clarified as "Moreover, ghost moths have undergone asymmetrical divergence of duplicated gene, e.g. zen gene, to deliver functional alterations in subsequent species, providing insights into the evolutionary process of Lepidoptera".

Results
Line 211-212. "In all, the genomic backgrounds and receptor repertoires are specific in T. xiaojinensis, which may confer to the structural specificities described above."
It is not clear what this statement means. How are these features specific in T. xia, and what structural specificities are being referred to.

R: We deleted this statement, as it is confusing in the current version of manuscript since we removed some functional observations from the text.

Line 234-235. "possible functional drift of TxiaOR19."
Please describe what is meant here more clearly with this term. Functional drift in what manner, or relative to what?

R: We delete the term "functional drift" and change the sentence as "The replacement of the TxiaOR19 duplications with canonical PRs suggests possible different tuning characteristics of TxiaOR19 from a PR that narrowly tunes to sex pheromone components"

Line 241-244. "The results showed that TxiaOR19 had less binding affinity towards the panel of the ghost moth emissions, and its responding spectrum were relatively broad, indicating that ghost moth T. xiaojinensis may show unconventional courtship behaviors comparing to higher moths."
Might this result also suggest that other ORs, especially within the OR19 tandem array may be instead tuned to ghost moth emissions? Why aren't docking profiles examined for other OR19 sub-family receptors? In any case, this kind of statement seems more like a discussion statement than a results statement. I understand it is used to transition to the next paragraph, however, the logic for looking at behavior in males and females (as it turns out, non-dimorphic behavior), could just as well follow from the lack of sexual dimorphism in the antennal lobe, perhaps much better, as compared to the hypothetical ligand docking studies, which are far from complete.

R: We redocked all TxiaOR19 array ORs to ligands, and the results showed that all tested TxiaORs are not specifically tuned to the female-specific compound oleamide (see Figure S16), which supports our idea that the TxiaOR19 array has different tuning characteristics compared to the narrowly tuned PRs. In the

manuscript, we have toned down our statements regarding OR functional docking, as this aspect is not our main focus in discussing the evolution of moth pheromone receptors. Please see the following statement: “The results showed that the TxiaOR19 array had less binding affinity towards the panel of ghost moth emissions, and the responding spectrum was relatively broad for all 16 tandem ORs.”

Regarding the transition from molecular to behavioral aspects in the manuscript, we followed your suggestion by initially discussing the lack of sexual dimorphism in the antennal lobe before transitioning to the discussion of non-dimorphic behavior. See the “Considering ghost moths, including T. xiaojinensis, lack sexual dimorphism in their antennal lobe (Figure 1D), we hypothesized that T. xiaojinensis may also exhibit unconventional calling and mating behaviors compared to higher moths.”

Discussion.
Line 256. "Himalaya ghost moths offer a basal model for the study of olfaction evolution in insects"
I am not sure it is correct to say "insects" here. It may be more correct instead to say "Lepidoptera" or at the very most, the "Lepidoptera/Trichoptera" sister clades, since caddisflies are also included….but not more generally insects.

R: We adjusted “insect” to “Lepidoptera”.

Line 278-279. "However, more experimental functional evidence needs to be provided for both TxiaOR19 and TxiaOR7…"
Again, here, shouldn't this refer to the TxiaOR19 sub-family/array ORs more generally, instead of only TxiaOR19?

R: We re-docked the TxiaOR array to ligands, please see the Figure S16. And we also adjust the “TxiaOR19” to “TxiaOR19 array” in the manuscript.

Line 282. "the TxiaOR19 array and LarmPR1 share motifs that reflect some exons on the loci"
It's not clear what this means. How do the motifs reflect exons? Which exons and which loci?

R: We removed “that reflect some exons on the loci” as there was no hard evidence to support this statement.

Line 282-283. "These motifs were separated before the evolution of Lepidoptera."
This is a vague statement. Do you mean before Lepidoptera and Trichoptera diverged from each other, or before the ghost moths split off from the more recently evolved moths? Please clarify.

R: We apologize for this vague statement. We clarified as “These motifs were likely to be already separated before Lepidoptera and Trichoptera diverged from each other”.

Line 286-287. "On the other hand, the shared motif regions could be traced back to earlier dipteran species"
Which shared motif regions? Are you referring to the ones in the PRs as shown in Fig 2C/2D/2E? Or motifs present in other organisms, as mentioned in the previous sentence?

R: We hope to compare the motifs of PRs to the TxiaOR19. We revised this sentence as “On the other hand, PRs shared motif regions with TxiaOR19 and the later could be blasted to ORs in dipteran species where large OR duplications existed such as in Bactrocera dorsalis”.

Line 297-298. "Enlarged MGCs in butterfly Piers rapae suggest that the sexual dimorphic sex pheromone recognition system is widely used by Lepidoptera"
Please check the species, as Piers rapae is not at all mentioned in the cited article, no. 49. I would also further caution against making this statement about the sexually dimorphic sex pheromone recognition system, as the authors of the cited article emphasize that their butterfly MGC is not implied to be homologous to the moth MGC.

R: We apologize for the inconsistent citation and unclearly statement. We revised this sentence as “The

enlarged MGCs in the butterflies, such as Pieris rapae and Godyris zavaleta [49], suggest that the sexual dimorphic brain organizations are widely presented by Lepidoptera, however, the link between morphology and pheromone recognition in butterflies requires further examination".

Materials and Methods.
Section on Scanning electron Microscopy.
For the washes, dehydration and drying steps, please specify the length of times for each of these steps.
R: We revised the paragraph by adding these essential information as "The antennae of 1-3 d adults were cut from base and fixed in 0.25% glutaraldehyde at 4 °C overnight. After three washes at room temperature with 0.1 M phosphate-buffered saline (PBS, pH 7.4) for 15 min each, antennae were dehydrated through a laddered ethanol series (30, 50, 70, 80, 90, and 100%) for 15 min each and dried for 15 min in a critical point drier (Bal-Tel CPD 030) before mounted on aluminum stubs. The mounted antennae were coated with gold spray (Bal-Tel SCD 005) and observed with SEM instrument (FEI Quanta 200,)."

Line 376. Change pollutions to contamination.
R: Revised.

Figures and Tables
Figure 2, Panels C-E. It is unclear why Motifs 1-3 are shown in 2C, while Motifs 4-6 are shown in 2D. And in 2E, motif 2 appears very similar to Motif 4, while Motif 3 appears very similar to Motifs 5 and 6. Based on this last point, the text from lines 204-211 is confusing, especially considering that in 2C and 2D, for the PR consensus representations, either Motifs 1-3 are shown in 2C or Motifs 4-6 are shown in 2D. If this is the PR consensus, and it is the same representation for 2C and 2D, shouldn't Motifs 1-6 be shown for both? Please try and re-write the relevant text in the main manuscript and figure legend so that this concept is clearer.

R: The Motifs 1-3 are highly conserved motifs of PRs, reviewed by Zhang and Löfstedt in 2015 (doi.org/10.3389/fevo.2015.00105). We firstly used the MEME suite to check the signature Motifs of the PR clade, and confirmed them according to published works. The same approach was used then to test TxiaOR19 mapped ORs, and we thus named the later motifs as Motif 4-6. As the two clades were separately investigated, they had some overlaps but should not be placed together. We are sorry for this mistake and removed PR consensus from Figure 2D in order not to draw any confusions. The descriptions in the manuscript as "Using MEME suite, we found that LarmPR1 exhibited all three signature motifs of PR consensus regions as reported before" and also as "On the other hand, the majority of TxiaOR19-mapped ORs had two motifs, with some having three motifs (named as motif 4-6) upon checking with the same approach".

Figure 3, Panel A. It is not clear where the burst events are. Are they marked by the red arrowheads? This should be written out clearly in the figure legend.
R: The red arrows indicate TE burst. We have added this information in the figure legend of Figure 3. See "Red arrowheads indicate TE burst events"

Figure S2. What do the black squares and grey bars represent? Please indicate their meaning in the figure legend.
R: We added explanations in the figure legend. Please see "Where square dots indicate each measurement of individual sample, grey bars indicate the ranges of O.I. from the species"

Figure S3. It appears that different sensilla types are present in the various different panels. Please indicate, with arrows and notations, in each panel, which sensilla type are the sensilla trichodea, as it is not obvious to an unfamiliar reader.
R: We added arrowheads to indicate sensilla trichodae in each panel, with figure legend revised "Arrowheads indicate representative sensilla trichodae morphology in each panel".

Figure S8. It appears that only 18 genes are shown here. It should be made clear in the figure legend that these are the intact homologues and the pseudogenes are excluded, if that is indeed the case.
R: Yes, it is the case. We added relevant clarification to the figure legend as "Tandem TxiaOr19 intact Or

duplications exclude pseudogenes”.

Table S2. The title of this table has been updated to say "comparison of chemosensory genes in head among the tested Lepidoptera. Is this referring to head only? Or is it an indication of head/antennal expression? Furthermore, in the previous round of revision, it was mentioned that the numbers for C. pomonella were inaccurate and they have been updated to reflect all ORs/GRs/IRs identified in the genome, as reported by Wan et al., 2019. However, now that the title has changed to reflect chemosensory genes in head, the numbers for C. pomonella are again inaccurate, as they reflect presence of these genes in the genome, not in the head.
R: We are sorry for this. The correct title for this table is revised to “Table S2. Comparison of chemosensory gene annotations among the tested or reported Lepidoptera.”

Reviewer #2: My comments to the manuscript have been properly addressed. I have no further concerns.
R: Thank you again for your time.
