# Supplementary material for: A ghost moth olfactory prototype of the lepidopteran sex communication
Source: Gigascience. 2024 Jul 19;13:giae044. doi: 10.1093/gigascience/giae044 (PMC11258902; doi:10.1093/gigascience/giae044)
Supplement: giae044_Reviewer_1_Report_Original_Submission — William B. Walker, Ph.D. -- 10/6/2023 Reviewed [file giae044_reviewer_1_report_original_submission.pdf]

Reviewer Report

Title: A ghost moth olfactory prototype of the lepidopteran sex communication

Version: Original Submission **Date:** 10/6/2023

Reviewer name: William B. Walker, Ph.D.

Reviewer Comments to Author:

The authors report on the early evolution of the olfactory system within Lepidoptera insects, framed upon the phenomena of intra-specific pheromone communication and subsequent reproductive behaviors, within three species of the primitive Exoporia lineage of Lepidoptera, compared to several other species of the more derived Ditrysian lineage of Lepidoptera. Within evolutionary contexts, olfactory organ morphology, olfactory neuroanatomy, olfactory receptor genes, transposon dynamics within genomic loci, and courtship behaviors are examined. The report is thus broad in scope, with an immense amount of data collected and examined, the methodology appears to be sound and are clearly described, however there are substantial concerns about the presentation of the results, in that the evidence to support claims being made is sometimes not clear or transparent, other times the conclusions drawn do not seem to be supported by the results presented. If these issues can be rectified, the report would provide a substantial contribution to our understanding of the early evolution of olfactory systems and olfactory-based sexual communication within Lepidoptera.
Specific comments are given for each section.
Abstract.
Line 37. "modern Lepidoptera".
What is a modern Lepidoptera? Is it meant to say derived (as compared to ancestral?)
Line 40. "Interestingly, the expanded TxiaOR19 does not function as canonical PRs".
There were no functional assays conducted on any ORs in this study. This statement should be revised.
Introduction.
Line 55-56. "In most insects, such as the vinegar fly, Drosophila melanogaster, males usually release the specific pheromone cVA to gain an advantage in recruiting females."
This sentence seems to be written incorrectly. As it is now, it is claiming that in most insects, males release cVA. Is this what is intended? Or is it meant to say that most in most insects, males release the specific (sex?) pheromone, such as is seen with Drosophila, with male release of cVA.
Line 79-81. "In Lepidoptera, the antennal lobe shows obvious sexual dimorphism. The male specific MGC is located at the entry of the antenna and exclusively processes pheromone signals."
While this statement is generally true, it is not "exclusively" true. There are examples within Lepidoptera where some regions of the MGC process no pheromone signals. See Trona et al., 2010 - Journal of Experimental Biology, and Namiki et al., 2008, Journal of Comparative Physiology A; both of these report on processing of non-pheromonal odorants by the MGC in respective species.
Line 90-92. "Moverover, ghost moths have undergone asymmetrical divergence of duplicated genes to deliver functional alterations in subsequent species, providing insights into the evolutionary process of Lepidoptera [21]."

It is not clear whether the correct reference is being cited here, or if it is indeed the correct reference, what the claim is referring to. The cited article does not mention ghost moths nor anything about asymmetric divergence of duplicated genes. So it is not clear how this statement is supported nor how the conclusion is reached.
Line 93 (and also on 102). "epidemic".
I don't think this is the correct word, I think it is meant to say "endemic"
Results.
Line 137-138. "This indicates that both male and female Himalaya ghost moths may keep pheromone reception with their shortened antennae".
Without actually testing the functional response profiles of neurons housed within the sensilla trichoidae of ghost moths, this is a speculative statement, and should not be presented in the results section, but instead in the discussion.
Line 140-141. "compared with other Lepidopteran moths (in general 50 to 80 glomeruli) (Figure 1E).
Where does this data come from? In addition to Figure 1E here, it is needed to refer to the relevant supplementary data file as well.
Line 149-150. "The cumulus which represents a major area involving pheromone reception"
A reference is needed for this statement. Furthermore, is it being suggested that his area involves pheromone processing in the ghost moth? Is anything known about the pheromones used in these species? This should be described in the appropriate section of the introduction.
Line 164. "A total of 23 TxiaORs were confirmed to be expressed from the genome and transcriptomes…"
Confirmed to be expressed where? In which tissues specifically?
Line 168-170. "This array, homologous to TxiaOR19, contained 16 homologues (TxiatdORs) and 18 pseudogenes (TxiatdpORs), which maintained the largest tandem duplications reported in lepidopterans (Figure s5, data s2).
Figure s5 does not show an array of tandem duplications, it just shows all of the candidate genes' exon/intron structure lined up in separate rows, some of which seem to have similar patterns, but many that do not. The representations are inexplicably named "testNN" and it is not clear the relevance of these names. To make the intended point, It would be far more useful to highlight with different colors homologous exons, and show an additional panel that diagrams their relative positions in the genome on the respective chromosome. The focus on the OR19 tandem expansion of ORs is a central point in this report. So this claim needs to be properly visualized.
Line 171-173. "The Maximum-Likelihood phylogeny analysis using 272 ORs showed that the TxiaOR19 array formed an earlier group where canonical type I PRs arose (Figure 2A, Data S3).
In looking at Figure 2A, it is not clear how well that statement is supported. The bootstrap support circle at the branchpoint between the OR19 clade and the canonical PR clade is rather small. It would be better to show the actual bootstrap support value number at this point instead of the circle representation in order to make it clearer to the reader.
Line 173-175. "This tree topology between the two clades was consistent when cross checked with both Neighbor-Joining and Bayesian methods (Figure s6).
This is true, but those trees do not appear to clearly support such close relationships between the PR clade and the OR19 clade. How can these differences be reconciled? In Figure 2A the relationship

between the two clades appears to be directly approximate, but in the Figure s6 trees, they do not.
Line 176-178. "The TxiaOR19 array suggested an earlier emergence than male-biased TxiaOR7, as it could blast to ORs in locust, aphid, soldier flu, mosquito and flea, with homologues predicted by CLANS in mosquito and flea (Figure 2C, Figure s7).
It is not clear how Figures 2C and S7 supports the claim that any TxiaORs have any homology with ORs from mosquito and flea, and it is not clear from figure 2C what data supports this claim. It would have been better to include these ORs (from mosquito and flea) in figure 2A in order to provide better support for homologous relationships between ORs across distant insect orders. It is well known that there is little evidence for homology between ORs across distant insect orders, See Hansson and Stensmyr 2011 review in Neuron, Figure 3. Solid evidence would be needed to be shown to support the claim being made, and as currently presented, that evidence is lacking.
Line 183-185. "suggesting possible evolutionary patterns similar to the duplicated zen family of orange swift moth, H. sylvina, which diverged to new functional gene families Shx"
This is a discussion point that should not be presented as results.
Line 185-186. "On the other hand, PRs could be traced back to a single LarmPR1 in the caddisfly."
What is this claim based on? It seems like it is being made from Figure 2D. However, it is not clear that anything shown in Figure 2D supports the claim. LarmPR1 does not seem to be connected or tracing to any other ORs in that figure.
Line 190-191. "Specifically, TxiaOR19 array arose from the LarmOR19-OR13a tandem (Figure 2E)."
What is the statistical support (Bootstrap values or Likelihood Ratio or other) for this claim? It does not seem to be apparent in the figure, so it is difficult to assess how well supported the phylogenetic relationships are.
Line 241-243. "which fits the predicted non-PR functioning of TxiaOR19 to a non-canonical female emission and the olfactory architectural observations."
What functional assays were done to show that TxiaOR19 does or does not function as a PR? Is this based on the molecular docking predictions shown in figure S8? If that is the case, it appears in that figure that the male-biased OR7 shows higher affinity to the female-specific emitted compound, Oleamide. This would seem to point to the potential for OR7 being a PR. How can the claim made here be reconciled with what is shown in Figure S8?
Discussion
Line 251-253. "We have also discovered that both males and females of these moths possess a compact olfactory system with distinct structures involved in sexual recognition"
What is the basis for this claim? Which structures? It was not shown that any structures are involved in sexual recognition in the ghost moths examined in this report. I would suggest to be careful about making assumptions about homologous structures from higher moths while at the same time trying to argue in favor of great distinctions in the ancestral ghost moths compared to the higher moths. It is better to present data about the function of the structures of the ghost moth instead.
Line 266-267. "OR19 duplications in these ghost moths do not appear to have enhanced functions"
What does this mean? Almost no evidence was presented demonstrating the function of these ORs.
Line 294. "These cues also suggest the ancestral roles of the ghost moths in terms of their sex role systems."
It is not clear what is meant by this.

Figures and Data
Figure S8. "or head space volatiles (blue)"
Are these blue traces both from females, or something else? It should be clarified in the legend.
Table S4. Concerning Cydia pomonella, Wan et al., 2019 reported 85 ORs, 65 GRs and 39 IRs (see page 4), so it is not clear what is being referred to here for 82 ORs, 15 IRs and 1 GR.

Methods

Are the methods appropriate to the aims of the study, are they well described, and are necessary controls included? Choose an item.

Conclusions

Are the conclusions adequately supported by the data shown? Choose an item.

Reporting Standards

Does the manuscript adhere to the journal's guidelines on minimum standards of reporting? Choose an item.

Choose an item.

Statistics

Are you able to assess all statistics in the manuscript, including the appropriateness of statistical tests used? Choose an item.

Quality of Written English

Please indicate the quality of language in the manuscript: Choose an item.

Declaration of Competing Interests

Please complete a declaration of competing interests, considering the following questions:

- Have you in the past five years received reimbursements, fees, funding, or salary from an organisation that may in any way gain or lose financially from the publication of this manuscript, either now or in the future?
- Do you hold any stocks or shares in an organisation that may in any way gain or lose financially from the publication of this manuscript, either now or in the future?
- Do you hold or are you currently applying for any patents relating to the content of the manuscript?
- Have you received reimbursements, fees, funding, or salary from an organization that holds or has applied for patents relating to the content of the manuscript?

- Do you have any other financial competing interests?
- Do you have any non-financial competing interests in relation to this paper?

If you can answer no to all of the above, write 'I declare that I have no competing interests' below. If your reply is yes to any, please give details below.

I declare that I have no competing interests.

I agree to the open peer review policy of the journal. I understand that my name will be included on my report to the authors and, if the manuscript is accepted for publication, my named report including any attachments I upload will be posted on the website along with the authors' responses. I agree for my report to be made available under an Open Access Creative Commons CC-BY license (http://creativecommons.org/licenses/by/4.0/). I understand that any comments which I do not wish to be included in my named report can be included as confidential comments to the editors, which will not be published.

Choose an item.

To further support our reviewers, we have joined with Publons, where you can gain additional credit to further highlight your hard work (see: https://publons.com/journal/530/gigascience). On publication of this paper, your review will be automatically added to Publons, you can then choose whether or not to claim your Publons credit. I understand this statement.

Yes Choose an item.
