# Supplementary material for: A ghost moth olfactory prototype of the lepidopteran sex communication
Source: Gigascience. 2024 Jul 19;13:giae044. doi: 10.1093/gigascience/giae044 (PMC11258902; doi:10.1093/gigascience/giae044)
Supplement: giae044_Reviewer_1_Report_Revision_1 — William B. Walker, Ph.D. -- 5/2/2024 Reviewed [file giae044_reviewer_1_report_revision_1.pdf]

Reviewer Report

Title: A ghost moth olfactory prototype of the lepidopteran sex communication

Version: Revision 1 **Date:** 5/2/2024

Reviewer name: William B. Walker, Ph.D.

Reviewer Comments to Author:

The manuscript is substantially improved after the previous round of revisions and most of the reviewer comments have been addressed. However, reading the updated manuscript in this new light has brought to the forefront several other minor concerns that must be addressed before the manuscript should be published. Furthermore, while the manuscript is generally written very well, numerous small language errors are noticeable throughout the text. It is recommended to ensure the manuscript is revised for language after all scientific edits are made.
Minor revisions are suggested as follows:
Introduction
Line 94-96. "Moreover, ghost moths have undergone asymmetrical divergence of duplicated genes to deliver functional alterations in subsequent species, proving insights into the evolutionary process of Lepidoptera."
Which genes? Is this a widespread phenomena across all different kinds of genes, or is this referring to specific genes or gene families? Please clarify.
Results
Line 211-212. "In all, the genomic backgrounds and receptor repertoires are specific in T. xiaojinensis, which may confer to the structural specificities described above."
It is not clear what this statement means. How are these features specific in T. xia, and what structural specificities are being referred to.
Line 234-235. "possible functional drift of TxiaOR19."
Please describe what is meant here more clearly with this term. Functional drift in what manner, or relative to what?
Line 241-244. "The results showed that TxiaOR19 had less binding affinity towards the panel of the ghost moth emissions, and its responding spectrum were relatively broad, indicating that ghost moth T. xiaojinensis may show unconventional courtship behaviors comparing to higher moths."
Might this result also suggest that other ORs, especially within the OR19 tandem array may be instead tuned to ghost moth emissions? Why aren't docking profiles examined for other OR19 sub-family receptors? In any case, this kind of statement seems more like a discussion statement than a results statement. I understand it is used to transition to the next paragraph, however, the logic for looking at behavior in males and females (as it turns out, non-dimorphic behavior), could just as well follow from the lack of sexual dimorphism in the antennal lobe, perhaps much better, as compared to the hypothetical ligand docking studies, which are far from complete.
Discussion.
Line 256. "Himalaya ghost moths offer a basal model for the study of olfaction evolution in insects"

I am not sure it is correct to say "insects" here. It may be more correct instead to say "Lepidoptera" or at the very most, the "Lepidoptera/Trichoptera" sister clades, since caddisflies are also included….but not more generally insects.

Line 278-279. "However, more experimental functional evidence needs to be provided for both TxiaOR19 and TxiaOR7…"

Again, here, shouldn't this refer to the TxiaOR19 sub-family/array ORs more generally, instead of only TxiaOR19?

Line 282. "the TxiaOR19 array and LarmPR1 share motifs that reflect some exons on the loci"

It's not clear what this means. How do the motifs reflect exons? Which exons and which loci?

Line 282-283. "These motifs were separated before the evolution of Lepidoptera."

This is a vague statement. Do you mean before Lepidoptera and Trichoptera diverged from each other, or before the ghost moths split off from the more recently evolved moths? Please clarify.

Line 286-287. "On the other hand, the shared motif regions could be traced back to earlier dipteran species"

Which shared motif regions? Are you referring to the ones in the PRs as shown in Fig 2C/2D/2E? Or motifs present in other organisms, as mentioned in the previous sentence?

Line 297-298. "Enlarged MGCs in butterfly Piers rapae suggest that the sexual dimorphic sex pheromone recognition system is widely used by Lepidoptera"

Please check the species, as Piers rapae is not at all mentioned in the cited article, no. 49. I would also further caution against making this statement about the sexually dimorphic sex pheromone recognition system, as the authors of the cited article emphasize that their butterfly MGC is not implied to be homologous to the moth MGC.

Materials and Methods.

Section on Scanning electron Microscopy.

For the washes, dehydration and drying steps, please specify the length of times for each of these steps.

Line 376. Change pollutions to contamination.

Figures and Tables

Figure 2, Panels C-E. It is unclear why Motifs 1-3 are shown in 2C, while Motifs 4-6 are shown in 2D. And in 2E, motif 2 appears very similar to Motif 4, while Motif 3 appears very similar to Motifs 5 and 6. Based on this last point, the text from lines 204-211 is confusing, especially considering that in 2C and 2D, for the PR consensus representations, either Motifs 1-3 are shown in 2C or Motifs 4-6 are shown in 2D. If this is the PR consensus, and it is the same representation for 2C and 2D, shouldn't Motifs 1-6 be shown for both? Please try and re-write the relevant text in the main manuscript and figure legend so that this concept is clearer.

Figure 3, Panel A. It is not clear where the burst events are. Are they marked by the red arrowheads? This should be written out clearly in the figure legend.

Figure S2. What do the black squares and grey bars represent? Please indicate their meaning in the figure legend.

Figure S3. It appears that different sensilla types are present in the various different panels. Please indicate, with arrows and notations, in each panel, which sensilla type are the sensilla trichodea, as it is not obvious to an unfamiliar reader.

Figure S8. It appears that only 18 genes are shown here. It should be made clear in the figure legend

that these are the intact homologues and the pseudogenes are excluded, if that is indeed the case. Table S2. The title of this table has been updated to say "comparison of chemosensory genes in head among the tested Lepidoptera. Is this referring to head only? Or is it an indication of head/antennal expression? Furthermore, in the previous round of revision, it was mentioned that the numbers for C. pomonella were inaccurate and they have been updated to reflect all ORs/GRs/IRs identified in the genome, as reported by Wan et al., 2019. However, now that the title has changed to reflect chemosensory genes in head, the numbers for C. pomonella are again inaccurate, as they reflect presence of these genes in the genome, not in the head.

Methods

Are the methods appropriate to the aims of the study, are they well described, and are necessary controls included? Choose an item.

Conclusions

Are the conclusions adequately supported by the data shown? Choose an item.

Reporting Standards

Does the manuscript adhere to the journal's guidelines on minimum standards of reporting? Choose an item.

Choose an item.

Statistics

Are you able to assess all statistics in the manuscript, including the appropriateness of statistical tests used? Choose an item.

Quality of Written English

Please indicate the quality of language in the manuscript: Choose an item.

Declaration of Competing Interests

Please complete a declaration of competing interests, considering the following questions:

- Have you in the past five years received reimbursements, fees, funding, or salary from an organisation that may in any way gain or lose financially from the publication of this manuscript, either now or in the future?
- Do you hold any stocks or shares in an organisation that may in any way gain or lose financially from the publication of this manuscript, either now or in the future?

- Do you hold or are you currently applying for any patents relating to the content of the manuscript?
- Have you received reimbursements, fees, funding, or salary from an organization that holds or has applied for patents relating to the content of the manuscript?
- Do you have any other financial competing interests?
- Do you have any non-financial competing interests in relation to this paper?

If you can answer no to all of the above, write 'I declare that I have no competing interests' below. If your reply is yes to any, please give details below.

I declare that I have no competing interests.

I agree to the open peer review policy of the journal. I understand that my name will be included on my report to the authors and, if the manuscript is accepted for publication, my named report including any attachments I upload will be posted on the website along with the authors' responses. I agree for my report to be made available under an Open Access Creative Commons CC-BY license (http://creativecommons.org/licenses/by/4.0/). I understand that any comments which I do not wish to be included in my named report can be included as confidential comments to the editors, which will not be published.

Choose an item.

To further support our reviewers, we have joined with Publons, where you can gain additional credit to further highlight your hard work (see: https://publons.com/journal/530/gigascience). On publication of this paper, your review will be automatically added to Publons, you can then choose whether or not to claim your Publons credit. I understand this statement.

Yes Choose an item.
