# Supplementary material for: A ghost moth olfactory prototype of the lepidopteran sex communication
Source: Gigascience. 2024 Jul 19;13:giae044. doi: 10.1093/gigascience/giae044 (PMC11258902; doi:10.1093/gigascience/giae044)
Supplement: giae044_Reviewer_2_Report_Original_Submission — Yi-Ming Weng, Ph.D -- 1/24/2024 Reviewed [file giae044_reviewer_2_report_original_submission.pdf]

Reviewer Report

Title: A ghost moth olfactory prototype of the lepidopteran sex communication

Version: Original Submission **Date:** 1/24/2024

Reviewer name: Yi-Ming Weng, Ph.D

Reviewer Comments to Author:

The manuscript entitled "A ghost moth olfactory prototype of the lepidopteran sex communication" is reviewed. This is a highly multidisciplinary work and difficult for me (and maybe to many other researchers) to review. Based on my expertise of insect genomics and molecular adaptation, I am providing my comments mainly on the related sections (i.e. genome and transcriptome assembly, orthology analyses, and gene evolution). In general, this is publishable material from my point of view, with some revision required to improve the manuscript. Here I list two of my major concerns and some minor suggestions.

Main concerns:

1. For the sensilla distribution analysis (Figure 1D), I feel the result is potentially biased by the different ways of counting the sensilla trichoidae in the retrieved studies. For example, Gargi et al. were counting all the sensilla trichoidae on the entire antenna, while the authors possibly divided the number by number of flagellum segments to represent the "mean" number of sensilla trichoidae of each flagellum to make it comparable with the data matrix generated in the current study. But this is assuming that all the flagellum segments have similar number of sensilla trichoidae, which is unlikely to be true. Ideally, counting all the sensilla trichoidae is required to perform the statistic analyses. My suggestion is to not include this section in the manuscript as it is not necessary for authors to derive their conclusions.
2. The completeness assessment of the genome assembly of T. armoricanus is not available. So does the transcriptome of A. jianchuanensis. These are very crucial as if the assembly less complete, the subsequent analyses will be confounded. My suggestion is to run BUSCO on the genome and transcriptome assembles.
3. I think the figures are too complicated for most readers. Too packed figures make it unpleasant to read but the role of the figures should be helping readers to quickly get the key information. For example, Figure 1B, C, D, F, G, and H; Figure 2B, C, E, H, and G; Figure 3B and C, can be separated figures in SI.

Minor suggestions:

1. Line 112 (results): I was expecting to see the assembly report of T. armoricanus as well, but it seems missing in the result sections.
2. Line 136: maybe address why H. cunea was selected for the comparison.
3. Line 165: not sure what the "57 annotations" means here.
4. Line 167: what is LG14?
5. Line359-360: please describe how the short read sequences were generated
6. Line 361-362: please provide more information about how the non-insect contigs were identified.
7. Line384: not sure what it means by "second generation genome" since there is no previous version of

genome assembly for the species. \
8. Line 406-407: What I know about orthofinder is to get the orthologs from protein sequences. Here it says "the orthologous genes of these 18 insect species were inferred from their genomes or transcriptomes" but those are DNA sequences not proteins.
9. Line425: I thought this should be protein-protein searching, but the queries are DNA, this is somewhat confusing me.
10. Line430: please provide more information about how the ORs were verified by PCRs.

Methods

Are the methods appropriate to the aims of the study, are they well described, and are necessary controls included? Choose an item.

Conclusions

Are the conclusions adequately supported by the data shown? Choose an item.

Reporting Standards

Does the manuscript adhere to the journal's guidelines on minimum standards of reporting? Choose an item.

Choose an item.

Statistics

Are you able to assess all statistics in the manuscript, including the appropriateness of statistical tests used? Choose an item.

Quality of Written English

Please indicate the quality of language in the manuscript: Choose an item.

Declaration of Competing Interests

Please complete a declaration of competing interests, considering the following questions:

- Have you in the past five years received reimbursements, fees, funding, or salary from an organisation that may in any way gain or lose financially from the publication of this manuscript, either now or in the future?
- Do you hold any stocks or shares in an organisation that may in any way gain or lose financially from the publication of this manuscript, either now or in the future?
- Do you hold or are you currently applying for any patents relating to the content of the manuscript?

- Have you received reimbursements, fees, funding, or salary from an organization that holds or has applied for patents relating to the content of the manuscript?
- Do you have any other financial competing interests?
- Do you have any non-financial competing interests in relation to this paper?

If you can answer no to all of the above, write 'I declare that I have no competing interests' below. If your reply is yes to any, please give details below.

I declare that I have no competing interests

I agree to the open peer review policy of the journal. I understand that my name will be included on my report to the authors and, if the manuscript is accepted for publication, my named report including any attachments I upload will be posted on the website along with the authors' responses. I agree for my report to be made available under an Open Access Creative Commons CC-BY license (http://creativecommons.org/licenses/by/4.0/). I understand that any comments which I do not wish to be included in my named report can be included as confidential comments to the editors, which will not be published.

Choose an item.

To further support our reviewers, we have joined with Publons, where you can gain additional credit to further highlight your hard work (see: https://publons.com/journal/530/gigascience). On publication of this paper, your review will be automatically added to Publons, you can then choose whether or not to claim your Publons credit. I understand this statement.

Yes Choose an item.
